# Metagenomic analysis reveals crosstalk between gut microbiota and glucose-lowering drugs targeting the gastrointestinal tract in Chinese patients with type 2 diabetes: a 6 month, two-arm randomised trial

**DOI:** 10.1007/s00125-022-05768-5

**Published:** 2022-08-05

**Authors:** Xiuying Zhang, Huahui Ren, Cuiling Zhao, Zhun Shi, Li Qiu, Fangming Yang, Xianghai Zhou, Xueyao Han, Kui Wu, Huanzi Zhong, Yufeng Li, Junhua Li, Linong Ji

**Affiliations:** 1 Department of Endocrinology and Metabolism, Peking University People’s Hospital, Peking University Diabetes Centre, Beijing, China; 2 BGI-Shenzhen, Shenzhen, China; 3 Laboratory of Genomics and Molecular Biomedicine, Department of Biology, University of Copenhagen, Copenhagen, Denmark; 4 Department of Endocrinology, Beijing Friendship Hospital Pinggu Campus, Capital Medical University, Beijing, China; 5 Guangdong Provincial Key Laboratory of Human Disease Genomics, Shenzhen Key Laboratory of Genomics, BGI-Shenzhen, Shenzhen, China; 6 Shenzhen Key Laboratory of Unknown Pathogen Identification, BGI-Shenzhen, Shenzhen, China

**Keywords:** Glucose-lowering drugs, Gut microbiota, Type 2 diabetes

## Abstract

**Aims/hypothesis:**

The use of oral glucose-lowering drugs, particularly those designed to target the gut ecosystem, is often observed in association with altered gut microbial composition or functional capacity in individuals with type 2 diabetes. The gut microbiota, in turn, plays crucial roles in the modulation of drug efficacy. We aimed to assess the impacts of acarbose and vildagliptin on human gut microbiota and the relationships between pre-treatment gut microbiota and therapeutic responses.

**Methods:**

This was a randomised, open-labelled, two-arm trial in treatment-naive type 2 diabetes patients conducted in Beijing between December 2016 and December 2017. One hundred participants with overweight/obesity and newly diagnosed type 2 diabetes were recruited from the Pinggu Hospital and randomly assigned to the acarbose (*n*=50) or vildagliptin (*n*=50) group using sealed envelopes. The treatment period was 6 months. Blood, faecal samples and visceral fat data from computed tomography images were collected before and after treatments to measure therapeutic outcomes and gut microbiota. Metagenomic datasets from a previous type 2 diabetes cohort receiving acarbose or glipizide for 3 months were downloaded and processed. Statistical analyses were applied to identify the treatment-related changes in clinical variables, gut microbiota and associations.

**Results:**

Ninety-two participants were analysed. After 6 months of acarbose (*n*=44) or vildagliptin (*n*=48) monotherapy, both groups achieved significant reductions in HbA_1c_ (from 60 to 46 mmol/mol [from 7.65% to 6.40%] in the acarbose group and from 59 to 44 mmol/mol [from 7.55% to 6.20%] in the vildagliptin group) and visceral fat areas (all adjusted *p* values for pre–post comparisons <0.05). Both arms showed drug-specific and shared changes in relative abundances of multiple gut microbial species and pathways, especially the common reductions in *Bacteroidetes* species. Three months and 6 months of acarbose-induced changes in microbial composition were highly similar in type 2 diabetes patients from the two independent studies. Vildagliptin treatment significantly enhanced fasting active glucagon-like peptide-1 (GLP-1) levels. Baseline gut microbiota, rather than baseline GLP-1 levels, were strongly associated with GLP-1 response to vildagliptin, and to a lesser extent with GLP-1 response to acarbose.

**Conclusions/interpretation:**

This study reveals common microbial responses in type 2 diabetes patients treated with two glucose-lowering drugs targeting the gut differently and acceptable performance of baseline gut microbiota in classifying individuals with different GLP-1 responses to vildagliptin. Our findings highlight bidirectional interactions between gut microbiota and glucose-lowering drugs.

**Trial registration:**

ClinicalTrials.gov NCT02999841

**Funding:**

National Key Research and Development Project: 2016YFC1304901.

**Graphical abstract:**

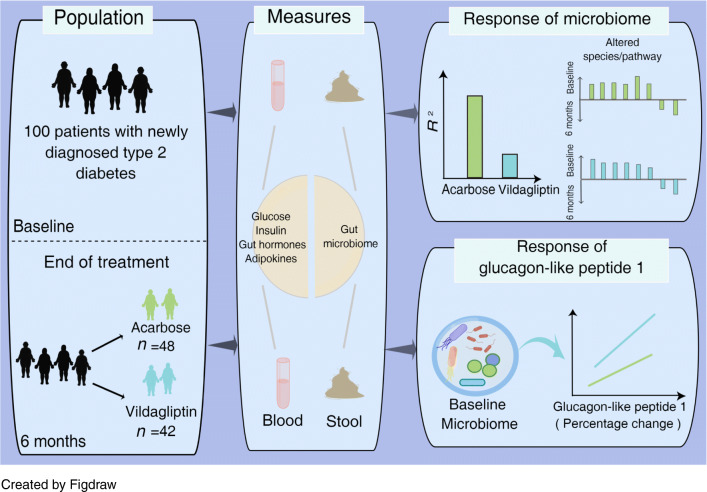

**Supplementary Information:**

The online version of this article (10.1007/s00125-022-05768-5) contains peer-reviewed but unedited supplementary material..

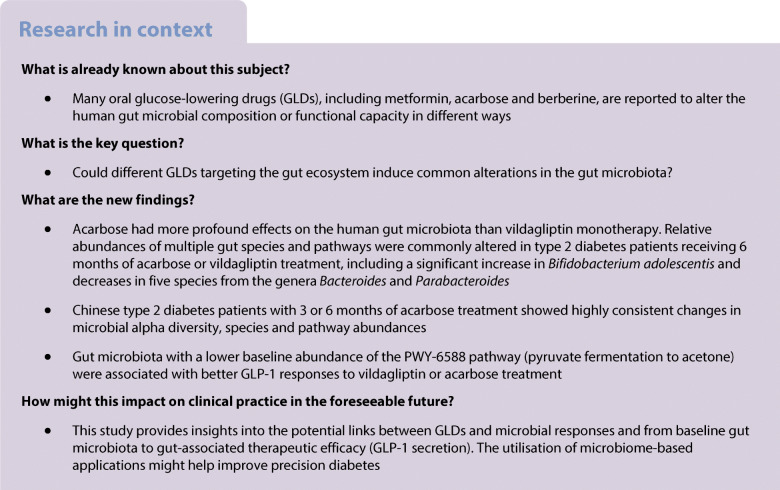



## Introduction

Over the past two decades, emerging evidence has indicated the importance of gut microbiota in maintaining host metabolic homeostasis and health [1]. Further investigations have revealed the influences of glucose-lowering drugs (GLDs) on the gut microbiota in rodents and humans, including biguanides [2–4], alpha-glucosidase inhibitors (AGIs) [5] and incretin-based drugs [6–8]. Conversely, the one exception, glipizide, a GLD of the sulfonylureas (SU) class, was reported to exhibit potent glucose-lowering effects but have no impact on the gut microbial composition in Chinese type 2 diabetes patients [5]. These results suggest that the underlying host–drug–microbe interactions but not the changes in plasma glucose levels are necessary conditions for treatment-related microbial alterations.

Different classes of medications have different targets. The SU class principally targets the ATP-sensitive potassium (K_ATP_) channels in the pancreatic beta cells [9], while several other classes are designed to treat type 2 diabetes via gastrointestinal (GI) mechanisms. For instance, AGIs mainly inhibit the degradation and absorption of complex carbohydrates in the small intestine, thus reducing postprandial glucose (PPG) [10]. Treatment with the AGI acarbose also dramatically increased gut *Bifidobacterium* abundances in type 2 diabetes patients [5]. For another example, dipeptidyl peptidase-4 inhibitors (DPP-4is) target dipeptidyl peptidase-4 (DPP-4) to decrease the clearance rate of glucagon-like peptide-1 (GLP-1) and gastric inhibitory polypeptide (GIP), two primary incretin hormones secreted by intestinal endocrine cells to stimulate insulin secretion [11]. A few studies in mice [6–8] have reported drug-induced changes in animal gut microbiota, while there are no currently available data investigating the roles of DPP-4i on the human gut microbiota. Interestingly, in addition to DPP-4is, AGI monotherapy was repeatedly reported to increase the circulating levels of active GLP-1, which might partially contribute to its therapeutic effects on glucose lowering and weight loss [12–16]. Additionally, gut microbes and specific microbial metabolites, including short-chain fatty acids (SCFAs) [17] and bile acids [18, 19], could also regulate the secretion of intestinal GLP-1, suggesting their potential influence on responses to GLDs targeting the GI tract (GIT). Although the links between GLDs and gut microbiota have been well documented in vitro and in animal models [20, 21], research gaps remain in potential crosstalk between human gut microbiota and GLP-1 and different GLDs, and their treatment responses in type 2 diabetes patients.

To narrow the knowledge gap, we conducted the VISA-T2D (Effect of Acarbose and Vildagliptin on Visceral Fat Distribution in Overweight and Obesity Patients With Newly Diagnosed Type 2 Diabetes Mellitus) study, a two-arm randomised, 6 month controlled trial, by assigning treatment-naive type 2 diabetes patients to AGI acarbose or DPP-4i vildagliptin treatment. We measured a set of clinical variables and the gut microbiota and analysed their pre- and post-changes in each treatment arm to assess the impacts of the two types of GLDs on glycaemic control, insulin and GLP-1 secretion, and weight loss, as well as the gut microbial composition. The important question was whether there are common and specific gut microbial responses to different GLDs targeting the GIT, and how host, drug and the gut microbiota interact in different arms. Finally, we included faecal metagenomes from a previous study [5], where participants were treated with 3 month acarbose or glipizide, for a parallel assessment of the impacts of different GLDs on the gut microbiota in Chinese type 2 diabetes patients.

## Methods

### Participants

In this study, we screened 30–70-year-old adults with overweight/obesity (24 kg/m^2^ ≤ BMI ≤ 30 kg/m^2^) and newly diagnosed, treatment-naive type 2 diabetes (ND-T2D) by a 75 g OGTT from December 2016 to December 2017 in Beijing, China. According to 1999 WHO criteria [22], 100 eligible individuals with ND-T2D (54 male participants and 46 female participants) and with 53 mmol/mol (7.0%) ≤ HbA_1c_ ≤ 75 mmol/mol (9%) were included. The details of exclusion criteria are available in the electronic supplementary material (ESM) Methods.

### Study design

The VISA-T2D study was designed as an exploratory, randomised, controlled, open-labelled, interventional trial (ESM Fig. 1). We estimated our sample size for this exploratory trial based on previous human gut microbial intervention studies relating to GLDs [2, 3, 5]. See the ESM Methods.

### Laboratory measurements

For each participant, anthropometric indicators including body weight (BW), height, BMI, waist and hip circumference, WHR and systolic and diastolic blood pressures (SBP and DBP, respectively) were measured by registered nurses at baseline and 6 month visits. Fasting and postprandial blood specimens were collected via venepuncture for laboratory measurements of glucose, insulin, lipids, gut hormones and adipokines. See the ESM Methods.

### Abdominal computed tomography scan and visceral fat area measurements

Quantitative computed tomography (QCT) scans were performed to measure the changes in visceral and subcutaneous fat areas (VFA and SFA, respectively). See the ESM Methods.

### Shotgun metagenomic sequencing for the VISA-T2D study cohort

Faecal specimens were self-collected at the baseline and 6 months visits in hospital using a sterile container with spoon. After sampling, all containers were rapidly placed in a cooler with dry ice and delivered to the laboratory. Approximately 200 mg (a level spoonful) of stool was then taken from the sterile container and placed into a 5 ml freezing tube by experienced technicians and stored immediately at −70°C before processing. Faecal microbial DNA extraction, shotgun sequencing and quality control (QC) of raw data were performed as previously described (ESM Table 1). See the ESM Methods.

### Gut microbiome analyses for the VISA-T2D cohort

All 181 metagenomes were processed by MetaPhlAn2 v2.7.0 [23] and HUMAnN2 v0.11.1 [24] to obtain relative abundance (RA) profiles at the taxonomic and functional levels. In total, 569 species and 469 pathways were identified (RA>0 in ≥1 faecal sample) in the VISA-T2D study cohort. We excluded rare microbial variables with a low occurrence (<20% of all samples), resulting in 117 species and 346 pathways for subsequent analysis. See the ESM Methods.

### Statistical analysis

#### Methods for comparative analysis

ANCOVA was performed to determine differences between treatment arms in clinical variables at baseline and 6 months. Before analysis, the centred log-ratio (Clr) transformation was applied to species and pathway profiles to deal with compositional bias [25]. Wilcoxon rank-sum test was used to detect between-group differences in Shannon index and Clr-transformed abundances at baseline and 6 months. Wilcoxon signed-rank test was conducted on pre–post paired samples to detect significantly altered clinical variables and microbial variables by vildagliptin and acarbose treatment, respectively. The Benjamini–Hochberg (BH) method was used to correct the multiple comparisons on clinical variables, species and pathways. A BH-adjusted *p* value <0.05 was considered significant. Permutational multivariate analysis of variance (PERMANOVA) was performed using Bray–Curtis dissimilarity at the species level to assess the inter-group microbial variations (acarbose vs vildagliptin) and the treatment-induced microbial variations (pre- vs post-treatment). See the ESM Methods.

#### Methods for association analysis

A generalised estimating equations (GEE) model using baseline and 6 months data of microbial features (Clr-transformed RAs) and clinical variables was built to assess their longitudinal associations in each treatment arm after adjustment for age and sex. A strict GEE model was followed to examine the significance of associations after adjustment for age, sex, BMI and VFA at L2-L3 intervertebral space (L2-L3 VFA). See the ESM Methods.

#### Evaluation of the relationship between baseline microbiome and GLP-1 response to treatment

We divided type 2 diabetes patients in the vildagliptin group into low (≤50.18%) and high (>50.18%) response groups based on the median value (50.18%) of the percentage change (PC%) of fasting GLP-1 (PC%-GLP-1). Next, we used sparse partial least squares discriminant analysis (sPLS-DA) to select baseline microbial variables (from 117 species and 165 pathways above the median variance) for distinguishing participants with low and high GLP-1 responses to vildagliptin. The robustness and performance of the ten selected variables were evaluated using acarbose samples as the external dataset. Spearman’s rank correlation analysis was performed to assess the relationships between the PC%-GLP-1 and baseline RAs of microbial variables, and individual predicted probabilities after adjusting for age and sex. See the ESM Methods.

#### Validation of acarbose-induced microbial changes in an external cohort

A total of 188 metagenomic datasets from a previous multicentre clinical study on Chinese individuals with ND-T2D with 3 month acarbose (51 participants, 102 samples) or glipizide (43 participants, 86 samples) treatment [5] were collected for two purposes: (1) to investigate the repeatability of microbial changes induced by acarbose; and (2) to use the glipizide group as a control treatment arm as the drug is known to specifically target pancreatic beta cells and to show no apparent impacts on gut microbiome. To keep consistency, we applied MetaPhlAn2 and HUMAnN2 with the same default settings and generated Clr-transformed RA profiles of 142 species and 256 pathways (occurrence ≥20%) for this cohort. We adopted the Wilcoxon signed-rank test for comparisons of microbial RAs between pre- and post-treatment samples, and the same GEE model (adjustment for age and sex) for assessment of longitudinal associations between microbial features and clinical variables in participants treated with 3 month acarbose or glipizide. A BH-adjusted *p* value <0.05 was considered significant.

### Ethics approval

This study was approved and conducted according to the guidance of the ethics committee of Peking University Health Science Center (2015PHB175-01) and the institutional review board of BGI (BGI-IRB 20163). Patient consent was not required for publication.

## Results

### Baseline characteristics of participants and clinical outcomes

One hundred eligible individuals with ND-T2D (54 male participants and 46 female participants) were randomly assigned to the acarbose or vildagliptin arm in a 1:1 ratio. Ninety-two participants completed the 6 month trial, including 48 participants in the acarbose arm and 44 participants in the vildagliptin arm (ESM Fig. 1). In addition, no serious drug-related adverse events were reported. At baseline, no significant differences were found in age and sex distributions between the two arms (*p*>0.05; Table 1). Baseline levels of clinical variables, including diabetes (HbA_1c_, fasting plasma glucose [FPG], PPG, fasting insulin [Fins], postprandial insulin [Pins] and HOMA-IR) and obesity (BW, BMI, and VFA and SFA at the L2-L3 and L4-L5 interspaces [L2-L3 VFA, L4-L5 VFA, L2-L3 SFA and L4-L5 SFA, respectively]) variables, blood lipids, gut hormones and adipokines, were also well balanced between groups (ANCOVA, *p*>0.05; Table 1).

**Table 1:** Demographic and metabolic characteristics of study participants

Characteristic	Baseline			6 Months			*p*^b^ (Pre vs Post)		ΔAcar vs ΔVild		
	Acar (*n*=50)	Vild (*n*=50)	*p*^a^	Acar (*n*=48)	Vild (*n*=44)	*p*^a^	Acar	Vild	ΔAcar	ΔVild	*p*^a^
Demographic characteristics											
Male/female, *n*	31/19	23/27	0.16								
Age, years	53 (9.8)	51.5 (8.8)	0.45								
Glycaemic characteristics											
HbA_1c_, mmol/mol	60.11 (6.33)	59.02 (7.07)	0.55	46.45 (6.63)	44.26 (7.47)	0.35	<0.0001*	<0.0001*	−14.46 (7.66)	−14.88 (7.13)	0.65
HbA_1c_, %	7.65 (0.58)	7.55 (0.65)	0.55	6.4 (0.61)	6.2 (0.68)	0.35	<0.0001*	<0.0001*	−1.32 (0.61)	−1.4 (0.7)	0.65
FPG, mmol/l	8.31 (1.81)	8.75 (1.33)	0.66	7.22 (1.14)	6.84 (1.27)	0.17	<0.0001*	<0.0001*	−1.36 (1.14)	−1.48 (1.27)	0.41
PPG, mmol/l	12.2 (2.31)	13.13 (2.75)	0.49	9.12 (1.72)	9.97 (2.51)	0.02	<0.0001*	<0.0001*	−3.65 (1.72)	−2.75 (2.51)	0.05
Fins, pmol/l	1.53 (1.3)	1.43 (0.83)	0.33	1.34 (0.59)	1.22 (0.73)	0.80	0.11	0.29	−0.37 (1.24)	−0.07 (0.69)	0.42
Pins, pmol/l	4.69 (3.79)	4.55 (3.11)	0.80	3.14 (2.23)	4.65 (4.3)	0.01	<0.0001*	0.86	−2.05 (3.03)	0.39 (3.15)	0.0019
HOMA-IR	4.11 (5.8)	3.9 (2.2)	0.28	2.84 (1.5)	2.78 (1.58)	0.42	0.0005*	0.0045	−2.01 (1.5)	−0.83 (1.58)	0.63
Obesity variables											
Weight, kg	73.25 (8.5)	69.75 (9.66)	1.00	70 (8.39)	68.5 (10.56)	0.39	<0.0001*	0.0039	−2.24 (8.39)	−1.33 (10.56)	0.15
BMI, kg/m^2^	26.83 (1.81)	26.83 (1.81)	0.87	26.17 (1.8)	26.33 (2.04)	0.31	<0.0001*	0.0036	−0.83 (1.8)	−0.55 (2.04)	0.14
L2-L3 VFA, cm^2^	235.9 (68.0)	211.1 (50.8)	0.09	200.9 (65.5)	191.6 (56.5)	0.46	<0.0001*	0.0005*	−28.0 (65.5)	−15.3 (56.5)	0.47
L2-L3 SFA, cm^2^	106.6 (33.6)	126.2 (37.4)	0.13	96.6 (33.9)	115.3 (37.7)	0.17	0.001*	0.07	−9.8 (33.9)	−5.43 (37.74)	0.27
L4-L5 VFA, cm^2^	189.8 (54.6)	162.7 (43.3)	0.10	153.9 (45.7)	153.2 (35.1)	0.87	0.0002*	0.0005*	−27 (45.67)	−13.7 (35.1)	0.71
L4-L5 SFA, cm^2^	157.3 (41.3)	162.6 (41.2)	0.23	144.7 (35.0)	162.1 (38.2)	0.04	0.02	0.38	−12.5 (35)	−3.2 (38.2)	0.39
Blood lipids											
CH, mmol/l	5.04 (0.87)	5.12 (0.95)	0.38	4.96 (1)	4.87 (0.88)	0.94	0.10	0.07	−0.22 (1)	−0.23 (0.88)	0.69
TG, mmol/l	1.99 (1.75)	1.97 (1.48)	0.23	1.54 (1.13)	1.71 (1.48)	0.30	0.01	0.73	−0.46 (1.13)	−0.16 (1.48)	0.23
HDL, mmol/l	1.24 (0.2)	1.26 (0.21)	0.07	1.29 (0.21)	1.26 (0.22)	0.90	0.02	0.83	0.06 (0.21)	0 (0.22)	0.18
LDL, mmol/l	2.95 (0.78)	2.85 (0.64)	0.64	2.84 (0.77)	2.76 (0.68)	0.58	0.20	0.05	−0.13 (0.77)	−0.17 (0.68)	0.90
BP											
mSBP, mmHg	127 (14.4)	129 (14.8)	0.37	126 (12.3)	127.7 (16.2)	0.13	0.17	0.35	−3 (12.3)	−2 (16.2)	0.81
mDBP, mmHg	78 (9.0)	78 (10.0)	0.69	74 (8.4)	78 (11.8)	0.15	0.03	0.92	−3 (8.4)	0.2 (12)	0.09
Gut hormones and adipokines											
Adiponectin, μg/ml	9.7 (6.5)	8.5 (7.3)	0.81	12.3 (6.4)	11.7 (6.1)	0.64	0.01	0.07	2.3 (6.4)	1.2 (6.1)	0.46
Leptin, ng/ml	11.1 (9.9)	17.6 (15.4)	0.23	5.8 (10.1)	9.2 (13.3)	0.45	0.0014*	0.03	−5.0 (10.1)	−6.7 (13.3)	0.82
GLP-1, pmol/l	3.1 (1.0)	3.1 (0.6)	0.99	3.1 (9.2)	4.9 (3.7)	0.50	0.24	<0.0001*	1.4 (9.2)	2.7 (4.0)	0.07
CCK, pg/ml	13.8 (96.8)	9.5 (18.5)	0.12	20.3 (345.7)	19.9 (46.6)	0.23	0.0013*	<0.0001*	51.2 (345.7)	18.8 (46.6)	0.48
Ghrelin, pg/ml	368 (275)	411 (316)	0.68	402 (302)	408 (264)	0.70	0.36	0.89	24 (302)	−15 (264)	0.23
PYY, pg/ml	117.4 (58.7)	133.1 (56.8)	0.61	142.9 (50.8)	127.4 (42.9)	0.07	0.17	0.10	9.3 (50.8)	−12.6 (42.9)	0.01

Continuous data are presented as mean (SD)

^a^*p* value from ANCOVA by adjusting for age, sex (continuous data) or χ^2^ test (categorical data)

^b^*p* value from paired Wilcoxon rank-sum tests (continuous data)

*BH-adjusted *p* value <0.05

Acar, the acarbose treatment group; CH, total cholesterol; Δ(Delta), pre–post changes in variables in each treatment group; mDBP, mean DBP; mSBP, mean SBP; TG, triglycerides; Vild, the vildagliptin treatment group

**Fig. 1:**
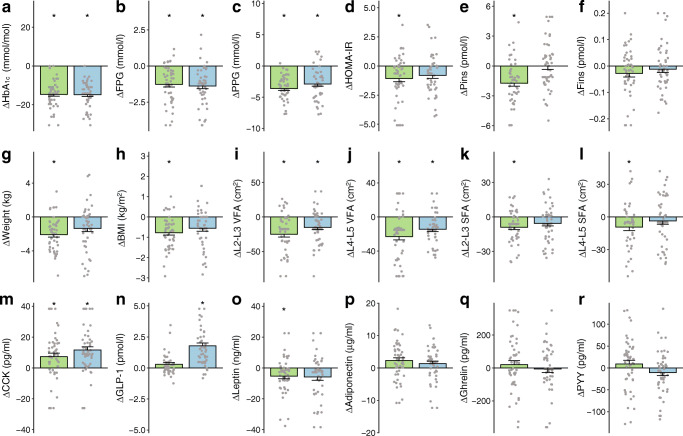
Major clinical outcomes in newly diagnosed type 2 diabetes patients after 6 month treatment with acarbose or vildagliptin. (**a**–**r**) Bar charts show changes in HbA_1c_ (**a**); FPG (**b**); PPG (**c**); HOMA-IR (**d**); Pins (**e**); Fins (**f**); weight (**g**); BMI (**h**); L2-L3 VFA, L2-L3 SFA, L4-L5 VFA and L4-L5 SFA (**i**–**l**); CCK (**m**); GLP-1 (**n**); leptin (**o**); adiponectin (**p**); ghrelin (**q**); and PYY (**r**), in the acarbose (light green) or vildagliptin (light blue) treatment group. Wilcoxon signed-rank test, *BH-adjusted *p*<0.05. The *y*-axis indicates the delta (Δ) post-minus pre-treatment value of each variable. Data are presented as mean+SEM (detailed BH-adjusted *p* values are presented in Table 1). Individual data points are shown on the graph in grey

After 6 months of treatment, HbA_1c_ levels in the acarbose (60 vs 46 mmol/mol [7.65% vs 6.40%]) and vildagliptin groups (59 vs 44 mmol/mol [7.55% vs 6.20%]) were lowered to similar levels and both reached the recommended target [26] (HbA_1c_ < 53 mmol/mol [7%]) (Wilcoxon signed-rank test, adjusted *p*<0.05; Fig. 1a). Both drugs also significantly improved FPG and PPG (Table 1, Fig. 1b,c), as well as VFAs (L2-L3 VFA and L4-L5 VFA) (Fig. 1i,j). Participants treated with acarbose had significant reductions in Pins, HOMA-IR and BW (adjusted *p*<0.05; Fig. 1d–g), and the latter two variables were moderately improved by vildagliptin (*p*<0.05 and adjusted *p*>0.05; Table 1)*.* The Pins**-**lowering effect of acarbose was also significantly superior to that of vildagliptin (ANCOVA, adjusted *p*<0.05; Table 1). Additionally, the changes over time in insulin and glycaemic variables were more highly correlated in the acarbose (*p*<0.05) than in the vildagliptin group (Spearman’s rank analysis; ESM Fig. 2a, b). Conversely, neither of the two drugs significantly affected Fins levels, blood lipids or blood pressures (adjusted *p*>0.05; Table 1).

When investigating gut hormones and adipokines, we showed that both drugs significantly increased serum cholecystokinin (CCK) levels (adjusted *p*<0.05; Fig. 1m), while 6 month vildagliptin specifically increased fasting active GLP-1 levels and acarbose specifically reduced fasting leptin levels (Fig. 1n,o). Neither of the drugs exhibited significant impacts on fasting levels of adiponectin or two gut hormones involved in appetite regulation, ghrelin and peptide YY (PYY) (adjusted *p*>0.05; Fig. 1p–r) [27, 28]. Despite similar glycaemic efficacy, our results suggest that the two drugs could benefit metabolic variables, gut hormones and adipokines in different ways.

### Responses of human gut microbiota to different GLDs

To better understand the impacts of different types of GLDs on human gut microbiota and the relationships between GLDs, microbiota and drug actions, we performed subsequent analyses on faecal samples from the VISA-T2D study (6 month acarbose or vildagliptin) and the previous study (3 month acarbose or glipizide) [5] using the same pipelines (see ESM Methods).

**Fig. 2:**
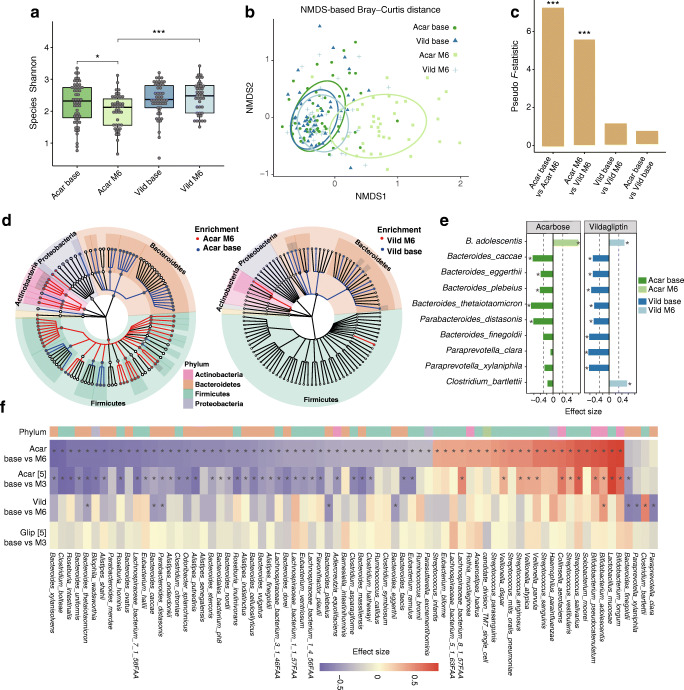
Changes in the gut microbial structure induced by glucose-lowering treatment. (**a**) Comparisons of alpha diversity (Shannon index at the species level) between four groups. Wilcoxon signed-rank test for comparisons between pre- and post-treatment groups with the same agent; Wilcoxon rank-sum test for comparisons between groups with acarbose or vildagliptin, *p<0.05, ***p<0.001. (**b**) Non-metric multidimensional scaling (NMDS) plot illustrating the Bray–Curtis dissimilarities of the gut microbial species composition in pre- and post-treatment samples. (**c**) Bar plots of pseudo *F*-statistic values showing the magnitudes of microbial dissimilarities for within- and between-treatment groups. PERMANOVA (*N*=999 permutations), ***p<0.001. (**d**) Taxonomic cladogram showing significantly altered microbial taxa in patients treated with 6 months of acarbose or vildagliptin (Wilcoxon signed-rank test, BH-adjusted *p*<0.05; see details in ESM Tables 2, 3). (**e**) Gut microbial species consistently respond to acarbose and vildagliptin treatment. Wilcoxon signed-rank test, *BH-adjusted *p*<0.05. Colour bars indicate pre–post treatment effect sizes estimated from Wilcoxon signed-rank tests on the Clr-transformed RAs of species in the two treatment groups. Effect size >0: dark green and dark blue indicate the higher RAs in pre-treatment groups with acarbose (Acar base) and vildagliptin (Vild base), respectively; effect size ≤0: light green (Acar M6) and light blue (Vild M6) indicate the higher RAs in post-treatment groups. The effect size is calculated as the *Z* statistic divided by the square root of the sample size. The dashed line indicates an absolute value of effect size at 0.3. (**f**) Heatmap showing significantly altered species in four treatment arms, including 6 month treatment with acarbose (Acar base vs M6; *n*=42) or vildagliptin (Vild base vs M6; *n*=41) in the current study, and 3 month treatment with acarbose (Acar base vs M3; *n*=51) or glipizide (Glip base vs M3; *n*=43) in a previous study of Chinese type 2 diabetes patients [5]. The colour key indicates pre–post treatment effect sizes. Wilcoxon signed-rank test, * indicates BH-adjusted *p*<0.05. Acar, acarbose; Vild, vildagliptin

No significant inter-group differences were observed in alpha diversity, beta diversity and species abundances for baseline samples in the current study (Wilcoxon rank-sum test, *p*>0.05; Fig. 2a,c) (ESM Table 2). Six months of acarbose but not vildagliptin led to significant decreases in microbial alpha diversity of type 2 diabetes patients (Wilcoxon signed-rank test, *p*<0.05; Fig. 2a). Acarbose also induced significant changes in the overall gut microbial composition (PERMANOVA for Bray–Curtis distance, *p*<0.05; Fig. 2b,c, ESM Fig. 3). Conversely, vildagliptin treatment showed no statistically significant impacts on microbial alpha or beta diversity (*p*>0.05; Fig. 2a–c, ESM Fig. 3). At the taxonomic level, we identified that the RAs of 76 and ten species were altered significantly by 6 month acarbose and vildagliptin monotherapy, respectively (adjusted *p*<0.05; Fig. 2d,e, ESM Tables 3, 4). At the functional level, acarbose significantly altered the RAs of 115 pathways (adjusted *p*<0.05) and vildagliptin only had moderate impacts on 51 pathways (*p*<0.05 and adjusted *p*>0.05) (ESM Tables 3, 4). In addition, acarbose induced more considerable changes in the structure of species–species co-occurrence networks (pre vs post, correlations for hub scores of species, Spearman’s ρ=0.33) than vildagliptin (Spearman’s ρ=0.75; ESM Fig. 4a, b, and see the ESM Methods). For instance, multiple *Streptococcus* species (e.g. *S. sanguinis* and *S. salivarius*), the most connected gut microbial taxa in the post-acarbose treatment group, exhibited significant positive associations with *Bifidobacterium longum* and negative associations with *Bacteroides* spp. (e.g. *B. caccae* and *B. stercoris*) (an absolute value of correlation coefficient >0.3; ESM Fig. 4c–e, ESM Table 5). Despite the differences in baseline gut microbial composition between the two acarbose study cohorts (ESM Fig. 5a, b), we demonstrated that 3 or 6 months of acarbose treatment consistently induced significant changes in RAs of 47 species and 39 functional pathways (BH-adjusted *p*<0.05 in both groups; Fig. 2f, ESM Fig. 5c, d, ESM Tables 3, 6), including the previously reported decreases of diversity and multiple *Bacteroides* species and the increases of *Bifidobacterium* and *Streptococcus* members [5]. Additionally, we also detected many species with differential abundances between acarbose and vildagliptin groups at 6 months, which largely overlapped the acarbose-induced changes (ESM Table 7). These results all supported the greater impacts of acarbose on human gut microbial diversity and ecological structures.

Notably, we found that participants receiving different drugs exhibited consistent changes in RAs of a set of gut microbial species and functional pathways. For instance, there were significant enrichments in RAs of *B. adolescentis* and reductions in RAs of *Bacteroides plebeius*, *B. caccae*, *Bacteroides eggerthii*, *Bacteroides thetaiotaomicron* and *Paraprevotella distasonis* in the gut of participants treated with either single agent (adjusted *p*<0.05; Fig. 2e, ESM Tables 3, 4). When considering a moderate trend toward significance (*p*<0.05 in both arms), we showed that 16 species were commonly reduced in the two treatment arms (ESM Fig. 6a) and all belonged to the phylum Bacteroidetes. Furthermore, most of the above species responding to 6 months of acarbose or vildagliptin were altered consistently in participants treated with 3 months of acarbose (adjusted *p*<0.05; ESM Fig. 6a). Among the commonly increased taxa, two *Bifidobacterium* members (*B. adolescentis* and *B. longum*) [29–32] and *Haemophilus parainfluenzae* [33, 34] have been repeatedly shown to have significantly higher RAs in healthy control groups than in type 2 diabetes patients. Both drugs also decreased the RAs of pathways involved in the biosynthesis of queuosine (PWY-6700 and PWY-6703), *lipopolysaccharide* (PWY-1269) and pyridoxal 5′-phosphate (PLP, PWY0-845 and PYRIDOXSYN-PWY), and increased the pathways of the mixed acid fermentation (FERMENTATION-PWY) and the biosynthesis of seleno amino acid (PWY-6936) (*p*<0.05 at both arms; ESM Fig. 6b, ESM Tables 3, 4). Correlation analysis on baseline RAs (ESM Fig. 7a) and the RA changes (ESM Fig. 7b) between responding species and pathways consistently revealed positive associations between several *Bacteroidetes* species and pathways (e.g. PWY0-845 and PYRIDOXSYN-PWY for the biosynthesis of PLP, PWY-7282: 4-amino-2-methyl-5-phosphomethylpyrimidine biosynthesis and ARGININE SYN4-PWY: l-ornithine biosynthesis) which were both reduced after treatment (adjusted *p*<0.05). Cumulative abundance analysis further supported that the RAs of the above-mentioned highly correlated pathways were mainly contributed by the *Bacteroides* species (ESM Fig. 7c)*.*

In addition, vildagliptin specifically elevated the RAs of *Clostridium bartlettii*, a known Firmicutes butyrate producer [35], and reduced the RAs of *Paraprevotella clara* and *Paraprevotella xylaniphila* (adjusted *p*<0.05 in the vildagliptin group and *p*>0.05 in 3 month and 6 month acarbose groups; Fig. 2e,f) [35]. We also repeated the previous finding [5] that glipizide, an effective GLD targeting the SU receptor on pancreatic beta cells [36], did not significantly alter the RAs of any gut species or pathways (Fig. 2f, ESM Fig. 6a, b, ESM Table 6). Altogether, these results suggested the existence of common and agent-specific gut microbial responses in type 2 diabetes patients receiving acarbose or vildagliptin monotherapy.

### Longitudinal associations between microbial abundances and metabolic variables

**Fig. 3:**
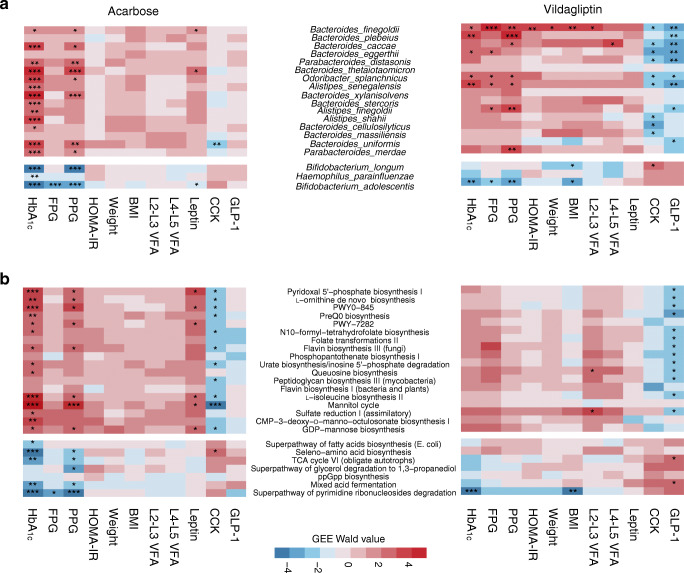
Longitudinal associations between clinical variables and microbial abundances. (**a**, **b**) Heatmaps resulting from Wald statistics of the longitudinal associations of clinical variables with the 19 species (**a**) and 25 pathways (**b**) with consistent responses to 6 month treatment with acarbose or vildagliptin. All the metabolic pathways are ranked in the same order as presented in ESM Fig. 5. Wald statistics are calculated based on multivariate regression models using GEE, adjusting for sex and age. *BH-adjusted *p*<0.05, **BH-adjusted *p*<0.01, ***BH-adjusted *p*<0.001. Blue indicates species/pathways of higher abundances in pre-treatment groups. Red indicates species/pathways of higher abundances in post-treatment groups

Given that acarbose and vildagliptin exert their glucose-lowering effects through distinct GI mechanisms, we asked how the responding microbial variables were correlated with metabolic variables during treatments by different agents. To answer this, we performed the GEE analysis and investigated the longitudinal correlations between the responding species/pathways and clinical variables, with adjustment for age and sex (see Methods). We found significant associations between HbA_1c_ and *Bifidobacterium* species (negative correlations) and a few *Bacteroidetes* species (positive correlations) in both groups (GEE, adjusted *p*<0.05; Fig. 3a). Conversely, few microbial features were correlated with obesity variables (e.g. BW, BMI and VFA) in either arm (Fig. 3a,b). There were also drug-dependent longitudinal association patterns including the PPG–microbiome associations in the acarbose group and the GLP-1–microbiome associations in the vildagliptin group (Fig. 3a,b), and most of the correlations remained significant even after adjustment for BMI and L2-L3 VFA (ESM Table 8). We also showed highly consistent association patterns between HbA_1c_/PPG and 47 acarbose-altered microbial species in the 3 month and 6 month cohorts (GEE, BH-adjusted *p*<0.05; ESM Fig. 8a, b). By contrast, no significant associations were found in the glipizide-treated type 2 diabetes patients between these species and any clinical variables (ESM Fig. 8c).

### Associations between baseline gut microbiota and post-treatment GLP-1 responses

**Fig. 4:**
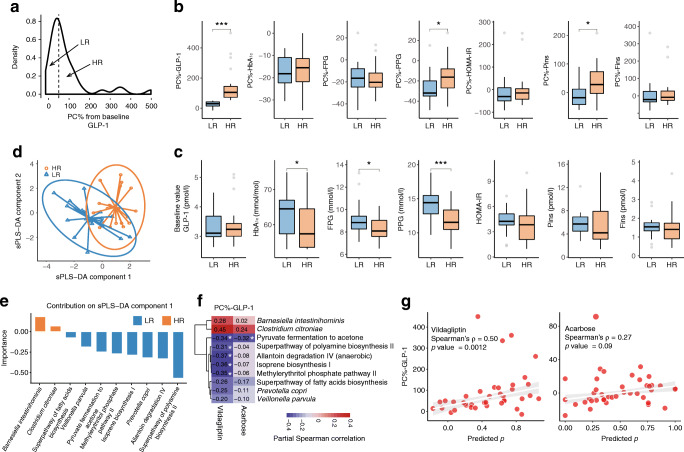
Links between baseline gut microbiota and post-treatment GLP-1 response. (**a**) Density curve of the PC% from baseline of fasting active GLP-1 in response to vildagliptin treatment. The dotted line represents a median value of 50.18%. Low response (LR): ≤50.18%; high response (HR): >50.18%. (**b**, **c**) Boxplots showing the comparisons of PC% from baseline (**b**) and baseline values (**c**) of GLP-1 and six type 2 diabetes-related variables between the two response subgroups. The *p* values were calculated using ANCOVA with adjustment for age and sex. *p<0.05, **p<0.01, ***p<0.001. (**d**) sPLS-DA to select baseline microbial features driving the separation of samples between the two subgroups. Individual samples from different subgroups are presented on a scatter plot using different colours (LR: blue; HR: orange) and 95% confidence ellipses. (**e**) Bar plot representing the contributions of the ten selected microbial features for the first sPLS-DA component. (**f**) Heatmap resulting from the coefficients of partial Spearman correlations (adjustment for age and sex) between PC%-GLP-1 and baseline abundances of ten selected microbial features in the two groups. *p<0.05. (**g**) Scatterplots showing the correlation between the PC%-GLP-1 and the sPLS-DA-based predicted probability in the vildagliptin group and the correlation between the PC%-GLP-1 and the predicted probability in the acarbose group. The *p* values and ρ values were calculated by Spearman’s rank correlation

The secretion of GLP-1 could be directly improved by vildagliptin and specific gut bacterial metabolites, such as SCFAs and secondary bile acids [37, 38]. The latter raised the next important question: whether baseline gut microbiota had potential impacts on GLP-1 responses to drug treatments. To answer this, we divided 40 type 2 diabetes patients in the vildagliptin group (who had pre- and post-treatment metagenomes and fasting GLP-1 values) into two subgroups according to their GLP-1 responses (see Methods), namely the high response (HR, *n*=20, PC%>50.18%) and low response (LR, *n*=20, PC%≤50.18%) groups (Fig. 4a). Likewise, the HR group also had a greater improvement in Pins levels than the LR group (*p*<0.05; Fig. 4b). At baseline, the two subgroups had no significant differences in GLP-1, insulin or HOMA-IR (*p*>0.05; Fig. 4c), but the LR group had worse glycaemic status than the HR group (*p*<0.05; Fig. 4c, ESM Table 9).

We next performed sPLS-DA to investigate whether baseline microbiota (RAs of species and pathways) could effectively distinguish participants with high and low GLP-1 responses to vildagliptin. We observed a clear separation of individuals between the HR and LR subgroups in the classification model (Fig. 4d). Among the ten selected microbial variables that had the highest contribution to sPLS-DA-1, the baseline RAs of *Barnesiella intestinihominis* and *Clostridium citroniae* were enriched in the HR group while those of *Veillonella parvula*, *Prevotella copri* and all six selected pathways were enriched in the LR group (Fig. 4e, ESM Fig. 9, ESM Table 10).

Although the fasting GLP-1 did not increase significantly in the acarbose group, we observed similar correlations between the PC%-GLP-1 and baseline RAs of the ten features in the two treatment arms, including the negative associations with PWY-6588: pyruvate fermentation to acetone (*p*<0.05, Spearman’s rank correlation; Fig. 4f). There were also positive correlations between the PC%-GLP-1 and individual predicted probabilities from classification models for the two groups (vildagliptin: ρ=0.5, *p*=0.0012; acarbose, ρ=0.27, *p*=0.09) (Fig. 4g). These results suggested that the baseline gut microbiota, in turn, might impact the heterogeneity of GLP-1 secretory responses to GLDs among different type 2 diabetes patients.

## Discussion

In the VISA-T2D study, we enrolled 100 individuals with ND-T2D, conducted a two-arm RCT and demonstrated the effects of 6 month initial monotherapy with acarbose or vildagliptin on the clinical outcomes, the gut microbiota and their mutual relationships. We reported that participants receiving either drug exhibited promising improvements in not only glycaemic control (HbA_1c_ <53 mmol/mol [7%]) but also abdominal VFA. The reduction in abdominal VFA, a major risk factor for cardiometabolic diseases, is in keeping with the reduced incident cardiometabolic diseases in individuals with type 2 diabetes/prediabetes treated with AGIs or DPP-4i [16, 39, 40].

Monotherapy with either drug had impacts on gut microbial composition, but acarbose exhibited a more profound influence on the gut ecosystem than vildagliptin. We also demonstrated highly similar microbial changes in Chinese type 2 diabetes patients treated with 3 month [5] or 6 month acarbose. Our data highlight the consistency of results between the two datasets and suggest that the identified microbial changes were likely induced as early as 3 months and persisted at least until 6 months. The moderate effect of 6 month vildagliptin treatment on the human gut microbiota, however, differed considerably from the existing findings using rodent models [6, 7, 41, 42]. For example, Liao et al reported that DPP-4i (sitagliptin) intervention significantly altered the gut microbial composition of mice with high-fat diet and its effect was more pronounced than that of acarbose [7]. In addition, Liao et al [7] observed increased *Bacteroides* while Wang et al [42] reported decreased *Bacteroides* taxa in the DPP-4i-treated mice. These contrasting findings have led to growing attention to potential confounding variables in animal-based microbiome studies, such as age, sex, strains, diets and suppliers [43].

Despite the differences in GI mechanisms for glycaemic control, we revealed that the two drugs could induce similar changes in gut microbial composition, especially the increases in *B. adolescentis* and the decreases in multiple species belonging to the phylum Bacteroidetes (*B. caccae* and *Bacteroides finegoldii*). Similarly, Wu et al reported a significant improvement in the growth rate of *B. adolescentis* in Swedish individuals treated with metformin [2]. A 3 day metformin intervention study of Chinese type 2 diabetes patients reported drug-induced decreases in *Bacteroides* spp. and microbial genes encoding bile salt hydrolases (BSHs) [3]. In addition, our previous cross-sectional study also found increased abundances of the above two *Bacteroides* species in treatment-naive type 2 diabetes patients compared with individuals with prediabetes or normal glucose tolerance [33]. These data have provided insights into the possible existence of GLD-induced reductions in *Bacteroides* spp., and these changes might contribute to alterations of the host bile acid pool, and consequently modulate the human metabolism. In addition to the known glucose-lowering effects, all three medicines are reported to effectively increase GLP-1 concentrations and reduce BW [12, 44], and the GIT has been considered a major target organ of the above drugs. By contrast, the GLD glipizide, which reduced glucose via a non-GIT mechanism, had shown no significant impacts on the human gut microbiota [5], suggesting that the drug-induced changes in host glucose levels had little or no relation to drug-induced changes in gut microbial composition. Further efforts are needed to answer whether non-glucose therapeutic benefits of different GLDs could cause common microbial changes, or whether specific microbial variables might serve as therapeutic targets and contribute to improved metabolic outcomes.

Last, we showed the classification performance of baseline gut microbiota in distinguishing patients with low and high fasting GLP-1 secretory responses to treatments. Importantly, we demonstrated that baseline abundances of microbial variables selected by the vildagliptin-based sPLS-DA model had similar associations with GLP-1 responses to acarbose treatment. In both treatment groups, worse GLP-1 responses were associated with a higher baseline abundance of the pathway PWY-6588: pyruvate fermentation to acetone. These findings support the roles of gut microbiota in modulating host GLP-1 secretion and highlight the clinical potential of microbiota-based patient stratification for diabetes precision medicine.

There are some limitations to this study. First, no placebo group was included in this two-arm study. Therefore, we were not able to accurately determine the clinical effectiveness and the degree of microbial impacts of the different drugs. However, this limitation could be partially eliminated as previous studies have revealed that the placebo effect of GLD therapies led to nonsignificant changes in HbA_1c_ and weight loss [45] and microbial composition in type 2 diabetes patients [2, 46]. Second, we did not observe significantly elevated fasting GLP-1 levels after 6 months of acarbose treatment. This was inconsistent with studies that reported improved releases of GLP-1 by acarbose, particularly the postprandial levels, in ND-T2D patients or healthy individuals [12–14]. However, we did not measure postprandial gut hormones and could not investigate possible links between such therapeutic benefits and gut microbiota. Future well-designed trials of larger groups of type 2 diabetes patients and highly related multi-omics data are needed to draw a clear mechanistic picture of possible crosstalk from drugs to gut microbial responses and from baseline gut microbiota to drug actions and their impacts on host metabolic health, and to accelerate the microbiome-based applications in diabetes treatment.

## Supplementary information

ESM (PDF 2652 kb)

## Data Availability

Metagenomic sequence data of the 181 faecal DNA samples from the VISA-T2D cohort have been deposited at the National Centre for Biotechnology Information BioProject Database with the dataset accession number PRJNA826552.

## Abbreviations

AGI: Alpha-glucosidase inhibitor
BH: Benjamini–Hochberg
BW: Body weight
CCK: Cholecystokinin
Clr: Centred log-ratio
DBP: Diastolic blood pressure
DPP-4i: Dipeptidyl peptidase-4 inhibitor
Fins: Fasting insulin
FPG: Fasting plasma glucose
GEE: Generalised estimating equations
GI: Gastrointestinal
GIT: GI tract
GLP-1: Glucagon-like peptide-1
HR: High response
L2-L3 SFA: SFA at L2-L3 intervertebral space
L4-L5 SFA: SFA at L4-L5 intervertebral space
L2-L3 VFA: VFA at L2-L3 intervertebral space
L4-L5 VFA: VFA at L4-L5 intervertebral space
LR: Low response
ND-T2D: Newly diagnosed, treatment-naive type 2 diabetes
GLD: Glucose-lowering drug
PC%: Percentage change
PC%-GLP-1: Percentage change of fasting GLP-1
PERMANOVA: Permutational multivariate analysis of variance
Pins: Postprandial insulin
PLP: Pyridoxal 5′-phosphate
PPG: Postprandial glucose
PYY: Peptide YY
RA: Relative abundance
SBP: Systolic blood pressure
SCFA: Short-chain fatty acid
SFA: Subcutaneous fat area
sPLS-DA: Sparse partial least squares discriminant analysis
SU: Sulfonylureas
VFA: Visceral fat area
VISA-T2D: Effect of Acarbose and Vildagliptin on Visceral Fat Distribution in Overweight and Obesity Patients With Newly Diagnosed Type 2 Diabetes Mellitus
