# Supplementary material for: Metagenomic analysis reveals crosstalk between gut microbiota and glucose-lowering drugs targeting the gastrointestinal tract in Chinese patients with type 2 diabetes: a 6 month, two-arm randomised trial
Source: Diabetologia. 2022 Aug 5;65(10):1613–26. doi: 10.1007/s00125-022-05768-5 (PMC9477956; doi:10.1007/s00125-022-05768-5)
Supplement: Supplementary file 1 — (PDF 2652 kb) [file 125_2022_5768_MOESM1_ESM.pdf]

Metagenomic analysis reveals crosstalk between gut microbiota and glucose-lowering drugs targeting the gastrointestinal tract in Chinese patients with type 2 diabetes: a 6 month two-arm, randomised trial

Zhang *et al.*

ESM Methods

Subjects

In this study, we screened 30-70 years-old overweight/obese (24kg/m^2 ≤ BMI≤ 30kg/m^2) adults with newly diagnosed, treatment-naïve T2D (NDT2D) by a 75-g oral glucose tolerance test (OGTT) from December 2016 to December 2017 in Beijing, China. According to 1999 WHO criteria[1], 100 eligible NDT2D patients (54 males and 46 females) with 53 mmol/mol (7.0%) ≤ HbA_{1c} ≤ 75 mmol/mol (9%) were included in the current study. Participants were excluded if they had one of the following: fertile women who were pregnant, breast-feeding, or willing to conceive; type 1 diabetes mellitus or other types of diabetes mellitus, gastrointestinal diseases, infectious liver diseases (hepatitis B and hepatitis C); endocrine diseases affecting glucose metabolism; psychiatric disorders; heavy drinker; neoplasia; or any other severe disease that could affect the outcomes of the study. None of the participants had used (taken/injected) medicines/food belonging to antibiotics, hormones, or probiotics at least one month before the initiation of the study. None of the participants had a treatment history for weight loss, dyslipidemia, hyperglycemia, or hypertension prior to this study. The protocol was approved by the ethics committee of Peking University People's Hospital and was conducted in compliance with the Declaration of Helsinki. The study was registered at ClinicalTrials.gov (NCT02999841). All participants provided informed consent.

Study design

The VISA-T2D study was designed as an exploratory, randomized, controlled, open-labeled, two-arm interventional trial without a placebo arm (**ESM Fig. 1**). The sample size for this exploratory trial (50 patients per treatment group) was determined based on previous human gut microbial intervention studies relating to glucose-lowering drugs (GLDs) (ranging from 22 to 51 subjects per treatment group)[2–4]. Briefly, one hundred newly diagnosed overweight/obese T2D patients were recruited for the study and were randomly assigned to the acarbose or vildagliptin group in a 1:1 ratio using computer-generated numbers sealed in opaque envelopes : (1) Acarbose group (n=50): acarbose (Bayer Medical and Health Co., Ltd., 50mg/tablet) starting at 50mg TID, chewed with the first meal, added to 100mg TID daily in the third week, aiming to minimize the incidence of common gastrointestinal side-effects of acarbose (e.g., flatulence and abdominal

cramps); (2) Vildagliptin group (*n*=50): vildagliptin (Swiss Novartis Limited, 50mg/tablet), 50mg BID (100mg daily), taking before meals. All participants were provided with lifestyle advice by the investigators at baseline, 3-month, and 6-month (endpoint) visits according to the recommendations of the Chinese Diabetes Guidelines (2013)[5]. For each participant, anthropometric indicators including body weight (BW), height, body mass index (BMI), waist, hip, waist-hip ratio (WHR), systolic (SBP) and diastolic blood pressures (DBP) were measured by registered nurses at baseline (pre) and 6-month (post) visits. Blood specimens were collected via venipuncture at the fasting and postprandial (2-hour after a routine 75g OGTT) status for laboratory measurements of glucose, insulin, lipids, gut hormones, adipokines, and other biochemical variabless. Abdominal CT scans were performed to measure the changes in adipose tissues pre- and post-antidiabetic treatments[6]. Urine and stool specimens on a fasting state were self-collected at the baseline and 6-month visits. Before stool sampling, sampling operations and precautions were informed to patients to avoid unnecessary contamination (e.g., urine or environmental samples). The urine specimens were used for routine tests, and the stool specimens were stored immediately at -70 °C for shotgun metagenomic sequencing.

Laboratory measurements

Hemoglobin A1c (HbA_{1c}) was measured using the cation-exchange high-pressure liquid chromatography (HPLC) method (Adams A1c HA-8160; Arkray, Kyoto, Japan). Plasma glucose levels (fasting glucose, FPG; postprandial glucose, PPG) were measured by the glucose oxidase method. Fasting serum lipid profiles including total cholesterol (CH), triglycerides (TG), high-density lipoprotein (HDL) and low-density lipoprotein (LDL) were measured using an automatic biochemical analyzer. Plasma concentrations of fasting insulin (Fins) and postprandial 2-hour insulin (Pins) were determined by electrochemiluminescence immunoassays (Elecsys 2010 system, Roche Diagnostics Ltd, Basel, Switzerland). The Homoeostasis Model of Insulin Resistance (HOMA-IR) score was calculated using the following formula: HOMA-IR = (FPG [mmol/L] × insulin [μU/mL]) /22.5. Serum concentrations of gut hormones (active glucagon-like peptide-1, GLP-1; ghrelin; and peptide YY, PYY) and two adipokines (adiponectin and leptin) were measured using standard ELISA kits at fasting state (EGLP-35K, EZGRT-89K, EZHPYYT66K,

MERCK-Millipore, USA). Serum cholecystokinin (CCK) was measured by the radioimmunoassay method (RK-069-04, Phoenixpeptide, USA).

Abdominal CT scan and VAT measurements

Non-enhanced abdominal CT scans were performed using a 64-slice multidetector scanner (LightSpeed VCT, General Electric Healthcare, Milwaukee, WI, USA). With a quantitative computed tomography (QCT) calibration phantom (Image analysis, Inc., USA) be placed beneath the participants, the computed tomography was acquired (120 kVp, 120-150 mA, and 5 mm thickness) in the supine position from the lung base to the pubic symphysis. The raw images were transferred to a QCT workstation (Mindways software, Inc., Austin, TX, USA). Measurements of areas (cm^2) of abdominal fat were performed at two slices (L2-L3 and L4-L5 lumbar intervertebral space) by two qualified physicians using Mindways QCT Pro 5.0 software according to previous literature[6]. For QCT scanning of each intervertebral space, the software positioned a closed spline at the margin between subcutaneous fat and abdominal muscle on each one mm-thick slice. Adipose tissue was segmented and mapped in blue color with a default threshold. The total (TFA) and visceral fat areas (VFA) were semi-automatically measured and exported to excel format using the Tissue Composition Module of the Mindways software. The subcutaneous fat area (SFA) was equal to TFA minus VFA. The VFA and SFA at the L2-L3 and L4-L5 interspaces were collected at the baseline and 6-month visits for each participant.

Shotgun metagenomic sequencing for the VISA-T2D study cohort

Fecal samples were collected at baseline and six months after treatment with acarbose or vildagliptin (ESM **Fig. 1**). Microbial DNA extraction from 181 fecal samples was performed using the MagPure Fast Stool DNA KF Kit B[7]. DNA nanoball (DNB) based DNA library construction and combinatorial probe-anchor synthesis (cPAS) based shotgun metagenomic sequencing with 100bp paired-end (PE) reads were applied to all samples (MGI, Shenzhen, China). A previously developed quality control (QC) workflow was used to filter low-quality and human reads[8]. On average, 88.7 M (± 5.6 M) high-quality, non-human reads were generated per sample after QC (ESM **Table 1**).

Gut microbiome analyses for the VISA-T2D cohort

Taxonomic profiling of the non-human high-quality reads was generated at four taxonomic ranks (phylum, family, genus, and species) using MetaPhlAn2[9] (version 2.7.0) with the default parameters. Shannon index (within-sample alpha diversity) and Bray-Curtis dissimilarities (between-sample beta diversity) at the species level were calculated to assess the inter-group differences at baseline and post-treatment groups, and the pre-post differences in the gut microbiota of T2D patients receiving different agents (R, package 'coin', v1.3-1). The HUMAnN2[10] (v0.11.1) pipeline was used for functional pathway profiling on metagenomic samples. Alpha-diversity for each sample was calculated on species RA using the Shannon index (R, package 'vegan', v2.5-6). A total of 569 species and 469 pathways were identified (RA>0 in at least one fecal sample) and microbial variables with a low occurrence (<20% in all samples) were considered as rare variables and excluded from analysis. We kept 117 species and 331 pathways for the VISA-T2D study cohort.

Statistical analysis

Methods for comparative analysis.

Comparisons of clinical variabless and microbial features between different groups Before group comparisons/association analyses, the centered log-ratio transformation (Clr) [11]was applied to the original RAs of microbial taxa and pathways to deal with zero values and reduce possible false-positive identification of differential microbial features. Analysis of covariance (ANCOVA) was performed to determine whether differences exist in clinical variables between two treatment groups at baseline and 6 months, respectively (**Table 1)**. Wilcoxon rank-sum test was used to detect differences in microbial Shannon index (at the species level) and Clr-transformed RAs between two groups at baseline and after treatment. The Benjamini–Hochberg (BH) method was used to correct the multiple comparisons on clinical variabless (ANCOVA), Clr-transformed RAs of species (n=117) and pathways (n=331) (Wilcoxon rank-sum test) between different groups. A BH adjusted P-value < 0.05 was considered significant.

Paired comparisons for pre-and post-treatment samples in the VISA-T2D cohort Wilcoxon signed-rank test was conducted on paired samples before and after antidiabetic treatment to detect significantly altered clinical variabless and gut microbial variables (alpha diversity, Clr-transformed

RA of species and pathways) in T2D patients treated with 6-month acarbose or vildagliptin, (**Table 1**; **Fig. 1a-d**; **Fig. 2a, d-f**). A BH-adjusted P-value < 0.05 was considered significant and a P-value <0.05 was considered a tendency towards statistical significance. Multiple testing corrections were applied based on the number of microbial species (*n*=117 for the VISA-T2D study) and pathways (*n*=331 for the VISA-T2D study). The effect size for Wilcoxon signed-rank test was estimated to indicate the magnitude and direction of differences (function *WilcoxonPairedR*, R package '*rcompanion*', v2.3.25).

To compare the degrees of post-treatment changes in clinical variables of different measurement scales, we normalized the changes of each variable to its baseline values. For each individual, percentage change from baseline of a given variable was calculated using the following equation:

$$Percentage\ change\ _{variables} = \frac{M6_{variables} - Base_{variables}}{Base_{variables}} * 100\%$$

Where $\text{Base}_{\text{variable}}$ and $\text{M6}_{\text{variable}}$ are values of the given variable from the same individual at baseline and 6-month in acarbose or vildagliptin group, respectively. ANCOVA was followed to determine whether any differences exist in percentage change of clinical variables between two treatment groups **(Table 1)**. A BH adjusted P-value < 0.05 was considered statistically significant. Spearman's rank correlation was used to measure statistical dependence among the values of percentage change in multiple clinical variables **(ESM Fig. 2)**.

Methods for association analysis

Association analyses between antidiabetic treatments and gut microbiota in two clinical cohorts

Permutational multivariate analysis of variance (PERMANOVA) was performed using Bray-Curtis dissimilarity at the species level (R, package '*vegan',* v2.5-6, function '*adonis*', permutations = 999) to assess the inter-group microbial variations at baseline and 6 months (acarbose vs. vildagliptin), and the treatment-induced microbial variations (pre- vs. post-treatment) in the same treatment arm. The magnitude of the overall compositional differences was evaluated by pseudo-F-statistic. A P-value < 0.05 was considered statistically significant. Non-metric multidimensional scaling (NMDS) was followed to visualize Bray-Curtis dissimilarities between different groups.

To evaluate how and to what extent GLDs can alter the ecological properties of the gut microbial communities in T2D patients, we performed network analysis for each group using Spare Correlations for Compositional data algorithm (SparCC) [12] at baseline and 6 months, respectively (R, package *'SpiecEasi'*, v1.0.7, function '*sparcc*'). A total of 117 species with a prevalence greater than 20% were included. To ensure the stability of correlation measurements in groups with a small sample size (n <=50), we used a bootstrap resampling method to create 50 samples per group with replacement and repeated the sampling procedure 100 times. The mean SparCC correlation coefficient between every two species was calculated based on 100 replicate measurements. To construct a binary-valued adjacency matrix, we converted the absolute coefficients (mean values) of 0.3 and higher values to 1 (defined as strong correlations), and the rest smaller values were converted to 0. We calculated multiple ecological variables including numbers of edges and nodes, density and average path of network, power-law degree of a scale-free network, and hub-score of each species to assess the topological differences in microbial networks (R, package '*igraph*', v1.2.4.2) (ESM **Fig. 4, ESM Table 5)**.

Generalized estimated equations (GEE) model was built to assess longitudinal associations between pre-post microbial profiles (Clr-based RAs of species and pathways) and pre-post absolute values of clinical variables in each treatment arm of the VISA-T2D study (6-month acarbose and vildagliptin), after adjustment for age and sex (R, package '*geeM*', v0.10.1)[13]. The GEE model for individual species/pathway and each clinical variable is shown below:

$$Clr(Species/Pathway)_{RA} \sim \beta 1\ clinical\ variable + \beta 2\ sex + \beta 3\ age + \epsilon$$

Where $\beta 1, \beta 2\ and\ \beta 3$ indicates the individual regression coefficient of clinical variable, sex and age, respectively; and ϵ indicates error term. The Wald statistic and P-value of the regression coefficient of the given clinical variable ($\beta 1$) were calculated to measure the significance and direction of its associations with species. A BH adjusted P-value < 0.05 is considered statistically significant.

A strict GEE model was followed to examine the significance of associations after adjustment for age, sex, BMI and L2-L3 VFA.

$$Clr(Species/Pathway)_{RA} \sim \beta 1\ clinical\ parameter + \beta 2\ BMI + \beta 3\ \mathrm{L2\_3}VFA + \beta 4\ sex + \beta 5\ age + \epsilon$$

Where $\beta 1, \beta 2, \beta 3, \beta 4$ and $\beta 5$ indicates the individual regression coefficient of clinical variable, sex, age, BMI and L2-L3 VFA, respectively; and ϵ indicates error term. The Wald statistic and P-value of the regression coefficient of the given clinical variable ($\beta 1$) were calculated to measure the significance and direction of its associations with species. A BH adjusted P-value < 0.05 is considered statistically significant.

Evaluation of the relationship between baseline microbiome and GLP-1 response to treatment in the VISA-T2D study cohort We divided patients into the low (≤ 50.18%) and high (>50.18%) response groups based on the median value (50.18%) of the percentage change of GLP-1 in the vildagliptin group **(Fig. 4a)**. ANCOVA was used to determine whether differences exist in baseline values and percentage changes in clinical variables between two response groups **(ESM Table 8).** An ANCOVA P-value < 0.05 was considered significant. Wilcoxon rank-sum test was used to detect the differences in Clr-transformed RA profiles of microbial species and pathways between response groups at baseline.

Next, we used sparse partial linear square discriminant analysis (sPLS-DA) to investigate whether the baseline gut microbiota (species and functional pathways) can distinguish T2D patients with low and high GLP-1 response to vildagliptin, the key therapeutic effect of DPP4i. Prior to the analysis, we excluded gut microbial pathways with low variance (below median pathway variance) to reduce the number of pathways for modeling, resulting in 165 pathways for the sPLS-DA analysis. Subsequently, sPLS-DA was conducted to select the most discriminative microbial variables (top 10) that can separate samples from different response groups and visualize the observations on a 2-dimensional map. A 95% confidence ellipse was plotted for each group using the R function '*plotIndiv*'. The performance of the sPLS-DA-based classification model was estimated using the receiver operator characteristic curve (ROC). The importance of microbial variables (4 species and 6 pathways, **Fig. 4e**) was calculated using the function '*plotLoadings*' to indicate their discriminating power (contribution) on separating two groups. All analyses in this section were performed with R package '*mixOmics*' (version 6.10.9)[14]. Further, we applied Least absolute shrinkage and selection operator (LASSO) to evaluate the robustness of the ten microbial variables selected by sPLS-DA

(**ESM Fig. 9a, b**) (R package 'glmnet', function 'glmnet')[15]. By shrinking the coefficients toward *0*, we confirmed that eight variables (four species and four pathways) had non-zero LASSO regression coefficients.

Although the acarbose group did not show a significant increase in fasting GLP-1 after treatment, we asked whether the baseline gut microbiota had similar relationships with GLP-1 responses in this group. Therefore, we applied the above sPLS-DA-based model on the acarbose group and estimated predicted probabilities for patients with different GLP-1 responses. Spearman's rank correlation analysis was used to assess possible associations between the ten selected microbial variables, the sPLS-DA-based predicted probabilities, and the percentage change of GLP-1 in two treatment groups.

Validation of acarbose-induced microbial changes in an external cohort A shotgun metagenomic dataset including 188 fecal samples (accession number PRJEB12124, 100bp PE reads from Illumina platform, average 21.1 M reads per sample) from Chinese NDT2D patients receiving 3-month treatment with α-glucosidase inhibitor (AGI) acarbose (51 patients with 102 samples) or the sulfonylurea glipizide 43 patients with 86 samples) [4] were collected for a parallel assessment on the impacts of different GLDss on the gut microbiota of T2D patients. To keep consistent, we processed all external metagenomes using the same above-mentioned pipelines (methods) to obtain RA profiles at the taxonomic (MetaPhlAn2-based, 142 species) and functional (HUMAnN2-based, 256 pathways) levels, applied Clr transformation on RA data, and conducted association analysis between microbiota and clinical variables based on GEE models.

REFERENCE

1. (1999) Making a difference. The World Health Report 1999. Health Millions 25(4):3–5
2. Wu H, Esteve E, Tremaroli V, et al (2017) Metformin alters the gut microbiome of individuals with treatment-naive type 2 diabetes, contributing to the therapeutic effects of the drug. Nat Med 23(7):850–858. https://doi.org/10.1038/nm.4345
3. Sun L, Xie C, Wang G, et al (2018) Gut microbiota and intestinal FXR mediate the clinical benefits of metformin. Nat Med 24(12):1919–1929. https://doi.org/10.1038/s41591-018-0222-4

4. Gu Y, Wang X, Li J, et al (2017) Analyses of gut microbiota and plasma bile acids enable stratification of patients for antidiabetic treatment. Nat Commun 8(1). https://doi.org/10.1038/s41467-017-01682-2
5. Chinese Diabetes Society (2014) China T2DM Prevention and Treatment Guideline (2013). Chin J Diabetes Mellit 6(447–98)
6. Yu AH, Duan-Mu YY, Zhang Y, et al (2018) Correlation between non-alcoholic fatty liver disease and visceral adipose tissue in non-obese chinese adults: A CT evaluation. Korean J Radiol 19(5):923–929. https://doi.org/10.3348/kjr.2018.19.5.923
7. Yang F, Sun J, Luo H, et al (2020) Assessment of fecal DNA extraction protocols for metagenomic studies. Gigascience 9(7). https://doi.org/10.1093/gigascience/giaa071
8. Fang C, Zhong H, Lin Y, et al (2018) Assessment of the cPAS-based BGISEQ-500 platform for metagenomic sequencing. Gigascience 7:1–8
9. Truong DT, Franzosa EA, Tickle TL, et al (2015) MetaPhlAn2 for enhanced metagenomic taxonomic profiling. Nat Methods 12(10):902–903. https://doi.org/10.1038/nmeth.3589
10. Franzosa EA, McIver LJ, Rahnavard G, et al (2018) Species-level functional profiling of metagenomes and metatranscriptomes. Nat Methods 15(11):962–968. https://doi.org/10.1038/s41592-018-0176-y
11. Aitchison J (1982) The Statistical Analysis of Compositional Data. J R Stat Soc Ser B 44(2):139–160. https://doi.org/10.1111/j.2517-6161.1982.tb01195.x
12. Friedman J, Alm EJ (2012) Inferring Correlation Networks from Genomic Survey Data. PLoS Comput Biol 8(9). https://doi.org/10.1371/journal.pcbi.1002687
13. McDaniel LS, Henderson NC, Rathouz PJ (2013) Fast Pure R Implementation of GEE: Application of the Matrix Package. R J 5(1):181–187
14. Rohart F, Gautier B, Singh A, Lê Cao KA (2017) mixOmics: An R package for ‘omics feature selection and multiple data integration. PLoS Comput Biol 13(11). https://doi.org/10.1371/journal.pcbi.1005752
15. Friedman J, Hastie T, Tibshirani R (2010) Regularization paths for generalized linear models via coordinate descent. J Stat Softw 33(1):1–22. https://doi.org/10.18637/jss.v033.i01

ESM Figures

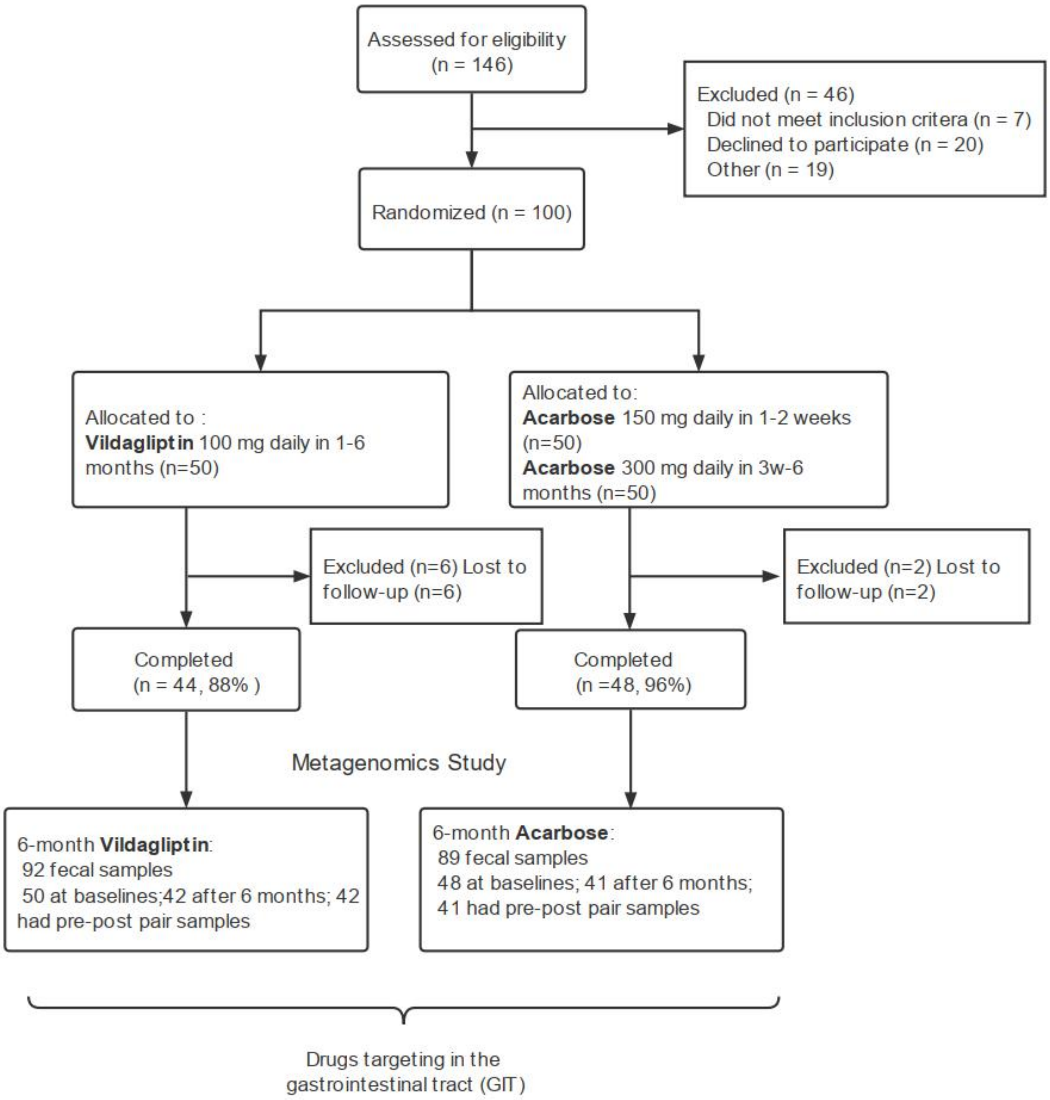

ESM Fig. 1: Flow chart of the clinical trial and metagenomic analysis of the VISA-T2D study (6-month monotherapy of vildagliptin or acarbose**).**

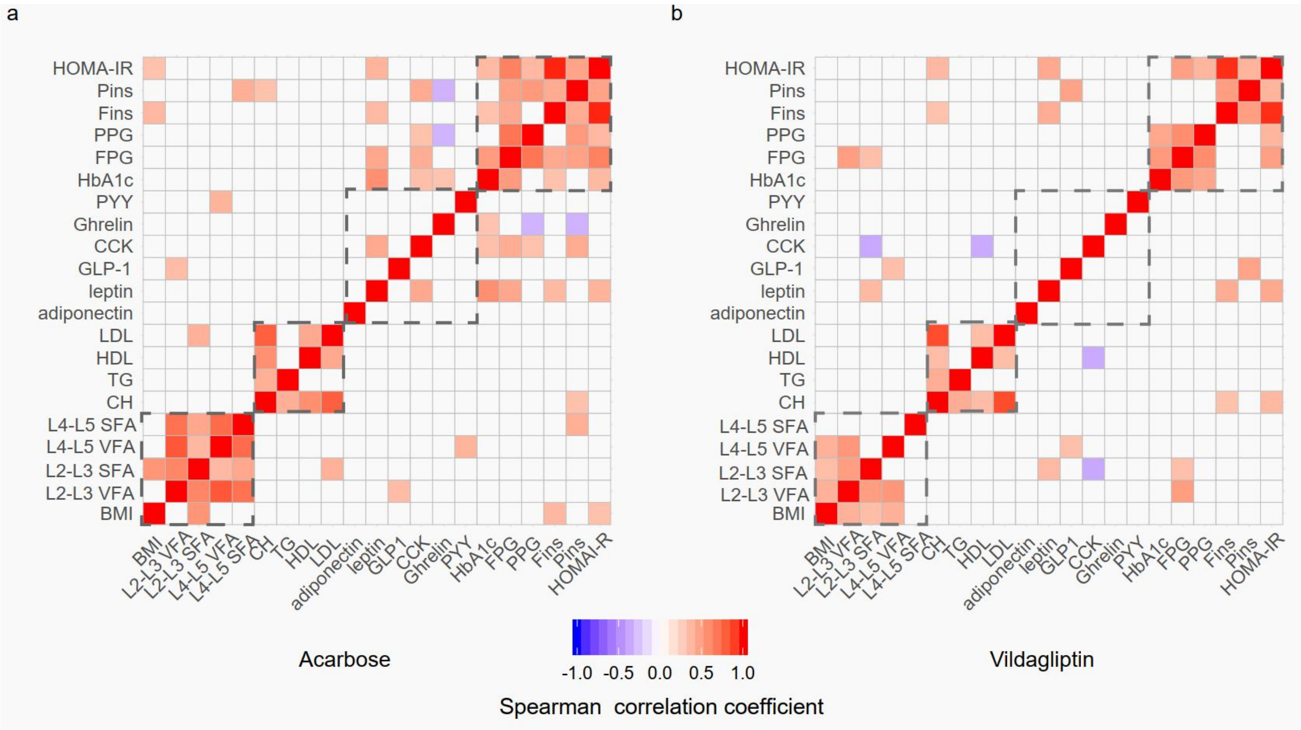

ESM Fig. 2: Correlation between the changes of clinical indices induced by Acarbose or Vildagliptin treatment.

a-b: Heatmap showing Spearman's rank correlation matrix of percentage changes of 21 selected clinical indices induced by 6-month acarbose **(a)** or vildagliptin treatment (**b**). To ensure robust comparisons between clinical indices with values measured on different scales, we normalized the treatment-induced changes of each variable to their baseline values. Colored boxes indicate the magnitude of Spearman's rank correlation coefficient (rho value): blue, $P<0.05$ and $rho<0$, significant negative correlations; white, $P>0.05$, not statistically significant; and red, $P<0.05$ and $rho>0$, significant positive correlations. Box with dashed lines indicates a group of indices related to a given metabolic condition or category, including i) diabetes (HOMA-IR, Pins, Fins, PPG, FPG, HbA_{1c}), ii) gut hormones and adipokines (PYY, ghrelin, CCK, GLP-1, leptin and adiponectin), iii) blood lipids (LDL, HDL, TG and CH) and iv) obesity (BMI, L2-L3 VFA, L2-L3 SFA, L4-L5 VFA and L4-L5 SFA).

Glycemic variables: HOMA-IR, homeostasis model assessment of insulin resistance; Pins, postprandial serum insulin; Fins, fasting serum insulin; PPG, 2-hour postprandial blood glucose; FPG, fasting plasma glucose and HbA_{1c}, glycated hemoglobin. Gut hormones and adipokines: CCK, cholecystokinin and GLP-1, Glucagon-like peptide-1. Blood lipids: LDL, low-density lipoprotein; HDL, high-density lipoprotein; TG, triglyceride and CH, cholesterol. Obese variables: L4-L5 SFA or VFA, subcutaneous fat area and visceral fat area at L4-L5 intervertebral space; L2-L3 SFA or VFA, subcutaneous fat area and visceral fat area at L2-L3 intervertebral space and BMI, Body mass index.

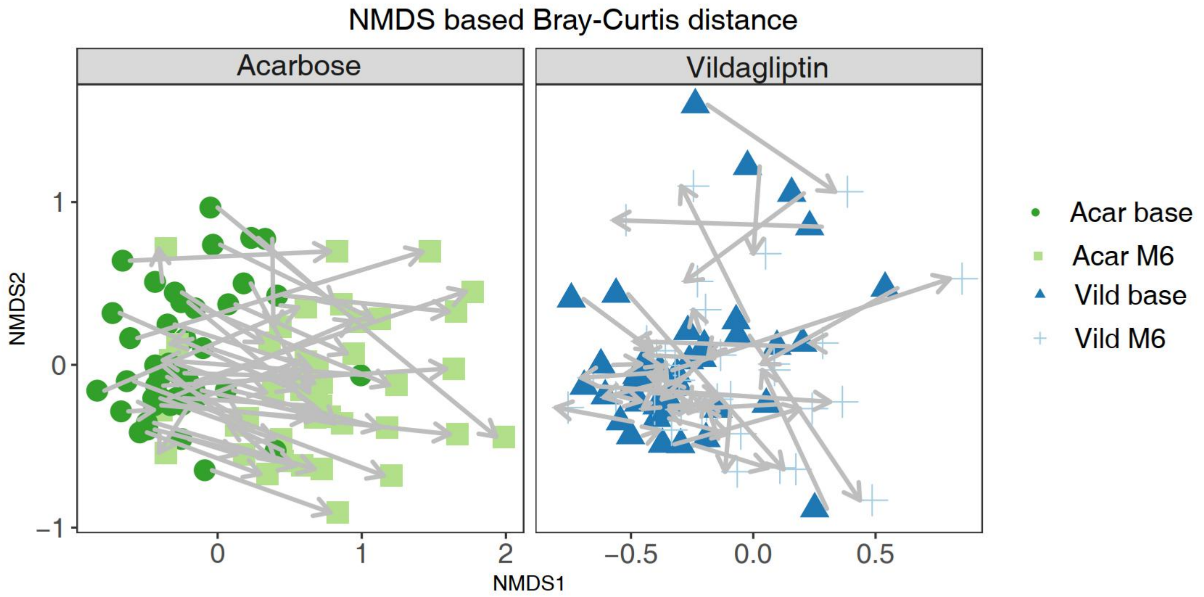

ESM Fig. 3: Shifts in gut microbial composition on patients treated with 6-month acarbose or vildagliptin

Species-based non-metric multidimensional scaling (NMDS) analysis of patients before and after 6-month treatment. Green, samples in the acarbose group, circles indicate baseline. Squares indicate 6-month. Blue, samples in the vildagliptin group, triangles indicate baseline, and crosses indicate 6-month. Arrows indicate paired samples from the same individual.

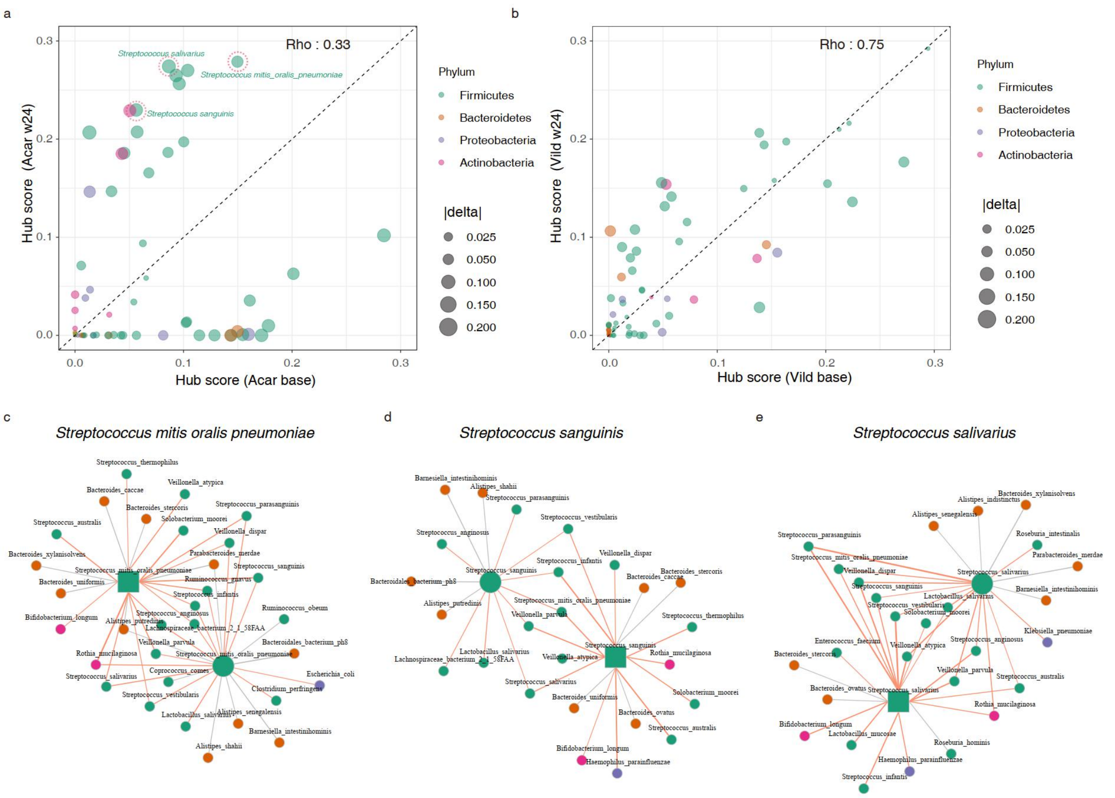

ESM Fig. 4: Microbial co-occurrence networks at baseline and after 6-month treatment with acarbose or vildagliptin

a-b: Scatter plots representing pre-and post-treatment hub scores of each species in the microbial species-species ecological network in the acarbose group (**a**) or the vildagliptin group (**b**). Spearman's rank coefficients (Rho) show the strength of correlation between paired pre-and post-treatment hub scores for each treatment group. The sizes of dots represent the absolute values of post minus pre-treatment hub score per species (|delta|). The greater the size, the greater the changes in hub score. Three *Streptococcus* species of the top maximum changed species are shown for the acarbose group.

c-e: Subnetwork for *Streptococcus mitis oralis pneumoniae* (**c**), *Streptococcus sanguinis* (**d**) and *Streptococcus salivarius* (**e**) at baseline (circle) and 6-month (rectangle) in the acarbose group. Each node represents a microbial species. Different colors represent different phyla: green, Firmicutes; orange, Bacteroidetes; pink, Actinobacteria; purple, Proteobacteria. The solid lines indicate edges between any two nodes (species) of strong correlations (light orange: positive; grey: negative), defined as an absolute value of Sparse Correlations for Compositional data (SparCC) correlation coefficient greater than 0.3.

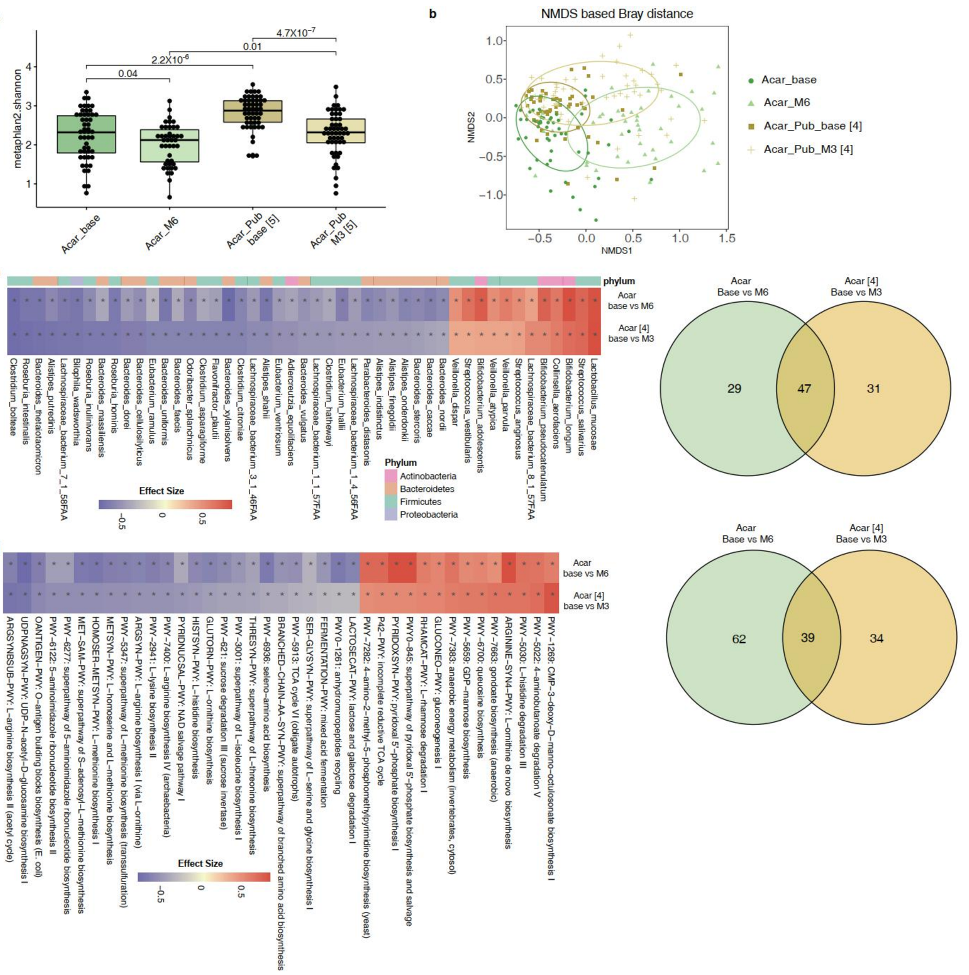

ESM Fig. 5: Assessment of consistent microbial changes induced by 3- and 6- month acarbose treatments.

a, Comparisons of alpha diversity (Shannon index at the species level) between four groups. Wilcoxon signed-rank test for comparisons between pre-and post-treatment samples in each arm; Wilcoxon rank-sum test for comparisons between different groups with 3-month (ref [4]) or 6-month acarbose.

b, Non-metric multidimensional scaling (NMDS) plot illustrating the Bray–Curtis dissimilarities of the gut microbial species composition of metagenomic samples in four groups.

c, Heatmap showing 47 consistently and significantly altered species in the two acarbose arms. Acar base vs. M6: 6-month treatment with acarbose (n=42, the current study); Acar base vs. M3: 3-month treatment with acarbose (n=51, ref [4]). Color key indicates pre-post treatment effect sizes based on Wilcoxon signed-rank test. *, BH-adjusted P-value <0.05. Venn plot showing the number of significantly altered species after 6-month (green) or 3-month acarbose treatment (yellow).

d, Heatmap showing 39 consistently and significantly altered pathways in the two acarbose arms Wilcoxon signed-rank test, *, BH-adjusted P-value <0.05. Venn plot showing the number of significantly altered pathways after 6-month (green) or 3-month acarbose treatment (yellow).

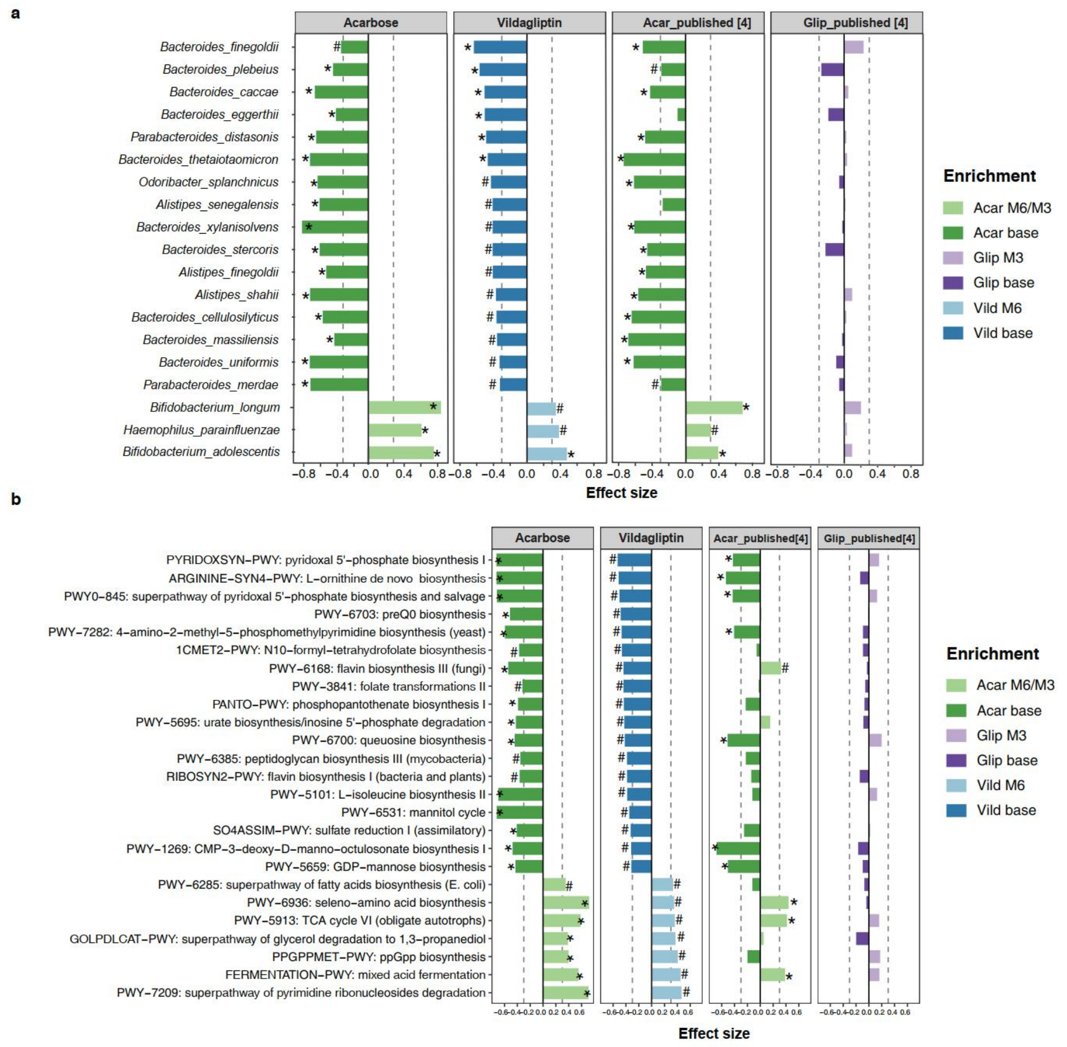

ESM Fig. 6: Gut microbial variables response consistently to 6-month acarbose and vildagliptin treatment. **a-b:** Gut microbial species (a) and functional pathways (b) response to treatment of acarbose, vildagliptin, or glipizide. Microbial variables (19 species and 25 pathways) had consistent responses (Wilcoxon signed-rank test, P-value <0.05) in both 6-month acarbose and vildagliptin groups are shown, together with their responses to 3-month acabose or glipizide (ref [4]). Color bars indicate pre-post treatment effect sizes estimated from Wilcoxon signed-rank tests on the Clr-transformed relative abundance of species or pathways in four treatment groups. #, P-value<0.05, *, BH-adjusted P-value <0.05.

Different colors indicate different response patterns: dark green, dark blue and dark purple indicate the higher effect sizes (abundances) in pre-treatment groups with acarbose (Acar base), vildagliptin (Vild base) and glipizide (Glip base), respectively; light green (Acar M6 or Acar M3), light blue (Vild M6) and light purple (Glip M3) indicate the higher effect sizes (abundances) in post-treatment groups. Dashed line indicates an absolute value of effect size at 0.3.

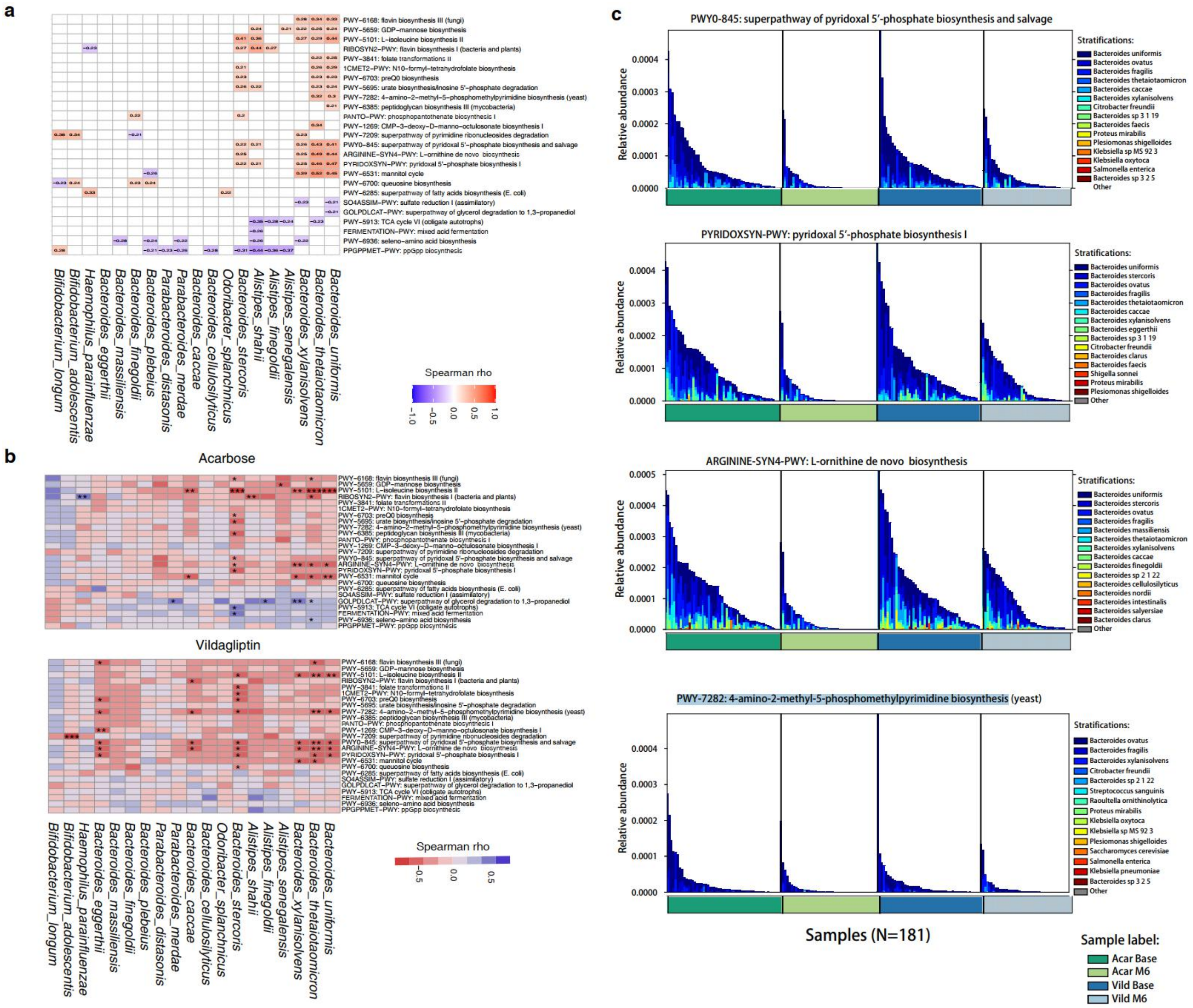

a
b
c
PWY-6168: flavin biosynthesis III (fungi)
PWY-5659: GDP-mannose biosynthesis
PWY-5101: L-isoleucine biosynthesis II
RIBOSYN2-PWY: flavin biosynthesis I (bacteria and plants)
PWY-3841: folate transformations II
1CMET2-PWY: N10-formyl-tetrahydrofolate biosynthesis
PWY-6703: preQ0 biosynthesis
PWY-5695: urate biosynthesis/inosine 5'-phosphate degradation
PWY-7282: 4-amino-2-methyl-5-phosphomethylpyrimidine biosynthesis (yeast)
PWY-6385: peptidoglycan biosynthesis III (mycobacteria)
PANTO-PWY: phosphopantothenate biosynthesis I
PWY-1269: CMP-3-deoxy-D-manno-octulosonate biosynthesis I
PWY-7209: superpathway of pyrimidine ribonucleosides degradation
PWY0-845: superpathway of pyridoxal 5'-phosphate biosynthesis and salvage
ARGININE-SYN4-PWY: L-ornithine de novo biosynthesis
PYRIDOXSYN-PWY: pyridoxal 5'-phosphate biosynthesis I
PWY-6531: mannitol cycle
PWY-6700: queuosine biosynthesis
PWY-6285: superpathway of fatty acids biosynthesis (E. coli)
SO4ASSIM-PWY: sulfate reduction I (assimilatory)
GOLPDLCAT-PWY: superpathway of glycerol degradation to 1,3-propanediol
PWY-5913: TCA cycle VI (obligate autotrophs)
FERMENTATION-PWY: mixed acid fermentation
PWY-6936: seleno-amino acid biosynthesis
PPGPPMET-PWY: ppGpp biosynthesis
Bifidobacterium_longum
Bifidobacterium_adolescentis
Haemophilus_parainfluenzae
Bacteroides_eggerthii
Bacteroides_massiliensis
Bacteroides_finegoldii
Bacteroides_plebeius
Parabacteroides_distasonis
Parabacteroides_merdae
Bacteroides_caccae
Bacteroides_cellulosilyticus
Odoribacter_splanchnicus
Bacteroides_stercoris
Alistipes_shahii
Alistipes_finegoldii
Alistipes_senegalensis
Bacteroides_xylanisolvens
Bacteroides_thetaiotaomicron
Bacteroides_uniformis
Spearman rho
Acarbose
Vildagliptin
PWY0-845: superpathway of pyridoxal 5'-phosphate biosynthesis and salvage
PYRIDOXSYN-PWY: pyridoxal 5'-phosphate biosynthesis I
ARGININE-SYN4-PWY: L-ornithine de novo biosynthesis
PWY-7282: 4-amino-2-methyl-5-phosphomethylpyrimidine biosynthesis (yeast)
Relative abundance
Stratifications:
Other
Samples (N=181)
Sample label:
Acar Base
Acar M6
Vild Base
Vild M6

ESM Fig. 7: Associations between common microbial variables responding to 6-month acarbose and vildagliptin treatment

a, Heatmap showing Spearman's rank correlations between the baseline RAs of 19 species (**X-axis, ESM Fig. 6a**) and 25 pathways **(Y-axis, ESM Fig. 6b)** across all baseline samples (N=98). Colored boxes indicate the magnitude of Spearman's rank correlation coefficient (rho value): blue, BH-adjusted $P<0.05$ and rho<0, significant negative correlations; white, $P >0.05$, not statistically significant; and red, BH-adjusted $P<0.05$ and rho>0, significant positive correlations.

b, Heatmap showing Spearman's rank correlations between the pre-post RA changes in 19 species and 25 pathways in the acarbose (upper) and vildagliptin (lower) group. The pre-post RA changes are calculated as the pre minus post-treatment values of each microbial variable.

c, Cumulative relative abundances of species contributing to selected microbial pathways. Four pathways (two microbial pathways involved in the biosynthesis of the pyridoxal 5′-phosphate: PWY0-845 and PYRIDOXSYN-PWY, ARGININE-SYN4-PWY: L-ornithine de novo biosynthesis and PWY-7282: 4-amino-2-methyl-5-phosphomethylpyrimidine biosynthesis (PWY-7282) that significantly and positively correlated with a few *Bacteroides* species are shown. Cumulative relative abundances are listed according to the contribution by annotated species at baseline and after 6-month treatment with acarbose or vildagliptin. Only the top 15 species contributing to the pathways are included.

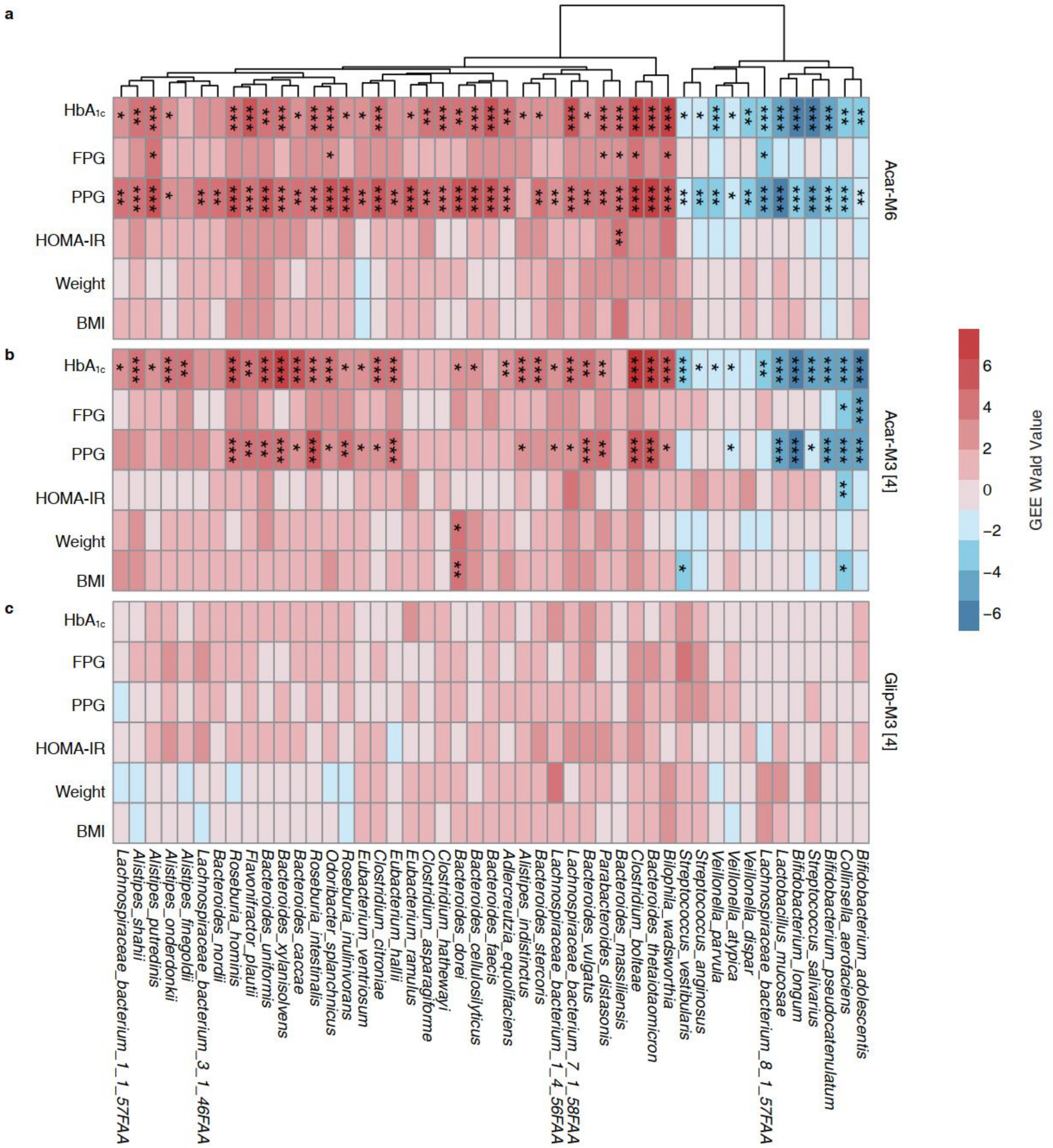

ESM Fig. 8: Longitudinal associations between metabolic variables and species abundances in T2D patients treated with acarbose or glipizide.

Heatmap resulting from Wald statistics of the longitudinal associations between metabolic variables and microbial species in 6-month acarbose (**a**), 3-month acarbose (ref [4])(**b**), or 3-month glipizide (ref [4]) (**c**) treatment group. Forty-seven species that significantly responded to both 3-month and 6-month treatments of acarbose in T2D patients from two different studies (**ESM Fig. 5**) are shown. Wald statistics are calculated based on multivariate regression models using generalized estimating equations (GEE), adjusting for sex and age. GEE, * BH-adjusted $P < 0.05$; **, BH-adjusted P<0.01； ***, BH-adjusted P<0.001.

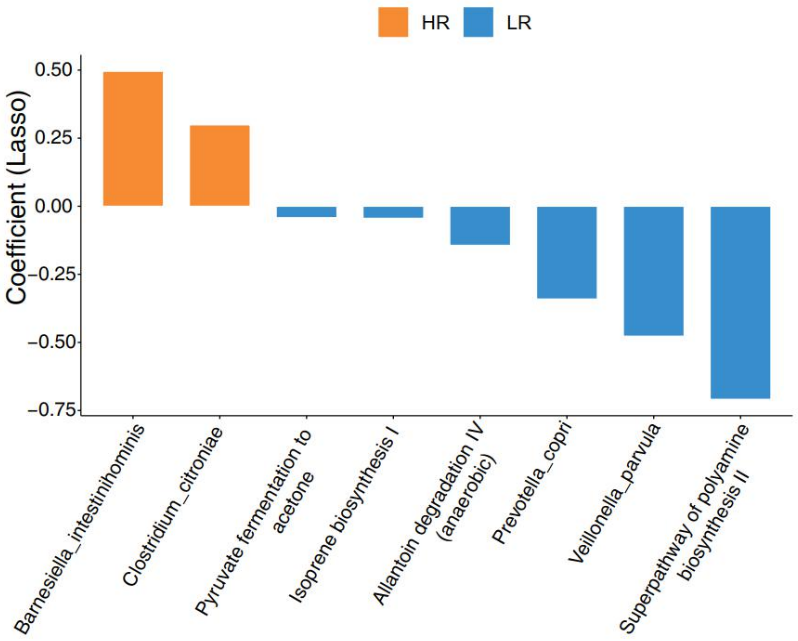

ESM Fig. 9: Evaluation of the relationships between baseline microbial variables and GLP-1 responses to vildagliptin using LASSO

Bar plot representing the LASSO (Least Absolute Shrinkage and Selection Operator) coefficients of selected microbial features by sPLS-DA (**Fig. 4**). Orange, variables enriched in the high-response (HR) subgroup; blue, variables (species or pathways) enriched in the low-response (LR) subgroup. Only eight variables with LASSO coefficients > 0 are shown.

ESM Tables

ESM Table 1. Sequencing statistics for faecal samples of the VISA-T2D study (N = 181) .

Subject ID	Nationality	# Raw Reads	# High quality reads	# Non-human clean reads	Clean reads ratio (%)
**V1-102100001**	CHN	91881722	89981205	88431604	96.25%
**V1-102100002**	CHN	92464892	90311967	88562444	95.78%
**V1-102100003**	CHN	92402170	90449394	88844386	96.15%
**V1-102100004**	CHN	92709386	90578523	88841178	95.83%
**V1-102100005**	CHN	92297044	89799698	87747214	95.07%
**V1-102100006**	CHN	94173692	92018707	90279736	95.87%
**V1-102100007**	CHN	92304924	90390886	88834504	96.24%
**V1-102100008**	CHN	93690942	91702957	90098946	96.17%
**V1-102100009**	CHN	91867290	89988005	88459650	96.29%
**V1-102100010**	CHN	92550138	90864004	89493752	96.70%
**V1-102100011**	CHN	93708204	91852768	90353280	96.42%
**V1-102100012**	CHN	92156632	90395306	88954008	96.52%
**V1-102100013**	CHN	86150716	84474500	83104534	96.46%
**V1-102100014**	CHN	92092644	90128188	88529568	96.13%
**V1-102100015**	CHN	92337428	90453173	88926182	96.31%
**V1-102100016**	CHN	92393118	90542027	89049270	96.38%
**V1-102100017**	CHN	92689828	91289466	90068952	97.17%
**V1-102100018**	CHN	92368064	90841768	89531344	96.93%
**V1-102100019**	CHN	88525798	87036204	85770866	96.89%
**V1-102100020**	CHN	92821944	90897088	89241490	96.14%
**V1-102100021**	CHN	91794112	90167540	88771440	96.71%
**V1-102100022**	CHN	92387860	90254306	88421572	95.71%
**V1-102100023**	CHN	92097010	90319153	88781248	96.40%
**V1-102100024**	CHN	92123880	90440975	89005282	96.61%
**V1-102100025**	CHN	92538420	90864593	89407158	96.62%
**V1-102100026**	CHN	92659704	90859355	89314152	96.39%
**V1-102100027**	CHN	92783546	91206600	89843876	96.83%
**V1-102100028**	CHN	93077872	91471644	90099568	96.80%
**V1-102100029**	CHN	92750928	90820216	89183272	96.15%
**V1-102100030**	CHN	91963590	90644509	89514736	97.34%
**V1-102100031**	CHN	94589698	92722693	91134248	96.35%
**V1-102100032**	CHN	92356632	90502733	88908388	96.27%
**V1-102100033**	CHN	92349396	91103146	90034132	97.49%
**V1-102100034**	CHN	92900096	91527467	90346198	97.25%

**V1-102100035**	CHN	92156552	90759374	89561872	97.18%
**V1-102100036**	CHN	92468102	91182850	90089892	97.43%
**V1-102100037**	CHN	93007260	91778239	90714508	97.53%
**V1-102100038**	CHN	92811812	91134635	89693186	96.64%
**V1-102100039**	CHN	92790212	91381857	90166858	97.17%
**V1-102100040**	CHN	92499230	90647624	89036796	96.26%
**V1-102100041**	CHN	92627728	90923591	89427316	96.54%
**V1-102100042**	CHN	92700590	90938352	89400626	96.44%
**V1-102100043**	CHN	92841268	91405615	90178812	97.13%
**V1-102100044A**	CHN	78813754	77315741	75968530	96.39%
**V1-102100045**	CHN	92794056	91234215	89870206	96.85%
**V1-102100046**	CHN	92669824	90891128	89356834	96.42%
**V1-102100047**	CHN	92711536	91343655	90176112	97.27%
**V1-102100048**	CHN	92429968	90997138	89752868	97.10%
**V1-102100049**	CHN	92754336	91026080	89534072	96.53%
**V1-102100050**	CHN	93131434	92041381	91092192	97.81%
**V1-102100051**	CHN	92703316	91262773	90023270	97.11%
**V1-102100052**	CHN	92522646	91106493	89891020	97.16%
**V1-102100053**	CHN	91546124	90350679	89313512	97.56%
**V1-102100054**	CHN	92901522	91602463	90488864	97.40%
**V1-102100055**	CHN	92995766	91661823	90496968	97.31%
**V1-102100056**	CHN	93311790	92112636	91077054	97.61%
**V1-102100057**	CHN	92945018	91694744	90617184	97.50%
**V1-102100058**	CHN	93239632	91840362	90623132	97.19%
**V1-102100059**	CHN	92559282	90976605	89590104	96.79%
**V1-102100060**	CHN	92928796	91452022	90170282	97.03%
**V1-102100061**	CHN	91456642	90117296	88942120	97.25%
**V1-102100062**	CHN	93257328	91805428	90552168	97.10%
**V1-102100063**	CHN	93034288	91746195	90608428	97.39%
**V1-102100064**	CHN	93290474	91867538	90630904	97.15%
**V1-102100065A**	CHN	92584842	91766168	91050428	98.34%
**V1-102100066**	CHN	93084626	91837995	90762554	97.51%
**V1-102100067**	CHN	92279750	91140372	90158690	97.70%
**V1-102100069**	CHN	92699018	91121974	89757656	96.83%
**V1-102100070**	CHN	92251372	91071329	90038958	97.60%
**V1-102100071**	CHN	92409436	90837533	89470570	96.82%
**V1-102100072**	CHN	92376536	90524235	88885162	96.22%
**V1-102100073**	CHN	93372652	92057824	90907244	97.36%
**V1-102100074**	CHN	92706998	91285174	90043042	97.13%
**V1-102100075**	CHN	92727908	91063493	89615292	96.64%
**V1-102100076**	CHN	93104490	91795015	90664906	97.38%

**V1-102100077**	CHN	93043008	91855251	90815210	97.61%
**V1-102100078**	CHN	92863000	91284659	89909128	96.82%
**V1-102100079**	CHN	92628244	91246637	90036084	97.20%
**V1-102100080r**	CHN	94155690	92533663	91117130	96.77%
**V1-102100082**	CHN	91673328	90410765	89298234	97.41%
**V1-102100083**	CHN	92963698	91178122	89580188	96.36%
**V1-102100084**	CHN	92897492	91388967	90078662	96.97%
**V1-102100085**	CHN	92662728	91525282	90546640	97.72%
**V1-102100086**	CHN	92485550	91462575	90577706	97.94%
**V1-102100087**	CHN	92829728	91803311	90924580	97.95%
**V1-102100088A**	CHN	92571654	91714230	90969600	98.27%
**V1-102100089**	CHN	93320982	92256188	91348286	97.89%
**V1-102100090**	CHN	92222132	91171146	90279456	97.89%
**V1-102100091**	CHN	93055240	91993952	91070848	97.87%
**V1-102100092**	CHN	92730604	91485110	90403344	97.49%
**V1-102100093**	CHN	92487888	91174048	90040534	97.35%
**V1-102100094**	CHN	92667864	91602169	90679780	97.85%
**V1-102100095**	CHN	92786184	91709793	90780074	97.84%
**V1-102100096**	CHN	92853986	91718007	90723234	97.71%
**V1-102100097**	CHN	92806172	91825809	90979440	98.03%
**V1-102100098**	CHN	92800466	91841902	91022788	98.08%
**V1-102100099A**	CHN	93447102	92433016	91541182	97.96%
**V1-102100100**	CHN	92670702	91312249	90120376	97.25%
**V8-102100001**	CHN	91896004	90557446	89368214	97.25%
**V8-102100002**	CHN	93798146	92171753	90723360	96.72%
**V8-102100003A**	CHN	92757698	91845156	91039824	98.15%
**V8-102100004**	CHN	92258638	90746746	89400634	96.90%
**V8-102100005**	CHN	92242680	90937402	89768626	97.32%
**V8-102100006**	CHN	92072266	90845784	89758456	97.49%
**V8-102100007**	CHN	94609670	93181954	91908822	97.15%
**V8-102100008**	CHN	94080490	92818592	91708550	97.48%
**V8-102100009**	CHN	92054858	90829380	89734060	97.48%
**V8-102100013**	CHN	93900248	92572863	91392786	97.33%
**V8-102100014**	CHN	92367926	91132953	90028918	97.47%
**V8-102100015A**	CHN	93132584	92003088	90994492	97.70%
**V8-102100016**	CHN	91830974	90501072	89314988	97.26%
**V8-102100017**	CHN	93697626	92428532	91300460	97.44%
**V8-102100018**	CHN	94296284	92506225	90896494	96.39%
**V8-102100019**	CHN	93423878	92015982	90763776	97.15%
**V8-102100021**	CHN	93888720	92562517	91379732	97.33%
**V8-102100023**	CHN	92623980	90424867	88400254	95.44%

**V8-102100024**	CHN	90553392	88482878	86576942	95.61%
**V8-102100025**	CHN	93555124	91552376	89720294	95.90%
**V8-102100026**	CHN	92495750	90052373	87781434	94.90%
**V8-102100027**	CHN	93262890	91013310	88909496	95.33%
**V8-102100028**	CHN	93259872	91071295	89073758	95.51%
**V8-102100029**	CHN	92186236	89514341	87045184	94.42%
**V8-102100031A**	CHN	92355052	91132143	90040540	97.49%
**V8-102100032**	CHN	92429730	90243880	88223838	95.45%
**V8-102100033**	CHN	92967038	90630793	88470886	95.16%
**V8-102100034**	CHN	92395192	90950185	89639958	97.02%
**V8-102100035A**	CHN	92211532	91151202	90206608	97.83%
**V8-102100036**	CHN	92563238	90157297	87914968	94.98%
**V8-102100037**	CHN	93508502	91468913	89579538	95.80%
**V8-102100038**	CHN	92811084	90440344	88223614	95.06%
**V8-102100039**	CHN	93428046	91637942	89983824	96.31%
**V8-102100040**	CHN	92461010	89923060	87551130	94.69%
**V8-102100041**	CHN	93379376	91088026	88993766	95.30%
**V8-102100042A**	CHN	93059546	91938123	90932096	97.71%
**V8-102100043**	CHN	93940660	91938646	90350522	96.18%
**V8-102100044**	CHN	93672404	91432450	89613656	95.67%
**V8-102100045A**	CHN	87217382	85876567	84690630	97.10%
**V8-102100046**	CHN	94414150	91794615	89699038	95.01%
**V8-102100047**	CHN	94099756	91980973	90295730	95.96%
**V8-102100048**	CHN	92258406	90230667	88626594	96.06%
**V8-102100049**	CHN	94586408	92119410	90174384	95.34%
**V8-102100050**	CHN	94421370	92187412	90409462	95.75%
**V8-102100051**	CHN	93625656	90906916	88734158	94.78%
**V8-102100052**	CHN	93589104	91263635	89415618	95.54%
**V8-102100053A**	CHN	55470212	53439352	51716108	93.23%
**V8-102100054**	CHN	94194780	91652054	89602816	95.13%
**V8-102100055**	CHN	93902928	91678260	89913358	95.75%
**V8-102100057**	CHN	94104910	91490172	89400038	95.00%
**V8-102100059**	CHN	93938288	91368304	89352844	95.12%
**V8-102100061**	CHN	93796844	90847842	88439616	94.29%
**V8-102100062**	CHN	93053552	88920469	85535838	91.92%
**V8-102100065**	CHN	93359354	91080400	89270692	95.62%
**V8-102100066**	CHN	92017578	89771337	87851806	95.47%
**V8-102100067**	CHN	93654280	91483639	89604140	95.68%
**V8-102100068**	CHN	90310590	87947612	85873228	95.09%
**V8-102100069**	CHN	85282746	82890214	80833166	94.78%
**V8-102100070A**	CHN	92067824	89503866	87311242	94.83%

**V8-102100072A**	CHN	45005718	42724626	40740174	90.52%
**V8-102100073**	CHN	93841610	91238814	88989306	94.83%
**V8-102100074**	CHN	92319550	90243673	88452724	95.81%
**V8-102100075**	CHN	93022664	91316352	89863902	96.60%
**V8-102100076A**	CHN	93622662	90338013	87493654	93.45%
**V8-102100079**	CHN	91593332	89741402	88172642	96.27%
**V8-102100082**	CHN	93962792	91732659	89852198	95.63%
**V8-102100083**	CHN	93485132	91771277	90298078	96.59%
**V8-102100084**	CHN	92419724	90971840	89723410	97.08%
**V8-102100085**	CHN	92288034	90785591	89485626	96.96%
**V8-102100086**	CHN	92538760	91055922	89769780	97.01%
**V8-102100087**	CHN	88238102	86582869	85157012	96.51%
**V8-102100088A**	CHN	86799988	84334005	82117424	94.61%
**V8-102100089**	CHN	92914718	91405034	90081220	96.95%
**V8-102100090**	CHN	92141208	90787235	89627170	97.27%
**V8-102100091**	CHN	92966430	91732774	90666128	97.53%
**V8-102100092**	CHN	92823230	91411778	90185232	97.16%
**V8-102100093**	CHN	92252340	89823554	87763146	95.13%
**V8-102100094**	CHN	92340918	89516654	87126050	94.35%
**V8-102100096A**	CHN	94421628	92059378	89942692	95.26%
**V8-102100097**	CHN	92512260	90244303	88339724	95.49%
**V8-102100098**	CHN	92289224	89827826	87771754	95.11%
**V8-102100099A**	CHN	52884666	50876312	49053330	92.76%
**V8-102100100**	CHN	92103940	89908958	88046820	95.60%

ESM Table 2: Comparisons of basline Clr-transformed relative abundances of species between acarbose (Acar) and vlidagaliptin (Vlid) groups

Species	Occurrence		Mean rank		Acar vs. Vlid (Wilcoxon rank sum test, clr-transformed)		
	Acar	Vlid	Acar	Vlid	P-value	BH adjusted P-value	Enrichment
Holdemania_filiformis	32.00%	54.17%	41.9000	57.4167	0.0069	0.8100	Acar = Vlid
Bacteroides_intestinalis	30.00%	41.67%	44.8800	54.3125	0.1007	0.9400	Acar = Vlid
Barnesiella_intestinihominis	48.00%	35.42%	54.1000	44.7083	0.1021	0.9400	Vlid = Acar
Adlercreutzia_equolifaciens	42.00%	54.17%	44.9800	54.2083	0.1082	0.9400	Acar = Vlid
Bacteroides_thetaiotaomicron	94.00%	93.75%	45.2000	53.9792	0.1265	0.9400	Acar = Vlid
Lachnospiraceae_bacterium_2_1_58FAA	54.00%	39.58%	53.7600	45.0625	0.1301	0.9400	Vlid = Acar
Bacteroides_cellulosilyticus	54.00%	60.42%	45.2600	53.9167	0.1319	0.9400	Acar = Vlid
Bacteroides_nordii	50.00%	64.58%	45.2800	53.8958	0.1337	0.9400	Acar = Vlid
Megamonas_rupellensis	40.00%	29.17%	53.5800	45.2500	0.1471	0.9400	Vlid = Acar
Bacteroides_stercoris	88.00%	91.67%	45.5200	53.6458	0.1573	0.9400	Acar = Vlid
Ruminococcus_gnavus	84.00%	70.83%	53.4600	45.3750	0.1594	0.9400	Vlid = Acar
Coprococcus_comes	70.00%	79.17%	45.6600	53.5000	0.1724	0.9400	Acar = Vlid
Bacteroides_vulgatus	96.00%	91.67%	53.0400	45.8125	0.2084	0.9400	Vlid = Acar
Paraprevotella_clara	28.00%	39.58%	45.9800	53.1667	0.2110	0.9400	Acar = Vlid
Veillonella_parvula	70.00%	62.50%	53.0200	45.8333	0.2110	0.9400	Vlid = Acar
Ruminococcus_obeum	98.00%	97.92%	46.0400	53.1042	0.2189	0.9400	Acar = Vlid
Alistipes_onderdonkii	70.00%	62.50%	52.8200	46.0417	0.2381	0.9400	Vlid = Acar
Bilophila_wadsworthia	68.00%	54.17%	52.8200	46.0417	0.2381	0.9400	Vlid = Acar
Eubacterium_ventriosum	64.00%	75.00%	46.3000	52.8333	0.2555	0.9400	Acar = Vlid
Haemophilus_parainfluenzae	62.00%	47.92%	52.6600	46.2083	0.2615	0.9400	Vlid = Acar
Dorea_formicigenerans	82.00%	79.17%	52.5600	46.3125	0.2769	0.9400	Vlid = Acar
Faecalibacterium_prausnitzii	94.00%	95.83%	46.4600	52.6667	0.2800	0.9400	Acar = Vlid
Bacteroides_fragilis	74.00%	64.58%	52.5200	46.3542	0.2832	0.9400	Vlid = Acar
Alistipes_shahii	68.00%	72.92%	46.4800	52.6458	0.2832	0.9400	Acar = Vlid
Bacteroides_finegoldii	44.00%	56.25%	46.5800	52.5417	0.2995	0.9400	Acar = Vlid
Escherichia_coli	98.00%	97.92%	52.4000	46.4792	0.3028	0.9400	Vlid = Acar
Bacteroides_plebeius	66.00%	75.00%	46.6400	52.4792	0.3095	0.9400	Acar = Vlid
Rothia_mucilaginosa	50.00%	37.50%	52.3000	46.5833	0.3198	0.9400	Vlid = Acar
Clostridium_asparagiforme	40.00%	52.08%	46.7000	52.4167	0.3198	0.9400	Acar = Vlid

Clostridium_bartlettii	74.00%	62.50%	52.3000	46.5833	0.3198	0.9400	Vlid = Acar
Bacteroides_coprocola	46.00%	37.50%	52.2800	46.6042	0.3232	0.9400	Vlid = Acar
Lactobacillus_mucosae	8.00%	8.33%	46.7800	52.3333	0.3338	0.9400	Acar = Vlid
Alistipes_putredinis	66.00%	66.67%	46.8200	52.2917	0.3409	0.9400	Acar = Vlid
Lactobacillus_salivarius	14.00%	14.58%	46.9000	52.2083	0.3556	0.9400	Acar = Vlid
Clostridium_symbiosum	44.00%	35.42%	52.0600	46.8333	0.3630	0.9400	Vlid = Acar
Clostridium_nexile	40.00%	25.00%	51.9600	46.9375	0.3821	0.9400	Vlid = Acar
Paraprevotella_xylaniphila	38.00%	47.92%	47.0800	52.0208	0.3898	0.9400	Acar = Vlid
Streptococcus_thermophilus	20.00%	8.33%	51.9200	46.9792	0.3898	0.9400	Vlid = Acar
Parabacteroides_goldsteinii	30.00%	33.33%	47.1200	51.9792	0.3977	0.9400	Acar = Vlid
Bacteroides_massiliensis	52.00%	52.08%	47.1600	51.9375	0.4057	0.9400	Acar = Vlid
Alistipes_finegoldii	56.00%	58.33%	47.1600	51.9375	0.4057	0.9400	Acar = Vlid
Ruminococcus_callidus	48.00%	56.25%	47.1800	51.9167	0.4097	0.9400	Acar = Vlid
Odoribacter_splanchnicus	56.00%	56.25%	47.2000	51.8958	0.4138	0.9400	Acar = Vlid
Ruminococcus_lactaris	34.00%	39.58%	47.2000	51.8958	0.4138	0.9400	Acar = Vlid
Bacteroides_dorei	70.00%	70.83%	47.2200	51.8750	0.4178	0.9400	Acar = Vlid
Lachnospiraceae_bacterium_1_1_57FAA	66.00%	64.58%	47.2400	51.8542	0.4219	0.9400	Acar = Vlid
Eubacterium_hallii	96.00%	85.42%	51.6800	47.2292	0.4386	0.9400	Vlid = Acar
Megamonas_funiformis	36.00%	27.08%	51.6800	47.2292	0.4386	0.9400	Vlid = Acar
Roseburia_intestinalis	78.00%	64.58%	51.6600	47.2500	0.4428	0.9400	Vlid = Acar
Lachnospiraceae_bacterium_1_4_56FAA	48.00%	58.33%	47.3600	51.7292	0.4470	0.9400	Acar = Vlid
Dorea_longicatena	90.00%	87.50%	47.3800	51.7083	0.4513	0.9400	Acar = Vlid
Ruminococcus_bromii	66.00%	56.25%	51.5400	47.3750	0.4685	0.9400	Vlid = Acar
Klebsiella_pneumoniae	68.00%	66.67%	51.5000	47.4167	0.4773	0.9400	Vlid = Acar
Lachnospiraceae_bacterium_5_1_63FAA	80.00%	89.58%	47.5400	51.5417	0.4861	0.9400	Acar = Vlid
Megasphaera_elsdenii	22.00%	22.92%	47.5600	51.5208	0.4906	0.9400	Acar = Vlid
Oxalobacter_formigenes	30.00%	25.00%	51.4000	47.5208	0.4996	0.9400	Vlid = Acar
Clostridium_leptum	34.00%	29.17%	51.3800	47.5417	0.5041	0.9400	Vlid = Acar
Collinsella_aerofaciens	58.00%	75.00%	47.6400	51.4375	0.5087	0.9400	Acar = Vlid
Bacteroides_ovatus	98.00%	100.00%	51.2800	47.6458	0.5271	0.9400	Vlid = Acar
Streptococcus_australis	44.00%	39.58%	51.2800	47.6458	0.5271	0.9400	Vlid = Acar
Pyramidobacter_piscolens	26.00%	16.67%	51.2600	47.6667	0.5317	0.9400	Vlid = Acar
Bacteroides_uniformis	94.00%	91.67%	47.8000	51.2708	0.5458	0.9400	Acar = Vlid
Clostridium_perfringens	12.00%	12.50%	47.8400	51.2292	0.5553	0.9400	Acar = Vlid
Roseburia_hominis	74.00%	66.67%	47.8800	51.1875	0.5649	0.9400	Acar = Vlid
Bacteroides_eggerthii	42.00%	33.33%	51.1000	47.8333	0.5697	0.9400	Vlid = Acar
Dialister_invisus	34.00%	27.08%	51.1000	47.8333	0.5697	0.9400	Vlid = Acar
Bifidobacterium_bifidum	20.00%	14.58%	51.0600	47.8750	0.5794	0.9400	Vlid = Acar

Bacteroides_salyersiae	26.00%	29.17%	47.9600	51.1042	0.5842	0.9400	Acar = Vlid
Streptococcus_anginosus	24.00%	20.83%	51.0000	47.9375	0.5940	0.9400	Vlid = Acar
Bifidobacterium_longum	82.00%	75.00%	50.9800	47.9583	0.5990	0.9400	Vlid = Acar
Bacteroides_caccae	78.00%	75.00%	50.9600	47.9792	0.6039	0.9400	Vlid = Acar
Streptococcus_salivarius	70.00%	64.58%	50.8600	48.0833	0.6289	0.9400	Vlid = Acar
Parabacteroides_distasonis	80.00%	85.42%	48.1600	50.8958	0.6340	0.9400	Acar = Vlid
Megamonas_hypermegale	40.00%	31.25%	50.8200	48.1250	0.6390	0.9400	Vlid = Acar
Enterobacter_cloacae	44.00%	43.75%	50.8000	48.1458	0.6441	0.9400	Vlid = Acar
Enterococcus_faecium	22.00%	16.67%	48.2400	50.8125	0.6544	0.9400	Acar = Vlid
Streptococcus_infantis	20.00%	10.42%	50.7600	48.1875	0.6544	0.9400	Vlid = Acar
Clostridium_ramosum	20.00%	20.83%	48.2600	50.7917	0.6595	0.9400	Acar = Vlid
Lachnospiraceae_bacterium_8_1_57FAA	22.00%	16.67%	48.3000	50.7500	0.6698	0.9400	Acar = Vlid
Eubacterium_rectale	82.00%	89.58%	48.3800	50.6667	0.6906	0.9400	Acar = Vlid
Anaerostipes_hadrus	70.00%	79.17%	48.4000	50.6458	0.6959	0.9400	Acar = Vlid
Bifidobacterium_pseudocatenulatum	54.00%	52.08%	50.5600	48.3958	0.7064	0.9400	Vlid = Acar
Lachnospiraceae_bacterium_3_1_46FAA	58.00%	64.58%	48.4400	50.6042	0.7064	0.9400	Acar = Vlid
Prevotella_copri	90.00%	89.58%	50.5400	48.4167	0.7117	0.9400	Vlid = Acar
Bifidobacterium_adolescentis	36.00%	33.33%	48.5000	50.5417	0.7223	0.9400	Acar = Vlid
Streptococcus_sanguinis	18.00%	16.67%	50.5000	48.4583	0.7223	0.9400	Vlid = Acar
candidate_division_TM7_single_cell_isola	28.00%	25.00%	48.5200	50.5208	0.7277	0.9400	Acar = Vlid
Clostridium_citroniae	60.00%	62.50%	48.5200	50.5208	0.7277	0.9400	Acar = Vlid
Parabacteroides_merdae	70.00%	75.00%	48.5400	50.5000	0.7330	0.9400	Acar = Vlid
Ruminococcus_sp_5_1_39BFAA	48.00%	54.17%	48.6000	50.4375	0.7491	0.9400	Acar = Vlid
Burkholderiales_bacterium_1_1_47	80.00%	75.00%	48.6200	50.4167	0.7545	0.9400	Acar = Vlid
Alistipes_indistinctus	50.00%	43.75%	50.3600	48.6042	0.7599	0.9400	Vlid = Acar
Streptococcus_parasanguinis	48.00%	47.92%	48.6600	50.3750	0.7653	0.9400	Acar = Vlid
Parasutterella_excrementihominis	80.00%	75.00%	48.6600	50.3750	0.7653	0.9400	Acar = Vlid
Bacteroides_faecis	44.00%	41.67%	50.3000	48.6667	0.7762	0.9400	Vlid = Acar
Acidaminococcus_intestini	24.00%	14.58%	50.1800	48.7917	0.8091	0.9400	Vlid = Acar
Streptococcus_mitis_oralis_pneumoniae	30.00%	25.00%	50.1200	48.8542	0.8256	0.9400	Vlid = Acar
Veillonella_atypica	40.00%	31.25%	50.1200	48.8542	0.8256	0.9400	Vlid = Acar
Flavonifractor_plautii	70.00%	68.75%	50.1000	48.8750	0.8312	0.9400	Vlid = Acar
Eubacterium_ramulus	74.00%	68.75%	48.9000	50.1250	0.8312	0.9400	Acar = Vlid
Coprococcus_catus	78.00%	75.00%	48.9000	50.1250	0.8312	0.9400	Acar = Vlid
Eubacterium_eligens	68.00%	70.83%	48.9200	50.1042	0.8367	0.9400	Acar = Vlid
Citrobacter_freundii	24.00%	27.08%	48.9400	50.0833	0.8423	0.9400	Acar = Vlid
Clostridium_hathewayi	50.00%	54.17%	50.0400	48.9375	0.8478	0.9400	Vlid = Acar
Eubacterium_biforme	26.00%	18.75%	50.0400	48.9375	0.8478	0.9400	Vlid = Acar

Bacteroides_xylanisolvens	88.00%	93.75%	48.9800	50.0417	0.8534	0.9400	Acar = Vlid
Streptococcus_vestibularis	14.00%	10.42%	49.0400	49.9792	0.8702	0.9400	Acar = Vlid
Clostridium_bolteae	82.00%	85.42%	49.0600	49.9583	0.8758	0.9400	Acar = Vlid
Roseburia_inulinivorans	84.00%	77.08%	49.0600	49.9583	0.8758	0.9400	Acar = Vlid
Ruminococcus_torques	98.00%	100.00%	49.8400	49.1458	0.9038	0.9501	Vlid = Acar
Solobacterium_moorei	10.00%	10.42%	49.1600	49.8542	0.9038	0.9501	Acar = Vlid
Bacteroidales_bacterium_ph8	52.00%	54.17%	49.8200	49.1667	0.9095	0.9501	Vlid = Acar
Veillonella_dispar	30.00%	25.00%	49.3200	49.6875	0.9490	0.9686	Acar = Vlid
Akkermansia_muciniphila	38.00%	35.42%	49.3400	49.6667	0.9547	0.9686	Acar = Vlid
Alistipes_senegalensis	52.00%	52.08%	49.3600	49.6458	0.9603	0.9686	Acar = Vlid
Clostridiales_bacterium_1_7_47FAA	38.00%	37.50%	49.6400	49.3542	0.9603	0.9686	Vlid = Acar
Lachnospiraceae_bacterium_7_1_58FAA	62.00%	58.33%	49.5600	49.4375	0.9830	0.9830	Vlid = Acar

ESM Table 3: Comparison of genera/species/pathways relative abundances (centered log-ratio, clr-transformed) between baseline and 6-month in the acarbose group

ESM Table 3a: Comparison of genera relative abundances between baseline and 6-month in the acarbose group

Genus	Relative abundance (mean)		Relative abundance (median)		Baseline vs. M6 (Wilcoxon signed-rank test, clr-transformed)			
	Baseline	6-month	Baseline	6-month	Effect_size	P-value	BH adjusted P-value	Enrichment
Bifidobacterium	0.4139	17.6499	0.1607	14.2470	0.8640	0.0000	0.0000	M6
Lactobacillus	0.0616	8.1207	0.0000	3.2839	0.8190	0.0000	0.0000	M6
Bacteroides	33.6524	8.9478	27.3139	0.8726	-0.7440	0.0000	0.0000	base
Roseburia	1.8247	0.5809	1.0053	0.0215	-0.7210	0.0000	0.0000	base
Solobacterium	0.0002	0.0025	0.0000	0.0000	0.7110	0.0000	0.0000	M6
Streptococcus	0.1115	1.9067	0.0159	0.2689	0.7040	0.0000	0.0000	M6
Alistipes	5.1538	0.9399	1.8744	0.0000	-0.7040	0.0000	0.0000	base
Bilophila	0.2884	0.0693	0.1583	0.0000	-0.6730	0.0000	0.0001	base
Actinomyces	0.0003	0.0081	0.0000	0.0000	0.6670	0.0000	0.0001	M6
Oscillibacter	0.2674	0.1628	0.0687	0.0000	-0.6590	0.0000	0.0001	base
Parabacteroides	2.6484	0.9854	1.7935	0.0866	-0.6500	0.0000	0.0001	base
Acidaminococcus	0.0064	0.3877	0.0000	0.0014	0.6450	0.0000	0.0001	M6
Clostridium	0.7935	0.1312	0.1596	0.0083	-0.6450	0.0000	0.0001	base
Megasphaera	0.3349	8.2853	0.0000	0.2028	0.6260	0.0000	0.0002	M6
Veillonella	0.1819	1.2290	0.0157	0.0445	0.6190	0.0001	0.0002	M6
Haemophilus	0.1266	1.0939	0.0010	0.0388	0.6160	0.0001	0.0002	M6
Granulicatella	0.0014	0.0067	0.0000	0.0010	0.6110	0.0001	0.0002	M6
Odoribacter	0.4103	0.0349	0.0946	0.0000	-0.6080	0.0001	0.0002	base
Holdemania	0.0168	0.0021	0.0011	0.0000	-0.6030	0.0001	0.0002	base
Collinsella	0.0817	0.6057	0.0317	0.1290	0.5000	0.0012	0.0029	M6
Adlercreutzia	0.0123	0.0020	0.0000	0.0000	-0.4920	0.0014	0.0033	base
Barnesiella	1.1679	0.0678	0.0000	0.0000	-0.4740	0.0021	0.0047	base
Flavonifractor	0.0257	0.0087	0.0070	0.0000	-0.4740	0.0021	0.0047	base
Gemella	0.0006	0.0032	0.0000	0.0000	0.4720	0.0022	0.0047	M6
Anaerostipes	0.0088	0.0302	0.0028	0.0075	0.4260	0.0058	0.0117	M6
Rothia	0.0095	0.0187	0.0000	0.0044	0.4000	0.0095	0.0182	M6
Subdoligranulum	2.0785	1.3349	0.5987	0.0893	-0.3970	0.0102	0.0189	base
Ruminococcus	0.9872	0.8461	0.6772	0.0050	-0.3950	0.0106	0.0189	base
Oxalobacter	0.0072	0.0000	0.0000	0.0000	-0.3890	0.0118	0.0203	base

Enterococcus	0.0086	0.0372	0.0000	0.0000	0.3530	0.0218	0.0363	M6
Parasutterella	0.1328	0.1540	0.0491	0.0163	-0.3360	0.0291	0.0470	base
Anaerotruncus	0.0088	0.0096	0.0000	0.0000	-0.3070	0.0461	0.0721	base
Akkermansia	0.0783	0.0526	0.0000	0.0000	-0.3040	0.0489	0.0741	base
Paraprevotella	0.5462	0.0471	0.0000	0.0000	-0.2810	0.0689	0.1013	none
Coprococcus	0.4190	0.3911	0.0710	0.0663	-0.2130	0.1671	0.2374	none
Blautia	1.4015	0.5777	0.3350	0.2702	-0.2110	0.1710	0.2374	none
Klebsiella	1.3550	1.2308	0.0406	0.0041	-0.2050	0.1830	0.2473	none
Prevotella	22.3263	15.5432	0.0455	0.1111	-0.1810	0.2424	0.3189	none
Dorea	0.1678	0.2756	0.1172	0.1604	0.1710	0.2685	0.3442	none
Eubacterium	4.5315	4.0505	2.4191	1.3592	-0.1590	0.3023	0.3779	none
Faecalibacterium	4.1276	5.0453	1.9777	3.1011	0.1460	0.3452	0.4209	none
Eggerthella	0.0060	0.0043	0.0007	0.0000	-0.1280	0.4057	0.4717	none
Megamonas	3.4273	6.2225	0.0014	0.0046	0.1280	0.4057	0.4717	none
Enterobacter	0.0560	0.0674	0.0000	0.0000	-0.1090	0.4799	0.5453	none
Citrobacter	0.0653	0.0130	0.0000	0.0000	0.0955	0.5360	0.5955	none
Pyramidobacter	0.0043	0.0349	0.0000	0.0000	0.0917	0.5526	0.6006	none
Coprobacillus	0.0240	0.0041	0.0000	0.0000	0.0704	0.6481	0.6895	none
Dialister	0.9561	1.3983	0.0000	0.0000	0.0608	0.6937	0.7226	none
Fusobacterium	0.4543	0.0188	0.0000	0.0000	-0.0492	0.7498	0.7651	none
Escherichia	4.7026	6.8819	0.5685	0.5569	0.0010	0.9950	0.9950	none

ESM Table 3b: Comparison of species relative abundances between baseline and 6-month in the acarbose group

**Species**	**Relative abundance (mean)**		**Relative abundance (median)**		**Baseline vs. M6 (Wilcoxon signed-rank test, clr-transformed)**			
	**Baseline**	**6-month**	**Baseline**	**6-month**	**Effect_size**	**P-value**	**BH adjusted P-value**	**Enrichment**
Bifidobacterium longum	0.1755	7.5879	0.0230	2.8067	0.8590	0.0000	0.0000	M6
Lactobacillus mucosae	0.0098	2.9111	0.0000	1.1072	0.8360	0.0000	0.0000	M6
Bacteroides xylanisolvens	0.2370	0.0421	0.0739	0.0000	-0.7920	0.0000	0.0000	base
Bifidobacterium adolescentis	0.0790	5.8299	0.0000	0.0030	0.7840	0.0000	0.0000	M6
Clostridium bolteae	0.0816	0.0028	0.0218	0.0000	-0.7610	0.0000	0.0000	base
Bifidobacterium bifidum	0.0265	0.6274	0.0000	0.0272	0.7590	0.0000	0.0000	M6
Bifidobacterium pseudocatenulatum	0.1317	1.5678	0.0010	0.3706	0.7450	0.0000	0.0000	M6
Solobacterium moorei	0.0002	0.0025	0.0000	0.0000	0.7420	0.0000	0.0000	M6

Streptococcus salivarius	0.0480	1.3773	0.0051	0.1419	0.7380	0.0000	0.0000	M6
Roseburia intestinalis	0.5636	0.0939	0.0826	0.0000	-0.7070	0.0000	0.0001	base
Bacteroides uniformis	2.3748	0.4804	0.4400	0.0042	-0.6990	0.0000	0.0001	base
Bacteroides thetaiotaomicron	0.9393	0.1658	0.3316	0.0025	-0.6970	0.0000	0.0001	base
Bilophila wadsworthia	0.0225	0.0023	0.0091	0.0000	-0.6970	0.0000	0.0001	base
Alistipes shahii	0.5661	0.0424	0.2246	0.0000	-0.6960	0.0000	0.0001	base
Parabacteroides merdae	1.5629	0.1766	0.5841	0.0000	-0.6910	0.0000	0.0001	base
Roseburia hominis	0.1747	0.0350	0.0406	0.0000	-0.6800	0.0000	0.0001	base
Streptococcus vestibularis	0.0007	0.0862	0.0000	0.0025	0.6760	0.0000	0.0001	M6
Bacteroides ovatus	2.9723	1.6486	0.8433	0.0051	-0.6670	0.0000	0.0001	base
Lachnospiraceae bacterium 7 1 58FAA	0.0074	0.0011	0.0015	0.0000	-0.6650	0.0000	0.0001	base
Eubacterium hallii	0.1673	0.0825	0.1076	0.0178	-0.6470	0.0000	0.0002	base
Bacteroides caccae	2.4908	0.3868	0.6884	0.0006	-0.6390	0.0000	0.0002	base
Collinsella aerofaciens	0.0723	0.6024	0.0187	0.1290	0.6280	0.0000	0.0002	M6
Lactobacillus salivarius	0.0127	0.0618	0.0000	0.0000	0.6280	0.0000	0.0002	M6
Haemophilus parainfluenzae	0.1265	1.0824	0.0010	0.0388	0.6260	0.0000	0.0002	M6
Parabacteroides distasonis	0.6422	0.3957	0.0790	0.0027	-0.6230	0.0001	0.0002	base
Alistipes onderdonkii	1.6432	0.1371	0.1407	0.0000	-0.6190	0.0001	0.0003	base
Clostridium citroniae	0.0147	0.0016	0.0050	0.0000	-0.6140	0.0001	0.0003	base
Streptococcus sanguinis	0.0007	0.0158	0.0000	0.0000	0.6080	0.0001	0.0003	M6
Odoribacter splanchnicus	0.4103	0.0347	0.0946	0.0000	-0.6060	0.0001	0.0003	base
Veillonella parvula	0.0930	0.5247	0.0076	0.0214	0.6050	0.0001	0.0003	M6
Acidaminococcus intestini	0.0029	0.0260	0.0000	0.0000	0.5970	0.0001	0.0004	M6
Clostridium perfringens	0.0034	0.0812	0.0000	0.0000	0.5880	0.0001	0.0005	M6
Megasphaera elsdenii	0.0208	0.3283	0.0000	0.0000	0.5850	0.0001	0.0005	M6
Alistipes putredinis	2.6731	0.7362	0.0742	0.0000	-0.5850	0.0001	0.0005	base
Alistipes senegalensis	0.0188	0.0047	0.0002	0.0000	-0.5830	0.0002	0.0005	base
Bacteroides stercoris	5.4739	0.5898	0.4329	0.0038	-0.5820	0.0002	0.0005	base
Bacteroidales bacterium ph8	0.5795	0.0518	0.0086	0.0000	-0.5800	0.0002	0.0005	base
Bacteroides nordii	0.2620	0.0009	0.0016	0.0000	-0.5760	0.0002	0.0006	base
Roseburia inulinivorans	1.0719	0.4346	0.3098	0.0000	-0.5720	0.0002	0.0006	base
Alistipes indistinctus	0.0808	0.0039	0.0045	0.0000	-0.5660	0.0002	0.0007	base
Bacteroides cellulosilyticus	0.0994	0.0193	0.0118	0.0000	-0.5450	0.0004	0.0012	base
Streptococcus anginosus	0.0040	0.0189	0.0000	0.0000	0.5200	0.0008	0.0020	M6
Veillonella atypica	0.0172	0.0552	0.0000	0.0029	0.5200	0.0008	0.0020	M6
Streptococcus mitis oralis pneumoniae	0.0023	0.0053	0.0000	0.0012	0.5180	0.0008	0.0021	M6
Bacteroides vulgatus	4.9402	2.8372	1.5884	0.1507	-0.5120	0.0009	0.0023	base
Alistipes finegoldii	0.1039	0.0097	0.0149	0.0000	-0.5030	0.0011	0.0029	base

Veillonella dispar	0.0024	0.0210	0.0000	0.0000	0.4950	0.0013	0.0033	M6
Lachnospiraceae bacterium 3 1 46FAA	0.1197	0.0089	0.0029	0.0000	-0.4890	0.0015	0.0037	base
Bacteroides dorei	1.6145	0.2490	0.0243	0.0007	-0.4740	0.0021	0.0051	base
Lachnospiraceae bacterium 1 1 57FAA	0.1526	0.0267	0.0494	0.0000	-0.4690	0.0023	0.0055	base
Streptococcus parasanguinis	0.0235	0.1050	0.0000	0.0035	0.4660	0.0025	0.0056	M6
Eubacterium ventriosum	0.0840	0.0300	0.0251	0.0000	-0.4660	0.0025	0.0056	base
Lachnospiraceae bacterium 1 4 56FAA	0.0175	0.0006	0.0009	0.0000	-0.4660	0.0025	0.0056	base
Flavonifractor plautii	0.0257	0.0087	0.0070	0.0000	-0.4490	0.0036	0.0079	base
candidate division TM7 single cell isolat	0.0011	0.0019	0.0000	0.0000	0.4290	0.0054	0.0115	M6
Anaerostipes hadrus	0.0084	0.0300	0.0027	0.0075	0.4260	0.0058	0.0122	M6
Bacteroides plebeius	3.8845	0.8390	0.0136	0.0001	-0.4210	0.0063	0.0129	base
Rothia mucilaginosa	0.0091	0.0176	0.0000	0.0039	0.4200	0.0065	0.0132	M6
Adlercreutzia equolifaciens	0.0123	0.0020	0.0000	0.0000	-0.4180	0.0068	0.0135	base
Barnesiella intestinihominis	1.1679	0.0678	0.0000	0.0000	-0.4120	0.0076	0.0148	base
Clostridium asparagiforme	0.0169	0.0030	0.0000	0.0000	-0.4100	0.0079	0.0151	base
Bacteroides massiliensis	1.6754	0.0990	0.0020	0.0000	-0.4060	0.0085	0.0158	base
Clostridium hathewayi	0.0787	0.0244	0.0012	0.0000	-0.4060	0.0085	0.0158	base
Lachnospiraceae bacterium 5 1 63FAA	0.0356	0.1710	0.0084	0.0231	0.4030	0.0091	0.0164	M6
Lachnospiraceae bacterium 8 1 57FAA	0.0126	0.0609	0.0000	0.0000	0.4030	0.0091	0.0164	M6
Eubacterium biforme	0.0308	0.0484	0.0000	0.0000	0.4000	0.0095	0.0168	M6
Ruminococcus callidus	0.0729	0.0124	0.0000	0.0000	-0.3970	0.0102	0.0178	base
Clostridium symbiosum	0.0181	0.0007	0.0000	0.0000	-0.3950	0.0106	0.0179	base
Enterococcus faecium	0.0026	0.0268	0.0000	0.0000	0.3950	0.0106	0.0179	M6
Streptococcus infantis	0.0006	0.0078	0.0000	0.0000	0.3920	0.0109	0.0183	M6
Bacteroides eggerthii	1.1056	0.0871	0.0000	0.0000	-0.3870	0.0122	0.0201	base
Bacteroides faecis	0.0958	0.0128	0.0000	0.0000	-0.3810	0.0135	0.0220	base
Streptococcus thermophilus	0.0197	0.0312	0.0000	0.0000	0.3610	0.0191	0.0305	M6
Eubacterium ramulus	0.0170	0.0105	0.0046	0.0000	-0.3430	0.0265	0.0418	base
Parasutterella excrementihominis	0.1328	0.1540	0.0491	0.0163	-0.3300	0.0320	0.0493	base
Ruminococcus bromii	0.6250	0.6562	0.1029	0.0000	-0.3300	0.0320	0.0493	base
Ruminococcus obeum	0.0866	0.0494	0.0584	0.0350	-0.3290	0.0330	0.0502	base
Bacteroides finegoldii	0.5364	0.0890	0.0000	0.0000	-0.3260	0.0351	0.0527	base
Coprococcus catus	0.0422	0.0842	0.0185	0.0016	-0.3130	0.0422	0.0625	base
Streptococcus australis	0.0038	0.0069	0.0000	0.0013	0.3060	0.0475	0.0695	M6
Burkholderiales bacterium 1 1 47	0.2275	0.2200	0.0899	0.0274	-0.2900	0.0599	0.0865	base
Ruminococcus sp 5 1 39BFAA	0.1924	0.0689	0.0000	0.0000	-0.2870	0.0633	0.0904	base
Oxalobacter formigenes	0.0072	0.0000	0.0000	0.0000	-0.2840	0.0651	0.0918	base
Bacteroides coprocola	2.6708	0.7505	0.0000	0.0000	-0.2680	0.0811	0.1117	none

Paraprevotella xylaniphila	0.0532	0.0020	0.0000	0.0000	-0.2680	0.0811	0.1117	none
Clostridium leptum	0.0090	0.0004	0.0000	0.0000	-0.2420	0.1166	0.1586	none
Holdemania filiformis	0.0064	0.0021	0.0000	0.0000	-0.2190	0.1559	0.2072	none
Prevotella copri	21.6684	15.0532	0.0353	0.0191	-0.2190	0.1559	0.2072	none
Akkermansia muciniphila	0.0783	0.0526	0.0000	0.0000	-0.2180	0.1595	0.2074	none
Clostridium nexile	0.1589	0.0112	0.0000	0.0000	-0.2180	0.1595	0.2074	none
Megamonas rupellensis	0.3452	0.6258	0.0000	0.0000	0.2050	0.1830	0.2353	none
Megamonas hypermegale	0.4369	0.3720	0.0000	0.0000	0.2020	0.1913	0.2433	none
Citrobacter freundii	0.0326	0.0082	0.0000	0.0000	0.1960	0.2044	0.2571	none
Ruminococcus torques	0.7399	0.3618	0.1732	0.0858	-0.1870	0.2276	0.2833	none
Clostridium bartlettii	0.0428	0.0342	0.0058	0.0026	-0.1740	0.2578	0.3175	none
Pyramidobacter piscolens	0.0043	0.0349	0.0000	0.0000	0.1730	0.2631	0.3207	none
Clostridium ramosum	0.0019	0.0014	0.0000	0.0000	0.1590	0.3023	0.3646	none
Ruminococcus lactaris	0.0949	0.1087	0.0000	0.0000	0.1570	0.3082	0.3679	none
Faecalibacterium prausnitzii	4.1276	5.0453	1.9777	3.1011	0.1560	0.3142	0.3713	none
Bacteroides salyersiae	0.0936	0.0038	0.0000	0.0000	-0.1480	0.3388	0.3964	none
Klebsiella pneumoniae	1.3526	1.2270	0.0397	0.0025	-0.1460	0.3452	0.3998	none
Eubacterium eligens	0.9462	0.4657	0.0613	0.0017	-0.1420	0.3581	0.4107	none
Ruminococcus gnavus	0.5740	0.1665	0.0201	0.0342	-0.1380	0.3713	0.4218	none
Eubacterium rectale	3.2503	3.4605	0.8786	0.1341	0.1300	0.3987	0.4485	none
Lachnospiraceae bacterium 2 1 58FAA	0.1429	0.0503	0.0002	0.0001	0.1280	0.4057	0.4521	none
Enterobacter cloacae	0.0503	0.0671	0.0000	0.0000	-0.1260	0.4128	0.4556	none
Dialister invisus	0.9328	1.3727	0.0000	0.0000	0.1190	0.4419	0.4832	none
Clostridiales bacterium 1 7 47FAA	0.0026	0.0077	0.0000	0.0000	-0.1170	0.4494	0.4868	none
Bacteroides intestinalis	0.0462	0.0043	0.0000	0.0000	-0.1070	0.4877	0.5235	none
Parabacteroides goldsteinii	0.0549	0.0152	0.0000	0.0000	-0.0974	0.5278	0.5613	none
Dorea formicigenerans	0.0616	0.0906	0.0420	0.0386	0.0878	0.5694	0.6002	none
Bacteroides fragilis	1.1627	0.4993	0.0426	0.0161	-0.0858	0.5779	0.6037	none
Megamonas funiformis	0.2822	0.6897	0.0000	0.0000	0.0839	0.5865	0.6073	none
Dorea longicatena	0.1017	0.1612	0.0657	0.0571	-0.0801	0.6038	0.6197	none
Paraprevotella clara	0.1569	0.0100	0.0000	0.0000	-0.0781	0.6126	0.6232	none
Escherichia coli	4.3135	6.5011	0.4981	0.5036	0.0145	0.9253	0.9333	none
Coprococcus comes	0.0693	0.1680	0.0307	0.0105	0.0068	0.9651	0.9651	none

453

ESM Table 3c: Comparison of pathway relative abundances between baseline and 6-month in the acarbose group

Pathway	Relative abundance (mean)		Relative abundance (median)		Baseline vs. M6 (Wilcoxon signed-rank test, clr-transformed)			
	Baseline	6-month	Baseline	6-month	Effect_size	P-value	BH adjusted P-value	Enrichment
UDPNAGSYN-PWY: UDP-N-acetyl-D-glucosamine biosynthesis I	0.0015	0.0066	0.0012	0.0043	0.8180	0.0000	0.0000	M6
P4-PWY: superpathway of L-lysine, L-threonine and L-methionine biosynthesis I	0.0015	0.0028	0.0014	0.0028	0.7330	0.0000	0.0001	M6
HOMOSER-METSYN-PWY: L-methionine biosynthesis I	0.0012	0.0027	0.0010	0.0023	0.7300	0.0000	0.0001	M6
ARGININE-SYN4-PWY: L-ornithine de novo biosynthesis	0.0055	0.0012	0.0056	0.0002	-0.7250	0.0000	0.0001	base
PYRIDOXSYN-PWY: pyridoxal 5'-phosphate biosynthesis I	0.0057	0.0016	0.0047	0.0008	-0.7250	0.0000	0.0001	base
PWY-6531: mannitol cycle	0.0005	0.0002	0.0004	0.0000	-0.7220	0.0000	0.0001	base
MET-SAM-PWY: superpathway of S-adenosyl-L-methionine biosynthesis	0.0018	0.0040	0.0016	0.0038	0.7220	0.0000	0.0001	M6
PWY0-845: superpathway of pyridoxal 5'-phosphate biosynthesis and salvage	0.0055	0.0015	0.0053	0.0006	-0.7180	0.0000	0.0001	base
PWY-6936: seleno-amino acid biosynthesis	0.0012	0.0034	0.0007	0.0033	0.7180	0.0000	0.0001	M6
PWY0-1261: anhydromuropeptides recycling	0.0018	0.0028	0.0013	0.0020	0.7130	0.0000	0.0001	M6
PWY-7234: inosine-5'-phosphate biosynthesis III	0.0022	0.0059	0.0020	0.0050	0.7100	0.0000	0.0001	M6
PWY-7209: superpathway of pyrimidine ribonucleosides degradation	0.0000	0.0004	0.0000	0.0002	0.7070	0.0000	0.0001	M6
PWY-5101: L-isoleucine biosynthesis II	0.0020	0.0005	0.0016	0.0000	-0.6970	0.0000	0.0002	base
OANTIGEN-PWY: O-antigen building blocks biosynthesis (E. coli)	0.0014	0.0043	0.0013	0.0036	0.6840	0.0000	0.0002	M6
PWY-2941: L-lysine biosynthesis II	0.0009	0.0037	0.0007	0.0022	0.6840	0.0000	0.0002	M6
PWY-7323: superpathway of GDP-mannose-derived O-antigen building blocks biosynthesis	0.0020	0.0009	0.0015	0.0004	-0.6820	0.0000	0.0002	base
METSYN-PWY: L-homoserine and L-methionine biosynthesis	0.0019	0.0038	0.0017	0.0036	0.6820	0.0000	0.0002	M6
ARGSYNBSUB-PWY: L-arginine biosynthesis II (acetyl cycle)	0.0023	0.0056	0.0022	0.0045	0.6760	0.0000	0.0002	M6
PWY-5347: superpathway of L-methionine biosynthesis (transsulfuration)	0.0021	0.0039	0.0019	0.0038	0.6680	0.0000	0.0002	M6
PWY-6731: starch degradation III	0.0000	0.0001	0.0000	0.0000	0.6680	0.0000	0.0002	M6
PWY-7560: methylerythritol phosphate pathway II	0.0014	0.0036	0.0010	0.0029	0.6600	0.0000	0.0003	M6
GLUTORN-PWY: L-ornithine biosynthesis	0.0018	0.0043	0.0015	0.0035	0.6570	0.0000	0.0003	M6
PWY-6270: isoprene biosynthesis I	0.0015	0.0037	0.0011	0.0032	0.6570	0.0000	0.0003	M6
DAPLYSINESYN-PWY: L-lysine biosynthesis I	0.0022	0.0040	0.0020	0.0042	0.6510	0.0000	0.0003	M6
ARGSYN-PWY: L-arginine biosynthesis I (via L-ornithine)	0.0036	0.0076	0.0036	0.0065	0.6500	0.0000	0.0003	M6
LACTOSECAT-PWY: lactose and galactose degradation I	0.0002	0.0011	0.0001	0.0005	0.6500	0.0000	0.0003	M6
PWY-7400: L-arginine biosynthesis IV (archaebacteria)	0.0038	0.0079	0.0038	0.0067	0.6390	0.0000	0.0004	M6
PWY-922: mevalonate pathway I	0.0000	0.0001	0.0000	0.0000	0.6390	0.0000	0.0004	M6
CENTFERM-PWY: pyruvate fermentation to butanoate	0.0001	0.0000	0.0001	0.0000	-0.6300	0.0000	0.0005	base
PWY-5910: superpathway of geranylgeranyldiphosphate biosynthesis I (via mevalonate)	0.0000	0.0001	0.0000	0.0000	0.6300	0.0000	0.0005	M6
PWY-6590: superpathway of Clostridium acetobutylicum acidogenic fermentation	0.0002	0.0000	0.0001	0.0000	-0.6280	0.0000	0.0005	base
PWY-5100: pyruvate fermentation to acetate and lactate II	0.0019	0.0048	0.0016	0.0032	0.6230	0.0001	0.0005	M6
HISTSYN-PWY: L-histidine biosynthesis	0.0025	0.0060	0.0023	0.0054	0.6190	0.0001	0.0006	M6

PWY-5121: superpathway of geranylgeranyl diphosphate biosynthesis II (via MEP)	0.0008	0.0015	0.0005	0.0011	0.6080	0.0001	0.0008	M6
PWY-621: sucrose degradation III (sucrose invertase)	0.0034	0.0069	0.0030	0.0057	0.6030	0.0001	0.0009	M6
PWY-4981: L-proline biosynthesis II (from arginine)	0.0003	0.0017	0.0003	0.0007	0.5990	0.0001	0.0010	M6
P42-PWY: incomplete reductive TCA cycle	0.0011	0.0006	0.0010	0.0003	-0.5970	0.0001	0.0010	base
PWY0-1297: superpathway of purine deoxyribonucleosides degradation	0.0015	0.0024	0.0009	0.0020	0.5960	0.0001	0.0010	M6
PWY-7282: 4-amino-2-methyl-5-phosphomethylpyrimidine biosynthesis (yeast)	0.0056	0.0025	0.0055	0.0017	-0.5930	0.0001	0.0010	base
PWY-4984: urea cycle	0.0011	0.0006	0.0007	0.0001	-0.5910	0.0001	0.0011	base
CITRULBIO-PWY: L-citrulline biosynthesis	0.0012	0.0007	0.0009	0.0001	-0.5890	0.0001	0.0011	base
PWY-5913: TCA cycle VI (obligate autotrophs)	0.0006	0.0022	0.0003	0.0011	0.5850	0.0001	0.0012	M6
COLANSYN-PWY: colanic acid building blocks biosynthesis	0.0016	0.0010	0.0016	0.0008	-0.5830	0.0002	0.0012	base
BRANCHED-CHAIN-AA-SYN-PWY: superpathway of branched amino acid biosynthesis	0.0061	0.0100	0.0054	0.0092	0.5590	0.0003	0.0021	M6
PWY0-781: aspartate superpathway	0.0014	0.0023	0.0013	0.0024	0.5590	0.0003	0.0021	M6
PWY-5103: L-isoleucine biosynthesis III	0.0052	0.0093	0.0045	0.0085	0.5590	0.0003	0.0021	M6
PWY66-409: superpathway of purine nucleotide salvage	0.0022	0.0034	0.0015	0.0035	0.5590	0.0003	0.0021	M6
FERMENTATION-PWY: mixed acid fermentation	0.0006	0.0013	0.0005	0.0009	0.5540	0.0003	0.0022	M6
PWY-3001: superpathway of L-isoleucine biosynthesis I	0.0055	0.0086	0.0052	0.0079	0.5510	0.0004	0.0024	M6
PWY-5030: L-histidine degradation III	0.0006	0.0002	0.0004	0.0000	-0.5430	0.0004	0.0029	base
PWY-6168: flavin biosynthesis III (fungi)	0.0065	0.0037	0.0061	0.0031	-0.5420	0.0005	0.0029	base
HISDEG-PWY: L-histidine degradation I	0.0004	0.0002	0.0003	0.0001	-0.5390	0.0005	0.0030	base
PWY-7383: anaerobic energy metabolism (invertebrates, cytosol)	0.0003	0.0001	0.0003	0.0000	-0.5390	0.0005	0.0030	base
P108-PWY: pyruvate fermentation to propanoate I	0.0002	0.0001	0.0001	0.0000	-0.5350	0.0005	0.0031	base
POLYAMINSYN3-PWY: superpathway of polyamine biosynthesis II	0.0002	0.0001	0.0001	0.0000	-0.5350	0.0005	0.0031	base
PWY-5022: 4-aminobutanoate degradation V	0.0007	0.0004	0.0005	0.0001	-0.5340	0.0005	0.0032	base
ARGORNPROST-PWY: arginine, ornithine and proline interconversion	0.0001	0.0000	0.0000	0.0000	-0.5140	0.0009	0.0049	base
PWY-6703: preQ0 biosynthesis	0.0113	0.0059	0.0117	0.0050	-0.5140	0.0009	0.0049	base
PWY-5173: superpathway of acetyl-CoA biosynthesis	0.0003	0.0005	0.0002	0.0002	0.5120	0.0009	0.0051	M6
NAD-BIOSYNTHESIS-II: NAD salvage pathway II	0.0008	0.0014	0.0004	0.0007	0.5080	0.0010	0.0054	M6
POLYISOPRENSYN-PWY: polyisoprenoid biosynthesis (E. coli)	0.0004	0.0006	0.0001	0.0002	0.5000	0.0012	0.0064	M6
PWY-6122: 5-aminoimidazole ribonucleotide biosynthesis II	0.0079	0.0119	0.0078	0.0106	0.4980	0.0012	0.0065	M6
PWY-6277: superpathway of 5-aminoimidazole ribonucleotide biosynthesis	0.0079	0.0119	0.0078	0.0106	0.4980	0.0012	0.0065	M6
RHAMCAT-PWY: L-rhamnose degradation I	0.0040	0.0026	0.0036	0.0023	-0.4920	0.0014	0.0072	base
PWY-5656: mannosylglycerate biosynthesis I	0.0001	0.0001	0.0000	0.0000	-0.4910	0.0015	0.0074	base
PWY-7204: pyridoxal 5'-phosphate salvage II (plants)	0.0009	0.0005	0.0006	0.0002	-0.4880	0.0016	0.0080	base
PWY-841: superpathway of purine nucleotides de novo biosynthesis I	0.0028	0.0042	0.0028	0.0044	0.4830	0.0017	0.0084	M6
THRESYN-PWY: superpathway of L-threonine biosynthesis	0.0052	0.0081	0.0053	0.0075	0.4830	0.0017	0.0084	M6
PWY0-1061: superpathway of L-alanine biosynthesis	0.0011	0.0019	0.0008	0.0012	0.4810	0.0018	0.0087	M6
PWY-7456: mannan degradation	0.0006	0.0001	0.0000	0.0000	-0.4770	0.0020	0.0093	base
PWY-1269: CMP-3-deoxy-D-manno-octulosonate biosynthesis I	0.0045	0.0031	0.0044	0.0023	-0.4740	0.0021	0.0100	base

PWY-6471: peptidoglycan biosynthesis IV (Enterococcus faecium)	0.0000	0.0002	0.0000	0.0000	0.4720	0.0022	0.0103	M6
PWY-4041: γ-glutamyl cycle	0.0008	0.0015	0.0003	0.0008	0.4690	0.0023	0.0104	M6
PWY-5345: superpathway of L-methionine biosynthesis (by sulfhydrylation)	0.0012	0.0009	0.0010	0.0008	-0.4690	0.0023	0.0104	base
PWY-6628: superpathway of L-phenylalanine biosynthesis	0.0016	0.0019	0.0012	0.0013	0.4510	0.0035	0.0155	M6
GLUCONEO-PWY: gluconeogenesis I	0.0013	0.0007	0.0009	0.0001	-0.4440	0.0040	0.0168	base
PWY-6859: all-trans-farnesol biosynthesis	0.0003	0.0005	0.0001	0.0002	0.4440	0.0040	0.0168	M6
PYRIDNUCSAL-PWY: NAD salvage pathway I	0.0007	0.0012	0.0005	0.0009	0.4440	0.0040	0.0168	M6
PWY-5505: L-glutamate and L-glutamine biosynthesis	0.0004	0.0003	0.0003	0.0001	-0.4430	0.0041	0.0172	base
PWY-6700: queuosine biosynthesis	0.0232	0.0134	0.0170	0.0107	-0.4410	0.0043	0.0177	base
PWY-5384: sucrose degradation IV (sucrose phosphorylase)	0.0005	0.0016	0.0003	0.0006	0.4370	0.0046	0.0189	M6
TRNA-CHARGING-PWY: tRNA charging	0.0015	0.0009	0.0011	0.0007	-0.4340	0.0050	0.0202	base
PWY-5659: GDP-mannose biosynthesis	0.0040	0.0026	0.0034	0.0023	-0.4290	0.0054	0.0215	base
PWY-5695: urate biosynthesis/inosine 5'-phosphate degradation	0.0139	0.0088	0.0133	0.0085	-0.4230	0.0061	0.0233	base
PWY-6969: TCA cycle V (2-oxoglutarate:ferredoxin oxidoreductase)	0.0019	0.0017	0.0016	0.0013	-0.4230	0.0061	0.0233	base
P122-PWY: heterolactic fermentation	0.0003	0.0006	0.0001	0.0002	0.4230	0.0061	0.0233	M6
P185-PWY: formaldehyde assimilation III (dihydroxyacetone cycle)	0.0006	0.0008	0.0002	0.0004	0.4180	0.0068	0.0255	M6
PWY0-1241: ADP-L-glycero-β-D-manno-heptose biosynthesis	0.0005	0.0009	0.0003	0.0004	0.4180	0.0068	0.0255	M6
SO4ASSIM-PWY: sulfate reduction I (assimilatory)	0.0020	0.0014	0.0009	0.0008	-0.4100	0.0079	0.0293	base
PWY-6125: superpathway of guanosine nucleotides de novo biosynthesis II	0.0065	0.0046	0.0062	0.0039	-0.4070	0.0082	0.0301	base
PWY-7663: gondoate biosynthesis (anaerobic)	0.0096	0.0063	0.0095	0.0061	-0.3980	0.0098	0.0357	base
PPGPPMET-PWY: ppGpp biosynthesis	0.0004	0.0007	0.0003	0.0003	0.3970	0.0102	0.0362	M6
PWY66-422: D-galactose degradation V (Leloir pathway)	0.0045	0.0059	0.0044	0.0051	0.3970	0.0102	0.0362	M6
PWY-5104: L-isoleucine biosynthesis IV	0.0008	0.0007	0.0005	0.0001	-0.3950	0.0106	0.0372	base
KETOGLUCONMET-PWY: ketogluconate metabolism	0.0002	0.0006	0.0001	0.0002	0.3920	0.0109	0.0381	M6
GOLPDLCAT-PWY: superpathway of glycerol degradation to 1,3-propanediol	0.0008	0.0015	0.0002	0.0004	0.3890	0.0118	0.0397	M6
PANTO-PWY: phosphopantothenate biosynthesis I	0.0140	0.0089	0.0146	0.0089	-0.3890	0.0118	0.0397	base
PWY4LZ-257: superpathway of fermentation (Chlamydomonas reinhardtii)	0.0003	0.0002	0.0002	0.0001	-0.3890	0.0118	0.0397	base
ARG+POLYAMINE-SYN: superpathway of arginine and polyamine biosynthesis	0.0016	0.0025	0.0013	0.0023	0.3870	0.0122	0.0403	M6
SER-GLYSYN-PWY: superpathway of L-serine and glycine biosynthesis I	0.0034	0.0046	0.0032	0.0041	0.3870	0.0122	0.0403	M6
PWY-6124: inosine-5'-phosphate biosynthesis II	0.0048	0.0067	0.0047	0.0057	0.3800	0.0140	0.0459	M6
1CMET2-PWY: N10-formyl-tetrahydrofolate biosynthesis	0.0109	0.0069	0.0112	0.0064	-0.3730	0.0155	0.0504	none
PWY-5367: petroselinate biosynthesis	0.0009	0.0013	0.0003	0.0009	0.3690	0.0166	0.0535	none
PWY-5973: cis-vaccenate biosynthesis	0.0098	0.0069	0.0102	0.0064	-0.3640	0.0184	0.0570	none
RIBOSYN2-PWY: flavin biosynthesis I (bacteria and plants)	0.0028	0.0020	0.0026	0.0016	-0.3640	0.0184	0.0570	none
GLYCOGENSYNTH-PWY: glycogen biosynthesis I (from ADP-D-Glucose)	0.0022	0.0032	0.0020	0.0022	0.3640	0.0184	0.0570	none
PWY-7357: thiamin formation from pyrithiamine and oxythiamine (yeast)	0.0069	0.0083	0.0058	0.0073	0.3640	0.0184	0.0570	none
PENTOSE-P-PWY: pentose phosphate pathway	0.0015	0.0021	0.0012	0.0015	0.3560	0.0211	0.0639	none
PWY-6317: galactose degradation I (Leloir pathway)	0.0045	0.0058	0.0044	0.0050	0.3560	0.0211	0.0639	none

PWY0-41: allantoin degradation IV (anaerobic)	0.0000	0.0000	0.0000	0.0000	-0.3530	0.0218	0.0643	none
PWY-6285: superpathway of fatty acids biosynthesis (E. coli)	0.0005	0.0009	0.0004	0.0005	0.3530	0.0218	0.0643	none
PWY-6385: peptidoglycan biosynthesis III (mycobacteria)	0.0111	0.0079	0.0121	0.0073	-0.3530	0.0218	0.0643	none
PWY-7371: 1,4-dihydroxy-6-naphthoate biosynthesis II	0.0006	0.0007	0.0002	0.0000	-0.3500	0.0232	0.0681	none
PWY-6897: thiamin salvage II	0.0078	0.0058	0.0071	0.0054	-0.3490	0.0240	0.0697	none
PWY-7228: superpathway of guanosine nucleotides de novo biosynthesis I	0.0073	0.0054	0.0069	0.0048	-0.3460	0.0248	0.0714	none
PWY-6123: inosine-5'-phosphate biosynthesis I	0.0056	0.0077	0.0055	0.0068	0.3440	0.0256	0.0731	none
PWY-6121: 5-aminoimidazole ribonucleotide biosynthesis I	0.0084	0.0106	0.0081	0.0097	0.3270	0.0341	0.0964	none
GLUCOSE1PMETAB-PWY: glucose and glucose-1-phosphate degradation	0.0008	0.0011	0.0004	0.0005	0.3260	0.0351	0.0985	none
PWY-3841: folate transformations II	0.0111	0.0076	0.0118	0.0068	-0.3220	0.0362	0.1008	none
PWY-5863: superpathway of phylloquinol biosynthesis	0.0006	0.0008	0.0003	0.0004	0.3210	0.0374	0.1030	none
SALVADEHYPOX-PWY: adenosine nucleotides degradation II	0.0010	0.0016	0.0005	0.0009	0.3180	0.0397	0.1086	none
PWY-6612: superpathway of tetrahydrofolate biosynthesis	0.0006	0.0008	0.0003	0.0003	0.3130	0.0422	0.1126	none
PWY-6126: superpathway of adenosine nucleotides de novo biosynthesis II	0.0081	0.0063	0.0080	0.0057	-0.3130	0.0422	0.1126	none
TCA: TCA cycle I (prokaryotic)	0.0021	0.0020	0.0017	0.0016	-0.3130	0.0422	0.1126	none
PWY-6527: stachyose degradation	0.0039	0.0050	0.0038	0.0044	0.3100	0.0448	0.1185	none
PWY-6284: superpathway of unsaturated fatty acids biosynthesis (E. coli)	0.0005	0.0008	0.0004	0.0004	0.3060	0.0475	0.1248	none
PWY-5920: superpathway of heme biosynthesis from glycine	0.0002	0.0002	0.0001	0.0001	-0.3020	0.0504	0.1273	none
PWY-7196: superpathway of pyrimidine ribonucleosides salvage	0.0025	0.0021	0.0020	0.0019	-0.3020	0.0504	0.1273	none
PYRIDNUCSYN-PWY: NAD biosynthesis I (from aspartate)	0.0039	0.0032	0.0033	0.0028	-0.3020	0.0504	0.1273	none
PWY0-1296: purine ribonucleosides degradation	0.0054	0.0068	0.0052	0.0067	0.3020	0.0504	0.1273	none
PWY-6353: purine nucleotides degradation II (aerobic)	0.0013	0.0019	0.0007	0.0013	0.3020	0.0504	0.1273	none
NONOXIPENT-PWY: pentose phosphate pathway (non-oxidative branch)	0.0056	0.0071	0.0059	0.0073	0.2980	0.0534	0.1339	none
GLYCOLYSIS-E-D: superpathway of glycolysis and Entner-Doudoroff	0.0014	0.0013	0.0010	0.0008	-0.2960	0.0549	0.1367	none
PWY-6282: palmitoleate biosynthesis I (from (5Z)-dodec-5-enoate)	0.0011	0.0016	0.0008	0.0009	0.2950	0.0565	0.1397	none
PWY-7220: adenosine deoxyribonucleotides de novo biosynthesis II	0.0047	0.0037	0.0044	0.0030	-0.2920	0.0582	0.1405	none
PWY-7222: guanosine deoxyribonucleotides de novo biosynthesis II	0.0047	0.0037	0.0044	0.0030	-0.2920	0.0582	0.1405	none
PWY-7388: octanoyl-[acyl-carrier protein] biosynthesis (mitochondria, yeast)	0.0009	0.0015	0.0005	0.0007	0.2920	0.0582	0.1405	none
POLYAMSYN-PWY: superpathway of polyamine biosynthesis I	0.0014	0.0020	0.0010	0.0016	0.2900	0.0599	0.1405	none
PWY0-1319: CDP-diacylglycerol biosynthesis II	0.0147	0.0102	0.0138	0.0091	-0.2900	0.0599	0.1405	none
PWY-5667: CDP-diacylglycerol biosynthesis I	0.0147	0.0102	0.0138	0.0091	-0.2900	0.0599	0.1405	none
PWY-5989: stearate biosynthesis II (bacteria and plants)	0.0016	0.0019	0.0013	0.0018	0.2900	0.0599	0.1405	none
PWY-5791: 1,4-dihydroxy-2-naphthoate biosynthesis II (plants)	0.0008	0.0010	0.0004	0.0004	0.2890	0.0616	0.1425	none
PWY-5837: 1,4-dihydroxy-2-naphthoate biosynthesis I	0.0008	0.0010	0.0004	0.0004	0.2890	0.0616	0.1425	none
FOLSYN-PWY: superpathway of tetrahydrofolate biosynthesis and salvage	0.0008	0.0010	0.0005	0.0004	0.2870	0.0633	0.1446	none
PWY-5188: tetrapyrrole biosynthesis I (from glutamate)	0.0021	0.0034	0.0018	0.0030	0.2870	0.0633	0.1446	none
PWY-724: superpathway of L-lysine, L-threonine and L-methionine biosynthesis II	0.0083	0.0102	0.0091	0.0102	0.2840	0.0651	0.1477	none
PWY-7187: pyrimidine deoxyribonucleotides de novo biosynthesis II	0.0014	0.0028	0.0011	0.0016	0.2810	0.0689	0.1551	none

PWY-6147: 6-hydroxymethyl-dihydropterin diphosphate biosynthesis I	0.0015	0.0019	0.0009	0.0014	0.2730	0.0768	0.1719	none
P124-PWY: Bifidobacterium shunt	0.0000	0.0001	0.0000	0.0000	0.2680	0.0811	0.1778	none
PHOSLIPSYN-PWY: superpathway of phospholipid biosynthesis I (bacteria)	0.0043	0.0035	0.0042	0.0034	-0.2680	0.0811	0.1778	none
PWY0-162: superpathway of pyrimidine ribonucleotides de novo biosynthesis	0.0066	0.0053	0.0065	0.0044	-0.2680	0.0811	0.1778	none
FASYN-INITIAL-PWY: superpathway of fatty acid biosynthesis initiation (E. coli)	0.0009	0.0015	0.0005	0.0008	0.2650	0.0856	0.1851	none
PWY0-862: (5Z)-dodec-5-enoate biosynthesis	0.0011	0.0016	0.0006	0.0009	0.2650	0.0856	0.1851	none
PWY-7211: superpathway of pyrimidine deoxyribonucleotides de novo biosynthesis	0.0028	0.0023	0.0025	0.0022	-0.2590	0.0926	0.1991	none
GLCMANNANAUT-PWY: superpathway of N-acetylglucosamine, N-acetylmannosamine and N-acetylneuraminate degradation	0.0035	0.0046	0.0033	0.0044	0.2560	0.0976	0.2070	none
PWY-5971: palmitate biosynthesis II (bacteria and plants)	0.0009	0.0014	0.0007	0.0007	0.2560	0.0976	0.2070	none
NONMEVIPP-PWY: methylerythritol phosphate pathway I	0.0163	0.0113	0.0152	0.0107	-0.2530	0.1001	0.2098	none
PWY-7229: superpathway of adenosine nucleotides de novo biosynthesis I	0.0097	0.0078	0.0095	0.0070	-0.2530	0.1001	0.2098	none
COA-PWY: coenzyme A biosynthesis I	0.0032	0.0045	0.0028	0.0037	0.2520	0.1027	0.2139	none
PWY-5083: NAD/NADH phosphorylation and dephosphorylation	0.0011	0.0013	0.0006	0.0006	0.2420	0.1166	0.2412	none
DENOVOPURINE2-PWY: superpathway of purine nucleotides de novo biosynthesis II	0.0015	0.0024	0.0012	0.0017	0.2380	0.1225	0.2519	none
ORNDEG-PWY: superpathway of ornithine degradation	0.0004	0.0005	0.0001	0.0001	0.2300	0.1351	0.2727	none
PWY-2942: L-lysine biosynthesis III	0.0218	0.0149	0.0170	0.0116	-0.2300	0.1351	0.2727	none
PWY-7664: oleate biosynthesis IV (anaerobic)	0.0012	0.0017	0.0007	0.0010	0.2300	0.1351	0.2727	none
BIOTIN-BIOSYNTHESIS-PWY: biotin biosynthesis I	0.0013	0.0012	0.0010	0.0007	-0.2270	0.1418	0.2827	none
PWY-7013: L-1,2-propanediol degradation	0.0005	0.0007	0.0002	0.0000	-0.2270	0.1418	0.2827	none
P164-PWY: purine nucleobases degradation I (anaerobic)	0.0003	0.0002	0.0002	0.0001	-0.2250	0.1452	0.2844	none
PWY-5918: superpathay of heme biosynthesis from glutamate	0.0005	0.0004	0.0003	0.0002	-0.2250	0.1452	0.2844	none
PWY-7184: pyrimidine deoxyribonucleotides de novo biosynthesis I	0.0044	0.0037	0.0043	0.0034	-0.2250	0.1452	0.2844	none
HEXITOLDEGSUPER-PWY: superpathway of hexitol degradation (bacteria)	0.0016	0.0019	0.0017	0.0017	0.2220	0.1487	0.2895	none
PWY-6545: pyrimidine deoxyribonucleotides de novo biosynthesis III	0.0034	0.0026	0.0033	0.0025	-0.2210	0.1522	0.2947	none
PWY-6901: superpathway of glucose and xylose degradation	0.0017	0.0019	0.0016	0.0015	0.2190	0.1559	0.2999	none
PWY-6113: superpathway of mycolate biosynthesis	0.0007	0.0010	0.0005	0.0005	0.2180	0.1595	0.3052	none
PWY-5690: TCA cycle II (plants and fungi)	0.0019	0.0022	0.0014	0.0015	-0.2140	0.1633	0.3088	none
PWY-6737: starch degradation V	0.0085	0.0097	0.0083	0.0098	0.2140	0.1633	0.3088	none
RUMP-PWY: formaldehyde oxidation I	0.0000	0.0000	0.0000	0.0000	-0.2110	0.1710	0.3215	none
THISYN-PWY: superpathway of thiamin diphosphate biosynthesis I	0.0025	0.0019	0.0019	0.0014	-0.2070	0.1789	0.3346	none
PWY-5097: L-lysine biosynthesis VI	0.0222	0.0153	0.0172	0.0122	-0.2040	0.1871	0.3422	none
PWY-6606: guanosine nucleotides degradation II	0.0004	0.0006	0.0003	0.0005	0.2040	0.1871	0.3422	none
PWY-6608: guanosine nucleotides degradation III	0.0015	0.0023	0.0009	0.0017	0.2040	0.1871	0.3422	none
PWY-7198: pyrimidine deoxyribonucleotides de novo biosynthesis IV	0.0004	0.0004	0.0002	0.0002	-0.2040	0.1871	0.3422	none
PWY0-42: 2-methylcitrate cycle I	0.0001	0.0001	0.0000	0.0000	-0.1990	0.1956	0.3558	none
FASYN-ELONG-PWY: fatty acid elongation -- saturated	0.0013	0.0017	0.0008	0.0012	0.1980	0.2000	0.3617	none
PWY-7210: pyrimidine deoxyribonucleotides biosynthesis from CTP	0.0005	0.0005	0.0003	0.0002	-0.1960	0.2044	0.3677	none

HEME-BIOSYNTHESIS-II: heme biosynthesis I (aerobic)	0.0005	0.0003	0.0003	0.0002	-0.1940	0.2089	0.3737	none
PWY-6507: 4-deoxy-L-threo-hex-4-enopyranuronate degradation	0.0024	0.0031	0.0020	0.0028	0.1900	0.2181	0.3881	none
PWY0-1298: superpathway of pyrimidine deoxyribonucleosides degradation	0.0007	0.0010	0.0005	0.0005	0.1880	0.2228	0.3944	none
ARO-PWY: chorismate biosynthesis I	0.0101	0.0107	0.0101	0.0110	0.1840	0.2324	0.4071	none
PWY0-1415: superpathway of heme biosynthesis from uroporphyrinogen-III	0.0004	0.0002	0.0002	0.0001	-0.1840	0.2324	0.4071	none
PWY-7328: superpathway of UDP-glucose-derived O-antigen building blocks biosynthesis	0.0003	0.0004	0.0003	0.0001	-0.1820	0.2374	0.4135	none
ENTBACSYN-PWY: enterobactin biosynthesis	0.0010	0.0015	0.0004	0.0004	0.1790	0.2474	0.4266	none
PWY-5484: glycolysis II (from fructose 6-phosphate)	0.0038	0.0033	0.0035	0.0029	-0.1790	0.2474	0.4266	none
ILEUSYN-PWY: L-isoleucine biosynthesis I (from threonine)	0.0140	0.0143	0.0142	0.0151	0.1740	0.2578	0.4376	none
PWY-6630: superpathway of L-tyrosine biosynthesis	0.0014	0.0015	0.0009	0.0009	0.1740	0.2578	0.4376	none
VALSYN-PWY: L-valine biosynthesis	0.0140	0.0143	0.0142	0.0151	0.1740	0.2578	0.4376	none
PWY-7242: D-fructuronate degradation	0.0027	0.0033	0.0027	0.0030	0.1730	0.2631	0.4421	none
PWY-5138: unsaturated, even numbered fatty acid β-oxidation	0.0002	0.0002	0.0000	0.0000	-0.1710	0.2685	0.4421	none
PWY-5897: superpathway of menaquinol-11 biosynthesis	0.0017	0.0018	0.0012	0.0010	0.1710	0.2685	0.4421	none
PWY-5898: superpathway of menaquinol-12 biosynthesis	0.0017	0.0018	0.0012	0.0010	0.1710	0.2685	0.4421	none
PWY-5899: superpathway of menaquinol-13 biosynthesis	0.0017	0.0018	0.0012	0.0010	0.1710	0.2685	0.4421	none
REDCITCYC: TCA cycle VIII (helicobacter)	0.0006	0.0006	0.0004	0.0003	0.1710	0.2685	0.4421	none
PWY-5705: allantoin degradation to glyoxylate III	0.0002	0.0004	0.0001	0.0001	0.1670	0.2794	0.4579	none
PWY-7199: pyrimidine deoxyribonucleosides salvage	0.0024	0.0021	0.0021	0.0021	-0.1650	0.2850	0.4648	none
COMPLETE-ARO-PWY: superpathway of aromatic amino acid biosynthesis	0.0105	0.0109	0.0108	0.0107	0.1640	0.2907	0.4671	none
PWY-561: superpathway of glyoxylate cycle and fatty acid degradation	0.0006	0.0006	0.0003	0.0002	-0.1640	0.2907	0.4671	none
PWY-6263: superpathway of menaquinol-8 biosynthesis II	0.0003	0.0002	0.0001	0.0000	-0.1640	0.2907	0.4671	none
METHGLYUT-PWY: superpathway of methylglyoxal degradation	0.0002	0.0003	0.0001	0.0001	0.1600	0.2965	0.4718	none
PWY-6609: adenine and adenosine salvage III	0.0058	0.0061	0.0057	0.0052	0.1600	0.2965	0.4718	none
P461-PWY: hexitol fermentation to lactate, formate, ethanol and acetate	0.0018	0.0028	0.0011	0.0019	0.1570	0.3082	0.4858	none
PWY-5747: 2-methylcitrate cycle II	0.0001	0.0001	0.0000	0.0000	-0.1570	0.3082	0.4858	none
PWY-4702: phytate degradation I	0.0003	0.0004	0.0001	0.0001	-0.1520	0.3263	0.5095	none
HEMESYN2-PWY: heme biosynthesis II (anaerobic)	0.0009	0.0013	0.0006	0.0008	0.1520	0.3263	0.5095	none
GLYCOCAT-PWY: glycogen degradation I (bacterial)	0.0006	0.0006	0.0004	0.0004	-0.1440	0.3516	0.5413	none
PWY-1042: glycolysis IV (plant cytosol)	0.0115	0.0100	0.0110	0.0097	-0.1440	0.3516	0.5413	none
PWY-7111: pyruvate fermentation to isobutanol (engineered)	0.0142	0.0144	0.0144	0.0147	0.1440	0.3516	0.5413	none
PWY-5860: superpathway of demethylmenaquinol-6 biosynthesis I	0.0008	0.0010	0.0004	0.0003	0.1400	0.3647	0.5562	none
PWY-6595: superpathway of guanosine nucleotides degradation (plants)	0.0002	0.0001	0.0002	0.0000	-0.1400	0.3647	0.5562	none
P23-PWY: reductive TCA cycle I	0.0000	0.0001	0.0000	0.0000	0.1380	0.3713	0.5587	none
PWY-5862: superpathway of demethylmenaquinol-9 biosynthesis	0.0008	0.0010	0.0004	0.0003	0.1380	0.3713	0.5587	none
PWY-7237: myo-, chiro- and scillo-inositol degradation	0.0015	0.0026	0.0003	0.0004	0.1380	0.3713	0.5587	none
PRPP-PWY: superpathway of histidine, purine, and pyrimidine biosynthesis	0.0013	0.0016	0.0012	0.0014	0.1360	0.3780	0.5662	none
PWY-6519: 8-amino-7-oxononanoate biosynthesis I	0.0011	0.0011	0.0008	0.0005	-0.1340	0.3848	0.5738	none

PWY-1861: formaldehyde assimilation II (RuMP Cycle)	0.0000	0.0000	0.0000	0.0000	-0.1320	0.3917	0.5814	none
GLYCOLYSIS: glycolysis I (from glucose 6-phosphate)	0.0039	0.0036	0.0037	0.0030	-0.1300	0.3987	0.5865	none
PWY-6305: putrescine biosynthesis IV	0.0017	0.0022	0.0015	0.0018	0.1300	0.3987	0.5865	none
PANTOSYN-PWY: pantothenate and coenzyme A biosynthesis I	0.0052	0.0048	0.0048	0.0044	-0.1260	0.4128	0.6019	none
PROTOCATECHUATE-ORTHO-CLEAVAGE-PWY: protocatechuate degradation II (ortho-cleavage pathway)	0.0001	0.0001	0.0000	0.0000	-0.1260	0.4128	0.6019	none
CALVIN-PWY: Calvin-Benson-Bassham cycle	0.0072	0.0077	0.0077	0.0075	0.1240	0.4200	0.6097	none
COBALSYN-PWY: adenosylcobalamin salvage from cobinamide I	0.0035	0.0036	0.0034	0.0032	0.1230	0.4272	0.6175	none
PWY-6892: thiazole biosynthesis I (E. coli)	0.0009	0.0012	0.0004	0.0004	0.1210	0.4345	0.6253	none
PWY-5861: superpathway of demethylmenaquinol-8 biosynthesis	0.0010	0.0011	0.0007	0.0005	0.1190	0.4419	0.6332	none
GLYCOLYSIS-TCA-GLYOX-BYPASS: superpathway of glycolysis, pyruvate dehydrogenase, TCA, and glyoxylate bypass	0.0011	0.0012	0.0007	0.0005	-0.1150	0.4569	0.6463	none
PEPTIDOGLYCANSYN-PWY: peptidoglycan biosynthesis I (meso-diaminopimelate containing)	0.0182	0.0145	0.0181	0.0144	-0.1150	0.4569	0.6463	none
PWY-7197: pyrimidine deoxyribonucleotide phosphorylation	0.0033	0.0032	0.0032	0.0027	-0.1150	0.4569	0.6463	none
PWY-5177: glutaryl-CoA degradation	0.0014	0.0019	0.0012	0.0015	0.1110	0.4722	0.6650	none
LPSSYN-PWY: superpathway of lipopolysaccharide biosynthesis	0.0002	0.0003	0.0001	0.0000	-0.1050	0.4956	0.6864	none
PWY4FS-7: phosphatidylglycerol biosynthesis I (plastidic)	0.0036	0.0035	0.0033	0.0028	-0.1050	0.4956	0.6864	none
PWY4FS-8: phosphatidylglycerol biosynthesis II (non-plastidic)	0.0036	0.0035	0.0033	0.0028	-0.1050	0.4956	0.6864	none
PWY-5676: acetyl-CoA fermentation to butanoate II	0.0009	0.0016	0.0007	0.0005	-0.1050	0.4956	0.6864	none
P441-PWY: superpathway of N-acetylneuraminate degradation	0.0012	0.0014	0.0010	0.0011	0.1030	0.5035	0.6916	none
PWY-6386: UDP-N-acetylmuramoyl-pentapeptide biosynthesis II (lysine-containing)	0.0181	0.0145	0.0184	0.0141	-0.1030	0.5035	0.6916	none
PWY-6163: chorismate biosynthesis from 3-dehydroquinate	0.0103	0.0102	0.0100	0.0102	0.1010	0.5115	0.6939	none
PWY-6387: UDP-N-acetylmuramoyl-pentapeptide biosynthesis I (meso-diaminopimelate containing)	0.0173	0.0140	0.0172	0.0140	-0.1010	0.5115	0.6939	none
PWY-6891: thiazole biosynthesis II (Bacillus)	0.0007	0.0008	0.0003	0.0003	0.1010	0.5115	0.6939	none
PWY-5845: superpathway of menaquinol-9 biosynthesis	0.0011	0.0011	0.0007	0.0005	0.0974	0.5278	0.7044	none
PWY-5850: superpathway of menaquinol-6 biosynthesis I	0.0011	0.0011	0.0007	0.0005	0.0974	0.5278	0.7044	none
PWY-5896: superpathway of menaquinol-10 biosynthesis	0.0011	0.0011	0.0007	0.0005	0.0974	0.5278	0.7044	none
PWY-6895: superpathway of thiamin diphosphate biosynthesis II	0.0020	0.0016	0.0013	0.0012	-0.0974	0.5278	0.7044	none
PWY0-166: superpathway of pyrimidine deoxyribonucleotides de novo biosynthesis (E. coli)	0.0050	0.0046	0.0049	0.0045	-0.0917	0.5526	0.7345	none
3-HYDROXYPHENYLACETATE-DEGRADATION-PWY: 4-hydroxyphenylacetate degradation	0.0002	0.0002	0.0000	0.0000	-0.0897	0.5610	0.7391	none
PWY-7409: phospholipid remodeling (phosphatidylethanolamine, yeast)	0.0004	0.0006	0.0001	0.0001	0.0897	0.5610	0.7391	none
GLYCOL-GLYOXDEG-PWY: superpathway of glycol metabolism and degradation	0.0003	0.0004	0.0001	0.0001	-0.0878	0.5694	0.7391	none
P105-PWY: TCA cycle IV (2-oxoglutarate decarboxylase)	0.0009	0.0011	0.0005	0.0004	-0.0878	0.5694	0.7391	none
PWY-5154: L-arginine biosynthesis III (via N-acetyl-L-citrulline)	0.0020	0.0020	0.0019	0.0018	-0.0878	0.5694	0.7391	none
PWY-7039: phosphatidate metabolism, as a signaling molecule	0.0001	0.0002	0.0000	0.0000	-0.0878	0.5694	0.7391	none

NAGLIPASYN-PWY: lipid IVA biosynthesis	0.0012	0.0014	0.0008	0.0010	0.0839	0.5865	0.7554	none
PWY0-1277: 3-phenylpropanoate and 3-(3-hydroxyphenyl)propanoate degradation	0.0005	0.0006	0.0002	0.0002	0.0839	0.5865	0.7554	none
ANAGLYCOLYSIS-PWY: glycolysis III (from glucose)	0.0090	0.0081	0.0094	0.0077	-0.0801	0.6038	0.7680	none
PWY-5136: fatty acid β-oxidation II (peroxisome)	0.0004	0.0005	0.0003	0.0003	-0.0801	0.6038	0.7680	none
HCAMHPDEG-PWY: 3-phenylpropanoate and 3-(3-hydroxyphenyl)propanoate degradation to 2-oxopent-4-enoate	0.0004	0.0005	0.0001	0.0001	0.0781	0.6126	0.7680	none
PWY-5723: Rubisco shunt	0.0009	0.0011	0.0007	0.0006	0.0781	0.6126	0.7680	none
PWY-5838: superpathway of menaquinol-8 biosynthesis I	0.0014	0.0013	0.0010	0.0007	0.0781	0.6126	0.7680	none
PWY-6690: cinnamate and 3-hydroxycinnamate degradation to 2-oxopent-4-enoate	0.0004	0.0005	0.0001	0.0001	0.0781	0.6126	0.7680	none
PWY-7221: guanosine ribonucleotides de novo biosynthesis	0.0219	0.0175	0.0180	0.0161	-0.0781	0.6126	0.7680	none
COA-PWY-1: coenzyme A biosynthesis II (mammalian)	0.0140	0.0141	0.0147	0.0136	0.0742	0.6302	0.7784	none
PWY0-321: phenylacetate degradation I (aerobic)	0.0002	0.0003	0.0000	0.0000	0.0742	0.6302	0.7784	none
PWY-6823: molybdenum cofactor biosynthesis	0.0003	0.0005	0.0001	0.0001	0.0742	0.6302	0.7784	none
THISYNARA-PWY: superpathway of thiamin diphosphate biosynthesis III (eukaryotes)	0.0043	0.0039	0.0043	0.0036	-0.0742	0.6302	0.7784	none
PWY-2723: trehalose degradation V	0.0004	0.0005	0.0001	0.0001	0.0724	0.6392	0.7865	none
FUCCAT-PWY: fucose degradation	0.0007	0.0009	0.0004	0.0003	0.0704	0.6481	0.7887	none
PWY0-1479: tRNA processing	0.0005	0.0007	0.0004	0.0003	-0.0704	0.6481	0.7887	none
SULFATE-CYS-PWY: superpathway of sulfate assimilation and cysteine biosynthesis	0.0012	0.0013	0.0007	0.0008	-0.0704	0.6481	0.7887	none
UBISYN-PWY: superpathway of ubiquinol-8 biosynthesis (prokaryotic)	0.0005	0.0006	0.0002	0.0002	0.0685	0.6571	0.7967	none
PWY-5840: superpathway of menaquinol-7 biosynthesis	0.0013	0.0013	0.0011	0.0008	0.0665	0.6662	0.8019	none
PWY-6467: Kdo transfer to lipid IVA III (Chlamydia)	0.0000	0.0000	0.0000	0.0000	-0.0665	0.6662	0.8019	none
P162-PWY: L-glutamate degradation V (via hydroxyglutarate)	0.0001	0.0002	0.0000	0.0000	0.0647	0.6753	0.8099	none
PWY-3781: aerobic respiration I (cytochrome c)	0.0000	0.0000	0.0000	0.0000	0.0626	0.6845	0.8107	none
FAO-PWY: fatty acid β-oxidation I	0.0005	0.0006	0.0004	0.0004	-0.0608	0.6937	0.8107	none
PWY-7208: superpathway of pyrimidine nucleobases salvage	0.0056	0.0056	0.0055	0.0044	-0.0608	0.6937	0.8107	none
PWYG-321: mycolate biosynthesis	0.0012	0.0015	0.0007	0.0007	0.0608	0.6937	0.8107	none
AEROBACTINSYN-PWY: aerobactin biosynthesis	0.0000	0.0001	0.0000	0.0000	-0.0588	0.7029	0.8107	none
ARGDEG-PWY: superpathway of L-arginine, putrescine, and 4-aminobutanoate degradation	0.0003	0.0004	0.0001	0.0001	0.0588	0.7029	0.8107	none
AST-PWY: L-arginine degradation II (AST pathway)	0.0003	0.0004	0.0001	0.0001	0.0588	0.7029	0.8107	none
ORNARGDEG-PWY: superpathway of L-arginine and L-ornithine degradation	0.0003	0.0004	0.0001	0.0001	0.0588	0.7029	0.8107	none
PWY-5189: tetrapyrrole biosynthesis II (from glycine)	0.0006	0.0008	0.0003	0.0003	0.0588	0.7029	0.8107	none
PWY-7269: NAD/NADP-NADH/NADPH mitochondrial interconversion (yeast)	0.0007	0.0009	0.0003	0.0002	0.0588	0.7029	0.8107	none
PWY-821: superpathway of sulfur amino acid biosynthesis (Saccharomyces cerevisiae)	0.0002	0.0002	0.0001	0.0001	-0.0588	0.7029	0.8107	none
DTDPRHAMSYN-PWY: dTDP-L-rhamnose biosynthesis I	0.0060	0.0056	0.0058	0.0044	-0.0549	0.7216	0.8208	none
HSERMETANA-PWY: L-methionine biosynthesis III	0.0014	0.0014	0.0013	0.0011	-0.0549	0.7216	0.8208	none
PWY-5686: UMP biosynthesis	0.0218	0.0170	0.0177	0.0157	-0.0549	0.7216	0.8208	none
TRPSYN-PWY: L-tryptophan biosynthesis	0.0043	0.0042	0.0040	0.0036	0.0549	0.7216	0.8208	none

PWY66-400: glycolysis VI (metazoan)	0.0044	0.0046	0.0039	0.0039	-0.0531	0.7310	0.8258	none
PWY-7279: aerobic respiration II (cytochrome c) (yeast)	0.0000	0.0000	0.0000	0.0000	0.0531	0.7310	0.8258	none
ECASYN-PWY: enterobacterial common antigen biosynthesis	0.0004	0.0006	0.0001	0.0001	0.0511	0.7404	0.8336	none
GALACTARDEG-PWY: D-galactarate degradation I	0.0004	0.0005	0.0001	0.0001	0.0492	0.7498	0.8385	none
GLUCARGALACTSUPER-PWY: superpathway of D-glucarate and D-galactarate degradation	0.0004	0.0005	0.0001	0.0001	0.0492	0.7498	0.8385	none
FUC-RHAMCAT-PWY: superpathway of fucose and rhamnose degradation	0.0008	0.0009	0.0004	0.0004	-0.0472	0.7593	0.8406	none
GLUCUROCAT-PWY: superpathway of β-D-glucuronide and D-glucuronate degradation	0.0027	0.0031	0.0027	0.0030	0.0472	0.7593	0.8406	none
PWY-6629: superpathway of L-tryptophan biosynthesis	0.0012	0.0012	0.0005	0.0004	-0.0472	0.7593	0.8406	none
PWY-5415: catechol degradation I (meta-cleavage pathway)	0.0001	0.0001	0.0000	0.0000	-0.0434	0.7785	0.8448	none
PWY-5855: ubiquinol-7 biosynthesis (prokaryotic)	0.0005	0.0006	0.0002	0.0002	0.0434	0.7785	0.8448	none
PWY-5856: ubiquinol-9 biosynthesis (prokaryotic)	0.0005	0.0006	0.0002	0.0002	0.0434	0.7785	0.8448	none
PWY-5857: ubiquinol-10 biosynthesis (prokaryotic)	0.0005	0.0006	0.0002	0.0002	0.0434	0.7785	0.8448	none
PWY-6708: ubiquinol-8 biosynthesis (prokaryotic)	0.0005	0.0006	0.0002	0.0002	0.0434	0.7785	0.8448	none
PWY-6803: phosphatidylcholine acyl editing	0.0008	0.0008	0.0004	0.0003	0.0434	0.7785	0.8448	none
P161-PWY: acetylene degradation	0.0006	0.0009	0.0004	0.0005	0.0415	0.7881	0.8497	none
PWY-5005: biotin biosynthesis II	0.0008	0.0007	0.0005	0.0003	-0.0415	0.7881	0.8497	none
PWY-7446: sulfoglycolysis	0.0004	0.0004	0.0001	0.0000	-0.0395	0.7977	0.8573	none
PWY-7315: dTDP-N-acetylthomosamine biosynthesis	0.0007	0.0009	0.0002	0.0002	0.0338	0.8268	0.8857	none
PWY-5417: catechol degradation III (ortho-cleavage pathway)	0.0000	0.0000	0.0000	0.0000	-0.0318	0.8365	0.8875	none
PWY-5431: aromatic compounds degradation via β-ketoadipate	0.0000	0.0000	0.0000	0.0000	-0.0318	0.8365	0.8875	none
PWY-7539: 6-hydroxymethyl-dihydropterin diphosphate biosynthesis III (Chlamydia)	0.0013	0.0014	0.0008	0.0009	0.0318	0.8365	0.8875	none
GALACTUROCAT-PWY: D-galacturonate degradation I	0.0029	0.0031	0.0026	0.0027	-0.0299	0.8463	0.8950	none
GLUCARDEG-PWY: D-glucarate degradation I	0.0004	0.0006	0.0002	0.0001	-0.0279	0.8561	0.9025	none
PWY0-1586: peptidoglycan maturation (meso-diaminopimelate containing)	0.0042	0.0046	0.0037	0.0037	-0.0261	0.8660	0.9071	none
PWY-6588: pyruvate fermentation to acetone	0.0002	0.0003	0.0001	0.0001	-0.0261	0.8660	0.9071	none
PWY0-1533: methylphosphonate degradation I	0.0004	0.0006	0.0001	0.0001	0.0241	0.8758	0.9145	none
GALACT-GLUCUROCAT-PWY: superpathway of hexuronide and hexuronate degradation	0.0029	0.0031	0.0028	0.0028	-0.0222	0.8857	0.9161	none
ANAEROFRUCAT-PWY: homolactic fermentation	0.0042	0.0042	0.0038	0.0043	0.0222	0.8857	0.9161	none
PWY-7003: glycerol degradation to butanol	0.0002	0.0003	0.0001	0.0002	0.0222	0.8857	0.9161	none
CATECHOL-ORTHO-CLEAVAGE-PWY: catechol degradation to β-ketoadipate	0.0000	0.0000	0.0000	0.0000	-0.0202	0.8955	0.9234	none
PWY0-1338: polymyxin resistance	0.0003	0.0005	0.0001	0.0001	0.0184	0.9054	0.9308	none
ASPASN-PWY: superpathway of L-aspartate and L-asparagine biosynthesis	0.0051	0.0050	0.0049	0.0053	0.0164	0.9154	0.9351	none
PWY-7219: adenosine ribonucleotides de novo biosynthesis	0.0279	0.0236	0.0241	0.0234	-0.0164	0.9154	0.9351	none
GLYOXYLATE-BYPASS: glyoxylate cycle	0.0007	0.0009	0.0004	0.0003	0.0145	0.9253	0.9395	none
PWY-6151: S-adenosyl-L-methionine cycle I	0.0207	0.0172	0.0153	0.0150	-0.0145	0.9253	0.9395	none
PWY-7254: TCA cycle VII (acetate-producers)	0.0006	0.0008	0.0002	0.0002	0.0106	0.9452	0.9567	none

PWY-4242: pantothenate and coenzyme A biosynthesis III	0.0024	0.0028	0.0018	0.0022	0.0068	0.9651	0.9739	none
PWY-6185: 4-methylcatechol degradation (ortho cleavage)	0.0000	0.0000	0.0000	0.0000	-0.0048	0.9751	0.9810	none
KDO-NAGLIPASYN-PWY: superpathway of (Kdo)2-lipid A biosynthesis	0.0003	0.0004	0.0001	0.0001	-0.0029	0.9850	0.9880	none
TCA-GLYOX-BYPASS: superpathway of glyoxylate bypass and TCA	0.0008	0.0010	0.0004	0.0003	0.0010	0.9950	0.9950	none

ESM Table 4: Comparison of genera/species/pathway (centered log-ratio, clr-transformed) relative abundances between baseline and 6-month in the vildagliptin group

ESM Table 4a: Comparison of genera relative abundances between baseline and 6-month in the vildagliptin group

Genus	Relative abundance (mean)		Relative abundance (median)		Baseline vs. M6 (Wilcoxon signed-rank test, clr-transformed)			
	Baseline	6-month	Baseline	6-month	Effect_size	P-value	BH adjusted P-value	Enrichment
Paraprevotella	1.1004	0.5379	0.0664	0.0057	-0.6810	0.0000	0.0008	base
Fusobacterium	0.0055	0.1986	0.0000	0.0000	-0.5270	0.0009	0.0113	base
Bifidobacterium	0.3325	0.9385	0.0831	0.3285	0.5270	0.0009	0.0113	M6
Parabacteroides	3.7108	2.3650	2.2664	1.2356	-0.5250	0.0009	0.0113	base
Bacteroides	34.5427	26.1120	29.9078	22.5822	-0.5000	0.0016	0.0158	base
Veillonella	0.1168	0.3084	0.0050	0.0177	0.3680	0.0201	0.1485	none
Haemophilus	0.0681	0.3007	0.0000	0.0099	0.3650	0.0208	0.1485	none
Barnesiella	0.4808	0.4755	0.0000	0.0000	-0.3380	0.0326	0.1810	none
Odoribacter	0.6190	0.3025	0.1988	0.0979	-0.3380	0.0326	0.1810	none
Parasutterella	0.2007	0.1067	0.0440	0.0185	-0.3080	0.0513	0.2233	none
Subdoligranulum	1.0024	2.0975	0.6307	1.2723	0.3080	0.0513	0.2233	none
Streptococcus	0.0714	0.3381	0.0116	0.0183	0.3040	0.0546	0.2233	none
Acidaminococcus	0.0441	0.0303	0.0000	0.0000	-0.3000	0.0581	0.2233	none
Collinsella	0.0568	0.1723	0.0399	0.0750	0.2770	0.0806	0.2878	none
Gemella	0.0002	0.0008	0.0000	0.0000	-0.2640	0.0956	0.3186	none
Faecalibacterium	5.8688	8.5160	3.7569	7.6480	0.2420	0.1254	0.3920	none
Prevotella	17.9564	16.9113	0.0693	0.0312	-0.2120	0.1789	0.5182	none
Alistipes	5.3330	3.8962	2.4092	1.6621	-0.2090	0.1878	0.5182	none
Dorea	0.2012	0.4036	0.1117	0.2009	0.2040	0.1969	0.5182	none
Enterobacter	0.0977	0.1998	0.0000	0.0005	0.1900	0.2316	0.5768	none
Eubacterium	5.7510	6.7748	3.2167	4.8596	0.1850	0.2422	0.5768	none
Clostridium	0.5066	0.5105	0.0626	0.1364	0.1710	0.2822	0.6415	none
Megasphaera	1.5111	1.4149	0.0000	0.0000	-0.1530	0.3332	0.7243	none
Oxalobacter	0.0090	0.0398	0.0000	0.0000	0.1380	0.3823	0.7646	none
Ruminococcus	1.7994	2.0397	0.7845	1.3893	0.1380	0.3823	0.7646	none
Citrobacter	0.0156	0.1326	0.0000	0.0000	0.1250	0.4278	0.8226	none
Holdemania	0.0178	0.0282	0.0119	0.0095	-0.1170	0.4597	0.8514	none
Adlercreutzia	0.0162	0.0277	0.0074	0.0041	-0.1080	0.4930	0.8522	none

Klebsiella	0.5134	1.1125	0.0235	0.0849	0.1040	0.5101	0.8522	none
Dialister	1.1785	0.8252	0.0000	0.0000	0.0977	0.5364	0.8522	none
Flavonifractor	0.0334	0.0349	0.0044	0.0057	0.0934	0.5542	0.8522	none
Roseburia	2.1987	3.0241	1.0383	1.9006	0.0851	0.5908	0.8522	none
Anaerostipes	0.0335	0.0597	0.0030	0.0063	0.0786	0.6190	0.8522	none
Escherichia	2.4577	3.2382	0.3213	0.3758	-0.0786	0.6190	0.8522	none
Granulicatella	0.0008	0.0022	0.0000	0.0000	0.0786	0.6190	0.8522	none
Megamonas	6.4217	8.4684	0.0017	0.0007	0.0765	0.6285	0.8522	none
Anaerotruncus	0.0030	0.0091	0.0000	0.0000	0.0743	0.6380	0.8522	none
Lactobacillus	0.0302	0.0151	0.0000	0.0000	0.0723	0.6477	0.8522	none
Bilophila	0.2490	0.2871	0.0965	0.0705	-0.0637	0.6868	0.8805	none
Actinomyces	0.0004	0.0014	0.0000	0.0000	0.0511	0.7470	0.8805	none
Akkermansia	0.1262	0.3447	0.0000	0.0000	-0.0511	0.7470	0.8805	none
Rothia	0.0069	0.0393	0.0000	0.0003	0.0489	0.7572	0.8805	none
Solobacterium	0.0001	0.0006	0.0000	0.0000	0.0489	0.7572	0.8805	none
Blautia	0.9387	1.3393	0.3813	0.4246	0.0425	0.7881	0.8955	none
Enterococcus	0.0137	0.0383	0.0000	0.0000	0.0255	0.8719	0.9414	none
Eggerthella	0.0070	0.0158	0.0002	0.0010	0.0234	0.8825	0.9414	none
Pyramidobacter	0.0076	0.0052	0.0000	0.0000	0.0212	0.8931	0.9414	none
Coprococcus	0.7645	0.5796	0.1907	0.2112	0.0191	0.9037	0.9414	none
Coprobacillus	0.0027	0.0227	0.0000	0.0000	0.0149	0.9250	0.9439	none
Oscillibacter	0.4137	1.1118	0.1443	0.1224	0.0000	1.0000	1.0000	none

EMS Table 4b: Comparison of species relative abundances between baseline and 6-month in the vildagliptin group

Species	Relative abundance (mean)		Relative abundance (median)		Baseline vs. M6 (Wilcoxon signed-rank test, clr-transformed)			
	Baseline	6-month	Baseline	6-month	Effect_size	P-value	BH adjusted P-value	Enrichment
Paraprevotella clara	0.3892	0.1780	0.0000	0.0000	-0.6470	0.0000	0.0030	base
Bacteroides finegoldii	0.5943	0.3272	0.0236	0.0004	-0.6340	0.0001	0.0030	base
Paraprevotella xylaniphila	0.1009	0.0510	0.0003	0.0000	-0.6250	0.0001	0.0030	base
Bacteroides plebeius	5.0819	3.2872	0.9545	0.5917	-0.5660	0.0003	0.0095	base
Clostridium bartlettii	0.0269	0.1070	0.0030	0.0282	0.5600	0.0004	0.0095	M6
Bacteroides caccae	0.8060	0.6491	0.3889	0.1974	-0.5060	0.0014	0.0241	base
Bacteroides eggerthii	0.9208	0.3967	0.0000	0.0000	-0.5040	0.0014	0.0241	base
Parabacteroides distasonis	0.6618	0.2717	0.0805	0.0228	-0.4870	0.0021	0.0305	base

Bifidobacterium adolescentis	0.0874	0.3856	0.0000	0.0021	0.4780	0.0025	0.0324	M6
Bacteroides thetaiotaomicron	0.9007	1.1450	0.4606	0.2024	-0.4680	0.0031	0.0363	base
Odoribacter splanchnicus	0.6075	0.3020	0.1988	0.0979	-0.4300	0.0066	0.0705	base
Alistipes senegalensis	0.0430	0.0144	0.0001	0.0000	-0.4100	0.0095	0.0769	base
Bacteroides stercoris	6.2701	4.9550	1.3269	0.7765	-0.4100	0.0095	0.0769	base
Bacteroides xylanisolvens	0.4625	0.3428	0.0843	0.0388	-0.4100	0.0095	0.0769	base
Alistipes finegoldii	0.2457	0.1173	0.0239	0.0101	-0.4080	0.0099	0.0769	base
Parabacteroides goldsteinii	0.0309	0.0272	0.0000	0.0000	-0.3890	0.0139	0.1017	none
Haemophilus parainfluenzae	0.0679	0.2849	0.0000	0.0099	0.3840	0.0150	0.1031	none
Coprococcus comes	0.1000	0.2239	0.0402	0.1135	0.3810	0.0161	0.1048	none
Alistipes shahii	0.9039	0.5125	0.6056	0.1313	-0.3700	0.0193	0.1191	none
Bacteroides cellulosilyticus	0.2780	0.1729	0.0444	0.0038	-0.3640	0.0215	0.1260	none
Bacteroides massiliensis	3.3909	2.5433	0.3393	0.1291	-0.3570	0.0239	0.1334	none
Bifidobacterium longum	0.1358	0.3079	0.0188	0.0460	0.3460	0.0285	0.1513	none
Bacteroides uniformis	3.4981	2.1614	0.9010	0.2170	-0.3270	0.0385	0.1956	none
Parabacteroides merdae	2.0304	1.4341	0.5268	0.4436	-0.3230	0.0410	0.2001	none
Lachnospiraceae bacterium 1 1 57FA	0.3272	0.4675	0.1029	0.1226	0.3160	0.0452	0.2116	none
Faecalibacterium prausnitzii	5.8688	8.5160	3.7569	7.6480	0.3100	0.0497	0.2237	none
Bacteroides intestinalis	0.1473	0.1278	0.0000	0.0000	-0.3020	0.0563	0.2426	none
Bacteroides salyersiae	0.1519	0.1191	0.0000	0.0000	-0.3000	0.0581	0.2426	none
Streptococcus infantis	0.0007	0.0003	0.0000	0.0000	-0.2860	0.0696	0.2705	none
Bacteroides dorei	0.9845	1.4844	0.0311	0.0082	-0.2850	0.0717	0.2705	none
Eubacterium biforme	0.0491	0.0384	0.0000	0.0000	-0.2850	0.0717	0.2705	none
Ruminococcus bromii	1.1614	1.1465	0.0090	0.1802	0.2780	0.0783	0.2854	none
Bacteroides coprocola	1.8204	2.3622	0.0000	0.0000	-0.2770	0.0806	0.2854	none
Parasutterella excrementihominis	0.2007	0.1067	0.0440	0.0185	-0.2740	0.0829	0.2854	none
Ruminococcus obeum	0.1200	0.1479	0.0824	0.0956	0.2700	0.0878	0.2935	none
Veillonella parvula	0.0697	0.0845	0.0009	0.0068	0.2660	0.0929	0.3020	none
Eubacterium rectale	4.0785	5.2992	1.4783	2.4358	0.2610	0.0983	0.3108	none
Bacteroides vulgatus	4.7083	3.1432	1.0069	0.7843	-0.2590	0.1010	0.3111	none
Bacteroides faecis	0.0096	0.0031	0.0000	0.0000	-0.2550	0.1068	0.3123	none
Streptococcus anginosus	0.0120	0.0330	0.0000	0.0000	-0.2550	0.1068	0.3123	none
Barnesiella intestinihominis	0.4808	0.4755	0.0000	0.0000	-0.2530	0.1097	0.3131	none
Bacteroides ovatus	0.9830	1.3326	0.3730	0.2086	-0.2390	0.1322	0.3528	none
Burkholderiales bacterium 1 1 47	0.2182	0.1484	0.1103	0.0669	-0.2390	0.1322	0.3528	none
Bacteroides nordii	0.0299	0.0230	0.0020	0.0001	-0.2360	0.1357	0.3528	none
Lactobacillus salivarius	0.0263	0.0056	0.0000	0.0000	-0.2360	0.1357	0.3528	none
Bifidobacterium pseudocatenulatum	0.0854	0.1983	0.0002	0.0132	0.2340	0.1393	0.3542	none

Dorea formicigenerans	0.0564	0.0929	0.0312	0.0622	0.2280	0.1504	0.3682	none
Collinsella aerofaciens	0.0508	0.1150	0.0344	0.0686	0.2250	0.1542	0.3682	none
Lachnospiraceae bacterium 5 1 63FA	0.0407	0.0754	0.0092	0.0177	0.2250	0.1542	0.3682	none
Oxalobacter formigenes	0.0090	0.0398	0.0000	0.0000	0.2230	0.1581	0.3701	none
Clostridiales bacterium 1 7 47FAA	0.0032	0.0109	0.0000	0.0000	0.2210	0.1621	0.3720	none
Lachnospiraceae bacterium 8 1 57FA	0.0292	0.0360	0.0000	0.0000	-0.2060	0.1923	0.4327	none
Acidaminococcus intestini	0.0094	0.0095	0.0000	0.0000	-0.2040	0.1969	0.4347	none
Ruminococcus gnavus	0.4623	0.5714	0.0122	0.0322	0.1990	0.2064	0.4472	none
Lachnospiraceae bacterium 7 1 58FA	0.0162	0.0139	0.0007	0.0029	0.1980	0.2113	0.4495	none
Prevotella copri	17.2434	15.8452	0.0576	0.0082	-0.1960	0.2162	0.4518	none
Streptococcus parasanguinis	0.0111	0.0427	0.0000	0.0000	-0.1930	0.2213	0.4542	none
Megamonas rupellensis	1.0969	0.6075	0.0000	0.0000	-0.1870	0.2369	0.4778	none
Alistipes putredinis	3.3408	2.4761	0.8144	0.4546	-0.1800	0.2532	0.5022	none
Dorea longicatena	0.1407	0.3046	0.0822	0.1209	0.1710	0.2822	0.5504	none
Eubacterium hallii	0.1443	0.2209	0.0963	0.1290	0.1680	0.2883	0.5530	none
Roseburia inulinivorans	1.1594	1.2491	0.2303	0.8148	0.1610	0.3070	0.5701	none
Streptococcus salivarius	0.0266	0.1924	0.0044	0.0040	0.1610	0.3070	0.5701	none
Enterobacter cloacae	0.0961	0.1831	0.0000	0.0000	0.1530	0.3332	0.6086	none
Clostridium citroniae	0.0137	0.0154	0.0033	0.0005	-0.1470	0.3537	0.6086	none
Clostridium hathewayi	0.0143	0.0594	0.0000	0.0001	0.1470	0.3537	0.6086	none
Eubacterium eligens	1.1555	0.9412	0.1382	0.1546	0.1470	0.3537	0.6086	none
Ruminococcus lactaris	0.2254	0.2884	0.0000	0.0000	-0.1470	0.3537	0.6086	none
Clostridium bolteae	0.2626	0.1272	0.0132	0.0127	-0.1450	0.3607	0.6116	none
Dialister invisus	1.1785	0.8252	0.0000	0.0000	0.1360	0.3897	0.6421	none
Flavonifractor plautii	0.0334	0.0349	0.0044	0.0057	0.1360	0.3897	0.6421	none
Ruminococcus torques	0.3562	0.6184	0.1563	0.2015	0.1340	0.3971	0.6453	none
Alistipes onderdonkii	0.4127	0.4770	0.0391	0.0501	-0.1210	0.4436	0.6920	none
Lachnospiraceae bacterium 2 1 58FA	0.0046	0.0083	0.0000	0.0000	0.1210	0.4436	0.6920	none
Rothia mucilaginosa	0.0065	0.0384	0.0000	0.0001	0.1210	0.4436	0.6920	none
Citrobacter freundii	0.0086	0.0686	0.0000	0.0000	0.1190	0.4516	0.6953	none
Bifidobacterium bifidum	0.0180	0.0406	0.0000	0.0000	-0.1130	0.4762	0.7236	none
Bilophila wadsworthia	0.0259	0.0270	0.0014	0.0038	0.1060	0.5015	0.7313	none
Clostridium asparagiforme	0.0099	0.0147	0.0004	0.0000	-0.1040	0.5101	0.7313	none
Clostridium symbiosum	0.0051	0.0389	0.0000	0.0000	0.1040	0.5101	0.7313	none
Pyramidobacter piscolens	0.0076	0.0052	0.0000	0.0000	0.1040	0.5101	0.7313	none
Bacteroidales bacterium ph8	0.2443	0.2175	0.0080	0.0067	-0.1020	0.5188	0.7313	none
Solobacterium moorei	0.0001	0.0006	0.0000	0.0000	0.1020	0.5188	0.7313	none
Roseburia intestinalis	0.6194	0.6079	0.0395	0.0741	0.0999	0.5276	0.7348	none

Eubacterium ramulus	0.0266	0.0410	0.0080	0.0138	0.0977	0.5364	0.7383	none
Ruminococcus sp 5 1 39BFAA	0.3508	0.5258	0.1281	0.1933	0.0957	0.5453	0.7418	none
Lachnospiraceae bacterium 3 1 46FA	0.0375	0.0721	0.0052	0.0084	0.0871	0.5816	0.7787	none
Adlercreutzia equolifaciens	0.0162	0.0277	0.0074	0.0041	-0.0829	0.6001	0.7787	none
Clostridium leptum	0.0076	0.0294	0.0000	0.0000	-0.0829	0.6001	0.7787	none
Ruminococcus callidus	0.0595	0.0787	0.0072	0.0163	0.0829	0.6001	0.7787	none
Lachnospiraceae bacterium 1 4 56FA	0.0129	0.0251	0.0008	0.0023	0.0808	0.6095	0.7787	none
candidate division TM7 single cell isol	0.0012	0.0027	0.0000	0.0000	0.0786	0.6190	0.7787	none
Streptococcus australis	0.0023	0.0044	0.0000	0.0001	0.0786	0.6190	0.7787	none
Escherichia coli	2.2641	2.9195	0.3078	0.3521	-0.0743	0.6380	0.7942	none
Megamonas funiformis	0.4002	1.1962	0.0000	0.0000	0.0617	0.6967	0.8580	none
Streptococcus sanguinis	0.0005	0.0025	0.0000	0.0000	0.0574	0.7167	0.8676	none
Megasphaera elsdenii	0.2445	0.4697	0.0000	0.0000	0.0552	0.7267	0.8676	none
Roseburia hominis	0.3981	1.1487	0.1159	0.0892	0.0552	0.7267	0.8676	none
Enterococcus faecium	0.0117	0.0289	0.0000	0.0000	0.0531	0.7368	0.8708	none
Coprococcus catus	0.0483	0.0567	0.0273	0.0336	0.0511	0.7470	0.8740	none
Holdemania filiformis	0.0142	0.0237	0.0014	0.0055	0.0468	0.7675	0.8866	none
Eubacterium ventriosum	0.1837	0.1444	0.0221	0.0213	-0.0446	0.7777	0.8866	none
Bacteroides fragilis	2.1314	0.3925	0.0089	0.0107	-0.0425	0.7881	0.8866	none
Clostridium ramosum	0.0011	0.0068	0.0000	0.0000	-0.0425	0.7881	0.8866	none
Lactobacillus mucosae	0.0015	0.0054	0.0000	0.0000	-0.0362	0.8193	0.9129	none
Veillonella dispar	0.0014	0.0054	0.0000	0.0000	0.0340	0.8297	0.9158	none
Clostridium perfringens	0.0030	0.0132	0.0000	0.0000	0.0297	0.8507	0.9216	none
Klebsiella pneumoniae	0.4949	1.0835	0.0189	0.0309	0.0297	0.8507	0.9216	none
Streptococcus mitis oralis pneumoniae	0.0016	0.0046	0.0000	0.0000	-0.0255	0.8719	0.9359	none
Streptococcus vestibularis	0.0049	0.0158	0.0000	0.0000	-0.0234	0.8825	0.9386	none
Anaerostipes hadrus	0.0168	0.0479	0.0022	0.0050	0.0191	0.9037	0.9441	none
Clostridium nexile	0.0167	0.0556	0.0000	0.0000	-0.0191	0.9037	0.9441	none
Megamonas hypermegale	0.5987	0.9146	0.0000	0.0000	0.0106	0.9464	0.9738	none
Alistipes indistinctus	0.3030	0.2105	0.0000	0.0000	0.0085	0.9571	0.9738	none
Veillonella atypica	0.0148	0.0236	0.0000	0.0000	-0.0085	0.9571	0.9738	none
Akkermansia muciniphila	0.1262	0.3447	0.0000	0.0000	-0.0043	0.9786	0.9786	none
Streptococcus thermophilus	0.0086	0.0349	0.0000	0.0000	-0.0043	0.9786	0.9786	none

EMS Table 4c: Comparison of pathway relative abundances between baseline and 6-month in the vildagliptin group

Pathway	Relative abundance (mean)		Relative abundance (median)		Baseline vs. M6 (Wilcoxon signed-rank test, clr-transformed)			
	Baseline	6-month	Baseline	6-month	Effect_size	P-value	BH adjusted P-value	Enrichment
PYRIDOXSYN-PWY: pyridoxal 5'-phosphate biosynthesis I	0.0059	0.0037	0.0052	0.0030	0.5300	0.0008	0.1402	none
ARGININE-SYN4-PWY: L-ornithine de novo biosynthesis	0.0061	0.0037	0.0052	0.0023	0.5140	0.0011	0.1402	none
PWY0-845: superpathway of pyridoxal 5'-phosphate biosynthesis and salvage	0.0059	0.0037	0.0057	0.0030	0.5010	0.0015	0.1402	none
PWY-6703: preQ0 biosynthesis	0.0116	0.0087	0.0119	0.0079	0.4820	0.0023	0.1402	none
PWY-7282: 4-amino-2-methyl-5-phosphomethylpyrimidine biosynthesis (yeast)	0.0064	0.0045	0.0059	0.0046	0.4680	0.0031	0.1402	none
P23-PWY: reductive TCA cycle I	0.0000	0.0001	0.0000	0.0000	-0.4650	0.0032	0.1402	none
PWY-7209: superpathway of pyrimidine ribonucleosides degradation	0.0000	0.0001	0.0000	0.0000	-0.4650	0.0032	0.1402	none
1CMET2-PWY: N10-formyl-tetrahydrofolate biosynthesis	0.0110	0.0086	0.0112	0.0082	0.4630	0.0034	0.1402	none
FERMENTATION-PWY: mixed acid fermentation	0.0004	0.0008	0.0002	0.0006	-0.4510	0.0044	0.1610	none
PWY-3841: folate transformations II	0.0112	0.0090	0.0116	0.0086	0.4380	0.0056	0.1684	none
PWY-6168: flavin biosynthesis III (fungi)	0.0061	0.0053	0.0063	0.0052	0.4380	0.0056	0.1684	none
PANTO-PWY: phosphopantothenate biosynthesis I	0.0151	0.0128	0.0154	0.0132	0.4330	0.0061	0.1684	none
PWY-5695: urate biosynthesis/inosine 5'-phosphate degradation	0.0146	0.0125	0.0146	0.0116	0.4250	0.0072	0.1813	none
PWY-6700: queuosine biosynthesis	0.0215	0.0172	0.0199	0.0153	0.4190	0.0081	0.1813	none
PWY-2942: L-lysine biosynthesis III	0.0206	0.0166	0.0195	0.0151	0.4160	0.0084	0.1813	none
PWY-5097: L-lysine biosynthesis VI	0.0211	0.0171	0.0192	0.0156	0.4100	0.0095	0.1813	none
PPGPPMET-PWY: ppGpp biosynthesis	0.0003	0.0006	0.0002	0.0003	-0.4080	0.0099	0.1813	none
PEPTIDOGLYCANSYN-PWY: peptidoglycan biosynthesis I (meso-diaminopimelate containing)	0.0181	0.0157	0.0179	0.0160	0.4080	0.0099	0.1813	none
PWY-6386: UDP-N-acetylmuramoyl-pentapeptide biosynthesis II (lysine-containing)	0.0181	0.0158	0.0182	0.0164	0.4030	0.0107	0.1856	none
PWY-7221: guanosine ribonucleotides de novo biosynthesis	0.0200	0.0166	0.0191	0.0152	0.3970	0.0120	0.1957	none
PWY-6387: UDP-N-acetylmuramoyl-pentapeptide biosynthesis I (meso-diaminopimelate containing)	0.0175	0.0153	0.0175	0.0157	0.3950	0.0124	0.1957	none
PWY-7219: adenosine ribonucleotides de novo biosynthesis	0.0272	0.0232	0.0255	0.0226	0.3910	0.0134	0.1977	none
PWY-5686: UMP biosynthesis	0.0203	0.0174	0.0188	0.0163	0.3840	0.0150	0.1977	none
PWY-6123: inosine-5'-phosphate biosynthesis I	0.0062	0.0057	0.0058	0.0062	0.3840	0.0150	0.1977	none
PWY-6385: peptidoglycan biosynthesis III (mycobacteria)	0.0122	0.0106	0.0129	0.0106	0.3840	0.0150	0.1977	none
RIBOSYN2-PWY: flavin biosynthesis I (bacteria and plants)	0.0025	0.0023	0.0023	0.0014	0.3830	0.0155	0.1977	none
PWY-5101: L-isoleucine biosynthesis II	0.0022	0.0019	0.0021	0.0009	0.3810	0.0161	0.1977	none
NONMEVIPP-PWY: methylerythritol phosphate pathway I	0.0161	0.0141	0.0164	0.0136	0.3780	0.0167	0.1978	none
GOLPDLCAT-PWY: superpathway of glycerol degradation to 1,3-propanediol	0.0008	0.0014	0.0001	0.0001	-0.3700	0.0193	0.2208	none
ANAGLYCOLYSIS-PWY: glycolysis III (from glucose)	0.0092	0.0083	0.0091	0.0080	0.3650	0.0208	0.2293	none
PWY-5913: TCA cycle VI (obligate autotrophs)	0.0003	0.0006	0.0001	0.0002	-0.3620	0.0223	0.2308	none
PWY-7039: phosphatidate metabolism, as a signaling molecule	0.0000	0.0001	0.0000	0.0000	-0.3620	0.0223	0.2308	none

PWY-5154: L-arginine biosynthesis III (via N-acetyl-L-citrulline)	0.0018	0.0018	0.0019	0.0016	0.3530	0.0257	0.2574	none
PWY-6936: seleno-amino acid biosynthesis	0.0009	0.0013	0.0005	0.0010	-0.3510	0.0266	0.2586	none
PWY-6531: mannitol cycle	0.0004	0.0003	0.0003	0.0002	0.3480	0.0275	0.2601	none
COA-PWY-1: coenzyme A biosynthesis II (mammalian)	0.0144	0.0133	0.0155	0.0131	0.3400	0.0315	0.2708	none
ILEUSYN-PWY: L-isoleucine biosynthesis I (from threonine)	0.0157	0.0144	0.0162	0.0141	0.3340	0.0348	0.2708	none
PWY0-162: superpathway of pyrimidine ribonucleotides de novo biosynthesis	0.0066	0.0062	0.0061	0.0057	0.3340	0.0348	0.2708	none
VALSYN-PWY: L-valine biosynthesis	0.0157	0.0144	0.0162	0.0141	0.3340	0.0348	0.2708	none
PWY-6285: superpathway of fatty acids biosynthesis (E. coli)	0.0004	0.0008	0.0002	0.0005	-0.3340	0.0348	0.2708	none
PWY-7237: myo-, chiro- and scillo-inositol degradation	0.0022	0.0033	0.0004	0.0005	-0.3340	0.0348	0.2708	none
ANAEROFRUCAT-PWY: homolactic fermentation	0.0037	0.0035	0.0034	0.0034	0.3320	0.0360	0.2708	none
PWY-7111: pyruvate fermentation to isobutanol (engineered)	0.0158	0.0146	0.0163	0.0143	0.3320	0.0360	0.2708	none
PWY-7234: inosine-5'-phosphate biosynthesis III	0.0021	0.0020	0.0020	0.0019	0.3320	0.0360	0.2708	none
PWY0-1319: CDP-diacylglycerol biosynthesis II	0.0148	0.0138	0.0157	0.0137	0.3270	0.0385	0.2708	none
PWY-5667: CDP-diacylglycerol biosynthesis I	0.0148	0.0138	0.0157	0.0137	0.3270	0.0385	0.2708	none
SO4ASSIM-PWY: sulfate reduction I (assimilatory)	0.0017	0.0014	0.0010	0.0009	0.3270	0.0385	0.2708	none
PWY-1269: CMP-3-deoxy-D-manno-octulosonate biosynthesis I	0.0040	0.0040	0.0036	0.0035	0.3190	0.0438	0.3019	none
PWY-7210: pyrimidine deoxyribonucleotides biosynthesis from CTP	0.0010	0.0009	0.0005	0.0005	0.3160	0.0452	0.3054	none
PWY-5659: GDP-mannose biosynthesis	0.0035	0.0034	0.0036	0.0033	0.3150	0.0467	0.3089	none
PWY0-1479: tRNA processing	0.0005	0.0004	0.0005	0.0002	0.3130	0.0482	0.3126	none
PWY0-1261: anhydromuropeptides recycling	0.0012	0.0016	0.0008	0.0012	-0.3080	0.0513	0.3128	none
PWY-7198: pyrimidine deoxyribonucleotides de novo biosynthesis IV	0.0008	0.0007	0.0004	0.0004	0.3080	0.0513	0.3128	none
PWY-7328: superpathway of UDP-glucose-derived O-antigen building blocks biosynthesis	0.0003	0.0003	0.0003	0.0002	0.3080	0.0513	0.3128	none
PWY-6113: superpathway of mycolate biosynthesis	0.0006	0.0009	0.0003	0.0007	-0.3070	0.0529	0.3128	none
PWY-7663: gondoate biosynthesis (anaerobic)	0.0100	0.0094	0.0098	0.0092	0.3070	0.0529	0.3128	none
P42-PWY: incomplete reductive TCA cycle	0.0011	0.0009	0.0009	0.0009	0.2970	0.0599	0.3476	none
PWY-6124: inosine-5'-phosphate biosynthesis II	0.0051	0.0051	0.0049	0.0054	0.2960	0.0617	0.3522	none
RHAMCAT-PWY: L-rhamnose degradation I	0.0034	0.0034	0.0033	0.0035	0.2930	0.0636	0.3569	none
PYRIDNUCSYN-PWY: NAD biosynthesis I (from aspartate)	0.0043	0.0043	0.0038	0.0036	0.2910	0.0656	0.3606	none
ASPASN-PWY: superpathway of L-aspartate and L-asparagine biosynthesis	0.0054	0.0054	0.0058	0.0055	0.2890	0.0675	0.3606	none
PHOSLIPSYN-PWY: superpathway of phospholipid biosynthesis I (bacteria)	0.0036	0.0036	0.0034	0.0033	0.2890	0.0675	0.3606	none
PWY-6151: S-adenosyl-L-methionine cycle I	0.0188	0.0161	0.0178	0.0137	0.2830	0.0738	0.3818	none
PWY-7446: sulfoglycolysis	0.0002	0.0002	0.0000	0.0000	0.2830	0.0738	0.3818	none
PWY0-781: aspartate superpathway	0.0011	0.0016	0.0010	0.0015	-0.2800	0.0760	0.3865	none
PWY-7199: pyrimidine deoxyribonucleosides salvage	0.0026	0.0028	0.0022	0.0029	0.2780	0.0783	0.3865	none
P4-PWY: superpathway of L-lysine, L-threonine and L-methionine biosynthesis I	0.0012	0.0017	0.0011	0.0016	-0.2770	0.0806	0.3865	none
PWY-5863: superpathway of phylloquinol biosynthesis	0.0005	0.0006	0.0003	0.0003	-0.2770	0.0806	0.3865	none
PWY-7229: superpathway of adenosine nucleotides de novo biosynthesis I	0.0099	0.0092	0.0099	0.0083	0.2770	0.0806	0.3865	none
PWY-6284: superpathway of unsaturated fatty acids biosynthesis (E. coli)	0.0005	0.0007	0.0003	0.0006	-0.2740	0.0829	0.3921	none

PWY-7211: superpathway of pyrimidine deoxyribonucleotides de novo biosynthesis	0.0029	0.0028	0.0028	0.0025	0.2640	0.0956	0.4189	none
PWY-7279: aerobic respiration II (cytochrome c) (yeast)	0.0000	0.0000	0.0000	0.0000	0.2640	0.0956	0.4189	none
PWY-7371: 1,4-dihydroxy-6-naphthoate biosynthesis II	0.0009	0.0011	0.0001	0.0000	0.2640	0.0956	0.4189	none
PWY-6609: adenine and adenosine salvage III	0.0065	0.0066	0.0060	0.0060	0.2610	0.0983	0.4189	none
PWY-7013: L-1,2-propanediol degradation	0.0004	0.0007	0.0001	0.0001	-0.2610	0.0983	0.4189	none
PWY-1042: glycolysis IV (plant cytosol)	0.0121	0.0128	0.0127	0.0122	0.2550	0.1068	0.4189	none
PWY-5484: glycolysis II (from fructose 6-phosphate)	0.0028	0.0025	0.0024	0.0019	0.2550	0.1068	0.4189	none
PWY-5083: NAD/NADH phosphorylation and dephosphorylation	0.0007	0.0009	0.0003	0.0004	-0.2530	0.1097	0.4189	none
PWY-5973: cis-vaccenate biosynthesis	0.0102	0.0100	0.0103	0.0101	0.2530	0.1097	0.4189	none
PWY-6606: guanosine nucleotides degradation II	0.0004	0.0004	0.0003	0.0003	0.2530	0.1097	0.4189	none
REDCITCYC: TCA cycle VIII (helicobacter)	0.0004	0.0005	0.0002	0.0003	-0.2530	0.1097	0.4189	none
CITRULBIO-PWY: L-citrulline biosynthesis	0.0012	0.0009	0.0008	0.0005	0.2510	0.1127	0.4189	none
PANTOSYN-PWY: pantothenate and coenzyme A biosynthesis I	0.0056	0.0058	0.0053	0.0057	0.2510	0.1127	0.4189	none
PWY-5791: 1,4-dihydroxy-2-naphthoate biosynthesis II (plants)	0.0005	0.0007	0.0003	0.0003	-0.2510	0.1127	0.4189	none
PWY-5837: 1,4-dihydroxy-2-naphthoate biosynthesis I	0.0005	0.0007	0.0003	0.0003	-0.2510	0.1127	0.4189	none
PWY-7228: superpathway of guanosine nucleotides de novo biosynthesis I	0.0077	0.0071	0.0076	0.0062	0.2510	0.1127	0.4189	none
PWY-724: superpathway of L-lysine, L-threonine and L-methionine biosynthesis II	0.0096	0.0096	0.0099	0.0100	0.2510	0.1127	0.4189	none
DTDPRHAMSYN-PWY: dTDP-L-rhamnose biosynthesis I	0.0068	0.0067	0.0064	0.0067	0.2480	0.1158	0.4189	none
FAO-PWY: fatty acid β-oxidation I	0.0003	0.0007	0.0002	0.0004	-0.2480	0.1158	0.4189	none
PWY-6595: superpathway of guanosine nucleotides degradation (plants)	0.0002	0.0001	0.0001	0.0001	0.2480	0.1158	0.4189	none
P122-PWY: heterolactic fermentation	0.0002	0.0004	0.0001	0.0002	-0.2470	0.1190	0.4189	none
PWY-4984: urea cycle	0.0010	0.0008	0.0007	0.0004	0.2470	0.1190	0.4189	none
PWY-6125: superpathway of guanosine nucleotides de novo biosynthesis II	0.0065	0.0060	0.0062	0.0053	0.2470	0.1190	0.4189	none
PWY-6126: superpathway of adenosine nucleotides de novo biosynthesis II	0.0082	0.0076	0.0080	0.0068	0.2470	0.1190	0.4189	none
THRESYN-PWY: superpathway of L-threonine biosynthesis	0.0062	0.0064	0.0065	0.0066	0.2450	0.1222	0.4256	none
PWY0-166: superpathway of pyrimidine deoxyribonucleotides de novo biosynthesis (E. coli)	0.0051	0.0049	0.0050	0.0046	0.2420	0.1254	0.4325	none
PWY-3781: aerobic respiration I (cytochrome c)	0.0000	0.0000	0.0000	0.0000	0.2360	0.1357	0.4631	none
TRNA-CHARGING-PWY: tRNA charging	0.0018	0.0016	0.0017	0.0015	0.2340	0.1393	0.4704	none
LACTOSECAT-PWY: lactose and galactose degradation I	0.0001	0.0002	0.0001	0.0001	-0.2320	0.1429	0.4730	none
PWY-7204: pyridoxal 5'-phosphate salvage II (plants)	0.0008	0.0007	0.0003	0.0003	0.2320	0.1429	0.4730	none
GLYCOLYSIS: glycolysis I (from glucose 6-phosphate)	0.0029	0.0026	0.0024	0.0020	0.2290	0.1466	0.4757	none
PWY0-1241: ADP-L-glycero-β-D-manno-heptose biosynthesis	0.0003	0.0005	0.0001	0.0002	-0.2290	0.1466	0.4757	none
CATECHOL-ORTHO-CLEAVAGE-PWY: catechol degradation to β-ketoadipate	0.0000	0.0000	0.0000	0.0000	-0.2280	0.1504	0.4786	none
DENOVOPURINE2-PWY: superpathway of purine nucleotides de novo biosynthesis II	0.0017	0.0015	0.0017	0.0011	0.2280	0.1504	0.4786	none
PWY-7539: 6-hydroxymethyl-dihydropterin diphosphate biosynthesis III (Chlamydia)	0.0011	0.0011	0.0007	0.0007	0.2250	0.1542	0.4862	none
PWY0-1061: superpathway of L-alanine biosynthesis	0.0007	0.0011	0.0004	0.0005	-0.2180	0.1662	0.5094	none
PWY-7220: adenosine deoxyribonucleotides de novo biosynthesis II	0.0047	0.0044	0.0042	0.0037	0.2180	0.1662	0.5094	none

PWY-7222: guanosine deoxyribonucleotides de novo biosynthesis II	0.0047	0.0044	0.0042	0.0037	0.2180	0.1662	0.5094	none
PWY-5173: superpathway of acetyl-CoA biosynthesis	0.0002	0.0003	0.0001	0.0002	-0.2170	0.1704	0.5127	none
PWY-7184: pyrimidine deoxyribonucleotides de novo biosynthesis I	0.0044	0.0042	0.0043	0.0040	0.2170	0.1704	0.5127	none
3-HYDROXYPHENYLACETATE-DEGRADATION-PWY: 4-hydroxyphenylacetate degradation	0.0002	0.0002	0.0000	0.0000	-0.2120	0.1789	0.5241	none
ARGORNPROST-PWY: arginine, ornithine and proline interconversion	0.0001	0.0001	0.0000	0.0000	0.2120	0.1789	0.5241	none
COLANSYN-PWY: colanic acid building blocks biosynthesis	0.0015	0.0014	0.0012	0.0013	0.2120	0.1789	0.5241	none
TRPSYN-PWY: L-tryptophan biosynthesis	0.0047	0.0048	0.0044	0.0044	0.2100	0.1833	0.5260	none
CENTFERM-PWY: pyruvate fermentation to butanoate	0.0001	0.0001	0.0001	0.0000	0.2090	0.1878	0.5260	none
PRPP-PWY: superpathway of histidine, purine, and pyrimidine biosynthesis	0.0013	0.0012	0.0013	0.0009	0.2090	0.1878	0.5260	none
PWY-5136: fatty acid β-oxidation II (peroxisome)	0.0003	0.0006	0.0002	0.0003	-0.2090	0.1878	0.5260	none
PWY-5971: palmitate biosynthesis II (bacteria and plants)	0.0008	0.0011	0.0005	0.0007	-0.2090	0.1878	0.5260	none
P161-PWY: acetylene degradation	0.0004	0.0007	0.0003	0.0005	-0.2060	0.1923	0.5260	none
P461-PWY: hexitol fermentation to lactate, formate, ethanol and acetate	0.0021	0.0028	0.0009	0.0016	-0.2060	0.1923	0.5260	none
PWY-6897: thiamin salvage II	0.0089	0.0092	0.0088	0.0088	0.2060	0.1923	0.5260	none
AEROBACTINSYN-PWY: aerobactin biosynthesis	0.0000	0.0000	0.0000	0.0000	0.2040	0.1969	0.5343	none
PWY-4242: pantothenate and coenzyme A biosynthesis III	0.0022	0.0025	0.0019	0.0024	0.2020	0.2016	0.5426	none
PWY-7456: mannan degradation	0.0004	0.0003	0.0000	0.0000	0.1990	0.2064	0.5507	none
P164-PWY: purine nucleobases degradation I (anaerobic)	0.0003	0.0004	0.0002	0.0004	-0.1980	0.2113	0.5507	none
PWY-6590: superpathway of Clostridium acetobutylicum acidogenic fermentation	0.0002	0.0001	0.0001	0.0001	0.1980	0.2113	0.5507	none
PWY-7323: superpathway of GDP-mannose-derived O-antigen building blocks biosynthesis	0.0015	0.0015	0.0012	0.0011	0.1980	0.2113	0.5507	none
PWY-6895: superpathway of thiamin diphosphate biosynthesis II	0.0015	0.0017	0.0007	0.0010	0.1930	0.2213	0.5722	none
PWY-6163: chorismate biosynthesis from 3-dehydroquinate	0.0111	0.0116	0.0110	0.0115	0.1910	0.2264	0.5809	none
PWY-1861: formaldehyde assimilation II (RuMP Cycle)	0.0000	0.0000	0.0000	0.0000	-0.1900	0.2316	0.5897	none
COMPLETE-ARO-PWY: superpathway of aromatic amino acid biosynthesis	0.0112	0.0118	0.0110	0.0122	0.1870	0.2369	0.5985	none
MET-SAM-PWY: superpathway of S-adenosyl-L-methionine biosynthesis	0.0020	0.0026	0.0018	0.0024	-0.1830	0.2477	0.6118	none
PWY-6467: Kdo transfer to lipid IVA III (Chlamydia)	0.0000	0.0000	0.0000	0.0000	0.1830	0.2477	0.6118	none
PWYG-321: mycolate biosynthesis	0.0008	0.0011	0.0004	0.0007	-0.1830	0.2477	0.6118	none
HCAMHPDEG-PWY: 3-phenylpropanoate and 3-(3-hydroxyphenyl)propanoate degradation to 2-oxopent-4-enoate	0.0002	0.0004	0.0000	0.0001	-0.1800	0.2532	0.6118	none
PWY-5415: catechol degradation I (meta-cleavage pathway)	0.0000	0.0001	0.0000	0.0000	-0.1800	0.2532	0.6118	none
PWY-6690: cinnamate and 3-hydroxycinnamate degradation to 2-oxopent-4-enoate	0.0002	0.0004	0.0000	0.0001	-0.1800	0.2532	0.6118	none
HOMOSER-METSYN-PWY: L-methionine biosynthesis I	0.0013	0.0016	0.0011	0.0015	-0.1790	0.2589	0.6209	none
PWY0-41: allantoin degradation IV (anaerobic)	0.0000	0.0000	0.0000	0.0000	0.1770	0.2646	0.6255	none
PWY-5367: petroselinate biosynthesis	0.0010	0.0014	0.0005	0.0010	-0.1770	0.2646	0.6255	none
CALVIN-PWY: Calvin-Benson-Bassham cycle	0.0087	0.0094	0.0085	0.0090	0.1720	0.2763	0.6440	none
PWY-621: sucrose degradation III (sucrose invertase)	0.0037	0.0048	0.0030	0.0045	-0.1720	0.2763	0.6440	none

METSYN-PWY: L-homoserine and L-methionine biosynthesis	0.0021	0.0026	0.0019	0.0025	-0.1710	0.2822	0.6488	none
PWY-561: superpathway of glyoxylate cycle and fatty acid degradation	0.0003	0.0006	0.0001	0.0002	-0.1710	0.2822	0.6488	none
ARO-PWY: chorismate biosynthesis I	0.0108	0.0117	0.0107	0.0117	0.1660	0.2944	0.6585	none
GLYCOCAT-PWY: glycogen degradation I (bacterial)	0.0007	0.0008	0.0005	0.0005	0.1660	0.2944	0.6585	none
PWY-6859: all-trans-farnesol biosynthesis	0.0002	0.0002	0.0001	0.0001	0.1660	0.2944	0.6585	none
PWY-7187: pyrimidine deoxyribonucleotides de novo biosynthesis II	0.0015	0.0014	0.0014	0.0010	0.1660	0.2944	0.6585	none
FUCCAT-PWY: fucose degradation	0.0004	0.0006	0.0002	0.0002	-0.1630	0.3007	0.6598	none
PWY-5177: glutaryl-CoA degradation	0.0019	0.0026	0.0014	0.0024	-0.1630	0.3007	0.6598	none
ARG+POLYAMINE-SYN: superpathway of arginine and polyamine biosynthesis	0.0015	0.0021	0.0010	0.0023	-0.1610	0.3070	0.6598	none
PWY-6121: 5-aminoimidazole ribonucleotide biosynthesis I	0.0089	0.0094	0.0091	0.0096	0.1610	0.3070	0.6598	none
THISYNARA-PWY: superpathway of thiamin diphosphate biosynthesis III (eukaryotes)	0.0045	0.0047	0.0046	0.0046	0.1610	0.3070	0.6598	none
THISYN-PWY: superpathway of thiamin diphosphate biosynthesis I	0.0019	0.0020	0.0011	0.0013	0.1610	0.3070	0.6598	none
PWY-6731: starch degradation III	0.0000	0.0000	0.0000	0.0000	-0.1600	0.3134	0.6650	none
PWY-7196: superpathway of pyrimidine ribonucleosides salvage	0.0023	0.0027	0.0023	0.0018	0.1600	0.3134	0.6650	none
COA-PWY: coenzyme A biosynthesis I	0.0034	0.0039	0.0031	0.0037	0.1570	0.3199	0.6683	none
PWY0-1296: purine ribonucleosides degradation	0.0064	0.0082	0.0059	0.0077	-0.1550	0.3265	0.6683	none
PWY-5989: stearate biosynthesis II (bacteria and plants)	0.0016	0.0020	0.0013	0.0018	-0.1550	0.3265	0.6683	none
METHGLYUT-PWY: superpathway of methylglyoxal degradation	0.0001	0.0002	0.0000	0.0000	0.1530	0.3332	0.6683	none
PWY0-1277: 3-phenylpropanoate and 3-(3-hydroxyphenyl)propanoate degradation	0.0003	0.0005	0.0001	0.0001	-0.1530	0.3332	0.6683	none
PWY-5347: superpathway of L-methionine biosynthesis (transsulfuration)	0.0022	0.0028	0.0020	0.0028	-0.1530	0.3332	0.6683	none
PWY-5897: superpathway of menaquinol-11 biosynthesis	0.0013	0.0016	0.0007	0.0008	-0.1530	0.3332	0.6683	none
PWY-5898: superpathway of menaquinol-12 biosynthesis	0.0013	0.0016	0.0007	0.0008	-0.1530	0.3332	0.6683	none
PWY-5899: superpathway of menaquinol-13 biosynthesis	0.0013	0.0016	0.0007	0.0008	-0.1530	0.3332	0.6683	none
NAGLIPASYN-PWY: lipid IVA biosynthesis	0.0010	0.0010	0.0009	0.0007	0.1510	0.3399	0.6778	none
PWY-7208: superpathway of pyrimidine nucleobases salvage	0.0063	0.0065	0.0062	0.0060	0.1490	0.3468	0.6873	none
PWY66-422: D-galactose degradation V (Leloir pathway)	0.0054	0.0068	0.0052	0.0064	-0.1470	0.3537	0.6942	none
PWY-3001: superpathway of L-isoleucine biosynthesis I	0.0064	0.0071	0.0068	0.0072	0.1450	0.3607	0.6942	none
PWY-5030: L-histidine degradation III	0.0007	0.0006	0.0006	0.0004	0.1450	0.3607	0.6942	none
PWY-6317: galactose degradation I (Leloir pathway)	0.0054	0.0068	0.0052	0.0064	-0.1450	0.3607	0.6942	none
PWY66-409: superpathway of purine nucleotide salvage	0.0016	0.0020	0.0010	0.0013	-0.1450	0.3607	0.6942	none
GALACTARDEG-PWY: D-galactarate degradation I	0.0002	0.0003	0.0000	0.0001	-0.1400	0.3750	0.7053	none
GLUCARGALACTSUPER-PWY: superpathway of D-glucarate and D-galactarate degradation	0.0002	0.0003	0.0000	0.0001	-0.1400	0.3750	0.7053	none
OANTIGEN-PWY: O-antigen building blocks biosynthesis (E. coli)	0.0013	0.0018	0.0012	0.0016	-0.1400	0.3750	0.7053	none
ORNDEG-PWY: superpathway of ornithine degradation	0.0002	0.0003	0.0001	0.0001	-0.1400	0.3750	0.7053	none
HEME-BIOSYNTHESIS-II: heme biosynthesis I (aerobic)	0.0003	0.0004	0.0002	0.0003	0.1380	0.3823	0.7109	none
PWY-5910: superpathway of geranylgeranyldiphosphate biosynthesis I (via mevalonate)	0.0000	0.0000	0.0000	0.0000	-0.1380	0.3823	0.7109	none
COBALSYN-PWY: adenosylcobalamin salvage from cobinamide I	0.0047	0.0057	0.0044	0.0051	-0.1340	0.3971	0.7262	none

FOLSYN-PWY: superpathway of tetrahydrofolate biosynthesis and salvage	0.0006	0.0007	0.0003	0.0003	0.1340	0.3971	0.7262	none
P185-PWY: formaldehyde assimilation III (dihydroxyacetone cycle)	0.0006	0.0004	0.0001	0.0002	-0.1340	0.3971	0.7262	none
PWY-7409: phospholipid remodeling (phosphatidylethanolamine, yeast)	0.0003	0.0003	0.0001	0.0001	0.1320	0.4046	0.7359	none
PWY-6471: peptidoglycan biosynthesis IV (Enterococcus faecium)	0.0000	0.0001	0.0000	0.0000	-0.1270	0.4200	0.7596	none
PWY-4041: γ-glutamyl cycle	0.0004	0.0006	0.0001	0.0002	-0.1250	0.4278	0.7670	none
PWY-5103: L-isoleucine biosynthesis III	0.0063	0.0081	0.0066	0.0077	-0.1230	0.4356	0.7670	none
PWY-6629: superpathway of L-tryptophan biosynthesis	0.0008	0.0011	0.0003	0.0004	0.1230	0.4356	0.7670	none
PWY-6891: thiazole biosynthesis II (Bacillus)	0.0005	0.0006	0.0001	0.0002	0.1230	0.4356	0.7670	none
PWY-922: mevalonate pathway I	0.0000	0.0000	0.0000	0.0000	-0.1230	0.4356	0.7670	none
PWY-5747: 2-methylcitrate cycle II	0.0000	0.0001	0.0000	0.0000	-0.1210	0.4436	0.7713	none
GLUCARDEG-PWY: D-glucarate degradation I	0.0003	0.0004	0.0001	0.0001	-0.1190	0.4516	0.7713	none
NAD-BIOSYNTHESIS-II: NAD salvage pathway II	0.0005	0.0008	0.0002	0.0003	-0.1190	0.4516	0.7713	none
HISDEG-PWY: L-histidine degradation I	0.0004	0.0004	0.0003	0.0002	0.1170	0.4597	0.7713	none
NONOXIPENT-PWY: pentose phosphate pathway (non-oxidative branch)	0.0071	0.0082	0.0070	0.0083	0.1170	0.4597	0.7713	none
PWY-6803: phosphatidylcholine acyl editing	0.0005	0.0007	0.0002	0.0003	-0.1170	0.4597	0.7713	none
PWY-7197: pyrimidine deoxyribonucleotide phosphorylation	0.0036	0.0038	0.0033	0.0033	0.1170	0.4597	0.7713	none
PWY-5384: sucrose degradation IV (sucrose phosphorylase)	0.0003	0.0005	0.0002	0.0003	-0.1150	0.4679	0.7713	none
PWY-5860: superpathway of demethylmenaquinol-6 biosynthesis I	0.0006	0.0008	0.0003	0.0003	-0.1150	0.4679	0.7713	none
PWY-5862: superpathway of demethylmenaquinol-9 biosynthesis	0.0006	0.0008	0.0003	0.0003	-0.1150	0.4679	0.7713	none
PWY-6507: 4-deoxy-L-threo-hex-4-enopyranuronate degradation	0.0029	0.0039	0.0024	0.0040	-0.1150	0.4679	0.7713	none
PWY-6737: starch degradation V	0.0103	0.0129	0.0101	0.0136	-0.1150	0.4679	0.7713	none
PWY0-42: 2-methylcitrate cycle I	0.0000	0.0001	0.0000	0.0000	-0.1130	0.4762	0.7713	none
GLYOXYLATE-BYPASS: glyoxylate cycle	0.0005	0.0007	0.0002	0.0003	-0.1110	0.4846	0.7713	none
PWY0-1297: superpathway of purine deoxyribonucleosides degradation	0.0011	0.0014	0.0007	0.0008	-0.1080	0.4930	0.7713	none
PWY0-1586: peptidoglycan maturation (meso-diaminopimelate containing)	0.0043	0.0050	0.0039	0.0046	0.1080	0.4930	0.7713	none
PWY-5417: catechol degradation III (ortho-cleavage pathway)	0.0000	0.0000	0.0000	0.0000	-0.1080	0.4930	0.7713	none
PWY-5431: aromatic compounds degradation via β-ketoadipate	0.0000	0.0000	0.0000	0.0000	-0.1080	0.4930	0.7713	none
PWY-6588: pyruvate fermentation to acetone	0.0002	0.0002	0.0001	0.0001	0.1080	0.4930	0.7713	none
DAPLYSINESYN-PWY: L-lysine biosynthesis I	0.0018	0.0022	0.0016	0.0020	-0.1060	0.5015	0.7713	none
P108-PWY: pyruvate fermentation to propanoate I	0.0002	0.0002	0.0001	0.0001	-0.1060	0.5015	0.7713	none
P441-PWY: superpathway of N-acetylneuraminate degradation	0.0010	0.0013	0.0007	0.0010	-0.1060	0.5015	0.7713	none
PWY-5845: superpathway of menaquinol-9 biosynthesis	0.0009	0.0010	0.0004	0.0005	-0.1060	0.5015	0.7713	none
PWY-5850: superpathway of menaquinol-6 biosynthesis I	0.0009	0.0010	0.0004	0.0005	-0.1060	0.5015	0.7713	none
PWY-5896: superpathway of menaquinol-10 biosynthesis	0.0009	0.0010	0.0004	0.0005	-0.1060	0.5015	0.7713	none
PWY-6612: superpathway of tetrahydrofolate biosynthesis	0.0004	0.0005	0.0002	0.0002	0.1060	0.5015	0.7713	none
PWY-6282: palmitoleate biosynthesis I (from (5Z)-dodec-5-enoate)	0.0008	0.0010	0.0005	0.0006	-0.1040	0.5101	0.7713	none
FUC-RHAMCAT-PWY: superpathway of fucose and rhamnose degradation	0.0006	0.0008	0.0003	0.0004	-0.1020	0.5188	0.7713	none
KETOGLUCONMET-PWY: ketogluconate metabolism	0.0002	0.0002	0.0001	0.0001	0.0999	0.5276	0.7713	none

PWY0-1533: methylphosphonate degradation I	0.0002	0.0003	0.0000	0.0001	0.0999	0.5276	0.7713	none
PWY-6122: 5-aminoimidazole ribonucleotide biosynthesis II	0.0084	0.0092	0.0081	0.0093	0.0999	0.5276	0.7713	none
PWY-6277: superpathway of 5-aminoimidazole ribonucleotide biosynthesis	0.0084	0.0092	0.0081	0.0093	0.0999	0.5276	0.7713	none
PYRIDNUCSAL-PWY: NAD salvage pathway I	0.0005	0.0007	0.0003	0.0005	-0.0999	0.5276	0.7713	none
FASYN-INITIAL-PWY: superpathway of fatty acid biosynthesis initiation (E. coli)	0.0007	0.0009	0.0004	0.0005	-0.0977	0.5364	0.7713	none
HISTSYN-PWY: L-histidine biosynthesis	0.0023	0.0027	0.0020	0.0028	0.0977	0.5364	0.7713	none
HSERMETANA-PWY: L-methionine biosynthesis III	0.0013	0.0014	0.0011	0.0012	0.0977	0.5364	0.7713	none
PWY-6545: pyrimidine deoxyribonucleotides de novo biosynthesis III	0.0037	0.0038	0.0040	0.0035	0.0977	0.5364	0.7713	none
PWY-7269: NAD/NADP-NADH/NADPH mitochondrial interconversion (yeast)	0.0004	0.0005	0.0001	0.0001	0.0977	0.5364	0.7713	none
PWY-7383: anaerobic energy metabolism (invertebrates, cytosol)	0.0003	0.0004	0.0002	0.0003	-0.0977	0.5364	0.7713	none
PWY-7388: octanoyl-[acyl-carrier protein] biosynthesis (mitochondria, yeast)	0.0006	0.0008	0.0003	0.0005	-0.0977	0.5364	0.7713	none
ENTBACSYN-PWY: enterobactin biosynthesis	0.0006	0.0008	0.0002	0.0003	0.0957	0.5453	0.7713	none
GLUCOSE1PMETAB-PWY: glucose and glucose-1-phosphate degradation	0.0005	0.0007	0.0002	0.0003	-0.0957	0.5453	0.7713	none
PWY-5855: ubiquinol-7 biosynthesis (prokaryotic)	0.0003	0.0004	0.0001	0.0002	-0.0957	0.5453	0.7713	none
PWY-5856: ubiquinol-9 biosynthesis (prokaryotic)	0.0003	0.0004	0.0001	0.0002	-0.0957	0.5453	0.7713	none
PWY-5857: ubiquinol-10 biosynthesis (prokaryotic)	0.0003	0.0004	0.0001	0.0002	-0.0957	0.5453	0.7713	none
PWY-6708: ubiquinol-8 biosynthesis (prokaryotic)	0.0003	0.0004	0.0001	0.0002	-0.0957	0.5453	0.7713	none
PWY-2941: L-lysine biosynthesis II	0.0009	0.0008	0.0004	0.0006	-0.0934	0.5542	0.7773	none
PWY-7357: thiamin formation from pyrithiamine and oxythiamine (yeast)	0.0086	0.0097	0.0078	0.0096	0.0934	0.5542	0.7773	none
PWY-5104: L-isoleucine biosynthesis IV	0.0010	0.0010	0.0006	0.0007	0.0914	0.5633	0.7867	none
GLYCOGENSYNTH-PWY: glycogen biosynthesis I (from ADP-D-Glucose)	0.0023	0.0028	0.0021	0.0028	-0.0893	0.5724	0.7927	none
P124-PWY: Bifidobacterium shunt	0.0000	0.0000	0.0000	0.0000	0.0893	0.5724	0.7927	none
HEXITOLDEGSUPER-PWY: superpathway of hexitol degradation (bacteria)	0.0014	0.0015	0.0012	0.0013	0.0871	0.5816	0.7978	none
PWY-6147: 6-hydroxymethyl-dihydropterin diphosphate biosynthesis I	0.0013	0.0014	0.0007	0.0007	0.0851	0.5908	0.7978	none
PWY-6353: purine nucleotides degradation II (aerobic)	0.0010	0.0013	0.0006	0.0007	-0.0851	0.5908	0.7978	none
PWY-6527: stachyose degradation	0.0046	0.0055	0.0046	0.0055	-0.0851	0.5908	0.7978	none
SER-GLYSYN-PWY: superpathway of L-serine and glycine biosynthesis I	0.0044	0.0052	0.0040	0.0052	0.0851	0.5908	0.7978	none
BRANCHED-CHAIN-AA-SYN-PWY: superpathway of branched amino acid biosynthesis	0.0072	0.0090	0.0079	0.0088	-0.0829	0.6001	0.7978	none
GLYCOLYSIS-E-D: superpathway of glycolysis and Entner-Doudoroff	0.0010	0.0012	0.0007	0.0009	0.0829	0.6001	0.7978	none
PWY-5100: pyruvate fermentation to acetate and lactate II	0.0023	0.0031	0.0021	0.0029	-0.0829	0.6001	0.7978	none
PWY-5505: L-glutamate and L-glutamine biosynthesis	0.0002	0.0004	0.0001	0.0002	-0.0829	0.6001	0.7978	none
PWY-5723: Rubisco shunt	0.0008	0.0009	0.0003	0.0004	-0.0829	0.6001	0.7978	none
PENTOSE-P-PWY: pentose phosphate pathway	0.0013	0.0015	0.0010	0.0013	0.0786	0.6190	0.8029	none
PWY-2723: trehalose degradation V	0.0003	0.0003	0.0001	0.0001	0.0786	0.6190	0.8029	none
PWY-6270: isoprene biosynthesis I	0.0019	0.0020	0.0008	0.0012	0.0786	0.6190	0.8029	none
PWY-7664: oleate biosynthesis IV (anaerobic)	0.0009	0.0011	0.0005	0.0007	-0.0786	0.6190	0.8029	none
ARGSYNBSUB-PWY: L-arginine biosynthesis II (acetyl cycle)	0.0020	0.0025	0.0019	0.0022	-0.0765	0.6285	0.8029	none
GLCMANNANAUT-PWY: superpathway of N-acetylglucosamine, N-acetylmannosamine	0.0041	0.0053	0.0040	0.0050	-0.0743	0.6380	0.8029	none

and N-acetylneuraminate degradation								
PWY-6263: superpathway of menaquinol-8 biosynthesis II	0.0002	0.0002	0.0000	0.0000	0.0743	0.6380	0.8029	none
PWY-6519: 8-amino-7-oxononanoate biosynthesis I	0.0009	0.0010	0.0004	0.0007	-0.0743	0.6380	0.8029	none
PWY-7560: methylerythritol phosphate pathway II	0.0017	0.0018	0.0007	0.0011	0.0743	0.6380	0.8029	none
PWY-841: superpathway of purine nucleotides de novo biosynthesis I	0.0025	0.0030	0.0023	0.0029	0.0743	0.6380	0.8029	none
ARGSYN-PWY: L-arginine biosynthesis I (via L-ornithine)	0.0033	0.0042	0.0035	0.0045	-0.0723	0.6477	0.8029	none
FASYN-ELONG-PWY: fatty acid elongation -- saturated	0.0010	0.0012	0.0006	0.0008	-0.0723	0.6477	0.8029	none
KDO-NAGLIPASYN-PWY: superpathway of (Kdo)2-lipid A biosynthesis	0.0002	0.0003	0.0000	0.0001	0.0723	0.6477	0.8029	none
PWY0-1338: polymyxin resistance	0.0002	0.0002	0.0000	0.0000	0.0723	0.6477	0.8029	none
PWY0-862: (5Z)-dodec-5-enoate biosynthesis	0.0008	0.0010	0.0005	0.0006	-0.0723	0.6477	0.8029	none
PWY-6628: superpathway of L-phenylalanine biosynthesis	0.0011	0.0014	0.0005	0.0009	0.0723	0.6477	0.8029	none
PWY-7400: L-arginine biosynthesis IV (archaebacteria)	0.0036	0.0045	0.0038	0.0049	-0.0723	0.6477	0.8029	none
PWY-821: superpathway of sulfur amino acid biosynthesis (Saccharomyces cerevisiae)	0.0002	0.0003	0.0001	0.0002	-0.0723	0.6477	0.8029	none
GLUCONEO-PWY: gluconeogenesis I	0.0012	0.0012	0.0008	0.0010	0.0702	0.6574	0.8029	none
PWY4FS-7: phosphatidylglycerol biosynthesis I (plastidic)	0.0028	0.0031	0.0022	0.0024	0.0702	0.6574	0.8029	none
PWY4FS-8: phosphatidylglycerol biosynthesis II (non-plastidic)	0.0028	0.0031	0.0022	0.0024	0.0702	0.6574	0.8029	none
PWY-5861: superpathway of demethylmenaquinol-8 biosynthesis	0.0007	0.0009	0.0003	0.0004	-0.0702	0.6574	0.8029	none
PWY0-321: phenylacetate degradation I (aerobic)	0.0001	0.0002	0.0000	0.0000	-0.0680	0.6671	0.8059	none
PWY-5188: tetrapyrrole biosynthesis I (from glutamate)	0.0026	0.0030	0.0020	0.0023	0.0680	0.6671	0.8059	none
RUMP-PWY: formaldehyde oxidation I	0.0000	0.0000	0.0000	0.0000	0.0680	0.6671	0.8059	none
PWY-5656: mannosylglycerate biosynthesis I	0.0001	0.0001	0.0000	0.0000	0.0659	0.6769	0.8089	none
PWY-6892: thiazole biosynthesis I (E. coli)	0.0007	0.0008	0.0002	0.0003	0.0659	0.6769	0.8089	none
PWY-7242: D-fructuronate degradation	0.0032	0.0042	0.0029	0.0046	-0.0659	0.6769	0.8089	none
POLYAMSYN-PWY: superpathway of polyamine biosynthesis I	0.0019	0.0025	0.0012	0.0018	-0.0637	0.6868	0.8148	none
PWY-5705: allantoin degradation to glyoxylate III	0.0002	0.0002	0.0000	0.0000	0.0637	0.6868	0.8148	none
ECASYN-PWY: enterobacterial common antigen biosynthesis	0.0002	0.0003	0.0000	0.0001	0.0617	0.6967	0.8207	none
PWY-6823: molybdenum cofactor biosynthesis	0.0002	0.0003	0.0000	0.0001	0.0617	0.6967	0.8207	none
GALACT-GLUCUROCAT-PWY: superpathway of hexuronide and hexuronate degradation	0.0033	0.0040	0.0030	0.0040	0.0595	0.7067	0.8294	none
PWY-6305: putrescine biosynthesis IV	0.0023	0.0029	0.0017	0.0023	-0.0574	0.7167	0.8323	none
PWY-7003: glycerol degradation to butanol	0.0002	0.0003	0.0001	0.0002	-0.0574	0.7167	0.8323	none
UBISYN-PWY: superpathway of ubiquinol-8 biosynthesis (prokaryotic)	0.0003	0.0004	0.0001	0.0001	-0.0574	0.7167	0.8323	none
ARGDEG-PWY: superpathway of L-arginine, putrescine, and 4-aminobutanoate degradation	0.0002	0.0002	0.0000	0.0000	0.0552	0.7267	0.8381	none
ORNARGDEG-PWY: superpathway of L-arginine and L-ornithine degradation	0.0002	0.0002	0.0000	0.0000	0.0552	0.7267	0.8381	none
PWY-5345: superpathway of L-methionine biosynthesis (by sulfhydrylation)	0.0010	0.0012	0.0006	0.0012	0.0531	0.7368	0.8439	none
PWY-7254: TCA cycle VII (acetate-producers)	0.0004	0.0006	0.0001	0.0002	-0.0531	0.7368	0.8439	none
P105-PWY: TCA cycle IV (2-oxoglutarate decarboxylase)	0.0006	0.0008	0.0003	0.0004	-0.0511	0.7470	0.8497	none

PWY-6969: TCA cycle V (2-oxoglutarate:ferredoxin oxidoreductase)	0.0016	0.0020	0.0016	0.0020	0.0511	0.7470	0.8497	none
GLUTORN-PWY: L-ornithine biosynthesis	0.0014	0.0018	0.0012	0.0014	-0.0489	0.7572	0.8554	none
PWY-5676: acetyl-CoA fermentation to butanoate II	0.0009	0.0012	0.0010	0.0008	0.0489	0.7572	0.8554	none
GLYCOLYSIS-TCA-GLYOX-BYPASS: superpathway of glycolysis, pyruvate dehydrogenase, TCA, and glyoxylate bypass	0.0007	0.0009	0.0004	0.0004	0.0468	0.7675	0.8582	none
PWY-5121: superpathway of geranylgeranyl diphosphate biosynthesis II (via MEP)	0.0008	0.0010	0.0004	0.0006	0.0468	0.7675	0.8582	none
PWY-6901: superpathway of glucose and xylose degradation	0.0014	0.0016	0.0012	0.0013	0.0468	0.7675	0.8582	none
PWY-4981: L-proline biosynthesis II (from arginine)	0.0003	0.0004	0.0002	0.0002	-0.0446	0.7777	0.8639	none
SULFATE-CYS-PWY: superpathway of sulfate assimilation and cysteine biosynthesis	0.0009	0.0012	0.0005	0.0009	-0.0446	0.7777	0.8639	none
BIOTIN-BIOSYNTHESIS-PWY: biotin biosynthesis I	0.0010	0.0011	0.0006	0.0008	-0.0425	0.7881	0.8724	none
PWY0-1298: superpathway of pyrimidine deoxyribonucleosides degradation	0.0005	0.0006	0.0004	0.0005	-0.0403	0.7984	0.8780	none
PWY-5840: superpathway of menaquinol-7 biosynthesis	0.0011	0.0013	0.0007	0.0008	-0.0403	0.7984	0.8780	none
GLUCUROCAT-PWY: superpathway of β-D-glucuronide and D-glucuronate degradation	0.0031	0.0038	0.0028	0.0038	0.0383	0.8088	0.8865	none
PWY-5138: unsaturated, even numbered fatty acid β-oxidation	0.0001	0.0002	0.0000	0.0000	-0.0362	0.8193	0.8888	none
GALACTUROCAT-PWY: D-galacturonate degradation I	0.0034	0.0043	0.0030	0.0045	0.0340	0.8297	0.8888	none
GLYCOL-GLYOXDEG-PWY: superpathway of glycol metabolism and degradation	0.0002	0.0002	0.0001	0.0001	0.0340	0.8297	0.8888	none
POLYAMINSYN3-PWY: superpathway of polyamine biosynthesis II	0.0004	0.0004	0.0001	0.0001	0.0340	0.8297	0.8888	none
PWY4LZ-257: superpathway of fermentation (Chlamydomonas reinhardtii)	0.0003	0.0003	0.0002	0.0002	-0.0340	0.8297	0.8888	none
PWY-5838: superpathway of menaquinol-8 biosynthesis I	0.0010	0.0012	0.0004	0.0006	-0.0340	0.8297	0.8888	none
TCA: TCA cycle I (prokaryotic)	0.0018	0.0022	0.0016	0.0022	0.0340	0.8297	0.8888	none
POLYISOPRENSYN-PWY: polyisoprenoid biosynthesis (E. coli)	0.0002	0.0003	0.0001	0.0001	0.0319	0.8402	0.8914	none
PWY-6185: 4-methylcatechol degradation (ortho cleavage)	0.0000	0.0000	0.0000	0.0000	-0.0319	0.8402	0.8914	none
PWY-6608: guanosine nucleotides degradation III	0.0017	0.0022	0.0009	0.0017	-0.0319	0.8402	0.8914	none
PWY-5005: biotin biosynthesis II	0.0007	0.0008	0.0002	0.0002	-0.0277	0.8613	0.9108	none
AST-PWY: L-arginine degradation II (AST pathway)	0.0002	0.0002	0.0000	0.0001	0.0255	0.8719	0.9161	none
P162-PWY: L-glutamate degradation V (via hydroxyglutarate)	0.0000	0.0000	0.0000	0.0000	-0.0255	0.8719	0.9161	none
LPSSYN-PWY: superpathway of lipopolysaccharide biosynthesis	0.0001	0.0002	0.0000	0.0000	-0.0234	0.8825	0.9243	none
UDPNAGSYN-PWY: UDP-N-acetyl-D-glucosamine biosynthesis I	0.0013	0.0017	0.0010	0.0013	-0.0212	0.8931	0.9325	none
PROTOCATECHUATE-ORTHO-CLEAVAGE-PWY: protocatechuate degradation II (ortho-cleavage pathway)	0.0000	0.0001	0.0000	0.0000	-0.0191	0.9037	0.9370	none
PWY66-400: glycolysis VI (metazoan)	0.0041	0.0049	0.0033	0.0039	0.0191	0.9037	0.9370	none
PWY0-1415: superpathway of heme biosynthesis from uroporphyrinogen-III	0.0002	0.0003	0.0001	0.0002	0.0171	0.9144	0.9370	none
PWY-5189: tetrapyrrole biosynthesis II (from glycine)	0.0004	0.0005	0.0002	0.0002	0.0171	0.9144	0.9370	none
PWY-5690: TCA cycle II (plants and fungi)	0.0022	0.0027	0.0012	0.0019	-0.0171	0.9144	0.9370	none
PWY-5920: superpathway of heme biosynthesis from glycine	0.0002	0.0002	0.0001	0.0001	-0.0171	0.9144	0.9370	none
PWY-4702: phytate degradation I	0.0002	0.0003	0.0000	0.0001	0.0149	0.9250	0.9450	none
PWY-6630: superpathway of L-tyrosine biosynthesis	0.0010	0.0013	0.0004	0.0008	0.0127	0.9357	0.9530	none

PWY-5918: superpathay of heme biosynthesis from glutamate	0.0003	0.0004	0.0002	0.0003	0.0106	0.9464	0.9609	none
PWY-5022: 4-aminobutanoate degradation V	0.0005	0.0005	0.0002	0.0003	0.0085	0.9571	0.9688	none
SALVADEHYPOX-PWY: adenosine nucleotides degradation II	0.0008	0.0010	0.0004	0.0005	-0.0064	0.9678	0.9767	none
HEMESYN2-PWY: heme biosynthesis II (anaerobic)	0.0006	0.0007	0.0003	0.0005	-0.0043	0.9786	0.9845	none
PWY-7315: dTDP-N-acetylthomosamine biosynthesis	0.0004	0.0005	0.0001	0.0001	-0.0021	0.9893	0.9893	none
TCA-GLYOX-BYPASS: superpathway of glyoxylate bypass and TCA	0.0006	0.0007	0.0002	0.0003	0.0021	0.9893	0.9893	none

ESM Table 5 : Network properties of species co-occurrence networks

	Hub score			
	Acarbose		Vildagliptin	
**Species**	**Baseline**	**6-month**	**Baseline**	**6-month**
Veillonella parvula	0.150	0.279	0.153	0.158
Streptococcus salivarius	0.086	0.274	0.012	0.090
Streptococcus mitis oralis pneumoniae	0.104	0.270	0.049	0.156
Veillonella atypica	0.093	0.265	0.031	0.046
Veillonella dispar	0.096	0.256	0.022	0.066
Rothia mucilaginosa	0.055	0.232	0.053	0.154
Streptococcus sanguinis	0.056	0.230	0.124	0.150
Streptococcus australis	0.057	0.207	0.052	0.132
Solobacterium moorei	0.013	0.207	0.072	0.115
Streptococcus parasanguinis	0.100	0.197	0.031	0.046
Streptococcus vestibularis	0.086	0.186	0.005	0.008
Streptococcus infantis	0.045	0.186	0.007	0.012
Bifidobacterium longum	0.043	0.185	0.137	0.078
Streptococcus anginosus	0.068	0.166	0.002	0.038
Lactobacillus mucosae	0.034	0.147	0.002	0.000
Haemophilus parainfluenzae	0.013	0.146	0.013	0.037
candidate division TM7 single cell isolate TM7c	0.112	0.125	0.116	0.149
Ruminococcus gnavus	0.285	0.102	0.294	0.292
Enterococcus faecium	0.063	0.094	0.019	0.000
Streptococcus thermophilus	0.006	0.071	0.019	0.002
Lachnospiraceae bacterium 2 1 58FAA	0.201	0.063	0.139	0.028
Lactobacillus salivarius	0.065	0.058	0.004	0.000
Enterobacter cloacae	0.014	0.047	0.004	0.021
Bifidobacterium bifidum	0.000	0.041	0.039	0.039
Citrobacter freundii	0.010	0.038	0.049	0.003
Lachnospiraceae bacterium 5 1 63FAA	0.161	0.035	0.024	0.108
Anaerostipes hadrus	0.054	0.034	0.025	0.086
Bifidobacterium adolescentis	0.000	0.025	0.000	0.000
Bifidobacterium pseudocatenulatum	0.032	0.021	0.079	0.036
Clostridium perfringens	0.103	0.014	0.001	0.000
Clostridium ramosum	0.102	0.013	0.139	0.206
Clostridium nexile	0.178	0.010	0.221	0.216
Collinsella aerofaciens	0.000	0.007	0.000	0.000

Bacteroides fragilis	0.150	0.004	0.145	0.092
Bacteroides dorei	0.000	0.003	0.000	0.000
Megasphaera elsdenii	0.001	0.002	0.005	0.000
Dorea formicigenerans	0.000	0.002	0.000	0.000
Akkermansia muciniphila	0.000	0.001	0.000	0.000
Escherichia coli	0.160	0.001	0.155	0.084
Clostridium hathewayi	0.155	0.001	0.272	0.177
Eubacterium rectale	0.000	0.001	0.000	0.000
Acidaminococcus intestini	0.020	0.000	0.029	0.000
Clostridiales bacterium 1 7 47FAA	0.036	0.000	0.143	0.194
Bacteroides faecis	0.000	0.000	0.000	0.001
Lachnospiraceae bacterium 1 4 56FAA	0.172	0.000	0.164	0.197
Flavonifractor plautii	0.144	0.000	0.202	0.155
Clostridium symbiosum	0.115	0.000	0.225	0.136
Lachnospiraceae bacterium 7 1 58FAA	0.042	0.000	0.065	0.096
Bacteroides nordii	0.005	0.000	0.000	0.000
Adlercreutzia equolifaciens	0.000	0.000	0.000	0.000
Alistipes senegalensis	0.000	0.000	0.000	0.000
Bacteroides cellulosilyticus	0.000	0.000	0.000	0.000
Bacteroides intestinalis	0.000	0.000	0.000	0.000
Clostridium leptum	0.000	0.000	0.000	0.000
Oxalobacter formigenes	0.000	0.000	0.000	0.000
Parabacteroides goldsteinii	0.000	0.000	0.000	0.006
Paraprevotella clara	0.000	0.000	0.000	0.001
Paraprevotella xylaniphila	0.000	0.000	0.000	0.001
Bacteroides ovatus	0.143	0.000	0.009	0.000
Clostridium bolteae	0.129	0.000	0.212	0.210
Klebsiella pneumoniae	0.081	0.000	0.054	0.037
Clostridium bartlettii	0.057	0.000	0.018	0.009
Dialister invisus	0.044	0.000	0.013	0.033
Bacteroides thetaiotaomicron	0.031	0.000	0.001	0.106
Clostridium citroniae	0.030	0.000	0.056	0.020
Bacteroides uniformis	0.022	0.000	0.000	0.005
Burkholderiales bacterium 1 1 47	0.017	0.000	0.000	0.000
Clostridium asparagiforme	0.017	0.000	0.020	0.079
Lachnospiraceae bacterium 3 1 46FAA	0.009	0.000	0.017	0.019
Bacteroides vulgatus	0.008	0.000	0.000	0.002
Roseburia intestinalis	0.008	0.000	0.000	0.000

Lachnospiraceae bacterium 8 1 57FAA	0.007	0.000	0.032	0.000
Parasutterella excrementihominis	0.003	0.000	0.000	0.000
Ruminococcus torques	0.000	0.000	0.058	0.141
Alistipes indistinctus	0.000	0.000	0.000	0.010
Bacteroides salyersiae	0.000	0.000	0.000	0.006
Bacteroides xylanisolvens	0.000	0.000	0.012	0.059
Eubacterium ramulus	0.000	0.000	0.000	0.010
Megamonas funiformis	0.000	0.000	0.024	0.002
Megamonas hypermegale	0.000	0.000	0.004	0.005
Megamonas rupellensis	0.000	0.000	0.044	0.012
Pyramidobacter piscolens	0.000	0.000	0.000	0.003
Ruminococcus sp 5 1 39BFAA	0.000	0.000	0.000	0.011

ESM Table 6: Comparison of species/pathway relative abundances between baseline and 3-month in published groups (Acarbose or Glipizide)

ESM Table 6a: Comparison of species relative abundances between baseline and 3-month in the published acarbose group

Species	Relative abundance (mean)		Relative abundance (median)		Baseline vs. M3 (Wilcoxon signed-rank test, clr-transformed)			
	Baseline	3-month	Baseline	3-month	Effect_size	P-value	BH adjusted P-value	Enrichment
Lactobacillus mucosae	0.0434	0.7363	0.0000	0.2750	0.8490	0.0000	0.0000	M3
Clostridium bolteae	0.2827	0.0175	0.0505	0.0000	-0.7600	0.0000	0.0000	base
Holdemania filiformis	0.0433	0.0066	0.0222	0.0000	-0.7510	0.0000	0.0000	base
Roseburia intestinalis	1.1535	0.0583	0.3152	0.0000	-0.7460	0.0000	0.0000	base
Bilophila unclassified	0.4818	0.1222	0.3484	0.0319	-0.7440	0.0000	0.0000	base
Bacteroides thetaiotaomicron	1.3780	0.3750	0.5758	0.0673	-0.7390	0.0000	0.0000	base
Alistipes putredinis	3.5738	0.9986	2.5760	0.0144	-0.7240	0.0000	0.0000	base
Streptococcus salivarius	0.7619	1.8619	0.0551	0.3708	0.7200	0.0000	0.0000	M3
Oscillibacter unclassified	0.5926	0.1737	0.3030	0.0100	-0.7090	0.0000	0.0000	base
Lachnospiraceae bacterium 7 1 58FAA	0.0133	0.0061	0.0091	0.0000	-0.7060	0.0000	0.0000	base
Bilophila wadsworthia	0.0475	0.0149	0.0334	0.0019	-0.7040	0.0000	0.0000	base
Roseburia inulinivorans	1.6051	0.4113	0.6729	0.0069	-0.6890	0.0000	0.0000	base
Bacteroides massiliensis	3.8600	0.4807	0.2794	0.0044	-0.6830	0.0000	0.0000	base
Parabacteroides goldsteinii	0.1308	0.0538	0.0383	0.0000	-0.6830	0.0000	0.0000	base
Bifidobacterium longum	0.8637	10.5588	0.0433	3.1525	0.6830	0.0000	0.0000	M3
Roseburia hominis	0.3280	0.0911	0.1268	0.0000	-0.6790	0.0000	0.0000	base
Bacteroides dorei	2.1408	0.2469	0.3082	0.0301	-0.6500	0.0000	0.0000	base
Bacteroides cellulosilyticus	0.3059	0.2250	0.0958	0.0002	-0.6440	0.0000	0.0000	base
Megasphaera unclassified	0.0864	0.8132	0.0000	0.0010	0.6340	0.0000	0.0001	M3
Prevotella stercorea	0.3731	0.9227	0.0000	0.0000	0.6300	0.0000	0.0001	M3
Eubacterium ramulus	0.1154	0.1150	0.0484	0.0056	-0.6250	0.0000	0.0001	base
Bacteroides uniformis	2.0056	0.9960	0.7810	0.1035	-0.6200	0.0000	0.0001	base
Bacteroides faecis	0.0982	0.0912	0.0036	0.0004	-0.6160	0.0000	0.0001	base
Clostridium asparagiforme	0.0282	0.0085	0.0119	0.0000	-0.6160	0.0000	0.0001	base
Flavonifractor plautii	0.0588	0.0161	0.0121	0.0000	-0.6160	0.0000	0.0001	base
Odoribacter splanchnicus	0.4793	0.1438	0.4065	0.0000	-0.6160	0.0000	0.0001	base

Bacteroides xylanisolvens	0.4249	0.2348	0.1195	0.0077	-0.6130	0.0000	0.0001	base
Clostridium citroniae	0.0233	0.0101	0.0127	0.0000	-0.6130	0.0000	0.0001	base
Collinsella aerofaciens	0.7115	1.7199	0.5491	0.9961	0.5970	0.0000	0.0001	M3
Anaerotruncus colihominis	0.0402	0.0047	0.0033	0.0000	-0.5830	0.0000	0.0002	base
Bacteroides intestinalis	0.2820	0.0700	0.0484	0.0000	-0.5760	0.0000	0.0002	base
Lachnospiraceae bacterium 3 1 46FAA	0.0508	0.0292	0.0140	0.0000	-0.5730	0.0000	0.0002	base
Olsenella unclassified	0.0015	0.0948	0.0000	0.0005	0.5660	0.0001	0.0003	M3
Alistipes shahii	0.6556	0.4476	0.3226	0.0088	-0.5640	0.0001	0.0003	base
Roseburia unclassified	0.0498	0.2050	0.0000	0.0024	0.5600	0.0001	0.0003	M3
Bifidobacterium pseudocatenulatum	0.6092	2.4560	0.0006	0.0584	0.5450	0.0001	0.0005	M3
Eubacterium ventriosum	0.2887	0.0966	0.1307	0.0216	-0.5410	0.0001	0.0005	base
Eggerthella unclassified	0.0521	0.0128	0.0106	0.0023	-0.5350	0.0001	0.0006	base
Lachnospiraceae bacterium 8 1 57FAA	0.0209	0.1124	0.0000	0.0000	0.5350	0.0001	0.0006	M3
Adlercreutzia equolifaciens	0.0686	0.0152	0.0305	0.0000	-0.5340	0.0001	0.0006	base
Eubacterium eligens	2.2526	0.7389	0.8295	0.0181	-0.5290	0.0002	0.0006	base
Bacteroides finegoldii	0.5367	0.0716	0.0016	0.0000	-0.5110	0.0003	0.0011	base
Bacteroides vulgatus	3.0887	1.2051	0.8080	0.1614	-0.5030	0.0003	0.0013	base
Lachnospiraceae bacterium 1 1 57FAA	0.5554	0.1941	0.1095	0.0147	-0.5030	0.0003	0.0013	base
Clostridium hathewayi	0.0308	0.0085	0.0058	0.0000	-0.5000	0.0004	0.0013	base
Dialister invisus	0.2576	0.1956	0.0000	0.0000	0.4970	0.0004	0.0014	M3
Clostridium leptum	0.0432	0.0144	0.0083	0.0000	-0.4940	0.0004	0.0015	base
Eubacterium hallii	0.2707	0.1839	0.1833	0.0816	-0.4910	0.0005	0.0016	base
Dasheen mosaic virus	0.0003	0.0008	0.0000	0.0000	0.4890	0.0005	0.0017	M3
Lachnospiraceae bacterium 1 4 56FAA	0.0462	0.0044	0.0036	0.0000	-0.4830	0.0006	0.0019	base
Parabacteroides distasonis	0.7363	0.5885	0.2613	0.0397	-0.4820	0.0006	0.0019	base
Alistipes indistinctus	0.2282	0.1283	0.0396	0.0000	-0.4770	0.0006	0.0021	base
Bacteroides salyersiae	0.3332	0.0882	0.0040	0.0000	-0.4760	0.0007	0.0021	base
Butyricimonas synergistica	0.0148	0.0051	0.0004	0.0000	-0.4760	0.0007	0.0021	base
Alistipes finegoldii	0.1515	0.0969	0.0186	0.0000	-0.4730	0.0007	0.0022	base
Alistipes onderdonkii	0.8844	0.3211	0.1048	0.0026	-0.4680	0.0008	0.0025	base
Bacteroides stercoris	3.7119	1.3521	0.4535	0.0127	-0.4580	0.0011	0.0031	base
Bacteroides caccae	0.8744	0.3949	0.5170	0.0082	-0.4230	0.0025	0.0073	base
Clostridiales bacterium 1 7 47FAA	0.0151	0.0114	0.0018	0.0000	-0.4190	0.0028	0.0079	base
Bacteroides nordii	0.0296	0.0196	0.0054	0.0000	-0.4120	0.0032	0.0090	base
Streptococcus anginosus	0.0054	0.0214	0.0000	0.0000	0.4090	0.0036	0.0097	M3
Desulfovibrio desulfuricans	0.0291	0.0016	0.0010	0.0000	-0.4070	0.0037	0.0099	base
Bacteroides clarus	0.1132	0.0214	0.0015	0.0000	-0.3960	0.0046	0.0123	base
Veillonella parvula	0.1148	0.4337	0.0089	0.0195	0.3950	0.0048	0.0125	M3

Streptococcus gordonii	0.0041	0.0070	0.0000	0.0014	0.3930	0.0049	0.0125	M3
Ruminococcus lactaris	0.5629	0.1672	0.2228	0.0005	-0.3930	0.0049	0.0125	base
Bifidobacterium adolescentis	0.4051	5.3557	0.0004	0.0013	0.3910	0.0052	0.0128	M3
Veillonella atypica	0.0711	0.0244	0.0000	0.0047	0.3910	0.0052	0.0128	M3
Bacteroides fragilis	1.2049	0.5489	0.1027	0.0317	-0.3880	0.0057	0.0138	base
Veillonella unclassified	0.6391	0.8721	0.0097	0.0273	0.3860	0.0059	0.0140	M3
Ruminococcus gnavus	1.1079	1.6846	0.0894	0.0228	-0.3810	0.0066	0.0154	base
Gordonibacter pamelaeae	0.0225	0.0039	0.0045	0.0000	-0.3770	0.0071	0.0163	base
Streptococcus vestibularis	0.0276	0.0478	0.0000	0.0000	0.3770	0.0071	0.0163	M3
Veillonella dispar	0.0227	0.0095	0.0000	0.0006	0.3670	0.0089	0.0201	M3
Oxalobacter formigenes	0.0366	0.0074	0.0000	0.0000	-0.3610	0.0099	0.0221	base
Ruminococcus sp 5 1 39BFAA	0.5563	0.2696	0.2884	0.1333	-0.3580	0.0105	0.0231	base
Streptococcus cristatus	0.0012	0.0013	0.0000	0.0000	0.3470	0.0130	0.0282	M3
Megamonas funiformis	0.1197	0.2295	0.0000	0.0000	0.3390	0.0156	0.0334	M3
Bacteroidales bacterium ph8	0.4167	0.2492	0.0652	0.0008	-0.3160	0.0239	0.0505	none
Gemella sanguinis	0.0032	0.0062	0.0000	0.0000	0.3040	0.0304	0.0633	none
Alistipes sp AP11	0.0694	0.0435	0.0000	0.0000	0.3020	0.0311	0.0633	none
Haemophilus parainfluenzae	0.1926	0.2078	0.0062	0.0321	0.3020	0.0311	0.0633	none
Peptostreptococcus unclassified	0.0007	0.0005	0.0000	0.0000	0.2980	0.0334	0.0671	none
Eggerthella lenta	0.0066	0.0013	0.0004	0.0000	-0.2940	0.0358	0.0711	none
Parabacteroides merdae	0.8071	0.4736	0.5934	0.0215	-0.2910	0.0374	0.0736	none
Bacteroides plebeius	4.6442	2.1306	0.4261	0.0236	-0.2900	0.0383	0.0744	none
Streptococcus sanguinis	0.0085	0.0128	0.0007	0.0016	0.2840	0.0419	0.0805	none
Bacteroides ovatus	1.8334	1.4941	0.5123	0.1999	-0.2790	0.0469	0.0890	none
Alistipes senegalensis	0.0306	0.0176	0.0014	0.0000	-0.2730	0.0512	0.0961	none
Clostridiaceae bacterium JC118	0.0035	0.0016	0.0000	0.0000	0.2630	0.0596	0.1105	none
Ruminococcus obeum	0.2633	0.1912	0.1929	0.1034	-0.2620	0.0608	0.1116	none
Prevotella copri	10.7286	12.2229	0.0271	0.0274	0.2620	0.0621	0.1128	none
Mitsuokella unclassified	0.0259	0.0351	0.0019	0.0036	0.2580	0.0662	0.1188	none
Megamonas unclassified	1.2847	1.2356	0.0039	0.0033	-0.2310	0.0990	0.1759	none
Subdoligranulum variabile	0.0014	0.0013	0.0000	0.0000	0.2300	0.1009	0.1774	none
Holdemania unclassified	0.0028	0.0026	0.0000	0.0000	0.2250	0.1069	0.1857	none
Escherichia coli	1.8894	0.6408	0.2114	0.0918	-0.2240	0.1090	0.1857	none
Ruminococcaceae bacterium D16	0.0042	0.0014	0.0000	0.0000	-0.2240	0.1090	0.1857	none
Bacteroides coprocola	2.1194	1.2335	0.0021	0.0008	-0.2170	0.1220	0.2057	none
Klebsiella pneumoniae	0.3430	1.0421	0.0007	0.0000	-0.2160	0.1242	0.2075	none
Actinomyces odontolyticus	0.0007	0.0003	0.0000	0.0000	0.2140	0.1265	0.2092	none
Paraprevotella unclassified	0.5432	0.1642	0.0008	0.0001	-0.2100	0.1337	0.2189	none

Coprococcus comes	0.3424	1.3369	0.2224	0.1243	0.1990	0.1542	0.2501	none
Klebsiella unclassified	0.0140	0.0106	0.0000	0.0000	0.1990	0.1570	0.2520	none
Clostridium symbiosum	0.0271	0.0104	0.0017	0.0000	-0.1890	0.1771	0.2817	none
Granulicatella adiacens	0.0038	0.0057	0.0000	0.0002	0.1880	0.1801	0.2838	none
Paraprevotella xylaniphila	0.0628	0.0128	0.0000	0.0000	-0.1860	0.1832	0.2859	none
Sutterella wadsworthensis	0.7651	0.7644	0.0001	0.0000	0.1850	0.1863	0.2880	none
Dorea formicigenerans	0.3774	0.2666	0.3148	0.1391	-0.1830	0.1894	0.2902	none
Bacteroides oleiciplenus	0.0090	0.0022	0.0000	0.0000	-0.1810	0.1958	0.2973	none
Parabacteroides johnsonii	0.0413	0.0338	0.0000	0.0000	-0.1790	0.1991	0.2995	none
Subdoligranulum unclassified	2.0613	1.8101	1.3627	0.6561	-0.1740	0.2160	0.3220	none
Streptococcus australis	0.0094	0.0143	0.0049	0.0061	0.1690	0.2266	0.3349	none
Paraprevotella clara	0.1621	0.0668	0.0000	0.0000	-0.1620	0.2451	0.3559	none
Ruminococcus flavefaciens	0.0006	0.0002	0.0000	0.0000	0.1620	0.2451	0.3559	none
Eubacterium biforme	0.2904	0.0978	0.0000	0.0000	0.1610	0.2489	0.3584	none
Clostridium nexile	0.4312	0.0397	0.0004	0.0000	-0.1600	0.2528	0.3608	none
Clostridium ramosum	0.0105	0.0251	0.0000	0.0000	0.1580	0.2567	0.3633	none
Escherichia unclassified	0.1164	0.0948	0.0254	0.0064	-0.1500	0.2853	0.4003	none
Candidatus Zinderia insecticola	0.0006	0.0008	0.0002	0.0003	0.1430	0.3069	0.4271	none
Enterobacter cloacae	0.2235	0.0022	0.0000	0.0000	-0.1340	0.3390	0.4679	none
Anaerotruncus unclassified	0.0139	0.0071	0.0073	0.0037	-0.1330	0.3438	0.4706	none
Faecalibacterium prausnitzii	6.7559	7.5327	6.4727	5.4825	0.1290	0.3583	0.4865	none
Eubacterium infirmum	0.0008	0.0002	0.0000	0.0000	0.1270	0.3632	0.4892	none
candidate division TM7 single cell isolate	0.0031	0.0014	0.0000	0.0000	-0.1190	0.3937	0.5259	none
Ruminococcus torques	0.9279	1.5472	0.7446	0.5710	0.1180	0.3989	0.5287	none
Streptococcus infantis	0.0049	0.0104	0.0019	0.0026	0.1120	0.4256	0.5596	none
Clostridium bartlettii	0.0272	0.0541	0.0150	0.0074	-0.1080	0.4421	0.5768	none
Erysipelotrichaceae bacterium 6 1 45	0.0023	0.0028	0.0000	0.0000	0.1050	0.4533	0.5835	none
Lachnospiraceae bacterium 3 1 57FAA	0.0049	0.0064	0.0000	0.0000	0.1040	0.4590	0.5835	none
Streptococcus parasanguinis	0.1091	0.0752	0.0125	0.0122	0.1040	0.4590	0.5835	none
Barnesiella intestinihominis	0.3525	0.2211	0.0000	0.0000	0.1020	0.4647	0.5835	none
Ruminococcus bromii	0.8592	1.4233	0.0063	0.0043	-0.1020	0.4647	0.5835	none
Parabacteroides unclassified	1.0804	1.6902	0.0631	0.0366	0.1010	0.4704	0.5863	none
Bacteroides eggerthii	0.5227	0.4713	0.0007	0.0008	-0.0958	0.4938	0.6109	none
Erysipelotrichaceae bacterium 2 2 44A	0.0015	0.0006	0.0000	0.0000	0.0919	0.5117	0.6284	none
Anaerostipes hadrus	0.0186	0.0902	0.0043	0.0027	0.0840	0.5486	0.6687	none
Ruminococcus callidus	0.1985	0.1163	0.0155	0.0000	-0.0801	0.5675	0.6867	none
Gemella unclassified	0.0005	0.0004	0.0000	0.0000	0.0787	0.5738	0.6894	none
Rothia mucilaginosa	0.0169	0.0239	0.0044	0.0013	-0.0723	0.6062	0.7206	none

Coprobacillus unclassified	0.1498	0.1827	0.0001	0.0000	-0.0709	0.6127	0.7206	none
Fusobacterium mortiferum	0.2280	0.0142	0.0000	0.0000	0.0709	0.6127	0.7206	none
Clostridium clostridioforme	0.1130	0.0028	0.0000	0.0000	-0.0682	0.6260	0.7310	none
Clostridium innocuum	0.0018	0.0004	0.0000	0.0000	0.0643	0.6460	0.7440	none
Deinococcus unclassified	0.0014	0.0009	0.0000	0.0000	0.0643	0.6460	0.7440	none
Cellulophaga unclassified	0.0017	0.0003	0.0000	0.0000	-0.0564	0.6869	0.7857	none
Megamonas hypermegale	0.3232	0.1920	0.0000	0.0000	-0.0472	0.7358	0.8359	none
Lachnospiraceae bacterium 2 1 58FAA	0.0472	0.3617	0.0042	0.0010	0.0459	0.7429	0.8382	none
Eubacterium rectale	7.3176	7.9513	1.2971	0.2421	0.0433	0.7571	0.8485	none
Dorea longicatena	0.2882	0.4634	0.1016	0.1043	-0.0407	0.7714	0.8521	none
Eubacterium siraeum	0.3567	0.0733	0.0000	0.0000	-0.0407	0.7714	0.8521	none
Ruminococcus albus	0.0010	0.0003	0.0000	0.0000	0.0393	0.7786	0.8521	none
Burkholderiales bacterium 1 1 47	0.1517	0.0973	0.0031	0.0093	0.0381	0.7858	0.8521	none
Solobacterium moorei	0.0028	0.0032	0.0002	0.0000	0.0381	0.7858	0.8521	none
Pedobacter unclassified	0.0037	0.0012	0.0000	0.0000	-0.0302	0.8293	0.8935	none
Erysipelotrichaceae bacterium 21 3	0.0053	0.0006	0.0000	0.0000	0.0276	0.8440	0.9035	none
Coprococcus catus	0.0932	0.1337	0.0728	0.0423	0.0249	0.8586	0.9076	none
Pseudoflavonifractor capillosus	0.0008	0.0003	0.0000	0.0000	-0.0249	0.8586	0.9076	none
Lachnospiraceae bacterium 5 1 63FAA	0.0783	0.1327	0.0430	0.0221	0.0223	0.8734	0.9173	none
Streptococcus mitis oralis pneumoniae	0.0103	0.0102	0.0027	0.0028	-0.0197	0.8882	0.9270	none
Akkermansia muciniphila	0.9997	0.7173	0.0011	0.0000	0.0171	0.9030	0.9367	none
Granulicatella unclassified	0.0079	0.0085	0.0050	0.0032	0.0157	0.9104	0.9385	none
Butyrivibrio unclassified	0.0117	0.0103	0.0097	0.0062	-0.0131	0.9253	0.9422	none
Megamonas rupellensis	0.2655	0.0970	0.0000	0.0000	0.0131	0.9253	0.9422	none
Parasutterella excrementihominis	0.1277	0.0851	0.0043	0.0114	0.0118	0.9328	0.9441	none
Peptostreptococcaceae noname unclassifi	0.0510	0.0246	0.0039	0.0037	-0.0066	0.9626	0.9684	none
Coprobacter fastidiosus	0.0340	0.0130	0.0000	0.0000	0.0026	0.9850	0.9850	none

EMS Table 6b: Comparison of pathway relative abundances between baseline and 3-month in the published acarbose group

Pathway	Relative abundance (mean)		Relative abundance (median)		Baseline vs. M3 (Wilcoxon signed-rank test, clr-transformed)			
	Baseline	3-month	Baseline	3-month	Effect_size	P-value	BH adjusted P-value	Enrichment

ARGSYNBSUB-PWY: L-arginine biosynthesis II (acetyl cycle)	0.0001	0.0003	0.0000	0.0002	0.7530	0.0000	0.0000	M3
UDPNAGSYN-PWY: UDP-N-acetyl-D-glucosamine biosynthesis I	0.0001	0.0003	0.0001	0.0002	0.7300	0.0000	0.0000	M3
PWY-1269: CMP-3-deoxy-D-manno-octulosonate biosynthesis I	0.0001	0.0000	0.0000	0.0000	-0.6810	0.0000	0.0001	base
PWY-6609: adenine and adenosine salvage III	0.0001	0.0001	0.0000	0.0001	0.6600	0.0000	0.0002	M3
PWY0-1296: purine ribonucleosides degradation	0.0001	0.0002	0.0001	0.0001	0.6410	0.0000	0.0002	M3
OANTIGEN-PWY: O-antigen building blocks biosynthesis (E. coli)	0.0001	0.0002	0.0001	0.0002	0.6300	0.0000	0.0003	M3
MET-SAM-PWY: superpathway of S-adenosyl-L-methionine biosynth	0.0001	0.0002	0.0001	0.0002	0.5970	0.0000	0.0006	M3
PWY-6122: 5-aminoimidazole ribonucleotide biosynthesis II	0.0001	0.0004	0.0001	0.0003	0.5980	0.0000	0.0006	M3
PWY-6277: superpathway of 5-aminoimidazole ribonucleotide bios	0.0001	0.0004	0.0001	0.0003	0.5980	0.0000	0.0006	M3
HOMOSER-METSYN-PWY: L-methionine biosynthesis I	0.0001	0.0001	0.0001	0.0001	0.5920	0.0000	0.0006	M3
ARGSYN-PWY: L-arginine biosynthesis I (via L-ornithine)	0.0002	0.0003	0.0001	0.0002	0.5700	0.0000	0.0007	M3
METSYN-PWY: L-homoserine and L-methionine biosynthesis	0.0001	0.0002	0.0001	0.0002	0.5810	0.0000	0.0007	M3
PWY-2941: L-lysine biosynthesis II	0.0001	0.0002	0.0000	0.0001	0.5690	0.0000	0.0007	M3
PWY-5022: 4-aminobutanoate degradation V	0.0000	0.0000	0.0000	0.0000	-0.5670	0.0001	0.0007	base
PWY-5347: superpathway of L-methionine biosynthesis (transsulfur	0.0001	0.0002	0.0001	0.0002	0.5780	0.0000	0.0007	M3
PWY-6121: 5-aminoimidazole ribonucleotide biosynthesis I	0.0001	0.0004	0.0001	0.0004	0.5700	0.0000	0.0007	M3
PWY-6151: S-adenosyl-L-methionine cycle I	0.0001	0.0003	0.0001	0.0001	0.5760	0.0000	0.0007	M3
PWY-7400: L-arginine biosynthesis IV (archaebacteria)	0.0002	0.0003	0.0002	0.0002	0.5690	0.0000	0.0007	M3
PYRIDNUCSAL-PWY: NAD salvage pathway I	0.0000	0.0001	0.0000	0.0000	0.5460	0.0001	0.0013	M3
HISTSYN-PWY: L-histidine biosynthesis	0.0002	0.0003	0.0001	0.0002	0.5380	0.0001	0.0015	M3
PWY-5030: L-histidine degradation III	0.0000	0.0000	0.0000	0.0000	-0.5390	0.0001	0.0015	base
ARGININE-SYN4-PWY: L-ornithine de novo biosynthesis	0.0000	0.0000	0.0000	0.0000	-0.5350	0.0001	0.0016	base
FASYN-ELONG-PWY: fatty acid elongation -- saturated	0.0001	0.0000	0.0000	0.0000	-0.5310	0.0002	0.0016	base
P441-PWY: superpathway of N-acetylneuraminate degradation	0.0001	0.0000	0.0000	0.0000	-0.5290	0.0002	0.0016	base
PWY-7663: gondoate biosynthesis (anaerobic)	0.0001	0.0000	0.0000	0.0000	-0.5310	0.0002	0.0016	base
PWY-4981: L-proline biosynthesis II (from arginine)	0.0000	0.0000	0.0000	0.0000	-0.5240	0.0002	0.0018	base
PWY-6700: queuosine biosynthesis	0.0001	0.0000	0.0000	0.0000	-0.5080	0.0003	0.0027	base
PWY-5659: GDP-mannose biosynthesis	0.0001	0.0001	0.0001	0.0000	-0.5040	0.0003	0.0029	base
GLUTORN-PWY: L-ornithine biosynthesis	0.0001	0.0001	0.0000	0.0001	0.5010	0.0003	0.0030	M3
THISYNARA-PWY: superpathway of thiamin diphosphate biosynthes	0.0002	0.0001	0.0001	0.0001	-0.4980	0.0004	0.0031	base
PWY-621: sucrose degradation III (sucrose invertase)	0.0002	0.0004	0.0002	0.0004	0.4830	0.0006	0.0046	M3
PWY-3001: superpathway of L-isoleucine biosynthesis I	0.0003	0.0005	0.0003	0.0004	0.4800	0.0006	0.0047	M3
PWY-7383: anaerobic energy metabolism (invertebrates, cytosol)	0.0000	0.0000	0.0000	0.0000	-0.4800	0.0006	0.0047	base
PWY-7664: oleate biosynthesis IV (anaerobic)	0.0000	0.0000	0.0000	0.0000	-0.4790	0.0006	0.0047	base
THRESYN-PWY: superpathway of L-threonine biosynthesis	0.0003	0.0004	0.0003	0.0004	0.4720	0.0008	0.0056	M3
HSERMETANA-PWY: L-methionine biosynthesis III	0.0001	0.0000	0.0001	0.0000	-0.4690	0.0008	0.0058	base
PWY-6123: inosine-5'-phosphate biosynthesis I	0.0002	0.0004	0.0002	0.0004	0.4660	0.0009	0.0061	M3
PWY-6386: UDP-N-acetylmuramoyl-pentapeptide biosynthesis II (lys	0.0000	0.0000	0.0000	0.0000	0.4630	0.0009	0.0063	M3

PWY-7328: superpathway of UDP-glucose-derived O-antigen buildin	0.0000	0.0000	0.0000	0.0000	-0.4610	0.0010	0.0066	base
ASPASN-PWY: superpathway of L-aspartate and L-asparagine biosyn	0.0001	0.0001	0.0001	0.0001	-0.4580	0.0011	0.0067	base
PWY-7111: pyruvate fermentation to isobutanol (engineered)	0.0004	0.0006	0.0004	0.0006	0.4580	0.0011	0.0067	M3
PWY-7184: pyrimidine deoxyribonucleotides de novo biosynthesis I	0.0001	0.0000	0.0000	0.0000	-0.4490	0.0013	0.0082	base
ILEUSYN-PWY: L-isoleucine biosynthesis I (from threonine)	0.0004	0.0007	0.0005	0.0006	0.4420	0.0016	0.0085	M3
PWY-5973: cis-vaccenate biosynthesis	0.0001	0.0002	0.0001	0.0001	0.4450	0.0015	0.0085	M3
PWY-6353: purine nucleotides degradation II (aerobic)	0.0000	0.0001	0.0000	0.0000	0.4420	0.0016	0.0085	M3
PWY-6936: seleno-amino acid biosynthesis	0.0000	0.0001	0.0000	0.0001	0.4410	0.0016	0.0085	M3
PWY-7210: pyrimidine deoxyribonucleotides biosynthesis from CTP	0.0000	0.0000	0.0000	0.0000	-0.4410	0.0016	0.0085	base
PWYG-321: mycolate biosynthesis	0.0000	0.0000	0.0000	0.0000	-0.4440	0.0015	0.0085	base
VALSYN-PWY: L-valine biosynthesis	0.0004	0.0007	0.0005	0.0006	0.4420	0.0016	0.0085	M3
GLYCOLYSIS-E-D: superpathway of glycolysis and Entner-Doudoroff	0.0000	0.0000	0.0000	0.0000	-0.4380	0.0017	0.0089	base
BRANCHED-CHAIN-AA-SYN-PWY: superpathway of branched amino a	0.0004	0.0006	0.0004	0.0005	0.4350	0.0019	0.0091	M3
GLUCONEO-PWY: gluconeogenesis I	0.0001	0.0000	0.0001	0.0000	-0.4350	0.0019	0.0091	base
RHAMCAT-PWY: L-rhamnose degradation I	0.0002	0.0001	0.0001	0.0001	-0.4340	0.0019	0.0093	base
PWY0-845: superpathway of pyridoxal 5'-phosphate biosynthesis an	0.0001	0.0000	0.0001	0.0000	-0.4300	0.0022	0.0103	base
PWY0-862: (5Z)-dodec-5-enoate biosynthesis	0.0000	0.0000	0.0000	0.0000	-0.4270	0.0023	0.0108	base
PYRIDOXSYN-PWY: pyridoxal 5'-phosphate biosynthesis I	0.0001	0.0000	0.0000	0.0000	-0.4260	0.0024	0.0109	base
PWY-6545: pyrimidine deoxyribonucleotides de novo biosynthesis I	0.0000	0.0000	0.0000	0.0000	-0.4210	0.0026	0.0118	base
P42-PWY: incomplete reductive TCA cycle	0.0000	0.0000	0.0000	0.0000	-0.4200	0.0027	0.0119	base
PWY-5913: TCA cycle VI (obligate autotrophs)	0.0001	0.0001	0.0000	0.0001	0.4160	0.0030	0.0129	M3
PWY-7371: 1,4-dihydroxy-6-naphthoate biosynthesis II	0.0000	0.0000	0.0000	0.0000	-0.4100	0.0033	0.0143	base
PWY-7282: 4-amino-2-methyl-5-phosphomethylpyrimidine biosynth	0.0003	0.0002	0.0003	0.0002	-0.4070	0.0037	0.0154	base
SER-GLYSYN-PWY: superpathway of L-serine and glycine biosynthesi	0.0001	0.0001	0.0000	0.0001	0.3920	0.0051	0.0209	M3
FERMENTATION-PWY: mixed acid fermentation	0.0000	0.0001	0.0000	0.0000	0.3850	0.0060	0.0245	M3
PWY-5667: CDP-diacylglycerol biosynthesis I	0.0000	0.0000	0.0000	0.0000	-0.3670	0.0087	0.0331	base
PWY-724: superpathway of L-lysine, L-threonine and L-methionine b	0.0004	0.0005	0.0004	0.0005	0.3670	0.0087	0.0331	M3
PWY0-1261: anhydromuropeptides recycling	0.0001	0.0001	0.0001	0.0001	0.3680	0.0084	0.0331	M3
PWY0-1319: CDP-diacylglycerol biosynthesis II	0.0000	0.0000	0.0000	0.0000	-0.3670	0.0087	0.0331	base
PHOSLIPSYN-PWY: superpathway of phospholipid biosynthesis I (ba	0.0000	0.0000	0.0000	0.0000	-0.3670	0.0089	0.0336	base
LACTOSECAT-PWY: lactose and galactose degradation I	0.0000	0.0000	0.0000	0.0000	0.3650	0.0092	0.0340	M3
PWY-6608: guanosine nucleotides degradation III	0.0000	0.0001	0.0000	0.0000	0.3510	0.0120	0.0421	M3
PWY-7211: superpathway of pyrimidine deoxyribonucleotides de no	0.0001	0.0001	0.0001	0.0000	-0.3510	0.0120	0.0421	base
PWY4FS-7: phosphatidylglycerol biosynthesis I (plastidic)	0.0000	0.0000	0.0000	0.0000	-0.3510	0.0120	0.0421	base
PWY4FS-8: phosphatidylglycerol biosynthesis II (non-plastidic)	0.0000	0.0000	0.0000	0.0000	-0.3510	0.0120	0.0421	base
PWY-5345: superpathway of L-methionine biosynthesis (by sulfhydr	0.0000	0.0000	0.0000	0.0000	-0.3420	0.0148	0.0512	none
PWY-5121: superpathway of geranylgeranyl diphosphate biosynthe	0.0000	0.0000	0.0000	0.0000	0.3370	0.0160	0.0546	none
CITRULBIO-PWY: L-citrulline biosynthesis	0.0001	0.0001	0.0001	0.0000	-0.3330	0.0173	0.0582	none

NAD-BIOSYNTHESIS-II: NAD salvage pathway II	0.0000	0.0000	0.0000	0.0000	0.3320	0.0177	0.0589	none
ARGORNPROST-PWY: arginine, ornithine and proline interconversio	0.0000	0.0000	0.0000	0.0000	-0.3280	0.0191	0.0627	none
SALVADEHYPOX-PWY: adenosine nucleotides degradation II	0.0000	0.0001	0.0000	0.0000	0.3260	0.0196	0.0635	none
BIOTIN-BIOSYNTHESIS-PWY: biotin biosynthesis I	0.0000	0.0000	0.0000	0.0000	-0.3220	0.0216	0.0693	none
FUC-RHAMCAT-PWY: superpathway of fucose and rhamnose degrad	0.0000	0.0000	0.0000	0.0000	-0.3190	0.0227	0.0719	none
DENOVOPURINE2-PWY: superpathway of purine nucleotides de nov	0.0001	0.0001	0.0001	0.0001	0.3150	0.0245	0.0755	none
PWY-5188: tetrapyrrole biosynthesis I (from glutamate)	0.0000	0.0000	0.0000	0.0000	-0.3150	0.0245	0.0755	none
PWY-6969: TCA cycle V (2-oxoglutarate:ferredoxin oxidoreductase)	0.0001	0.0001	0.0001	0.0001	-0.3090	0.0270	0.0822	none
PWY-6519: 8-amino-7-oxononanoate biosynthesis I	0.0000	0.0000	0.0000	0.0000	-0.3080	0.0276	0.0832	none
HEXITOLDEGSUPER-PWY: superpathway of hexitol degradation (bact	0.0001	0.0000	0.0001	0.0000	-0.3070	0.0283	0.0832	none
METHGLYUT-PWY: superpathway of methylglyoxal degradation	0.0000	0.0000	0.0000	0.0000	0.3070	0.0283	0.0832	none
PWY-5100: pyruvate fermentation to acetate and lactate II	0.0001	0.0001	0.0001	0.0000	-0.3050	0.0290	0.0843	none
DAPLYSINESYN-PWY: L-lysine biosynthesis I	0.0001	0.0000	0.0000	0.0000	-0.3020	0.0311	0.0894	none
PWY66-400: glycolysis VI (metazoan)	0.0001	0.0001	0.0001	0.0000	-0.3010	0.0318	0.0905	none
COA-PWY: coenzyme A biosynthesis I	0.0001	0.0002	0.0001	0.0002	0.2950	0.0349	0.0983	none
PWY-7228: superpathway of guanosine nucleotides de novo biosynt	0.0004	0.0003	0.0004	0.0002	-0.2870	0.0401	0.1116	none
GLYCOLYSIS: glycolysis I (from glucose 6-phosphate)	0.0001	0.0001	0.0001	0.0000	-0.2860	0.0410	0.1129	none
COLANSYN-PWY: colanic acid building blocks biosynthesis	0.0000	0.0000	0.0000	0.0000	-0.2840	0.0419	0.1142	none
P164-PWY: purine nucleobases degradation I (anaerobic)	0.0000	0.0000	0.0000	0.0000	-0.2830	0.0429	0.1144	none
PWY-5484: glycolysis II (from fructose 6-phosphate)	0.0001	0.0001	0.0001	0.0000	-0.2830	0.0429	0.1144	none
PWY-6803: phosphatidylcholine acyl editing	0.0000	0.0000	0.0000	0.0000	-0.2800	0.0459	0.1198	none
PWY0-1586: peptidoglycan maturation (meso-diaminopimelate con	0.0002	0.0001	0.0002	0.0001	-0.2800	0.0459	0.1198	none
PWY-7204: pyridoxal 5'-phosphate salvage II (plants)	0.0000	0.0000	0.0000	0.0000	-0.2790	0.0469	0.1201	none
TRNA-CHARGING-PWY: tRNA charging	0.0001	0.0000	0.0000	0.0000	-0.2790	0.0469	0.1201	none
PWY-5138: unsaturated, even numbered fatty acid β-oxidation	0.0000	0.0000	0.0000	0.0000	-0.2770	0.0480	0.1215	none
PWY66-422: D-galactose degradation V (Leloir pathway)	0.0003	0.0004	0.0003	0.0003	0.2740	0.0501	0.1258	none
PWY-5676: acetyl-CoA fermentation to butanoate II	0.0000	0.0000	0.0000	0.0000	0.2700	0.0535	0.1329	none
PWY-7221: guanosine ribonucleotides de novo biosynthesis	0.0005	0.0004	0.0005	0.0003	-0.2690	0.0547	0.1346	none
GLCMANNANAUT-PWY: superpathway of N-acetylglucosamine, N-ace	0.0001	0.0001	0.0001	0.0000	-0.2670	0.0559	0.1362	none
PWY-5840: superpathway of menaquinol-7 biosynthesis	0.0000	0.0000	0.0000	0.0000	0.2660	0.0571	0.1365	none
PWY-7013: L-1,2-propanediol degradation	0.0000	0.0000	0.0000	0.0000	-0.2660	0.0571	0.1365	none
GLYCOLYSIS-TCA-GLYOX-BYPASS: superpathway of glycolysis, pyruvate	0.0000	0.0000	0.0000	0.0000	-0.2620	0.0621	0.1446	none
PWY-6317: galactose degradation I (Leloir pathway)	0.0003	0.0004	0.0003	0.0003	0.2620	0.0621	0.1446	none
PWY-6897: thiamin salvage II	0.0000	0.0000	0.0000	0.0000	-0.2620	0.0621	0.1446	none
FUCCAT-PWY: fucose degradation	0.0000	0.0000	0.0000	0.0000	-0.2590	0.0648	0.1495	none
PWY-5154: L-arginine biosynthesis III (via N-acetyl-L-citrulline)	0.0001	0.0001	0.0000	0.0000	-0.2560	0.0676	0.1545	none
P124-PWY: Bifidobacterium shunt	0.0000	0.0000	0.0000	0.0000	0.2530	0.0704	0.1596	none

P4-PWY: superpathway of L-lysine, L-threonine and L-methionine bi	0.0001	0.0001	0.0000	0.0000	-0.2520	0.0719	0.1601	none
SO4ASSIM-PWY: sulfate reduction I (assimilatory)	0.0000	0.0000	0.0000	0.0000	-0.2520	0.0719	0.1601	none
P122-PWY: heterolactic fermentation	0.0000	0.0000	0.0000	0.0000	0.2510	0.0734	0.1620	none
PWY-5384: sucrose degradation IV (sucrose phosphorylase)	0.0000	0.0000	0.0000	0.0000	-0.2490	0.0749	0.1625	none
PWY0-1297: superpathway of purine deoxyribonucleosides degrada	0.0001	0.0000	0.0000	0.0000	-0.2490	0.0749	0.1625	none
ARO-PWY: chorismate biosynthesis I	0.0006	0.0008	0.0006	0.0007	0.2480	0.0765	0.1645	none
PWY-5690: TCA cycle II (plants and fungi)	0.0000	0.0000	0.0000	0.0000	-0.2450	0.0796	0.1699	none
COMPLETE-ARO-PWY: superpathway of aromatic amino acid biosynt	0.0006	0.0008	0.0006	0.0007	0.2420	0.0829	0.1740	none
PWY-1861: formaldehyde assimilation II (RuMP Cycle)	0.0000	0.0000	0.0000	0.0000	-0.2420	0.0829	0.1740	none
COA-PWY-1: coenzyme A biosynthesis II (mammalian)	0.0002	0.0003	0.0002	0.0002	0.2410	0.0846	0.1760	none
PWY-7357: thiamin formation from pyrithiamine and oxythiamine (y	0.0000	0.0000	0.0000	0.0000	-0.2390	0.0880	0.1817	none
TCA: TCA cycle I (prokaryotic)	0.0001	0.0001	0.0001	0.0001	-0.2340	0.0952	0.1950	none
PANTO-PWY: phosphopantothenate biosynthesis I	0.0006	0.0005	0.0006	0.0004	-0.2270	0.1049	0.2131	none
PWY-6385: peptidoglycan biosynthesis III (mycobacteria)	0.0004	0.0004	0.0004	0.0003	-0.2250	0.1069	0.2155	none
ANAEROFRUCAT-PWY: homolactic fermentation	0.0002	0.0001	0.0001	0.0001	-0.2210	0.1132	0.2263	none
PWY-5103: L-isoleucine biosynthesis III	0.0000	0.0000	0.0000	0.0000	-0.2200	0.1153	0.2288	none
NONOXIPENT-PWY: pentose phosphate pathway (non-oxidative bra	0.0004	0.0005	0.0004	0.0004	0.2200	0.1175	0.2314	none
GLYCOCAT-PWY: glycogen degradation I (bacterial)	0.0000	0.0000	0.0000	0.0000	-0.2140	0.1265	0.2454	none
PWY0-1298: superpathway of pyrimidine deoxyribonucleosides degr	0.0000	0.0000	0.0000	0.0000	-0.2140	0.1265	0.2454	none
GLYCOGENSYNTH-PWY: glycogen biosynthesis I (from ADP-D-Glucose	0.0000	0.0000	0.0000	0.0000	-0.2130	0.1289	0.2481	none
PWY0-781: aspartate superpathway	0.0001	0.0001	0.0000	0.0000	-0.2040	0.1437	0.2724	none
REDCITCYC: TCA cycle VIII (helicobacter)	0.0000	0.0000	0.0000	0.0000	-0.2040	0.1437	0.2724	none
PPGPPMET-PWY: ppGpp biosynthesis	0.0000	0.0000	0.0000	0.0000	-0.1990	0.1570	0.2954	none
PWY-6163: chorismate biosynthesis from 3-dehydroquinate	0.0006	0.0008	0.0006	0.0007	0.1970	0.1597	0.2985	none
P105-PWY: TCA cycle IV (2-oxoglutarate decarboxylase)	0.0000	0.0000	0.0000	0.0000	-0.1950	0.1654	0.3068	none
ALL-CHORISMATE-PWY: superpathway of chorismate metabolism	0.0000	0.0000	0.0000	0.0000	-0.1920	0.1711	0.3152	none
PWY-7229: superpathway of adenosine nucleotides de novo biosyn	0.0001	0.0002	0.0001	0.0001	0.1900	0.1741	0.3184	none
ANAGLYCOLYSIS-PWY: glycolysis III (from glucose)	0.0005	0.0005	0.0005	0.0005	0.1880	0.1801	0.3202	none
GLYOXYLATE-BYPASS: glyoxylate cycle	0.0000	0.0000	0.0000	0.0000	-0.1880	0.1801	0.3202	none
POLYISOPRENSYN-PWY: polyisoprenoid biosynthesis (E. coli)	0.0000	0.0000	0.0000	0.0000	0.1880	0.1801	0.3202	none
PWY-5918: superpathay of heme biosynthesis from glutamate	0.0000	0.0000	0.0000	0.0000	-0.1880	0.1801	0.3202	none
PWY-7187: pyrimidine deoxyribonucleotides de novo biosynthesis II	0.0001	0.0001	0.0001	0.0001	0.1860	0.1832	0.3234	none
PWY-5971: palmitate biosynthesis II (bacteria and plants)	0.0001	0.0000	0.0000	0.0000	-0.1690	0.2266	0.3973	none
LPSSYN-PWY: superpathway of lipopolysaccharide biosynthesis	0.0000	0.0000	0.0000	0.0000	-0.1680	0.2302	0.4009	none
AEROBACTINSYN-PWY: aerobactin biosynthesis	0.0000	0.0000	0.0000	0.0000	0.1640	0.2413	0.4146	none
PWY-6630: superpathway of L-tyrosine biosynthesis	0.0000	0.0000	0.0000	0.0000	-0.1640	0.2413	0.4146	none
NAGLIPASYN-PWY: lipid IVA biosynthesis	0.0000	0.0000	0.0000	0.0000	-0.1600	0.2528	0.4250	none
PWY-5686: UMP biosynthesis	0.0004	0.0003	0.0004	0.0003	-0.1570	0.2607	0.4250	none

PWY-5863: superpathway of phylloquinol biosynthesis	0.0000	0.0000	0.0000	0.0000	0.1580	0.2567	0.4250	none
PWY-6147: 6-hydroxymethyl-dihydropterin diphosphate biosynthesi	0.0000	0.0000	0.0000	0.0000	-0.1570	0.2607	0.4250	none
PWY-7219: adenosine ribonucleotides de novo biosynthesis	0.0012	0.0013	0.0012	0.0012	0.1580	0.2567	0.4250	none
PWY0-1338: polymyxin resistance	0.0000	0.0000	0.0000	0.0000	-0.1570	0.2607	0.4250	none
PWY0-1533: methylphosphonate degradation I	0.0000	0.0000	0.0000	0.0000	-0.1600	0.2528	0.4250	none
UBISYN-PWY: superpathway of ubiquinol-8 biosynthesis (prokaryoti	0.0000	0.0000	0.0000	0.0000	-0.1580	0.2567	0.4250	none
PWY-5695: urate biosynthesis/inosine 5'-phosphate degradation	0.0001	0.0001	0.0001	0.0001	0.1550	0.2687	0.4354	none
AST-PWY: L-arginine degradation II (AST pathway)	0.0000	0.0000	0.0000	0.0000	-0.1460	0.2981	0.4770	none
THISYN-PWY: superpathway of thiamin diphosphate biosynthesis I	0.0001	0.0000	0.0000	0.0000	-0.1460	0.2981	0.4770	none
HISDEG-PWY: L-histidine degradation I	0.0000	0.0000	0.0000	0.0000	-0.1410	0.3114	0.4921	none
PWY-1042: glycolysis IV (plant cytosol)	0.0006	0.0007	0.0006	0.0006	0.1410	0.3114	0.4921	none
RIBOSYN2-PWY: flavin biosynthesis I (bacteria and plants)	0.0001	0.0001	0.0001	0.0000	-0.1400	0.3159	0.4931	none
SULFATE-CYS-PWY: superpathway of sulfate assimilation and cystei	0.0000	0.0000	0.0000	0.0000	-0.1400	0.3159	0.4931	none
PRPP-PWY: superpathway of histidine, purine, and pyrimidine biosy	0.0001	0.0000	0.0001	0.0000	-0.1390	0.3204	0.4971	none
PWY-6737: starch degradation V	0.0007	0.0007	0.0006	0.0007	0.1380	0.3250	0.5012	none
PWY-6507: 4-deoxy-L-threo-hex-4-enopyranuronate degradation	0.0000	0.0000	0.0000	0.0000	-0.1300	0.3534	0.5418	none
PWY-5101: L-isoleucine biosynthesis II	0.0000	0.0000	0.0000	0.0000	-0.1230	0.3783	0.5663	none
PWY-6285: superpathway of fatty acids biosynthesis (E. coli)	0.0000	0.0000	0.0000	0.0000	-0.1230	0.3783	0.5663	none
PWY-6823: molybdenum cofactor biosynthesis	0.0000	0.0000	0.0000	0.0000	-0.1250	0.3732	0.5663	none
PWY0-1479: tRNA processing	0.0000	0.0000	0.0000	0.0000	-0.1230	0.3783	0.5663	none
HEME-BIOSYNTHESIS-II: heme biosynthesis I (aerobic)	0.0000	0.0000	0.0000	0.0000	-0.1170	0.4041	0.5932	none
PWY-5855: ubiquinol-7 biosynthesis (prokaryotic)	0.0000	0.0000	0.0000	0.0000	-0.1140	0.4148	0.5932	none
PWY-5856: ubiquinol-9 biosynthesis (prokaryotic)	0.0000	0.0000	0.0000	0.0000	-0.1140	0.4148	0.5932	none
PWY-5857: ubiquinol-10 biosynthesis (prokaryotic)	0.0000	0.0000	0.0000	0.0000	-0.1140	0.4148	0.5932	none
PWY-6305: putrescine biosynthesis IV	0.0001	0.0001	0.0001	0.0001	0.1160	0.4094	0.5932	none
PWY-6527: stachyose degradation	0.0003	0.0003	0.0002	0.0003	0.1160	0.4094	0.5932	none
PWY-6708: ubiquinol-8 biosynthesis (prokaryotic)	0.0000	0.0000	0.0000	0.0000	-0.1140	0.4148	0.5932	none
PWY-6892: thiazole biosynthesis I (E. coli)	0.0000	0.0000	0.0000	0.0000	-0.1160	0.4094	0.5932	none
PWY-6124: inosine-5'-phosphate biosynthesis II	0.0000	0.0000	0.0000	0.0000	-0.1050	0.4533	0.6447	none
GALACTARDEG-PWY: D-galactarate degradation I	0.0000	0.0000	0.0000	0.0000	0.1020	0.4647	0.6450	none
GLUCARGALACTSUPER-PWY: superpathway of D-glucarate and D-gala	0.0000	0.0000	0.0000	0.0000	0.1020	0.4647	0.6450	none
P108-PWY: pyruvate fermentation to propanoate I	0.0000	0.0000	0.0000	0.0000	0.1040	0.4590	0.6450	none
PWY-4041: γ-glutamyl cycle	0.0000	0.0000	0.0000	0.0000	0.1020	0.4647	0.6450	none
PWY-6113: superpathway of mycolate biosynthesis	0.0000	0.0000	0.0000	0.0000	-0.0997	0.4762	0.6450	none
PWY-6126: superpathway of adenosine nucleotides de novo biosyn	0.0001	0.0001	0.0001	0.0001	0.0997	0.4762	0.6450	none
PWY-7220: adenosine deoxyribonucleotides de novo biosynthesis II	0.0001	0.0001	0.0000	0.0000	0.0997	0.4762	0.6450	none
PWY-7222: guanosine deoxyribonucleotides de novo biosynthesis II	0.0001	0.0001	0.0000	0.0000	0.0997	0.4762	0.6450	none
PWY-7269: NAD/NADP-NADH/NADPH mitochondrial interconversion (	0.0000	0.0000	0.0000	0.0000	-0.1010	0.4704	0.6450	none

PWY-7242: D-fructuronate degradation	0.0002	0.0002	0.0002	0.0001	0.0972	0.4879	0.6574	none
KDO-NAGLIPASYN-PWY: superpathway of (Kdo)2-lipid A biosynthesis	0.0000	0.0000	0.0000	0.0000	-0.0958	0.4938	0.6619	none
PWY0-1277: 3-phenylpropanoate and 3-(3-hydroxyphenyl)propanoat	0.0000	0.0000	0.0000	0.0000	-0.0933	0.5057	0.6743	none
PANTOSYN-PWY: pantothenate and coenzyme A biosynthesis I	0.0002	0.0002	0.0002	0.0002	0.0906	0.5178	0.6868	none
PWY-6595: superpathway of guanosine nucleotides degradation (pl	0.0000	0.0000	0.0000	0.0000	-0.0853	0.5423	0.7157	none
KETOGLUCONMET-PWY: ketogluconate metabolism	0.0000	0.0000	0.0000	0.0000	-0.0840	0.5486	0.7202	none
TCA-GLYOX-BYPASS: superpathway of glyoxylate bypass and TCA	0.0000	0.0000	0.0000	0.0000	-0.0801	0.5675	0.7412	none
PWY-7208: superpathway of pyrimidine nucleobases salvage	0.0001	0.0002	0.0001	0.0001	0.0774	0.5802	0.7540	none
CALVIN-PWY: Calvin-Benson-Bassham cycle	0.0004	0.0005	0.0004	0.0004	0.0748	0.5931	0.7644	none
FOLSYN-PWY: superpathway of tetrahydrofolate biosynthesis and s	0.0000	0.0000	0.0000	0.0000	-0.0723	0.6062	0.7644	none
GLUCOSE1PMETAB-PWY: glucose and glucose-1-phosphate degradat	0.0000	0.0000	0.0000	0.0000	-0.0723	0.6062	0.7644	none
NONMEVIPP-PWY: methylerythritol phosphate pathway I	0.0008	0.0008	0.0008	0.0007	0.0735	0.5996	0.7644	none
PWY-6606: guanosine nucleotides degradation II	0.0000	0.0000	0.0000	0.0000	-0.0723	0.6062	0.7644	none
PWY-6628: superpathway of L-phenylalanine biosynthesis	0.0001	0.0001	0.0000	0.0000	0.0723	0.6062	0.7644	none
PWY-6612: superpathway of tetrahydrofolate biosynthesis	0.0000	0.0000	0.0000	0.0000	-0.0669	0.6326	0.7939	none
FAO-PWY: fatty acid β-oxidation I	0.0000	0.0000	0.0000	0.0000	-0.0657	0.6393	0.7957	none
GALACT-GLUCUROCAT-PWY: superpathway of hexuronide and hexuro	0.0002	0.0001	0.0001	0.0001	-0.0630	0.6528	0.7957	none
ORNDEG-PWY: superpathway of ornithine degradation	0.0000	0.0000	0.0000	0.0000	-0.0630	0.6528	0.7957	none
P162-PWY: L-glutamate degradation V (via hydroxyglutarate)	0.0000	0.0000	0.0000	0.0000	0.0643	0.6460	0.7957	none
PWY-5989: stearate biosynthesis II (bacteria and plants)	0.0000	0.0000	0.0000	0.0000	0.0643	0.6460	0.7957	none
PWY-7039: phosphatidate metabolism, as a signaling molecule	0.0000	0.0000	0.0000	0.0000	0.0630	0.6528	0.7957	none
ARG+POLYAMINE-SYN: superpathway of arginine and polyamine bio	0.0001	0.0001	0.0001	0.0000	0.0604	0.6663	0.7971	none
HCAMHPDEG-PWY: 3-phenylpropanoate and 3-(3-hydroxyphenyl)pro	0.0000	0.0000	0.0000	0.0000	-0.0604	0.6663	0.7971	none
PWY-6690: cinnamate and 3-hydroxycinnamate degradation to 2-oxo	0.0000	0.0000	0.0000	0.0000	-0.0604	0.6663	0.7971	none
PWY0-162: superpathway of pyrimidine ribonucleotides de novo bi	0.0002	0.0002	0.0002	0.0001	-0.0618	0.6595	0.7971	none
1CMET2-PWY: N10-formyl-tetrahydrofolate biosynthesis	0.0004	0.0003	0.0004	0.0003	-0.0564	0.6869	0.8179	none
PEPTIDOGLYCANSYN-PWY: peptidoglycan biosynthesis I (meso-diam	0.0007	0.0007	0.0007	0.0006	0.0538	0.7007	0.8305	none
GLUCARDEG-PWY: D-glucarate degradation I	0.0000	0.0000	0.0000	0.0000	0.0525	0.7077	0.8311	none
GOLPDLCAT-PWY: superpathway of glycerol degradation to 1,3-propa	0.0000	0.0000	0.0000	0.0000	0.0525	0.7077	0.8311	none
PWY-6284: superpathway of unsaturated fatty acids biosynthesis (E.	0.0000	0.0000	0.0000	0.0000	-0.0513	0.7147	0.8354	none
DTDPRHAMSYN-PWY: dTDP-L-rhamnose biosynthesis I	0.0003	0.0004	0.0003	0.0003	0.0486	0.7287	0.8441	none
PWY-6467: Kdo transfer to lipid IVA III (Chlamydia)	0.0000	0.0000	0.0000	0.0000	-0.0486	0.7287	0.8441	none
PWY-5097: L-lysine biosynthesis VI	0.0008	0.0009	0.0008	0.0008	0.0447	0.7500	0.8571	none
PWY-7315: dTDP-N-acetylthomosamine biosynthesis	0.0000	0.0000	0.0000	0.0000	-0.0447	0.7500	0.8571	none
PWY-7446: sulfoglycolysis	0.0000	0.0000	0.0000	0.0000	0.0447	0.7500	0.8571	none
PWY-5705: allantoin degradation to glyoxylate III	0.0000	0.0000	0.0000	0.0000	-0.0433	0.7571	0.8614	none
PWY-6125: superpathway of guanosine nucleotides de novo biosynt	0.0001	0.0001	0.0001	0.0001	0.0393	0.7786	0.8780	none
PWY0-166: superpathway of pyrimidine deoxyribonucleotides de no	0.0003	0.0003	0.0003	0.0002	-0.0393	0.7786	0.8780	none

PWY-5838: superpathway of menaquinol-8 biosynthesis I	0.0000	0.0000	0.0000	0.0000	-0.0367	0.7930	0.8865	none
PWY-6901: superpathway of glucose and xylose degradation	0.0000	0.0000	0.0000	0.0000	0.0367	0.7930	0.8865	none
PWY-5791: 1,4-dihydroxy-2-naphthoate biosynthesis II (plants)	0.0000	0.0000	0.0000	0.0000	0.0354	0.8002	0.8868	none
PWY-5837: 1,4-dihydroxy-2-naphthoate biosynthesis I	0.0000	0.0000	0.0000	0.0000	0.0354	0.8002	0.8868	none
PWY-7199: pyrimidine deoxyribonucleosides salvage	0.0001	0.0001	0.0001	0.0001	0.0342	0.8075	0.8910	none
GLUCUROCAT-PWY: superpathway of β-D-glucuronide and D-gl	0.0001	0.0001	0.0001	0.0001	-0.0328	0.8147	0.8913	none
PWY0-1241: ADP-L-glycero-β-D-manno-heptose biosynthesis	0.0000	0.0000	0.0000	0.0000	-0.0328	0.8147	0.8913	none
PWY-6387: UDP-N-acetylmuramoyl-pentapeptide biosynthesis I (me	0.0007	0.0006	0.0007	0.0006	0.0315	0.8220	0.8955	none
PWY-7237: myo-, chiro- and scillo-inositol degradation	0.0000	0.0001	0.0000	0.0000	-0.0288	0.8366	0.9075	none
POLYAMSYN-PWY: superpathway of polyamine biosynthesis I	0.0001	0.0001	0.0000	0.0000	-0.0276	0.8440	0.9116	none
PWY-5861: superpathway of demethylmenaquinol-8 biosynthesis	0.0000	0.0000	0.0000	0.0000	-0.0262	0.8513	0.9157	none
GALACTUROCAT-PWY: D-galacturonate degradation I	0.0002	0.0002	0.0002	0.0002	0.0237	0.8660	0.9237	none
PWY-3841: folate transformations II	0.0006	0.0005	0.0005	0.0005	-0.0237	0.8660	0.9237	none
P461-PWY: hexitol fermentation to lactate, formate, ethanol and ac	0.0001	0.0001	0.0001	0.0000	0.0223	0.8734	0.9278	none
PENTOSE-P-PWY: pentose phosphate pathway	0.0000	0.0000	0.0000	0.0000	0.0210	0.8808	0.9317	none
COBALSYN-PWY: adenosylcobalamin salvage from cobinamide I	0.0002	0.0002	0.0002	0.0002	0.0197	0.8882	0.9319	none
PWY0-1415: superpathway of heme biosynthesis from uroporphyrin	0.0000	0.0000	0.0000	0.0000	0.0197	0.8882	0.9319	none
ENTBACSYN-PWY: enterobactin biosynthesis	0.0000	0.0000	0.0000	0.0000	-0.0183	0.8956	0.9320	none
PWY-6703: preQ0 biosynthesis	0.0000	0.0000	0.0000	0.0000	0.0183	0.8956	0.9320	none
PWY-5897: superpathway of menaquinol-11 biosynthesis	0.0000	0.0000	0.0000	0.0000	-0.0144	0.9179	0.9399	none
PWY-5898: superpathway of menaquinol-12 biosynthesis	0.0000	0.0000	0.0000	0.0000	-0.0144	0.9179	0.9399	none
PWY-5899: superpathway of menaquinol-13 biosynthesis	0.0000	0.0000	0.0000	0.0000	-0.0144	0.9179	0.9399	none
PYRIDNUCSYN-PWY: NAD biosynthesis I (from aspartate)	0.0002	0.0002	0.0001	0.0001	0.0144	0.9179	0.9399	none
PROTOCATECHUATE-ORTHO-CLEAVAGE-PWY: protocatechuate degrad	0.0000	0.0000	0.0000	0.0000	-0.0105	0.9402	0.9551	none
PWY0-1061: superpathway of L-alanine biosynthesis	0.0000	0.0000	0.0000	0.0000	0.0105	0.9402	0.9551	none
PWY-841: superpathway of purine nucleotides de novo biosynthesi	0.0001	0.0000	0.0000	0.0000	0.0092	0.9477	0.9589	none
HEMESYN2-PWY: heme biosynthesis II (anaerobic)	0.0000	0.0000	0.0000	0.0000	-0.0079	0.9551	0.9627	none
PWY-2942: L-lysine biosynthesis III	0.0008	0.0008	0.0008	0.0006	-0.0066	0.9626	0.9664	none
PWY-6282: palmitoleate biosynthesis I (from (5Z)-dodec-5-enoate)	0.0000	0.0000	0.0000	0.0000	-0.0013	0.9925	0.9925	none

EMS Table 6c: Comparison of species relative abundances between baseline and 3-month in the published glipizide group

Species	Relative abundance (mean)		Relative abundance (median)		Baseline vs. M3 (Wilcoxon signed-rank test, clr-transformed)			
	Baseline	3-month	Baseline	3-month	Effect_size	P-value	BH adjusted P-value	Enrichment

Lachnospiraceae bacterium 5 1 63FAA	0.0602	0.0798	0.0180	0.0190	0.3370	0.0271	0.8571	none
Clostridium innocuum	0.0013	0.0029	0.0000	0.0000	0.3290	0.0307	0.8571	none
Paraprevotella unclassified	0.2132	0.2890	0.0000	0.0000	-0.3100	0.0425	0.8571	none
Ruminococcus albus	0.0008	0.0020	0.0000	0.0004	0.3050	0.0450	0.8571	none
candidate division TM7 single cell isolate TM7c	0.0027	0.0018	0.0000	0.0000	-0.3000	0.0490	0.8571	none
Megamonas unclassified	2.8432	3.1510	0.0149	0.0083	-0.2940	0.0534	0.8571	none
Megasphaera unclassified	0.3270	0.1507	0.0000	0.0000	-0.2930	0.0549	0.8571	none
Roseburia unclassified	0.0063	0.0696	0.0000	0.0000	0.2910	0.0564	0.8571	none
Ruminococcus obeum	0.2668	0.4035	0.2176	0.2651	0.2740	0.0720	0.8571	none
Bacteroides plebeius	3.1696	2.8508	0.0136	0.0069	-0.2730	0.0739	0.8571	none
Bacteroides vulgatus	2.7071	2.5059	1.4512	0.7717	-0.2730	0.0739	0.8571	none
Megamonas rupellensis	0.3958	0.3960	0.0006	0.0000	-0.2610	0.0864	0.8571	none
Veillonella unclassified	0.0356	0.0895	0.0108	0.0116	0.2590	0.0886	0.8571	none
Subdoligranulum unclassified	2.2926	2.9039	1.1703	1.5024	0.2560	0.0933	0.8571	none
Anaerostipes hadrus	0.0281	0.0285	0.0054	0.0061	0.2410	0.1137	0.8571	none
Streptococcus salivarius	0.1198	0.4348	0.0622	0.0755	0.2380	0.1193	0.8571	none
Bacteroides dorei	1.8148	1.8492	0.3576	0.2102	-0.2360	0.1222	0.8571	none
Ruminococcus lactaris	0.1174	0.1165	0.0000	0.0000	-0.2360	0.1222	0.8571	none
Bacteroides finegoldii	0.4675	0.5202	0.0013	0.0114	0.2330	0.1251	0.8571	none
Lachnospiraceae bacterium 1 1 57FAA	0.3782	0.6043	0.2184	0.2665	0.2320	0.1281	0.8571	none
Barnesiella intestinihominis	0.6249	0.7118	0.0049	0.0109	0.2300	0.1312	0.8571	none
Prevotella stercorea	0.5047	0.4576	0.0000	0.0000	-0.2240	0.1407	0.8571	none
Actinomyces odontolyticus	0.0014	0.0015	0.0000	0.0000	-0.2230	0.1440	0.8571	none
Bacteroides stercoris	6.1707	6.3189	1.3916	1.2467	-0.2230	0.1440	0.8571	none
Lachnospiraceae bacterium 3 1 46FAA	0.0983	0.0535	0.0161	0.0107	-0.2150	0.1577	0.8571	none
Gemella unclassified	0.0008	0.0003	0.0000	0.0000	-0.2120	0.1649	0.8571	none
Desulfovibrio desulfuricans	0.0080	0.0062	0.0000	0.0000	0.2100	0.1687	0.8571	none
Clostridium clostridioforme	0.0173	0.0086	0.0000	0.0000	0.2070	0.1724	0.8571	none
Lachnospiraceae bacterium 1 4 56FAA	0.0630	0.1202	0.0088	0.0007	-0.2040	0.1801	0.8571	none
Parabacteroides johnsonii	0.1910	0.1776	0.0008	0.0000	-0.2040	0.1801	0.8571	none
Streptococcus anginosus	0.0235	0.0889	0.0003	0.0000	-0.2040	0.1801	0.8571	none
Coprococcus catus	0.0899	0.1158	0.0727	0.1011	0.2030	0.1841	0.8571	none
Bifidobacterium longum	0.6261	0.6545	0.0540	0.0887	0.2010	0.1881	0.8571	none
Clostridium nexile	0.0941	0.1400	0.0000	0.0000	-0.1980	0.1922	0.8571	none
Eubacterium eligens	1.5013	1.9877	0.5161	1.2360	0.1970	0.1964	0.8571	none
Megamonas funiformis	0.1976	0.0989	0.0000	0.0000	-0.1950	0.2006	0.8571	none
Roseburia hominis	0.3251	0.3656	0.1816	0.0713	-0.1940	0.2048	0.8571	none
Streptococcus australis	0.0066	0.0088	0.0021	0.0057	0.1920	0.2092	0.8571	none

Bacteroides eggerthii	2.7914	1.7200	0.0003	0.0017	-0.1890	0.2136	0.8571	none
Dorea formicigenerans	0.3629	0.3678	0.3429	0.2697	-0.1830	0.2319	0.8571	none
Bacteroides oleiciplenus	0.0041	0.0023	0.0000	0.0000	-0.1770	0.2464	0.8571	none
Parabacteroides unclassified	0.3009	0.5528	0.0000	0.0000	0.1770	0.2464	0.8571	none
Parabacteroides goldsteinii	0.0700	0.1062	0.0080	0.0127	0.1740	0.2564	0.8571	none
Ruminococcus torques	0.9366	1.1685	0.7109	0.6956	0.1740	0.2564	0.8571	none
Clostridiales bacterium 1 7 47FAA	0.0064	0.0078	0.0005	0.0015	0.1710	0.2614	0.8571	none
Klebsiella pneumoniae	0.3379	0.1843	0.0090	0.0076	-0.1690	0.2666	0.8571	none
Klebsiella unclassified	0.0018	0.0093	0.0000	0.0000	0.1690	0.2666	0.8571	none
Oxalobacter formigenes	0.0278	0.0269	0.0000	0.0000	-0.1690	0.2666	0.8571	none
Sutterella wadsworthensis	0.4890	0.3693	0.0000	0.0000	-0.1690	0.2666	0.8571	none
Cellulophaga unclassified	0.0011	0.0006	0.0000	0.0000	-0.1660	0.2771	0.8571	none
Deinococcus unclassified	0.0017	0.0021	0.0000	0.0000	0.1660	0.2771	0.8571	none
Eubacterium ventriosum	0.1177	0.1592	0.0430	0.1121	0.1660	0.2771	0.8571	none
Peptostreptococcaceae noname unclassified	0.0310	0.0279	0.0000	0.0069	0.1660	0.2771	0.8571	none
Rothia mucilaginosa	0.0076	0.0124	0.0008	0.0026	0.1660	0.2771	0.8571	none
Faecalibacterium prausnitzii	7.3430	6.8672	5.5074	6.1103	0.1600	0.2935	0.8811	none
Erysipelotrichaceae bacterium 21 3	0.0046	0.0076	0.0000	0.0000	0.1520	0.3162	0.8811	none
Roseburia intestinalis	0.9841	0.9621	0.2525	0.2647	-0.1510	0.3221	0.8811	none
Paraprevotella clara	0.0921	0.1111	0.0000	0.0000	-0.1490	0.3280	0.8811	none
Coprobacter fastidiosus	0.0135	0.0218	0.0000	0.0000	0.1470	0.3340	0.8811	none
Eubacterium hallii	0.3166	0.3828	0.2459	0.2101	0.1470	0.3340	0.8811	none
Clostridium leptum	0.0467	0.2271	0.0113	0.0135	0.1450	0.3401	0.8811	none
Roseburia inulinivorans	1.4725	0.9102	0.7396	0.6718	-0.1450	0.3401	0.8811	none
Veillonella atypica	0.0066	0.0110	0.0000	0.0000	0.1450	0.3401	0.8811	none
Fusobacterium mortiferum	0.2098	0.3639	0.0000	0.0000	-0.1440	0.3463	0.8811	none
Megamonas hypermegale	0.7491	0.4982	0.0000	0.0000	-0.1440	0.3463	0.8811	none
Burkholderiales bacterium 1 1 47	0.1460	0.1276	0.0146	0.0154	-0.1420	0.3525	0.8811	none
Bilophila wadsworthia	0.0707	0.0547	0.0478	0.0347	-0.1400	0.3588	0.8811	none
Erysipelotrichaceae bacterium 6 1 45	0.0054	0.0038	0.0000	0.0003	0.1400	0.3588	0.8811	none
Clostridium bolteae	0.1219	0.0852	0.0412	0.0373	-0.1340	0.3781	0.9075	none
Veillonella parvula	0.0379	0.0349	0.0054	0.0069	0.1330	0.3846	0.9075	none
Oscillibacter unclassified	0.5733	0.5313	0.2518	0.3785	0.1310	0.3913	0.9075	none
Streptococcus sanguinis	0.0058	0.0048	0.0018	0.0000	-0.1310	0.3913	0.9075	none
Adlercreutzia equolifaciens	0.1241	0.1065	0.0409	0.0341	-0.1270	0.4047	0.9134	none
Ruminococcus sp 5 1 39BFAA	0.6845	0.5862	0.5316	0.4846	-0.1270	0.4047	0.9134	none
Eubacterium rectale	10.8016	10.3705	5.6463	6.1707	-0.1250	0.4116	0.9165	none
Anaerotruncus colihominis	0.0326	0.0301	0.0040	0.0090	0.1230	0.4185	0.9196	none

Solobacterium moorei	0.0018	0.0014	0.0000	0.0000	-0.1200	0.4325	0.9261	none
Streptococcus gordonii	0.0021	0.0037	0.0000	0.0000	-0.1200	0.4325	0.9261	none
Eggerthella unclassified	0.0611	0.0569	0.0105	0.0241	0.1100	0.4688	0.9737	none
Subdoligranulum variabile	0.0016	0.0017	0.0001	0.0007	0.1050	0.4913	0.9737	none
Escherichia coli	1.8384	1.6219	0.1935	0.1426	0.1010	0.5066	0.9737	none
Prevotella copri	9.2113	8.9492	0.0193	0.0189	0.1010	0.5066	0.9737	none
Streptococcus infantis	0.0041	0.0067	0.0009	0.0000	-0.1010	0.5066	0.9737	none
Alistipes shahii	0.9078	0.8782	0.3944	0.5803	0.0958	0.5301	0.9737	none
Bifidobacterium adolescentis	0.3946	0.7036	0.0006	0.0000	0.0958	0.5301	0.9737	none
Clostridium symbiosum	0.0176	0.0221	0.0022	0.0045	0.0958	0.5301	0.9737	none
Bacteroides uniformis	1.8555	1.6757	0.5886	0.5148	-0.0939	0.5380	0.9737	none
Streptococcus mitis oralis pneumoniae	0.0113	0.0089	0.0030	0.0030	-0.0939	0.5380	0.9737	none
Ruminococcaceae bacterium D16	0.0059	0.0059	0.0000	0.0000	-0.0921	0.5460	0.9737	none
Alistipes indistinctus	0.0568	0.1344	0.0143	0.0084	-0.0884	0.5622	0.9737	none
Dialister invisus	0.0227	0.1190	0.0000	0.0000	0.0884	0.5622	0.9737	none
Ruminococcus callidus	0.1891	0.2205	0.0114	0.0565	0.0884	0.5622	0.9737	none
Alistipes sp AP11	0.1190	0.1306	0.0000	0.0000	-0.0846	0.5786	0.9737	none
Clostridium bartlettii	0.0356	0.0364	0.0114	0.0115	-0.0846	0.5786	0.9737	none
Lachnospiraceae bacterium 8 1 57FAA	0.1007	0.0516	0.0000	0.0000	0.0828	0.5869	0.9737	none
Butyrivibrio unclassified	0.0099	0.0106	0.0090	0.0093	0.0810	0.5952	0.9737	none
Lachnospiraceae bacterium 7 1 58FAA	0.0177	0.0266	0.0069	0.0053	-0.0791	0.6036	0.9737	none
Bacteroidales bacterium ph8	0.4074	0.5603	0.1289	0.1199	-0.0773	0.6121	0.9737	none
Dasheen mosaic virus	0.0004	0.0008	0.0000	0.0000	0.0773	0.6121	0.9737	none
Bacteroides nordii	0.0280	0.0210	0.0103	0.0097	-0.0755	0.6205	0.9737	none
Streptococcus vestibularis	0.0039	0.0050	0.0000	0.0000	-0.0755	0.6205	0.9737	none
Bifidobacterium pseudocatenulatum	0.6567	0.4478	0.0151	0.0284	0.0737	0.6291	0.9737	none
Alistipes putredinis	3.6967	3.5248	2.4796	2.6544	-0.0718	0.6377	0.9737	none
Paraprevotella xylaniphila	0.1251	0.2594	0.0000	0.0000	-0.0718	0.6377	0.9737	none
Akkermansia muciniphila	0.5562	1.0314	0.0000	0.0000	-0.0700	0.6463	0.9737	none
Bacteroides fragilis	1.1999	1.5440	0.1262	0.0916	-0.0700	0.6463	0.9737	none
Clostridiaceae bacterium JC118	0.0020	0.0019	0.0000	0.0000	-0.0682	0.6550	0.9737	none
Holdemania filiformis	0.0285	0.0273	0.0182	0.0150	-0.0682	0.6550	0.9737	none
Candidatus Zinderia insecticola	0.0005	0.0008	0.0003	0.0002	0.0663	0.6638	0.9737	none
Bacteroides clarus	0.0376	0.0368	0.0006	0.0000	-0.0645	0.6726	0.9737	none
Pedobacter unclassified	0.0033	0.0032	0.0000	0.0000	-0.0645	0.6726	0.9737	none
Clostridium asparagiforme	0.0151	0.0181	0.0069	0.0066	0.0627	0.6814	0.9737	none
Eubacterium biforme	0.2597	0.3686	0.0000	0.0000	0.0627	0.6814	0.9737	none
Alistipes onderdonkii	0.7666	0.6164	0.1286	0.1309	-0.0607	0.6903	0.9737	none

Holdemania unclassified	0.0020	0.0025	0.0000	0.0000	0.0607	0.6903	0.9737	none
Odoribacter splanchnicus	0.6213	0.5918	0.5215	0.4698	-0.0589	0.6992	0.9737	none
Parabacteroides merdae	1.0740	0.9382	0.7337	0.8351	-0.0589	0.6992	0.9737	none
Bacteroides coprocola	1.2790	1.1491	0.0006	0.0006	-0.0570	0.7082	0.9737	none
Collinsella aerofaciens	0.4283	0.5242	0.2070	0.1961	0.0570	0.7082	0.9737	none
Dorea longicatena	0.2797	0.3396	0.1654	0.2039	0.0570	0.7082	0.9737	none
Parasutterella excrementihominis	0.1511	0.1463	0.0061	0.0068	-0.0570	0.7082	0.9737	none
Ruminococcus bromii	2.0222	2.9738	0.0188	0.8685	0.0552	0.7172	0.9737	none
Ruminococcus flavefaciens	0.0016	0.0018	0.0000	0.0000	0.0552	0.7172	0.9737	none
Clostridium ramosum	0.0131	0.0076	0.0000	0.0000	-0.0515	0.7353	0.9739	none
Bacteroides caccae	1.6693	1.5731	0.7308	0.6407	0.0497	0.7444	0.9739	none
Bacteroides ovatus	1.4029	1.4217	0.2675	0.3600	-0.0479	0.7536	0.9739	none
Clostridium hathewayi	0.0286	0.0332	0.0044	0.0045	0.0479	0.7536	0.9739	none
Coprobacillus unclassified	0.0321	0.0431	0.0005	0.0000	-0.0479	0.7536	0.9739	none
Gemella sanguinis	0.0014	0.0013	0.0000	0.0000	-0.0479	0.7536	0.9739	none
Eubacterium infirmum	0.0004	0.0004	0.0000	0.0000	-0.0461	0.7627	0.9739	none
Bacteroides salyersiae	0.3099	0.2581	0.0000	0.0000	-0.0442	0.7720	0.9739	none
Bacteroides intestinalis	0.2059	0.4527	0.0753	0.0263	-0.0424	0.7812	0.9739	none
Gordonibacter pamelaeae	0.0242	0.0229	0.0045	0.0039	0.0406	0.7905	0.9739	none
Bilophila unclassified	0.5601	0.4842	0.5086	0.4470	-0.0387	0.7998	0.9739	none
Granulicatella unclassified	0.0049	0.0044	0.0029	0.0030	-0.0387	0.7998	0.9739	none
Eggerthella lenta	0.0057	0.0082	0.0000	0.0002	-0.0368	0.8092	0.9739	none
Bacteroides thetaiotaomicron	0.7613	0.8554	0.4256	0.4033	0.0349	0.8185	0.9739	none
Granulicatella adiacens	0.0020	0.0018	0.0000	0.0000	-0.0349	0.8185	0.9739	none
Olsenella unclassified	0.0006	0.0008	0.0000	0.0000	-0.0349	0.8185	0.9739	none
Streptococcus parasanguinis	0.0481	0.1223	0.0083	0.0174	0.0331	0.8279	0.9739	none
Anaerotruncus unclassified	0.0126	0.0109	0.0026	0.0039	0.0313	0.8374	0.9739	none
Eubacterium ramulus	0.1017	0.0844	0.0764	0.0529	-0.0313	0.8374	0.9739	none
Streptococcus cristatus	0.0010	0.0016	0.0000	0.0000	0.0313	0.8374	0.9739	none
Ruminococcus gnavus	0.3505	0.5805	0.0534	0.0419	-0.0294	0.8468	0.9739	none
Lachnospiraceae bacterium 3 1 57FAA CT1	0.0054	0.0093	0.0000	0.0000	-0.0276	0.8563	0.9739	none
Peptostreptococcus unclassified	0.0004	0.0005	0.0000	0.0000	-0.0258	0.8658	0.9739	none
Bacteroides cellulosilyticus	0.3200	0.5206	0.0521	0.0170	0.0258	0.8658	0.9739	none
Parabacteroides distasonis	0.8546	0.7770	0.4346	0.3121	0.0258	0.8658	0.9739	none
Bacteroides massiliensis	1.4899	1.7125	0.0111	0.0067	-0.0239	0.8753	0.9739	none
Bacteroides xylanisolvens	0.2370	0.2251	0.1140	0.0866	-0.0221	0.8848	0.9739	none
Erysipelotrichaceae bacterium 2 2 44A	0.0020	0.0013	0.0000	0.0000	0.0203	0.8943	0.9739	none
Haemophilus parainfluenzae	0.1659	0.1695	0.0033	0.0115	0.0203	0.8943	0.9739	none

Pseudoflavonifractor capillosus	0.0003	0.0013	0.0000	0.0000	0.0203	0.8943	0.9739	none
Bacteroides faecis	0.0281	0.0319	0.0019	0.0017	0.0185	0.9039	0.9739	none
Flavonifractor plautii	0.0489	0.0537	0.0161	0.0128	-0.0185	0.9039	0.9739	none
Alistipes senegalensis	0.0320	0.0290	0.0015	0.0021	0.0147	0.9230	0.9756	none
Coprococcus comes	0.5123	0.4545	0.2760	0.3315	-0.0147	0.9230	0.9756	none
Enterobacter cloacae	0.0096	0.0390	0.0000	0.0000	-0.0147	0.9230	0.9756	none
Clostridium citroniae	0.0250	0.0272	0.0164	0.0146	-0.0129	0.9326	0.9796	none
Lactobacillus mucosae	0.0001	0.0003	0.0000	0.0000	-0.0110	0.9422	0.9835	none
Mitsuokella unclassified	0.0105	0.0081	0.0039	0.0040	0.0092	0.9519	0.9873	none
Butyricimonas synergistica	0.0155	0.0120	0.0000	0.0000	-0.0074	0.9615	0.9912	none
Eubacterium siraeum	0.6549	0.3921	0.0000	0.0000	-0.0055	0.9711	0.9949	none
Alistipes finegoldii	0.1242	0.1250	0.0275	0.0201	0.0037	0.9807	0.9963	none
Lachnospiraceae bacterium 2 1 58FAA	0.0329	0.0367	0.0024	0.0027	-0.0019	0.9904	0.9963	none
Veillonella dispar	0.0026	0.0041	0.0000	0.0000	0.0019	0.9904	0.9963	none
Escherichia unclassified	0.3457	0.2652	0.0318	0.0371	0.0000	1.0000	1.0000	none

EMS Table 6d: Comparison of pathway relative abundances between baseline and 3-month in the published glipizide group

Pathway	Relative abundance (mean)		Relative abundance (median)		Baseline vs. M3 (Wilcoxon signed-rank test, clr-transformed)			
	Baseline	3-month	Baseline	3-month	Effect_size	P-value	BH adjusted P-value	Enrichment
1CMET2-PWY: N10-formyl-tetrahydrofolate biosynthesis	0.0004	0.0004	0.0004	0.0004	-0.0917	0.5526	0.9889	none
AEROBACTINSYN-PWY: aerobactin biosynthesis	0.0000	0.0000	0.0000	0.0000	-0.1360	0.3780	0.9889	none
ALL-CHORISMATE-PWY: superpathway of chorismate metabolism	0.0000	0.0000	0.0000	0.0000	0.3750	0.0150	0.6632	none
ANAEROFRUCAT-PWY: homolactic fermentation	0.0002	0.0001	0.0001	0.0001	-0.2300	0.1351	0.9889	none
ANAGLYCOLYSIS-PWY: glycolysis III (from glucose)	0.0005	0.0005	0.0005	0.0005	-0.0647	0.6753	0.9889	none
ARG+POLYAMINE-SYN: superpathway of arginine and polyamine biosynthesis	0.0001	0.0001	0.0001	0.0000	0.0511	0.7404	0.9889	none
ARGININE-SYN4-PWY: L-ornithine de novo biosynthesis	0.0000	0.0000	0.0000	0.0000	-0.1360	0.3780	0.9889	none
ARGORNPROST-PWY: arginine, ornithine and proline interconversion	0.0000	0.0000	0.0000	0.0000	-0.0974	0.5278	0.9889	none
ARGSYN-PWY: L-arginine biosynthesis I (via L-ornithine)	0.0001	0.0001	0.0001	0.0001	-0.0839	0.5865	0.9889	none
ARGSYNBSUB-PWY: L-arginine biosynthesis II (acetyl cycle)	0.0001	0.0001	0.0000	0.0001	-0.0492	0.7498	0.9889	none
ARO-PWY: chorismate biosynthesis I	0.0006	0.0006	0.0006	0.0006	-0.0184	0.9054	0.9889	none
ASPASN-PWY: superpathway of L-aspartate and L-asparagine biosynthesis	0.0002	0.0002	0.0002	0.0001	-0.0299	0.8463	0.9889	none
AST-PWY: L-arginine degradation II (AST pathway)	0.0000	0.0000	0.0000	0.0000	-0.0608	0.6937	0.9889	none
BIOTIN-BIOSYNTHESIS-PWY: biotin biosynthesis I	0.0000	0.0000	0.0000	0.0000	0.0377	0.8074	0.9889	none

BRANCHED-CHAIN-AA-SYN-PWY: superpathway of branched amino acid biosynthesis	0.0004	0.0004	0.0004	0.0004	0.0068	0.9651	0.9889	none
CALVIN-PWY: Calvin-Benson-Bassham cycle	0.0005	0.0005	0.0005	0.0005	-0.0029	0.9850	0.9889	none
CITRULBIO-PWY: L-citrulline biosynthesis	0.0001	0.0001	0.0000	0.0000	-0.0048	0.9751	0.9889	none
COA-PWY-1: coenzyme A biosynthesis II (mammalian)	0.0002	0.0002	0.0001	0.0001	0.0010	0.9950	0.9950	none
COA-PWY: coenzyme A biosynthesis I	0.0001	0.0001	0.0001	0.0001	0.0029	0.9850	0.9889	none
COBALSYN-PWY: adenosylcobalamin salvage from cobinamide I	0.0002	0.0002	0.0002	0.0002	-0.0184	0.9054	0.9889	none
COLANSYN-PWY: colanic acid building blocks biosynthesis	0.0000	0.0000	0.0000	0.0000	-0.0415	0.7881	0.9889	none
COMPLETE-ARO-PWY: superpathway of aromatic amino acid biosynthesis	0.0006	0.0006	0.0006	0.0006	-0.0145	0.9253	0.9889	none
DAPLYSINESYN-PWY: L-lysine biosynthesis I	0.0000	0.0001	0.0000	0.0000	0.2130	0.1671	0.9889	none
DENOVOPURINE2-PWY: superpathway of purine nucleotides de novo biosynthesis II	0.0001	0.0001	0.0001	0.0001	-0.0742	0.6302	0.9889	none
DTDPRHAMSYN-PWY: dTDP-L-rhamnose biosynthesis I	0.0004	0.0003	0.0003	0.0003	-0.0974	0.5278	0.9889	none
ENTBACSYN-PWY: enterobactin biosynthesis	0.0000	0.0000	0.0000	0.0000	0.0647	0.6753	0.9889	none
FAO-PWY: fatty acid β-oxidation I	0.0000	0.0000	0.0000	0.0000	0.0858	0.5779	0.9889	none
FASYN-ELONG-PWY: fatty acid elongation -- saturated	0.0001	0.0000	0.0000	0.0000	-0.1230	0.4272	0.9889	none
FERMENTATION-PWY: mixed acid fermentation	0.0000	0.0000	0.0000	0.0000	0.1640	0.2907	0.9889	none
FOLSYN-PWY: superpathway of tetrahydrofolate biosynthesis and salvage	0.0000	0.0000	0.0000	0.0000	-0.0048	0.9751	0.9889	none
FUC-RHAMCAT-PWY: superpathway of fucose and rhamnose degradation	0.0000	0.0000	0.0000	0.0000	-0.0145	0.9253	0.9889	none
FUCCAT-PWY: fucose degradation	0.0000	0.0000	0.0000	0.0000	-0.0145	0.9253	0.9889	none
GALACT-GLUCUROCAT-PWY: superpathway of hexuronide and hexuronate degradation	0.0001	0.0001	0.0001	0.0001	-0.0454	0.7689	0.9889	none
GALACTARDEG-PWY: D-galactarate degradation I	0.0000	0.0000	0.0000	0.0000	0.1820	0.2374	0.9889	none
GALACTUROCAT-PWY: D-galacturonate degradation I	0.0002	0.0002	0.0001	0.0002	-0.0164	0.9154	0.9889	none
GLCMANNANAUT-PWY: superpathway of N-acetylglucosamine, N-acetylmannosamine	0.0001	0.0001	0.0001	0.0001	0.1280	0.4057	0.9889	none
GLUCARDEG-PWY: D-glucarate degradation I	0.0000	0.0000	0.0000	0.0000	0.1360	0.3780	0.9889	none
GLUCARGALACTSUPER-PWY: superpathway of D-glucarate and D-galactarate degradati	0.0000	0.0000	0.0000	0.0000	0.1820	0.2374	0.9889	none
GLUCONEO-PWY: gluconeogenesis I	0.0001	0.0001	0.0001	0.0001	-0.0781	0.6126	0.9889	none
GLUCOSE1PMETAB-PWY: glucose and glucose-1-phosphate degradation	0.0000	0.0000	0.0000	0.0000	-0.0917	0.5526	0.9889	none
GLUCUROCAT-PWY: superpathway of β-D-glucuronide and D-glucuronate degradati	0.0001	0.0001	0.0001	0.0001	-0.0356	0.8171	0.9889	none
GLUTORN-PWY: L-ornithine biosynthesis	0.0001	0.0001	0.0000	0.0000	-0.0318	0.8365	0.9889	none
GLYCOCAT-PWY: glycogen degradation I (bacterial)	0.0000	0.0000	0.0000	0.0000	-0.0202	0.8955	0.9889	none
GLYCOGENSYNTH-PWY: glycogen biosynthesis I (from ADP-D-Glucose)	0.0000	0.0000	0.0000	0.0000	-0.0145	0.9253	0.9889	none
GLYCOLYSIS-E-D: superpathway of glycolysis and Entner-Doudoroff	0.0000	0.0000	0.0000	0.0000	-0.2020	0.1913	0.9889	none
GLYCOLYSIS-TCA-GLYOX-BYPASS: superpathway of glycolysis, pyruvate dehydrogen	0.0000	0.0000	0.0000	0.0000	-0.0935	0.5442	0.9889	none
GLYCOLYSIS: glycolysis I (from glucose 6-phosphate)	0.0001	0.0001	0.0001	0.0001	-0.2190	0.1559	0.9889	none
GLYOXYLATE-BYPASS: glyoxylate cycle	0.0000	0.0000	0.0000	0.0000	0.0647	0.6753	0.9889	none
GOLPDLCAT-PWY: superpathway of glycerol degradation to 1,3-propanediol	0.0000	0.0000	0.0000	0.0000	-0.1980	0.2000	0.9889	none
HCAMHPDEG-PWY: 3-phenylpropanoate and 3-(3-hydroxyphenyl)propanoate degradatio	0.0000	0.0000	0.0000	0.0000	0.0492	0.7498	0.9889	none
HEME-BIOSYNTHESIS-II: heme biosynthesis I (aerobic)	0.0000	0.0000	0.0000	0.0000	0.1420	0.3581	0.9889	none
HEMESYN2-PWY: heme biosynthesis II (anaerobic)	0.0000	0.0000	0.0000	0.0000	-0.1790	0.2474	0.9889	none

HEXITOLDEGSUPER-PWY: superpathway of hexitol degradation (bacteria)	0.0001	0.0000	0.0001	0.0000	-0.3180	0.0397	0.9889	none
HISDEG-PWY: L-histidine degradation I	0.0000	0.0000	0.0000	0.0000	0.1010	0.5115	0.9889	none
HISTSYN-PWY: L-histidine biosynthesis	0.0002	0.0002	0.0002	0.0001	0.0048	0.9751	0.9889	none
HOMOSER-METSYN-PWY: L-methionine biosynthesis I	0.0001	0.0001	0.0001	0.0001	0.0801	0.6038	0.9889	none
HSERMETANA-PWY: L-methionine biosynthesis III	0.0001	0.0001	0.0001	0.0001	-0.0917	0.5526	0.9889	none
ILEUSYN-PWY: L-isoleucine biosynthesis I (from threonine)	0.0004	0.0004	0.0004	0.0004	0.0048	0.9751	0.9889	none
KDO-NAGLIPASYN-PWY: superpathway of (Kdo)2-lipid A biosynthesis	0.0000	0.0000	0.0000	0.0000	0.1460	0.3452	0.9889	none
KETOGLUCONMET-PWY: ketogluconate metabolism	0.0000	0.0000	0.0000	0.0000	0.1300	0.3987	0.9889	none
LACTOSECAT-PWY: lactose and galactose degradation I	0.0000	0.0000	0.0000	0.0000	0.0184	0.9054	0.9889	none
LPSSYN-PWY: superpathway of lipopolysaccharide biosynthesis	0.0000	0.0000	0.0000	0.0000	-0.1240	0.4200	0.9889	none
MET-SAM-PWY: superpathway of S-adenosyl-L-methionine biosynthesis	0.0001	0.0001	0.0001	0.0001	0.0048	0.9751	0.9889	none
METHGLYUT-PWY: superpathway of methylglyoxal degradation	0.0000	0.0000	0.0000	0.0000	-0.0858	0.5779	0.9889	none
METSYN-PWY: L-homoserine and L-methionine biosynthesis	0.0001	0.0001	0.0001	0.0001	0.0647	0.6753	0.9889	none
NAD-BIOSYNTHESIS-II: NAD salvage pathway II	0.0000	0.0000	0.0000	0.0000	0.1820	0.2374	0.9889	none
NAGLIPASYN-PWY: lipid IVA biosynthesis	0.0000	0.0000	0.0000	0.0000	-0.0202	0.8955	0.9889	none
NONMEVIPP-PWY: methylerythritol phosphate pathway I	0.0008	0.0007	0.0008	0.0007	-0.0454	0.7689	0.9889	none
NONOXIPENT-PWY: pentose phosphate pathway (non-oxidative branch)	0.0004	0.0004	0.0004	0.0004	-0.0241	0.8758	0.9889	none
OANTIGEN-PWY: O-antigen building blocks biosynthesis (E. coli)	0.0001	0.0001	0.0001	0.0001	-0.0724	0.6392	0.9889	none
ORNDEG-PWY: superpathway of ornithine degradation	0.0000	0.0000	0.0000	0.0000	0.2590	0.0926	0.9889	none
P105-PWY: TCA cycle IV (2-oxoglutarate decarboxylase)	0.0000	0.0000	0.0000	0.0000	0.0801	0.6038	0.9889	none
P108-PWY: pyruvate fermentation to propanoate I	0.0000	0.0000	0.0000	0.0000	0.0685	0.6571	0.9889	none
P122-PWY: heterolactic fermentation	0.0000	0.0000	0.0000	0.0000	0.1170	0.4494	0.9889	none
P124-PWY: Bifidobacterium shunt	0.0000	0.0000	0.0000	0.0000	0.1010	0.5115	0.9889	none
P162-PWY: L-glutamate degradation V (via hydroxyglutarate)	0.0000	0.0000	0.0000	0.0000	-0.1940	0.2089	0.9889	none
P164-PWY: purine nucleobases degradation I (anaerobic)	0.0000	0.0000	0.0000	0.0000	0.0472	0.7593	0.9889	none
P4-PWY: superpathway of L-lysine, L-threonine and L-methionine biosynthesis I	0.0001	0.0001	0.0000	0.0000	0.2140	0.1633	0.9889	none
P42-PWY: incomplete reductive TCA cycle	0.0000	0.0000	0.0000	0.0000	0.2530	0.1001	0.9889	none
P441-PWY: superpathway of N-acetylneuraminate degradation	0.0001	0.0000	0.0000	0.0000	-0.2040	0.1871	0.9889	none
P461-PWY: hexitol fermentation to lactate, formate, ethanol and acetate	0.0001	0.0001	0.0000	0.0000	-0.1530	0.3202	0.9889	none
PANTO-PWY: phosphopantothenate biosynthesis I	0.0007	0.0006	0.0006	0.0006	-0.0685	0.6571	0.9889	none
PANTOSYN-PWY: pantothenate and coenzyme A biosynthesis I	0.0002	0.0002	0.0002	0.0002	0.0087	0.9551	0.9889	none
PENTOSE-P-PWY: pentose phosphate pathway	0.0000	0.0000	0.0000	0.0000	0.2100	0.1749	0.9889	none
PEPTIDOGLYCANSYN-PWY: peptidoglycan biosynthesis I (meso-diaminopimelate contai	0.0007	0.0007	0.0007	0.0006	-0.0492	0.7498	0.9889	none
PHOSLIPSYN-PWY: superpathway of phospholipid biosynthesis I (bacteria)	0.0000	0.0000	0.0000	0.0000	0.0762	0.6214	0.9889	none
POLYAMSYN-PWY: superpathway of polyamine biosynthesis I	0.0001	0.0001	0.0000	0.0000	0.0917	0.5526	0.9889	none
POLYISOPRENSYN-PWY: polyisoprenoid biosynthesis (E. coli)	0.0000	0.0000	0.0000	0.0000	0.1740	0.2578	0.9889	none
PPGPPMET-PWY: ppGpp biosynthesis	0.0000	0.0000	0.0000	0.0000	0.1760	0.2526	0.9889	none
PROTOCATECHUATE-ORTHO-CLEAVAGE-PWY: protocatechuate degradation II (orth	0.0000	0.0000	0.0000	0.0000	-0.1170	0.4494	0.9889	none

PRPP-PWY: superpathway of histidine, purine, and pyrimidine biosynthesis	0.0001	0.0001	0.0001	0.0001	-0.0492	0.7498	0.9889	none
PWY-1042: glycolysis IV (plant cytosol)	0.0006	0.0006	0.0006	0.0006	-0.0048	0.9751	0.9889	none
PWY-1269: CMP-3-deoxy-D-manno-octulosonate biosynthesis I	0.0001	0.0001	0.0000	0.0000	-0.1670	0.2794	0.9889	none
PWY-1861: formaldehyde assimilation II (RuMP Cycle)	0.0000	0.0000	0.0000	0.0000	-0.0048	0.9751	0.9889	none
PWY-2941: L-lysine biosynthesis II	0.0000	0.0000	0.0000	0.0000	-0.0994	0.5196	0.9889	none
PWY-2942: L-lysine biosynthesis III	0.0008	0.0008	0.0007	0.0007	-0.0608	0.6937	0.9889	none
PWY-3001: superpathway of L-isoleucine biosynthesis I	0.0003	0.0003	0.0003	0.0003	-0.0087	0.9551	0.9889	none
PWY-3841: folate transformations II	0.0006	0.0005	0.0006	0.0005	-0.0569	0.7122	0.9889	none
PWY-4041: γ-glutamyl cycle	0.0000	0.0000	0.0000	0.0000	0.1670	0.2794	0.9889	none
PWY-4981: L-proline biosynthesis II (from arginine)	0.0000	0.0000	0.0000	0.0000	-0.1380	0.3713	0.9889	none
PWY-5022: 4-aminobutanoate degradation V	0.0000	0.0000	0.0000	0.0000	-0.2210	0.1522	0.9889	none
PWY-5030: L-histidine degradation III	0.0000	0.0000	0.0000	0.0000	-0.2270	0.1418	0.9889	none
PWY-5097: L-lysine biosynthesis VI	0.0008	0.0008	0.0008	0.0008	-0.0549	0.7216	0.9889	none
PWY-5100: pyruvate fermentation to acetate and lactate II	0.0001	0.0001	0.0001	0.0000	0.0029	0.9850	0.9889	none
PWY-5101: L-isoleucine biosynthesis II	0.0000	0.0000	0.0000	0.0000	0.1280	0.4057	0.9889	none
PWY-5103: L-isoleucine biosynthesis III	0.0000	0.0000	0.0000	0.0000	-0.2750	0.0748	0.9889	none
PWY-5121: superpathway of geranylgeranyl diphosphate biosynthesis II (via MEP)	0.0000	0.0000	0.0000	0.0000	0.2130	0.1671	0.9889	none
PWY-5138: unsaturated, even numbered fatty acid β-oxidation	0.0000	0.0000	0.0000	0.0000	0.1560	0.3142	0.9889	none
PWY-5154: L-arginine biosynthesis III (via N-acetyl-L-citrulline)	0.0001	0.0001	0.0000	0.0001	-0.0801	0.6038	0.9889	none
PWY-5188: tetrapyrrole biosynthesis I (from glutamate)	0.0000	0.0000	0.0000	0.0000	0.1640	0.2907	0.9889	none
PWY-5345: superpathway of L-methionine biosynthesis (by sulfhydrylation)	0.0000	0.0000	0.0000	0.0000	-0.0145	0.9253	0.9889	none
PWY-5347: superpathway of L-methionine biosynthesis (transsulfuration)	0.0001	0.0001	0.0001	0.0001	0.0338	0.8268	0.9889	none
PWY-5384: sucrose degradation IV (sucrose phosphorylase)	0.0000	0.0000	0.0000	0.0000	-0.2610	0.0902	0.9889	none
PWY-5484: glycolysis II (from fructose 6-phosphate)	0.0001	0.0001	0.0001	0.0001	-0.2180	0.1595	0.9889	none
PWY-5659: GDP-mannose biosynthesis	0.0001	0.0001	0.0001	0.0001	-0.0955	0.5360	0.9889	none
PWY-5667: CDP-diacylglycerol biosynthesis I	0.0000	0.0000	0.0000	0.0000	0.0878	0.5694	0.9889	none
PWY-5676: acetyl-CoA fermentation to butanoate II	0.0000	0.0000	0.0000	0.0000	0.0261	0.8660	0.9889	none
PWY-5686: UMP biosynthesis	0.0003	0.0003	0.0003	0.0002	-0.0858	0.5779	0.9889	none
PWY-5690: TCA cycle II (plants and fungi)	0.0000	0.0000	0.0000	0.0000	-0.1380	0.3713	0.9889	none
PWY-5695: urate biosynthesis/inosine 5'-phosphate degradation	0.0001	0.0001	0.0001	0.0001	-0.0858	0.5779	0.9889	none
PWY-5705: allantoin degradation to glyoxylate III	0.0000	0.0000	0.0000	0.0000	0.2380	0.1225	0.9889	none
PWY-5791: 1,4-dihydroxy-2-naphthoate biosynthesis II (plants)	0.0000	0.0000	0.0000	0.0000	0.1810	0.2424	0.9889	none
PWY-5837: 1,4-dihydroxy-2-naphthoate biosynthesis I	0.0000	0.0000	0.0000	0.0000	0.1810	0.2424	0.9889	none
PWY-5838: superpathway of menaquinol-8 biosynthesis I	0.0000	0.0000	0.0000	0.0000	0.1190	0.4419	0.9889	none
PWY-5840: superpathway of menaquinol-7 biosynthesis	0.0000	0.0000	0.0000	0.0000	0.0299	0.8463	0.9889	none
PWY-5855: ubiquinol-7 biosynthesis (prokaryotic)	0.0000	0.0000	0.0000	0.0000	0.3730	0.0155	0.6632	none
PWY-5856: ubiquinol-9 biosynthesis (prokaryotic)	0.0000	0.0000	0.0000	0.0000	0.3730	0.0155	0.6632	none
PWY-5857: ubiquinol-10 biosynthesis (prokaryotic)	0.0000	0.0000	0.0000	0.0000	0.3730	0.0155	0.6632	none

PWY-5861: superpathway of demethylmenaquinol-8 biosynthesis	0.0000	0.0000	0.0000	0.0000	0.1170	0.4494	0.9889	none
PWY-5863: superpathway of phylloquinol biosynthesis	0.0000	0.0000	0.0000	0.0000	0.2920	0.0582	0.9889	none
PWY-5897: superpathway of menaquinol-11 biosynthesis	0.0000	0.0000	0.0000	0.0000	0.1790	0.2474	0.9889	none
PWY-5898: superpathway of menaquinol-12 biosynthesis	0.0000	0.0000	0.0000	0.0000	0.1790	0.2474	0.9889	none
PWY-5899: superpathway of menaquinol-13 biosynthesis	0.0000	0.0000	0.0000	0.0000	0.1790	0.2474	0.9889	none
PWY-5913: TCA cycle VI (obligate autotrophs)	0.0000	0.0000	0.0000	0.0000	0.1600	0.2965	0.9889	none
PWY-5918: superpathay of heme biosynthesis from glutamate	0.0000	0.0000	0.0000	0.0000	0.1480	0.3388	0.9889	none
PWY-5971: palmitate biosynthesis II (bacteria and plants)	0.0001	0.0000	0.0000	0.0000	-0.0569	0.7122	0.9889	none
PWY-5973: cis-vaccenate biosynthesis	0.0002	0.0002	0.0002	0.0001	-0.1710	0.2685	0.9889	none
PWY-5989: stearate biosynthesis II (bacteria and plants)	0.0000	0.0000	0.0000	0.0000	-0.1870	0.2276	0.9889	none
PWY-6113: superpathway of mycolate biosynthesis	0.0000	0.0000	0.0000	0.0000	-0.0974	0.5278	0.9889	none
PWY-6121: 5-aminoimidazole ribonucleotide biosynthesis I	0.0001	0.0001	0.0001	0.0001	-0.0647	0.6753	0.9889	none
PWY-6122: 5-aminoimidazole ribonucleotide biosynthesis II	0.0001	0.0001	0.0001	0.0001	-0.0647	0.6753	0.9889	none
PWY-6123: inosine-5'-phosphate biosynthesis I	0.0002	0.0002	0.0002	0.0002	-0.0685	0.6571	0.9889	none
PWY-6124: inosine-5'-phosphate biosynthesis II	0.0000	0.0000	0.0000	0.0000	0.1110	0.4722	0.9889	none
PWY-6125: superpathway of guanosine nucleotides de novo biosynthesis II	0.0001	0.0001	0.0001	0.0001	-0.1150	0.4569	0.9889	none
PWY-6126: superpathway of adenosine nucleotides de novo biosynthesis II	0.0001	0.0001	0.0001	0.0001	-0.1090	0.4799	0.9889	none
PWY-6147: 6-hydroxymethyl-dihydropterin diphosphate biosynthesis I	0.0000	0.0000	0.0000	0.0000	0.0087	0.9551	0.9889	none
PWY-6151: S-adenosyl-L-methionine cycle I	0.0001	0.0001	0.0001	0.0001	0.0549	0.7216	0.9889	none
PWY-6163: chorismate biosynthesis from 3-dehydroquinate	0.0007	0.0006	0.0007	0.0006	-0.0318	0.8365	0.9889	none
PWY-621: sucrose degradation III (sucrose invertase)	0.0002	0.0002	0.0002	0.0002	-0.0068	0.9651	0.9889	none
PWY-6277: superpathway of 5-aminoimidazole ribonucleotide biosynthesis	0.0001	0.0001	0.0001	0.0001	-0.0647	0.6753	0.9889	none
PWY-6282: palmitoleate biosynthesis I (from (5Z)-dodec-5-enoate)	0.0000	0.0000	0.0000	0.0000	-0.1420	0.3581	0.9889	none
PWY-6284: superpathway of unsaturated fatty acids biosynthesis (E. coli)	0.0000	0.0000	0.0000	0.0000	-0.0087	0.9551	0.9889	none
PWY-6285: superpathway of fatty acids biosynthesis (E. coli)	0.0000	0.0000	0.0000	0.0000	-0.0704	0.6481	0.9889	none
PWY-6305: putrescine biosynthesis IV	0.0001	0.0001	0.0001	0.0001	0.0549	0.7216	0.9889	none
PWY-6317: galactose degradation I (Leloir pathway)	0.0003	0.0003	0.0003	0.0003	0.0048	0.9751	0.9889	none
PWY-6353: purine nucleotides degradation II (aerobic)	0.0000	0.0000	0.0000	0.0000	-0.1820	0.2374	0.9889	none
PWY-6385: peptidoglycan biosynthesis III (mycobacteria)	0.0004	0.0004	0.0004	0.0004	-0.0029	0.9850	0.9889	none
PWY-6386: UDP-N-acetylmuramoyl-pentapeptide biosynthesis II (lysine-containing)	0.0000	0.0000	0.0000	0.0000	-0.0917	0.5526	0.9889	none
PWY-6387: UDP-N-acetylmuramoyl-pentapeptide biosynthesis I (meso-diaminopimelate co	0.0006	0.0006	0.0006	0.0006	-0.0492	0.7498	0.9889	none
PWY-6467: Kdo transfer to lipid IVA III (Chlamydia)	0.0000	0.0000	0.0000	0.0000	0.0318	0.8365	0.9889	none
PWY-6507: 4-deoxy-L-threo-hex-4-enopyranuronate degradation	0.0000	0.0000	0.0000	0.0000	0.2360	0.1256	0.9889	none
PWY-6519: 8-amino-7-oxononanoate biosynthesis I	0.0000	0.0000	0.0000	0.0000	0.0377	0.8074	0.9889	none
PWY-6527: stachyose degradation	0.0003	0.0003	0.0003	0.0003	-0.0184	0.9054	0.9889	none
PWY-6545: pyrimidine deoxyribonucleotides de novo biosynthesis III	0.0001	0.0000	0.0000	0.0000	-0.0588	0.7029	0.9889	none
PWY-6595: superpathway of guanosine nucleotides degradation (plants)	0.0000	0.0000	0.0000	0.0000	-0.0665	0.6662	0.9889	none
PWY-6606: guanosine nucleotides degradation II	0.0000	0.0000	0.0000	0.0000	-0.0665	0.6662	0.9889	none

PWY-6608: guanosine nucleotides degradation III	0.0000	0.0000	0.0000	0.0000	-0.2410	0.1195	0.9889	none
PWY-6609: adenine and adenosine salvage III	0.0001	0.0001	0.0000	0.0000	0.1560	0.3142	0.9889	none
PWY-6612: superpathway of tetrahydrofolate biosynthesis	0.0000	0.0000	0.0000	0.0000	-0.0029	0.9850	0.9889	none
PWY-6628: superpathway of L-phenylalanine biosynthesis	0.0000	0.0001	0.0000	0.0000	0.1680	0.2739	0.9889	none
PWY-6630: superpathway of L-tyrosine biosynthesis	0.0000	0.0000	0.0000	0.0000	0.0549	0.7216	0.9889	none
PWY-6690: cinnamate and 3-hydroxycinnamate degradation to 2-oxopent-4-enoate	0.0000	0.0000	0.0000	0.0000	0.0492	0.7498	0.9889	none
PWY-6700: queuosine biosynthesis	0.0000	0.0000	0.0000	0.0000	0.1990	0.1956	0.9889	none
PWY-6703: preQ0 biosynthesis	0.0000	0.0000	0.0000	0.0000	-0.0106	0.9452	0.9889	none
PWY-6708: ubiquinol-8 biosynthesis (prokaryotic)	0.0000	0.0000	0.0000	0.0000	0.3730	0.0155	0.6632	none
PWY-6737: starch degradation V	0.0007	0.0007	0.0007	0.0007	-0.0338	0.8268	0.9889	none
PWY-6803: phosphatidylcholine acyl editing	0.0000	0.0000	0.0000	0.0000	0.1090	0.4799	0.9889	none
PWY-6823: molybdenum cofactor biosynthesis	0.0000	0.0000	0.0000	0.0000	0.1170	0.4494	0.9889	none
PWY-6892: thiazole biosynthesis I (E. coli)	0.0000	0.0000	0.0000	0.0000	0.1460	0.3452	0.9889	none
PWY-6897: thiamin salvage II	0.0000	0.0000	0.0000	0.0000	-0.0917	0.5526	0.9889	none
PWY-6901: superpathway of glucose and xylose degradation	0.0000	0.0000	0.0000	0.0000	0.1910	0.2135	0.9889	none
PWY-6936: seleno-amino acid biosynthesis	0.0000	0.0000	0.0000	0.0000	-0.0395	0.7977	0.9889	none
PWY-6969: TCA cycle V (2-oxoglutarate:ferredoxin oxidoreductase)	0.0001	0.0001	0.0001	0.0001	-0.0377	0.8074	0.9889	none
PWY-7013: L-1,2-propanediol degradation	0.0000	0.0000	0.0000	0.0000	-0.0608	0.6937	0.9889	none
PWY-7039: phosphatidate metabolism, as a signaling molecule	0.0000	0.0000	0.0000	0.0000	0.1360	0.3780	0.9889	none
PWY-7111: pyruvate fermentation to isobutanol (engineered)	0.0004	0.0004	0.0004	0.0004	-0.0704	0.6481	0.9889	none
PWY-7184: pyrimidine deoxyribonucleotides de novo biosynthesis I	0.0001	0.0000	0.0000	0.0000	-0.0647	0.6753	0.9889	none
PWY-7187: pyrimidine deoxyribonucleotides de novo biosynthesis II	0.0001	0.0001	0.0001	0.0001	-0.0917	0.5526	0.9889	none
PWY-7199: pyrimidine deoxyribonucleosides salvage	0.0001	0.0001	0.0001	0.0001	-0.0029	0.9850	0.9889	none
PWY-7204: pyridoxal 5'-phosphate salvage II (plants)	0.0000	0.0000	0.0000	0.0000	0.0415	0.7881	0.9889	none
PWY-7208: superpathway of pyrimidine nucleobases salvage	0.0002	0.0002	0.0001	0.0001	-0.1600	0.2965	0.9889	none
PWY-7210: pyrimidine deoxyribonucleotides biosynthesis from CTP	0.0000	0.0000	0.0000	0.0000	-0.0665	0.6662	0.9889	none
PWY-7211: superpathway of pyrimidine deoxyribonucleotides de novo biosynthesis	0.0001	0.0001	0.0001	0.0000	-0.0377	0.8074	0.9889	none
PWY-7219: adenosine ribonucleotides de novo biosynthesis	0.0012	0.0012	0.0012	0.0011	-0.0318	0.8365	0.9889	none
PWY-7220: adenosine deoxyribonucleotides de novo biosynthesis II	0.0001	0.0001	0.0001	0.0000	-0.1010	0.5115	0.9889	none
PWY-7221: guanosine ribonucleotides de novo biosynthesis	0.0005	0.0004	0.0004	0.0004	-0.1210	0.4345	0.9889	none
PWY-7222: guanosine deoxyribonucleotides de novo biosynthesis II	0.0001	0.0001	0.0001	0.0000	-0.1010	0.5115	0.9889	none
PWY-7228: superpathway of guanosine nucleotides de novo biosynthesis I	0.0003	0.0003	0.0003	0.0003	-0.1030	0.5035	0.9889	none
PWY-7229: superpathway of adenosine nucleotides de novo biosynthesis I	0.0001	0.0001	0.0001	0.0001	-0.0994	0.5196	0.9889	none
PWY-7237: myo-, chiro- and scillo-inositol degradation	0.0001	0.0001	0.0000	0.0000	-0.0878	0.5694	0.9889	none
PWY-7242: D-fructuronate degradation	0.0002	0.0001	0.0001	0.0001	0.0029	0.9850	0.9889	none
PWY-724: superpathway of L-lysine, L-threonine and L-methionine biosynthesis II	0.0004	0.0004	0.0004	0.0004	-0.0665	0.6662	0.9889	none
PWY-7269: NAD/NADP-NADH/NADPH mitochondrial interconversion (yeast)	0.0000	0.0000	0.0000	0.0000	0.0819	0.5951	0.9889	none
PWY-7282: 4-amino-2-methyl-5-phosphomethylpyrimidine biosynthesis (yeast)	0.0003	0.0003	0.0003	0.0003	-0.0917	0.5526	0.9889	none

PWY-7315: dTDP-N-acetylthomosamine biosynthesis	0.0000	0.0000	0.0000	0.0000	0.1840	0.2324	0.9889	none
PWY-7328: superpathway of UDP-glucose-derived O-antigen building blocks biosynthesis	0.0000	0.0000	0.0000	0.0000	-0.1600	0.2965	0.9889	none
PWY-7357: thiamin formation from pyrithiamine and oxythiamine (yeast)	0.0000	0.0000	0.0000	0.0000	-0.0819	0.5951	0.9889	none
PWY-7371: 1,4-dihydroxy-6-naphthoate biosynthesis II	0.0000	0.0000	0.0000	0.0000	-0.1340	0.3848	0.9889	none
PWY-7383: anaerobic energy metabolism (invertebrates, cytosol)	0.0000	0.0000	0.0000	0.0000	0.1030	0.5035	0.9889	none
PWY-7400: L-arginine biosynthesis IV (archaebacteria)	0.0002	0.0002	0.0002	0.0002	-0.0955	0.5360	0.9889	none
PWY-7446: sulfoglycolysis	0.0000	0.0000	0.0000	0.0000	0.0434	0.7785	0.9889	none
PWY-7663: gondoate biosynthesis (anaerobic)	0.0001	0.0000	0.0000	0.0000	-0.1230	0.4272	0.9889	none
PWY-7664: oleate biosynthesis IV (anaerobic)	0.0000	0.0000	0.0000	0.0000	0.0549	0.7216	0.9889	none
PWY-841: superpathway of purine nucleotides de novo biosynthesis I	0.0000	0.0000	0.0000	0.0000	0.0801	0.6038	0.9889	none
PWY0-1061: superpathway of L-alanine biosynthesis	0.0000	0.0000	0.0000	0.0000	0.0839	0.5865	0.9889	none
PWY0-1241: ADP-L-glycero-β-D-manno-heptose biosynthesis	0.0000	0.0000	0.0000	0.0000	0.1980	0.2000	0.9889	none
PWY0-1261: anhydromuropeptides recycling	0.0001	0.0001	0.0001	0.0001	-0.0549	0.7216	0.9889	none
PWY0-1277: 3-phenylpropanoate and 3-(3-hydroxyphenyl)propanoate degradation	0.0000	0.0000	0.0000	0.0000	0.0261	0.8660	0.9889	none
PWY0-1296: purine ribonucleosides degradation	0.0001	0.0001	0.0001	0.0001	0.1460	0.3452	0.9889	none
PWY0-1297: superpathway of purine deoxyribonucleosides degradation	0.0000	0.0000	0.0000	0.0000	-0.0955	0.5360	0.9889	none
PWY0-1298: superpathway of pyrimidine deoxyribonucleosides degradation	0.0000	0.0000	0.0000	0.0000	-0.0974	0.5278	0.9889	none
PWY0-1319: CDP-diacylglycerol biosynthesis II	0.0000	0.0000	0.0000	0.0000	0.0878	0.5694	0.9889	none
PWY0-1338: polymyxin resistance	0.0000	0.0000	0.0000	0.0000	0.0878	0.5694	0.9889	none
PWY0-1415: superpathway of heme biosynthesis from uroporphyrinogen-III	0.0000	0.0000	0.0000	0.0000	-0.0299	0.8463	0.9889	none
PWY0-1479: tRNA processing	0.0000	0.0000	0.0000	0.0000	-0.1530	0.3202	0.9889	none
PWY0-1533: methylphosphonate degradation I	0.0000	0.0000	0.0000	0.0000	0.1990	0.1956	0.9889	none
PWY0-1586: peptidoglycan maturation (meso-diaminopimelate containing)	0.0002	0.0002	0.0002	0.0002	0.0164	0.9154	0.9889	none
PWY0-162: superpathway of pyrimidine ribonucleotides de novo biosynthesis	0.0002	0.0001	0.0002	0.0001	-0.1640	0.2907	0.9889	none
PWY0-166: superpathway of pyrimidine deoxyribonucleotides de novo biosynthesis (E. coli)	0.0003	0.0003	0.0003	0.0002	-0.1070	0.4877	0.9889	none
PWY0-781: aspartate superpathway	0.0001	0.0001	0.0000	0.0000	0.1740	0.2578	0.9889	none
PWY0-845: superpathway of pyridoxal 5'-phosphate biosynthesis and salvage	0.0001	0.0001	0.0001	0.0001	0.1280	0.4057	0.9889	none
PWY0-862: (5Z)-dodec-5-enoate biosynthesis	0.0000	0.0000	0.0000	0.0000	0.0935	0.5442	0.9889	none
PWY4FS-7: phosphatidylglycerol biosynthesis I (plastidic)	0.0000	0.0000	0.0000	0.0000	0.0472	0.7593	0.9889	none
PWY4FS-8: phosphatidylglycerol biosynthesis II (non-plastidic)	0.0000	0.0000	0.0000	0.0000	0.0472	0.7593	0.9889	none
PWY66-400: glycolysis VI (metazoan)	0.0001	0.0001	0.0001	0.0000	-0.0801	0.6038	0.9889	none
PWY66-422: D-galactose degradation V (Leloir pathway)	0.0003	0.0003	0.0003	0.0003	-0.0048	0.9751	0.9889	none
PWYG-321: mycolate biosynthesis	0.0000	0.0000	0.0000	0.0000	-0.1340	0.3848	0.9889	none
PYRIDNUCSAL-PWY: NAD salvage pathway I	0.0000	0.0000	0.0000	0.0000	0.1980	0.2000	0.9889	none
PYRIDNUCSYN-PWY: NAD biosynthesis I (from aspartate)	0.0002	0.0002	0.0002	0.0002	-0.0377	0.8074	0.9889	none
PYRIDOXSYN-PWY: pyridoxal 5'-phosphate biosynthesis I	0.0001	0.0001	0.0000	0.0000	0.1590	0.3023	0.9889	none
REDCITCYC: TCA cycle VIII (helicobacter)	0.0000	0.0000	0.0000	0.0000	0.0704	0.6481	0.9889	none
RHAMCAT-PWY: L-rhamnose degradation I	0.0002	0.0001	0.0001	0.0001	-0.0974	0.5278	0.9889	none

RIBOSYN2-PWY: flavin biosynthesis I (bacteria and plants)	0.0001	0.0001	0.0000	0.0001	-0.1420	0.3581	0.9889	none
SALVADEHYPOX-PWY: adenosine nucleotides degradation II	0.0000	0.0000	0.0000	0.0000	-0.0029	0.9850	0.9889	none
SER-GLYSYN-PWY: superpathway of L-serine and glycine biosynthesis I	0.0000	0.0000	0.0000	0.0000	-0.0511	0.7404	0.9889	none
SO4ASSIM-PWY: sulfate reduction I (assimilatory)	0.0000	0.0000	0.0000	0.0000	0.0202	0.8955	0.9889	none
SULFATE-CYS-PWY: superpathway of sulfate assimilation and cysteine biosynthesis	0.0000	0.0000	0.0000	0.0000	0.0164	0.9154	0.9889	none
TCA-GLYOX-BYPASS: superpathway of glyoxylate bypass and TCA	0.0000	0.0000	0.0000	0.0000	0.0318	0.8365	0.9889	none
TCA: TCA cycle I (prokaryotic)	0.0001	0.0001	0.0001	0.0001	-0.0202	0.8955	0.9889	none
THISYN-PWY: superpathway of thiamin diphosphate biosynthesis I	0.0001	0.0001	0.0000	0.0000	0.1240	0.4200	0.9889	none
THISYNARA-PWY: superpathway of thiamin diphosphate biosynthesis III (eukaryotes)	0.0002	0.0002	0.0001	0.0002	-0.1320	0.3917	0.9889	none
THRESYN-PWY: superpathway of L-threonine biosynthesis	0.0003	0.0003	0.0003	0.0003	-0.0395	0.7977	0.9889	none
TRNA-CHARGING-PWY: tRNA charging	0.0000	0.0000	0.0000	0.0000	-0.1150	0.4569	0.9889	none
UBISYN-PWY: superpathway of ubiquinol-8 biosynthesis (prokaryotic)	0.0000	0.0000	0.0000	0.0000	0.3920	0.0109	0.6632	none
UDPNAGSYN-PWY: UDP-N-acetyl-D-glucosamine biosynthesis I	0.0001	0.0001	0.0001	0.0000	0.0222	0.8857	0.9889	none
VALSYN-PWY: L-valine biosynthesis	0.0004	0.0004	0.0004	0.0004	0.0048	0.9751	0.9889	none

ESM Table 7: Comparisons of Clr-transformed relative abundances of species between acarbose (Acar) and vlidagaliptin (Vlid) at 6 month

Species	Occurrence		Mean rank		Acar vs. Vlid (Wilcoxon rank sum test, clr-transformed)			Enrichment (Base vs. M6)	
	Acar	Vlid	Acar	Vlid	P-value	BH adjusted P-value	Enrichment	Acar	Vlid
Acidaminococcus_intestini	0.4048	0.1951	50.9048	32.8780	0.0007	0.0024	Vlid < Acar	M6	none
Adlercreutzia_equolifaciens	0.2143	0.6098	34.0714	50.1220	0.0024	0.0069	Acar < Vlid	base	none
Akkermansia_muciniphila	0.1190	0.3659	41.5714	42.4390	0.8698	0.8927	Acar = Vlid	none	none
Alistipes_finegoldii	0.1667	0.6341	32.5714	51.6585	0.0003	0.0014	Acar < Vlid	base	base
Alistipes_indistinctus	0.1190	0.4634	37.4524	46.6585	0.0819	0.1245	Acar = Vlid	base	none
Alistipes_onderdonkii	0.2857	0.6341	34.1429	50.0488	0.0026	0.0072	Acar < Vlid	base	none
Alistipes_putredinis	0.3333	0.7073	32.9762	51.2439	0.0006	0.0022	Acar < Vlid	base	none
Alistipes_senegalensis	0.0714	0.4390	37.5476	46.5610	0.0885	0.1328	Acar = Vlid	base	base
Alistipes_shahii	0.2143	0.7317	29.9524	54.3415	0.0000	0.0000	Acar < Vlid	base	none
Anaerostipes_hadrus	0.7857	0.8049	44.1905	39.7561	0.4021	0.5113	Vlid = Acar	M6	none
Bacteroidales_bacterium_ph8	0.0952	0.5610	34.0000	50.1951	0.0022	0.0065	Acar < Vlid	base	none
Bacteroides_caccae	0.5238	0.6585	37.4048	46.7073	0.0788	0.1229	Acar = Vlid	base	base
Bacteroides_cellulosilyticus	0.1667	0.5610	35.1429	49.0244	0.0087	0.0200	Acar < Vlid	base	none
Bacteroides_coprocola	0.3571	0.3902	44.0952	39.8537	0.4228	0.5263	Vlid = Acar	none	none
Bacteroides_dorei	0.5714	0.6098	41.0714	42.9512	0.7224	0.8050	Acar = Vlid	base	none
Bacteroides_eggerthii	0.1429	0.2927	43.6667	40.2927	0.5237	0.6253	Vlid = Acar	base	base
Bacteroides_faecis	0.1429	0.4146	40.3571	43.6829	0.5297	0.6260	Acar = Vlid	base	none
Bacteroides_finegoldii	0.2381	0.4878	39.0238	45.0488	0.2549	0.3351	Acar = Vlid	base	base
Bacteroides_fragilis	0.7619	0.6585	44.1905	39.7561	0.4021	0.5113	Vlid = Acar	none	none
Bacteroides_intestinalis	0.0714	0.3659	40.1905	43.8537	0.4888	0.5957	Acar = Vlid	none	none
Bacteroides_massiliensis	0.2857	0.6098	34.4524	49.7317	0.0039	0.0095	Acar < Vlid	base	none
Bacteroides_nordii	0.1190	0.4878	36.5000	47.6341	0.0354	0.0637	Acar = Vlid	base	none
Bacteroides_ovatus	0.7619	0.9512	33.2857	50.9268	0.0009	0.0027	Acar < Vlid	base	none
Bacteroides_plebeius	0.5000	0.6341	38.0238	46.0732	0.1282	0.1808	Acar = Vlid	base	base
Bacteroides_salyersiae	0.0952	0.3171	42.6429	41.3415	0.8057	0.8645	Vlid = Acar	none	none
Bacteroides_stercoris	0.6667	0.9024	29.9048	54.3902	0.0000	0.0000	Acar < Vlid	base	base
Bacteroides_thetaiotaomicron	0.5714	0.9268	28.9048	55.4146	0.0000	0.0000	Acar < Vlid	base	base
Bacteroides_uniformis	0.6667	0.8780	31.0952	53.1707	0.0000	0.0003	Acar < Vlid	base	none
Bacteroides_vulgatus	0.9286	0.9268	36.1190	48.0244	0.0245	0.0494	Acar < Vlid	base	none
Bacteroides_xylanisolvens	0.4048	0.8780	29.0714	55.2439	0.0000	0.0000	Acar < Vlid	base	base

Barnesiella_intestinihominis	0.1905	0.4634	38.8333	45.2439	0.2257	0.3001	Acar = Vlid	base	none
Bifidobacterium_adolescentis	0.6667	0.5854	47.5000	36.3659	0.0354	0.0637	Vlid = Acar	M6	M6
Bifidobacterium_bifidum	0.5952	0.2195	54.0952	29.6098	0.0000	0.0000	Vlid < Acar	M6	none
Bifidobacterium_longum	0.9286	0.8049	57.3571	26.2683	0.0000	0.0000	Vlid < Acar	M6	none
Bifidobacterium_pseudocatenulatu	0.7619	0.6341	51.7143	32.0488	0.0002	0.0010	Vlid < Acar	M6	none
Bilophila_wadsworthia	0.1667	0.6585	32.2143	52.0244	0.0002	0.0010	Acar < Vlid	base	none
Burkholderiales_bacterium_1_1_47	0.6429	0.8049	41.4762	42.5366	0.8412	0.8710	Acar = Vlid	base	none
candidate_division_TM7_single_cel	0.3571	0.3415	46.0476	37.8537	0.1215	0.1755	Vlid = Acar	M6	none
Citrobacter_freundii	0.1667	0.3659	42.5238	41.4634	0.8412	0.8710	Vlid = Acar	none	none
Clostridiales_bacterium_1_7_47FA	0.2143	0.4390	40.7857	43.2439	0.6423	0.7440	Acar = Vlid	none	none
Clostridium_asparagiforme	0.0952	0.4390	38.2619	45.8293	0.1527	0.2078	Acar = Vlid	base	none
Clostridium_bartlettii	0.5952	0.8537	35.0714	49.0976	0.0080	0.0188	Acar < Vlid	none	M6
Clostridium_bolteae	0.1667	0.8293	29.5000	54.8049	0.0000	0.0000	Acar < Vlid	base	none
Clostridium_citroniae	0.1429	0.5122	36.6905	47.4390	0.0422	0.0738	Acar = Vlid	base	none
Clostridium_hathewayi	0.1905	0.4878	39.8333	44.2195	0.4072	0.5123	Acar = Vlid	base	none
Clostridium_leptum	0.0952	0.3415	41.1905	42.8293	0.7568	0.8353	Acar = Vlid	none	none
Clostridium_nexile	0.1429	0.2683	45.9286	37.9756	0.1329	0.1851	Vlid = Acar	none	none
Clostridium_perfringens	0.3333	0.2195	48.7143	35.1220	0.0102	0.0230	Vlid < Acar	M6	none
Clostridium_ramosum	0.2381	0.2195	47.7857	36.0732	0.0269	0.0516	Vlid = Acar	none	none
Clostridium_symbiosum	0.1905	0.3415	42.6667	41.3171	0.7987	0.8645	Vlid = Acar	base	none
Collinsella_aerofaciens	0.6667	0.7073	46.9762	36.9024	0.0570	0.0926	Vlid = Acar	M6	none
Coprococcus_catus	0.5476	0.7561	37.9762	46.1220	0.1237	0.1765	Acar = Vlid	base	none
Coprococcus_comes	0.5952	0.8049	37.2381	46.8780	0.0685	0.1083	Acar = Vlid	none	none
Dialister_invisus	0.2619	0.3902	43.5238	40.4390	0.5599	0.6551	Vlid = Acar	none	none
Dorea_formicigenerans	0.7381	0.8293	41.2619	42.7561	0.7777	0.8504	Acar = Vlid	none	none
Dorea_longicatena	0.8333	0.9024	36.9048	47.2195	0.0513	0.0857	Acar = Vlid	none	none
Enterobacter_cloacae	0.2857	0.4634	42.6190	41.3659	0.8128	0.8645	Vlid = Acar	none	none
Enterococcus_faecium	0.4048	0.2927	48.0952	35.7561	0.0197	0.0412	Vlid < Acar	M6	none
Escherichia_coli	1.0000	1.0000	45.7857	38.1220	0.1476	0.2031	Vlid = Acar	none	none
Eubacterium_biforme	0.2619	0.2195	47.8333	36.0244	0.0256	0.0500	Vlid = Acar	M6	none
Eubacterium_eligens	0.5238	0.7561	36.8333	47.2927	0.0481	0.0816	Acar = Vlid	none	none
Eubacterium_hallii	0.6905	0.8780	33.2381	50.9756	0.0008	0.0027	Acar < Vlid	base	none
Eubacterium_ramulus	0.4286	0.8293	33.2857	50.9268	0.0009	0.0027	Acar < Vlid	base	none
Eubacterium_rectale	0.8810	0.9512	37.0238	47.0976	0.0570	0.0926	Acar = Vlid	none	none
Eubacterium_ventriosum	0.2619	0.7561	33.0000	51.2195	0.0006	0.0022	Acar < Vlid	base	none
Faecalibacterium_prausnitzii	0.9286	0.9756	36.4762	47.6585	0.0346	0.0637	Acar = Vlid	none	none
Flavonifractor_plautii	0.3333	0.7317	35.3095	48.8537	0.0105	0.0231	Acar < Vlid	base	none
Haemophilus_parainfluenzae	0.8333	0.6341	47.3333	36.5366	0.0413	0.0733	Vlid = Acar	M6	none

Holdemania_filiformis	0.0714	0.6341	31.3095	52.9512	0.0000	0.0003	Acar < Vlid	none	none
Klebsiella_pneumoniae	0.5952	0.6829	42.5714	41.4146	0.8270	0.8710	Vlid = Acar	none	none
Lachnospiraceae_bacterium_1_1_5	0.4524	0.8780	28.8571	55.4634	0.0000	0.0000	Acar < Vlid	base	none
Lachnospiraceae_bacterium_1_4_5	0.1429	0.5122	37.6667	46.4390	0.0974	0.1442	Acar = Vlid	base	none
Lachnospiraceae_bacterium_2_1_5	0.5000	0.4634	46.8810	37.0000	0.0619	0.0992	Vlid = Acar	none	none
Lachnospiraceae_bacterium_3_1_4	0.2381	0.7073	32.2381	52.0000	0.0002	0.0010	Acar < Vlid	base	none
Lachnospiraceae_bacterium_5_1_6	0.8810	0.9268	42.9762	41.0000	0.7088	0.8050	Vlid = Acar	M6	none
Lachnospiraceae_bacterium_7_1_5	0.0952	0.6341	31.6667	52.5854	0.0001	0.0005	Acar < Vlid	base	none
Lachnospiraceae_bacterium_8_1_5	0.4286	0.1707	51.1905	32.5854	0.0004	0.0018	Vlid < Acar	M6	none
Lactobacillus_mucosae	0.8810	0.2439	59.9762	23.5854	0.0000	0.0000	Vlid < Acar	M6	none
Lactobacillus_salivarius	0.4286	0.1463	52.5000	31.2439	0.0001	0.0004	Vlid < Acar	M6	none
Megamonas_funiformis	0.3571	0.2927	47.9286	35.9268	0.0233	0.0479	Vlid < Acar	none	none
Megamonas_hypermegale	0.4524	0.3659	47.2619	36.6098	0.0441	0.0759	Vlid = Acar	none	none
Megamonas_rupellensis	0.4524	0.2439	51.5714	32.1951	0.0003	0.0012	Vlid < Acar	none	none
Megasphaera_elsdenii	0.4048	0.2927	48.2857	35.5610	0.0162	0.0344	Vlid < Acar	M6	none
Odoribacter_splanchnicus	0.0952	0.5366	34.4762	49.7073	0.0040	0.0095	Acar < Vlid	base	base
Oxalobacter_formigenes	0.0000	0.3171	40.2857	43.7561	0.5120	0.6175	Acar = Vlid	base	none
Parabacteroides_distasonis	0.6429	0.7317	37.8333	46.2683	0.1109	0.1623	Acar = Vlid	base	base
Parabacteroides_goldsteinii	0.1190	0.2439	45.1905	38.7317	0.2223	0.2989	Vlid = Acar	none	none
Parabacteroides_merdae	0.4048	0.7805	32.6190	51.6098	0.0003	0.0014	Acar < Vlid	base	none
Paraprevotella_clara	0.0952	0.3902	39.5238	44.5366	0.3435	0.4466	Acar = Vlid	none	base
Paraprevotella_xylaniphila	0.0238	0.3659	40.0714	43.9756	0.4607	0.5673	Acar = Vlid	none	base
Parasutterella_excrementihominis	0.6667	0.7805	41.8333	42.1707	0.9492	0.9492	Acar = Vlid	base	none
Prevotella_copri	0.8333	0.8780	42.9524	41.0244	0.7156	0.8050	Vlid = Acar	none	none
Pyramidobacter_piscolens	0.0952	0.3171	43.1429	40.8293	0.6620	0.7593	Vlid = Acar	none	none
Roseburia_hominis	0.2381	0.6829	33.2381	50.9756	0.0008	0.0027	Acar < Vlid	base	none
Roseburia_intestinalis	0.3095	0.7561	32.1667	52.0732	0.0002	0.0010	Acar < Vlid	base	none
Roseburia_inulinivorans	0.4762	0.9024	30.2857	54.0000	0.0000	0.0001	Acar < Vlid	base	none
Rothia_mucilaginosa	0.6667	0.5366	49.8095	34.0000	0.0028	0.0075	Vlid < Acar	M6	none
Ruminococcus_bromii	0.4286	0.7317	36.1667	47.9756	0.0256	0.0500	Acar = Vlid	base	none
Ruminococcus_callidus	0.1667	0.6098	33.8810	50.3171	0.0019	0.0057	Acar < Vlid	base	none
Ruminococcus_gnavus	0.7143	0.7561	41.6429	42.3659	0.8913	0.9068	Acar = Vlid	none	none
Ruminococcus_lactaris	0.1905	0.3902	41.8095	42.1951	0.9419	0.9492	Acar = Vlid	none	none
Ruminococcus_obeum	0.9048	1.0000	32.8810	51.3415	0.0005	0.0020	Acar < Vlid	base	none
Ruminococcus_sp_5_1_39BFAA	0.1905	0.6341	34.4286	49.7561	0.0038	0.0094	Acar < Vlid	base	none
Ruminococcus_torques	0.9286	1.0000	37.4524	46.6585	0.0819	0.1245	Acar = Vlid	none	none
Solobacterium_moorei	0.4524	0.2195	52.3095	31.4390	0.0001	0.0005	Vlid < Acar	M6	none
Streptococcus_anginosus	0.4762	0.1707	52.0714	31.6829	0.0001	0.0007	Vlid < Acar	M6	none

Streptococcus_australis	0.5952	0.5122	47.6905	36.1707	0.0295	0.0557	Vlid = Acar	M6	none
Streptococcus_infantis	0.3810	0.0976	54.0476	29.6585	0.0000	0.0000	Vlid < Acar	M6	none
Streptococcus_mitis_oralis_pneumo	0.6190	0.3171	51.6667	32.0976	0.0002	0.0011	Vlid < Acar	M6	none
Streptococcus_parasanguinis	0.6667	0.4634	49.7857	34.0244	0.0029	0.0075	Vlid < Acar	M6	none
Streptococcus_salivarius	0.9762	0.7805	54.4286	29.2683	0.0000	0.0000	Vlid < Acar	M6	none
Streptococcus_sanguinis	0.4762	0.2927	50.8333	32.9512	0.0007	0.0026	Vlid < Acar	M6	none
Streptococcus_thermophilus	0.3095	0.2195	49.7143	34.0976	0.0032	0.0081	Vlid < Acar	M6	none
Streptococcus_vestibularis	0.5714	0.1707	54.3810	29.3171	0.0000	0.0000	Vlid < Acar	M6	none
Veillonella_atypica	0.6667	0.3902	50.2857	33.5122	0.0015	0.0047	Vlid < Acar	M6	none
Veillonella_dispar	0.4524	0.2683	49.9048	33.9024	0.0025	0.0070	Vlid < Acar	M6	none
Veillonella_parvula	0.9286	0.7561	48.4048	35.4390	0.0143	0.0309	Vlid < Acar	M6	none

ESM Table 8: Longitudinal associations between clinical variables and microbial species after adjustment for BMI and L2-L3 VFA

ESM Table 8a: Longitudinal associations between clinical variables and microbial species after adjustment for BMI and L2-L3 VFA in the acarbose group.

	HbA$_{1c}$		FPG		PPG		HOMA-IR		leptin		CCK		GLP--1	
	Wald	P.adjust	Wald	P.adjust	Wald	P.adjust	Wald	P.adjust	Wald	P.adjust	Wald	P.adjust	Wald	P.adjust
Bifidobacterium_adolescentis	-5.1033	0.0000	-3.6831	0.0002	-4.7959	0.0000	-0.7153	0.4744	-2.0737	0.0381	1.5340	0.1250	-0.7786	0.4362
Bacteroides_caccae	3.7601	0.0002	0.1656	0.8685	2.2385	0.0252	0.0295	0.9765	2.0050	0.0450	0.0129	0.9897	-1.1288	0.2590
Bacteroides_eggerthii	0.4576	0.6472	0.7442	0.4567	0.7540	0.4508	-0.4868	0.6264	-0.3723	0.7097	-0.6605	0.5089	1.2378	0.2158
Bacteroides_plebeius	0.9294	0.3527	0.1339	0.8935	-0.0697	0.9444	-2.1240	0.0337	0.9688	0.3326	-0.5620	0.5741	0.2311	0.8172
Bacteroides_thetaiotaomicron	5.1791	0.0000	1.2314	0.2182	3.9209	0.0001	0.9886	0.3228	3.7448	0.0002	-1.7424	0.0814	-0.9029	0.3666
Parabacteroides_distasonis	3.2545	0.0011	1.9716	0.0487	2.7734	0.0055	-0.3097	0.7568	2.0187	0.0435	0.3002	0.7640	0.1951	0.8453
Bifidobacterium_longum	-5.8189	0.0000	-1.3790	0.1679	-6.1510	0.0000	-0.0161	0.9871	-2.2145	0.0268	0.8643	0.3874	0.6545	0.5128
Bacteroides_cellulosilyticus	1.6095	0.1075	-0.0662	0.9472	0.4083	0.6831	-1.4207	0.1554	-0.0428	0.9659	-1.3084	0.1907	0.5024	0.6154
Bacteroides_finegoldii	1.4486	0.1475	2.2210	0.0264	1.7526	0.0797	-0.5623	0.5739	2.1492	0.0316	0.0115	0.9908	-0.3063	0.7594
Bacteroides_massiliensis	0.4120	0.6804	0.5233	0.6008	0.9999	0.3173	-0.5176	0.6047	-0.5620	0.5741	-1.9644	0.0495	-0.8069	0.4197
Bacteroides_stercoris	2.6381	0.0083	0.2746	0.7836	1.6476	0.0994	-0.7107	0.4773	2.2531	0.0243	-1.6425	0.1005	-0.8861	0.3755
Bacteroides_uniformis	3.9004	0.0001	0.4401	0.6599	2.6768	0.0074	1.8083	0.0706	1.8002	0.0718	-3.0655	0.0022	-0.0804	0.9359
Bacteroides_xylanisolvens	5.6808	0.0000	-0.8283	0.4075	4.0025	0.0001	0.2514	0.8015	1.8980	0.0577	-2.6689	0.0076	-0.9303	0.3522
Odoribacter_splanchnicus	3.2332	0.0012	2.0880	0.0368	2.1770	0.0295	-0.7046	0.4810	0.6371	0.5241	-0.4397	0.6601	-0.3285	0.7425
Parabacteroides_merdae	3.4259	0.0006	1.3204	0.1867	2.5577	0.0105	0.1853	0.8530	1.1344	0.2566	-1.8196	0.0688	-0.0478	0.9619
Alistipes_finegoldii	2.6684	0.0076	1.3066	0.1914	1.2510	0.2109	-0.7665	0.4434	0.8231	0.4104	-1.2401	0.2149	0.3991	0.6898
Alistipes_senegalensis	2.6298	0.0085	1.5211	0.1282	1.7616	0.0781	-0.9331	0.3508	0.2490	0.8033	-1.8616	0.0627	0.8364	0.4029
Alistipes_shahii	3.1192	0.0018	-0.2054	0.8372	1.2841	0.1991	-1.5328	0.1253	1.6035	0.1088	-1.5485	0.1215	-0.0383	0.9695
Haemophilus_parainfluenzae	-2.6522	0.0080	-1.6387	0.1013	-1.7843	0.0744	0.0004	0.9996	-1.0152	0.3100	0.4895	0.6245	0.0894	0.9288

ESM Table 8b: Longitudinal associations between clinical variables and microbial species after adjustment for BMI and L2-L3 VFA in the vildagliptin group.

	HbA$_{1c}$		FPG		PPG		HOMA-IR		leptin		CCK		GLP-1	
	Wald	P.adjust	Wald	P.adjust	Wald	P.adjust	Wald	P.adjust	Wald	P.adjust	Wald	P.adjust	Wald	P.adjust
Bifidobacterium_adolescentis	-2.4612	0.0138	-1.7329	0.0831	-2.4229	0.0154	-0.1671	0.8673	1.6938	0.0903	1.2070	0.2274	1.1738	0.2405
Bacteroides_caccae	0.4096	0.6821	0.2223	0.8241	0.5971	0.5504	-0.1177	0.9063	-0.3832	0.7016	-2.1735	0.0297	-3.0186	0.0025
Bacteroides_eggerthii	2.1180	0.0342	2.3444	0.0191	1.0397	0.2985	0.1166	0.9072	-0.6105	0.5415	-2.9971	0.0027	-2.8906	0.0038
Bacteroides_plebeius	3.1125	0.0019	2.4324	0.0150	3.6908	0.0002	1.1410	0.2539	0.4850	0.6277	-1.5994	0.1097	-2.8271	0.0047
Bacteroides_thetaiotaomicron	1.2393	0.2152	1.1788	0.2385	0.5286	0.5971	0.2603	0.7947	-0.9383	0.3481	-0.2069	0.8361	-1.6165	0.1060

Parabacteroides_distasonis	2.3042	0.0212	0.3837	0.7012	0.1641	0.8696	-0.7143	0.4751	-0.5784	0.5630	-2.5773	0.0100	-3.8788	0.0001
Bifidobacterium_longum	-1.5850	0.1130	-0.2187	0.8269	-0.1386	0.8897	1.0402	0.2983	-0.0483	0.9615	2.8476	0.0044	1.4104	0.1584
Bacteroides_cellulosilyticus	2.0748	0.0380	1.2748	0.2024	1.6179	0.1057	-0.1320	0.8950	-1.0132	0.3110	-2.9169	0.0035	-1.4357	0.1511
Bacteroides_finegoldii	1.0340	0.3011	2.6646	0.0077	1.2830	0.1995	1.4967	0.1345	0.9046	0.3657	-1.6836	0.0923	-1.6312	0.1028
Bacteroides_massiliensis	-0.5228	0.6011	-0.7158	0.4741	-0.7988	0.4244	-1.4644	0.1431	-0.6828	0.4947	-1.7009	0.0890	-0.5583	0.5766
Bacteroides_stercoris	0.3457	0.7295	-0.2269	0.8205	-0.0390	0.9689	-0.1606	0.8724	-0.3473	0.7284	-0.7285	0.4663	-1.1609	0.2457
Bacteroides_uniformis	0.8520	0.3942	0.8459	0.3976	0.2898	0.7719	-0.7031	0.4820	-0.9801	0.3271	-2.0040	0.0451	-2.6217	0.0087
Bacteroides_xylanisolvens	1.9881	0.0468	1.8929	0.0584	2.4964	0.0125	-0.4570	0.6477	-0.2353	0.8140	-0.7416	0.4583	-2.1386	0.0325
Odoribacter_splanchnicus	2.3639	0.0181	3.0988	0.0019	2.5711	0.0101	2.4466	0.0144	0.5731	0.5666	-2.6491	0.0081	-2.5659	0.0103
Parabacteroides_merdae	1.7054	0.0881	1.4003	0.1614	3.2281	0.0012	0.8851	0.3761	0.5820	0.5605	-0.6939	0.4878	-1.8381	0.0660
Alistipes_finegoldii	1.0358	0.3003	2.0739	0.0381	2.1814	0.0292	-0.2954	0.7677	1.0043	0.3152	-1.3772	0.1684	-2.2989	0.0215
Alistipes_senegalensis	2.5787	0.0099	2.4776	0.0132	2.3003	0.0214	0.6737	0.5005	0.4869	0.6263	-1.9899	0.0466	-2.8199	0.0048
Alistipes_shahii	1.8318	0.0670	1.2500	0.2113	0.7056	0.4804	-1.0251	0.3053	-0.6875	0.4917	-2.8207	0.0048	-1.3404	0.1801
Haemophilus_parainfluenzae	-2.0148	0.0439	-1.0179	0.3087	-1.8325	0.0669	0.7589	0.4479	-0.1356	0.8922	1.3321	0.1828	1.7734	0.0762

ESM Table 9: Comparisons of basline values and percentage change of clinical variables between T2D patients with low (LR) and high GLP-1 reponses (HR) to vildagliptin treatment

	Baseline		Percentage change from baseline	
	**beta (HR vs. LR)**	**P***	**beta (HR vs. LR)**	**P***
Age	0.0399	0.4897	0.0399	0.4897
Sex	0.7120	0.0351	NA	NA
GLP-1	0.0010	0.9961	1.1353	0.0004
Leptin	-6.0950	0.2493	0.4728	0.5065
Peptide YY	17.2297	0.2706	-0.0366	0.7867
Adiponectin	-2.0184	0.3682	0.2328	0.3037
Ghrelin	-2.6007	0.9831	0.0570	0.7251
Cholecystokinin	-6.6304	0.3416	5.5484	0.1335
HbA_{1c}	-0.4633	0.0250	0.0158	0.5637
FPG	-0.9246	0.0486	0.0117	0.8285
PPG	-3.0141	0.0007	0.1290	0.0413
Fins	-0.3557	0.8512	0.1411	0.6514
Pins	-2.4755	0.7273	0.4574	0.0240
HOMA-IR	-0.4688	0.4925	0.1687	0.5420
TG	0.1855	0.7445	0.0430	0.7450
LDL	-0.1511	0.5377	-0.0003	0.9972
HDL	-0.0666	0.3528	-0.0057	0.9091
L2-L3 VFA	-12.9222	0.3903	0.0390	0.4144
L4-L5 VFA	-14.6477	0.3020	0.0934	0.0823
Weight	0.5655	0.7868	-0.0066	0.6487
BMI	-0.0609	0.9152	-0.0066	0.6499

P value * from Analysis of covariance analysis adjust age, sex (exclude age , sex)

ESM Table 10: Major taxonomic contributors of the 6 selected pathways by sPLS-DA.

	Acar base	Acar M6	Vild_base	Vild_M6
**PWY-6285: superpathway of fatty acids biosynthesis**				
unclassfied	100.00%	100.00%	100.00%	100.00%
**PWY-6588: pyruvate fermentation to acetone**				
Lachnospiraceae_bacterium_5_1_63FAA	72.98%	54.32%	69.84%	66.44%
Anaerostipes_hadrus	27.02%	45.68%	30.16%	33.56%
**PWY-6270: isoprene biosynthesis I**				
Phascolarctobacterium_succinatutens	56.31%	48.81%	89.58%	81.91%
Klebsiella_pneumoniae	32.73%	47.76%	8.28%	10.48%
Klebsiella_variicola	0.00%	0.70%	0.00%	0.00%
Citrobacter_freundii	10.96%	2.54%	2.05%	7.61%
Enterobacter_cloacae	0.00%	0.18%	0.00%	0.00%
Salmonella_enterica	0.00%	0.00%	0.09%	0.00%
**PWY0-41: allantoin degradation IV (anaerobic)**				
unclassfied	100.00%	100.00%	100.00%	100.00%
**POLYAMINSYN3-PWY: superpathway of polyamine biosynthesis II**				
unclassfied	100.00%	100.00%	100.00%	100.00%
**NONOXIPENT-PWY: pentose phosphate pathway**				
Faecalibacterium_prausnitzii	27.50%	22.24%	23.52%	28.24%
Megamonas_funiformis	14.30%	12.24%	13.26%	22.45%
Megamonas_hypermegale	4.88%	11.22%	11.31%	9.88%
Megamonas_rupellensis	5.07%	8.76%	6.57%	4.79%
Eubacterium_eligens	5.50%	2.94%	4.66%	4.32%
Bacteroides_xylanisolvens	7.06%	1.45%	5.76%	3.36%
Ruminococcus_bromii	3.33%	3.33%	5.27%	3.71%
Bacteroides_thetaiotaomicron	6.00%	0.61%	5.02%	2.60%
Lachnospiraceae_bacterium_5_1_63FAA	1.56%	8.30%	1.81%	1.24%
Klebsiella_pneumoniae	3.61%	3.85%	1.35%	2.39%
Others	21.19%	25.06%	21.47%	17.02%

490
